# Supplementary material for: A practical fluorosulfonylating platform via photocatalytic imidazolium-based SO2F radical reagent
Source: Nat Commun. 2022 Jun 18;13:3515. doi: 10.1038/s41467-022-31296-2 (PMC9206656; doi:10.1038/s41467-022-31296-2)
Supplement: Supplementary file 1 — Supplementary Information [file 41467_2022_31296_MOESM1_ESM.pdf]

Supplementary Information

A practical fluorosulfonylating platform via photocatalytic imidazolium-based SO_2F radical reagent

Zhang & Li et al.

Supplementary Methods

I. General Methods

All reactions were performed in flame-dried glassware with magnetic stirring bar and sealed with a rubber septum. The solvents were distilled by standard methods. Reagents were obtained from commercial suppliers and used without further purification unless otherwise noted. Silica gel column chromatography was carried out using silica Gel 60 (230–400 mesh). Analytical thin layer chromatography (TLC) was done using silica Gel (silica gel 60 F254). TLC plates were analyzed by an exposure to ultraviolet (UV) light and/or submersion in phosphomolybdic acid solution or submersion in $KMnO_4$ solution or in I_2. NMR experiments were measured on a Bruker AVANCE III-400 or 500 spectrometer and carried out in chloroform-*d* ($CDCl_3$) or acetonitrile-d_3 (CD_3CN). ^{1}H NMR and ^{13}C NMR spectra were recorded at 400 MHz or 500 MHz and 100 MHz or 125 MHz spectrometers, respectively. ^{19}F NMR spectra were recorded at 376 MHz or 470 MHz spectrometers. Chemical shifts are reported as δ values relative to internal TMS (δ 0.00 for ^{1}H NMR), chloroform (δ 7.26 for ^{1}H NMR), acetonitrile (δ 1.94 for ^{1}H NMR), chloroform (δ 77.00 for ^{13}C NMR), and acetonitrile (δ 1.32 or 118.26 for ^{13}C NMR) in parts per million (ppm). The following abbreviations are used for the multiplicities: s: singlet, d: doublet, dd: doublet of doublet, t: triplet, q: quadruplet, m: multiplet, br: broad signal for proton spectra; Coupling constants (*J*) are reported in Hertz (Hz). Melting points were uncorrected. Infrared spectra were obtained on agilent Cary630. HRMS were recorded on a Bruker miccOTOF-Q111. GC-MS spectra were performed on Agilent 5977B.

Medium-sized screw-cap test tubes (8 mL) were used for all 0.10 mmol scale reactions: Fisher 13 x 100 mm tubes (Cat. No.1495935C)

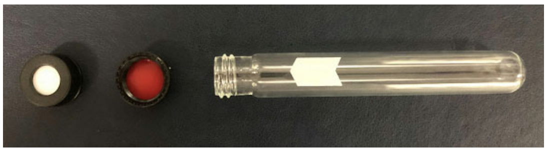

Supplementary Figure 1. Fisher 13 x 100 mm tubes

Cap with Septa: Thermo Scientific ASM PHN CAP w/PTFE/SIL (Cat. No.03378316)

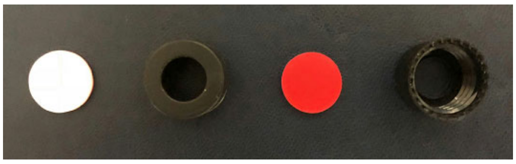

Supplementary Figure 2. Cap with Septa

II. Synthesis of Starting Materials

Substrates **1j-1u**, **1w, 1y-1z, 1aa-1ab** were purchased from commercial sources (Alfa, TCI, Energy and Macklin) and used as received.

Substrates **1a-1d** were prepared according to the literature.[1]

Substrates **2f** were prepared according to the literature.[2]

Substrates **5a-5k** were prepared according to the literature.[3]

Substrates **7a-7l** were prepared according to the literature.[4]

III. Synthesis of Sulfonyl fluoride imidazolium salt (2a-2e)

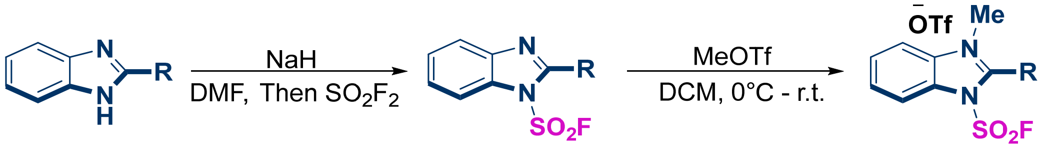

General Procedure:

1) Sodium hydride (60% dispersion in mineral oil.) (36 mmol, 1.2 equiv.) was added to corresponding imidazole (30 mmol, 1 equiv.) in N,N-Dimethylformamide (100 mL). The mixture was stirred at room temperature for 1 hour; A balloon volume of sulfuryl fluoride gas was then added to the reaction system. After the reaction was completed by TLC monitoring, the reaction mixture was evaporated in *vacuo*. Then, the reaction mixture was quenched with water and extracted with ethyl acetate (60 mL x 3). The organic layer was dried over Na_2SO_4, and evaporated in vacuo. The product was purified by flash column chromatography on silica gel with n-pentane/ethyl acetate as eluent to give the corresponding intermediate A.

2) To a solution of the corresponding intermediate A in DCM (50 mL) was added dropwise MeOTf (45 mmol) at 0 °C. Then, the mixture was stirred at room temperature for 12 hours, while monitoring by TLC. After that time, the mixture was concentrated under rotary evaporation to give a white solid (or a viscous liquid) crude product, to which *tert*-butyl methyl ether (30 mL) was added. With vigorous stirring, a solid precipitate was formed. The precipitate was washed with *tert*-butyl methyl ether (30 mL × 3) and dried in *vacuo* to yield the title compound (**2a-2e**) as a white solid.

1-(fluorosulfonyl)-3-methyl-2-phenyl-1H-benzo[d]imidazol-3-ium trifluoromethanesulfonate (2a)

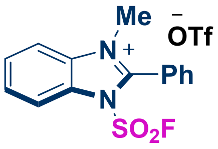

2a

73%, white solid: m.p. 169-170 °C; ^{1}H NMR (400 MHz, Acetonitrile-d_3) δ 8.24 – 8.17 (m, 1H), 8.15 – 8.06 (m, 1H), 8.03 – 7.91 (m, 2H), 7.91 – 7.85 (m, 3H), 7.83 – 7.72 (m, 2H), 3.95 (s, 3H). ^{13}C NMR (101 MHz, Acetonitrile-d_3) δ 154.2, 135.4, 132.7, 131.6, 131.4, 130.6, 130.4, 122.0 (q, J = 320.8 Hz), 120.79, 115.92, 115.90, 35.45. 120.8, 115.9, 115.9, 35.5. ^{19}F NMR (376 MHz, Acetonitrile-d_3) δ 64.76, -79.23. HRMS(ESI): caled for $C_{14}H_{12}FN_2O_2S^+$ $[M]^+$ 291.0598; found 291.0596.

1-(fluorosulfonyl)-3-methyl-2-(4-(trifluoromethyl)phenyl)-1H-benzo[d]imidazol-3-ium trifluoromethanesulfonate (2b)

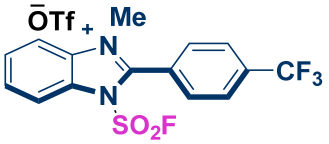

2b

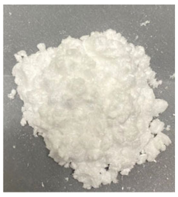

69%; white solid: m.p. 165-166 °C; ^{1}H NMR (400 MHz, Acetonitrile-d_3) δ 8.24 – 8.20 (m, 1H), 8.17 – 8.11 (m, 1H), 8.09 (d, J = 0.9 Hz, 4H), 8.03 – 7.95 (m, 2H), 3.96 (s, 3H). ^{13}C NMR (101 MHz, Acetonitrile-d_3) δ 152.5, 136.1 (q, J = 33.1 Hz), 132.8, 132.6, 132.0, 130.7, 130.6, 127.6 (q, J = 3.8 Hz), 125.9, 124.8, 123.1, 122.0 (q, J = 320.8 Hz), 116.0, 115.9, 35.6. ^{19}F NMR (376 MHz, Acetonitrile-d_3) δ 64.62, -63.88, -79.31.; HRMS(ESI): caled for $C_{15}H_{11}F_4N_2O_2S^+$ $[M]^+$ 359.0472; found 359.0471.

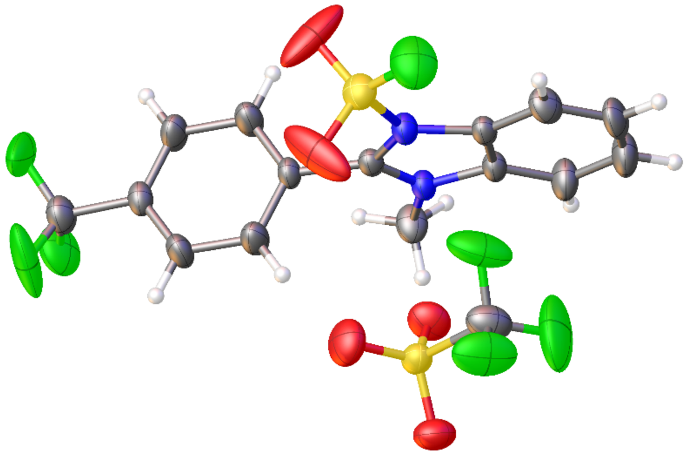

Supplementary Figure 3. X-ray crystallography for **2b** (CCDC number: 2164689)

2-(2-chlorophenyl)-1-(fluorosulfonyl)-3-methyl-1H-benzo[d]imidazol-3-iumtrifluoromethanesulfonate (2c)

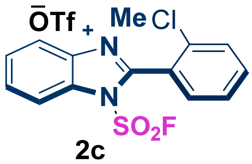

2c

65%; white solid: m.p. 145-146 °C; ^{1}H NMR (400 MHz, Acetonitrile-d_3) δ 8.23 (dt, J = 7.4, 0.9 Hz, 1H), 8.20 – 8.13 (m, 1H), 8.07 – 7.96 (m, 2H), 7.95 – 7.87 (m, 2H), 7.83 (dd, J = 8.3, 1.2 Hz, 1H), 7.75 (td, J = 7.6, 1.2 Hz, 1H), 4.00 (s, 3H). ^{13}C NMR (101 MHz, Acetonitrile-d_3) δ 150.7, 137.3, 135.2, 133.4, 132.6, 132.2, 131.7, 130.8, 130.5, 129.5, 122.0 (q, J = 321.0 Hz), 120.3, 116.4, 116.0, 35.6. ^{19}F NMR (376 MHz, Acetonitrile-d_3) δ 63.52, -79.24. HRMS(ESI): caled for $C_{14}H_{11}ClFN_2O_2S^+$ [M]$^+$ 325.0208; found 325.0207.

2-(4-fluorophenyl)-1-(fluorosulfonyl)-3-methyl-1H-benzo[d]imidazol-3-iumtrifluoromethanesulfonate (2d)

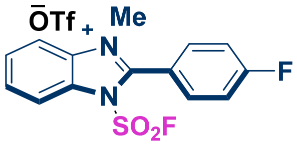

2d

60%; white solid: m.p. 148-149 °C; ^{1}H NMR (400 MHz, Acetonitrile-d_3) δ 8.24 – 8.16 (m, 1H), 8.14 – 8.04 (m, 1H), 8.01 – 7.95 (m, 2H), 7.94 – 7.89 (m, 2H), 7.59 – 7.48 (m, 2H), 3.95 (s, 3H). ^{13}C NMR (101 MHz, Acetonitrile-d_3) δ 168.4, 165.8, 153.4, 134.6 (d, J = 9.8 Hz), 132.7, 131.8, 130.6, 122.0 (q, J = 320.7 Hz), 118.1, 116.9 (d, J = 3.4 Hz), 116.0, 115.9, 35.49. ^{19}F NMR (376 MHz, $CDCl_3$) δ 64.65, -79.30, -104.18– -104.25 (m). HRMS(ESI): caled for $C_{14}H_{11}F_2N_2O_2S^+$ [M]$^+$ 309.0504; found 309.0505.

1-(fluorosulfonyl)-2-(4-methoxyphenyl)-3-methyl-1H-benzo[d]imidazol-3-iumtrifluoromethanesulfonate (2e)

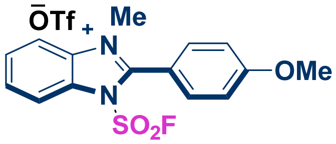

2e

43%; white solid: m.p. 162-163 °C; ^{1}H NMR (400 MHz, Acetonitrile-d_3) δ 8.23 – 8.13 (m, 1H), 8.12 – 8.00 (m, 1H), 7.99 – 7.89 (m, 2H), 7.85 – 7.74 (m, 2H), 7.33 – 7.25 (m, 2H), 3.96 (s, 3H), 3.95 (s, 3H). ^{13}C NMR (101 MHz, Acetonitrile-d_3) δ 165.4, 154.7, 133.7, 132.7, 131.4, 130.6, 130.3, 122.1 (q, J = 320.9 Hz), 116.1, 116.0, 115.7, 111.9, 56.8, 35.4. ^{19}F NMR (376 MHz, Acetonitrile-d_3) δ 64.72, -79.28. HRMS(ESI): caled for $C_{15}H_{14}FN_2O_3S^+$ [M]$^+$ 321.0704; found 321.0704.

IV. Cyclic Voltammetry Studies for 2a-2e

Unless otherwise noted, the cyclic voltammetry measurements were conducted on a MPI-A multi-functional electrochemical and chemiluminescent system (Shanghai CH Instrument Ltd. Co., China) at room temperature, with a polished Pt plate as the working electrode, platinum thread as the counter electrode and Ag-$AgNO_3$ (0.1 M) in

CH_3CN as the reference electrode, tetrabutylammonium tetrafluoroborate (0.1 M) was used as the supporting electrolyte, using Fc+/Fc as the internal standard, the scan rate was 0.2 V/s.

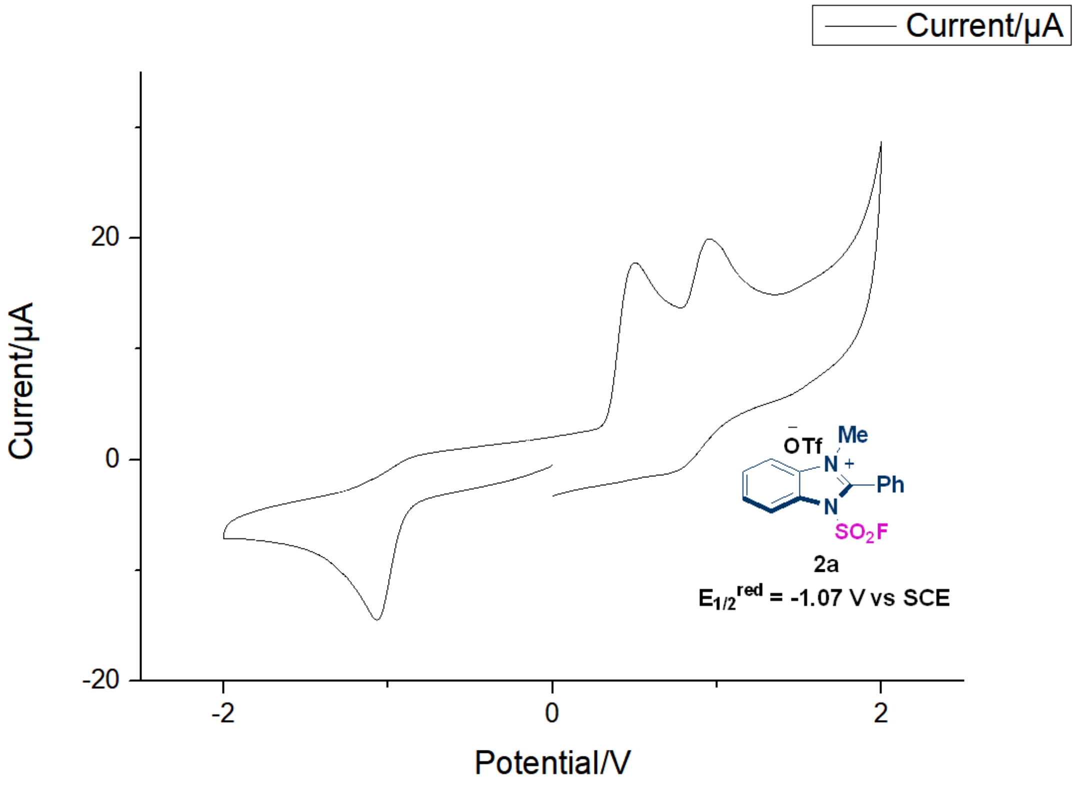

Supplementary Figure 4. Cyclic voltammograms of **2a**

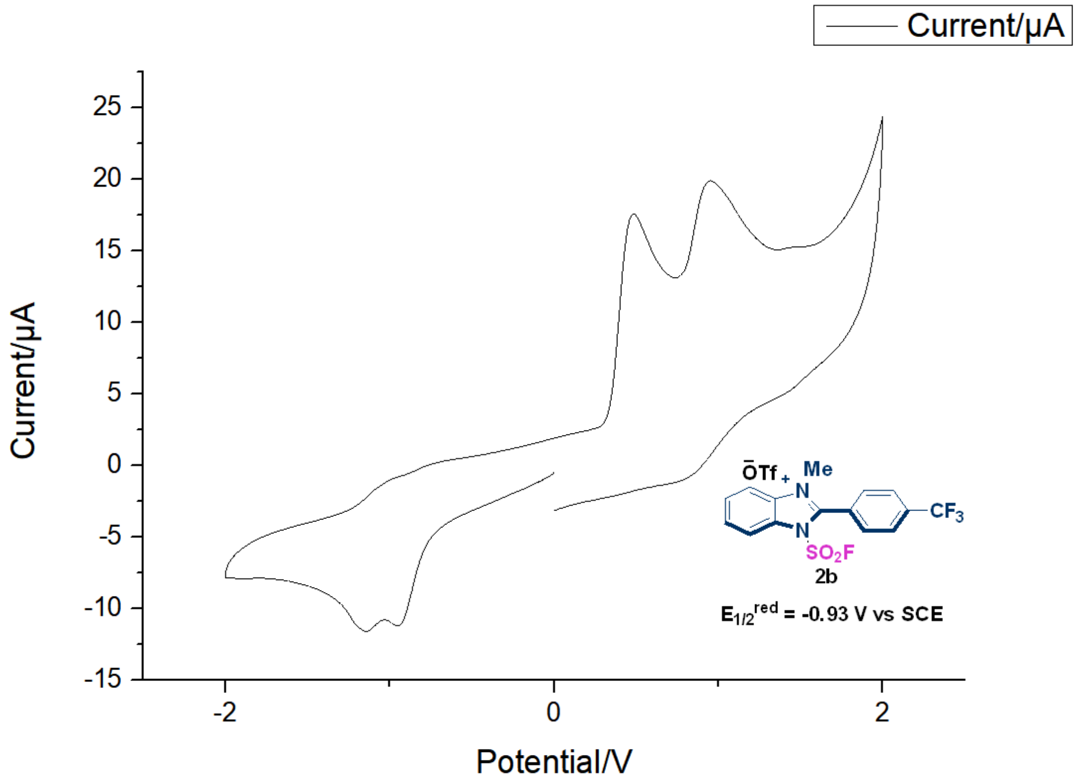

Supplementary Figure 5. Cyclic voltammograms of **2b**

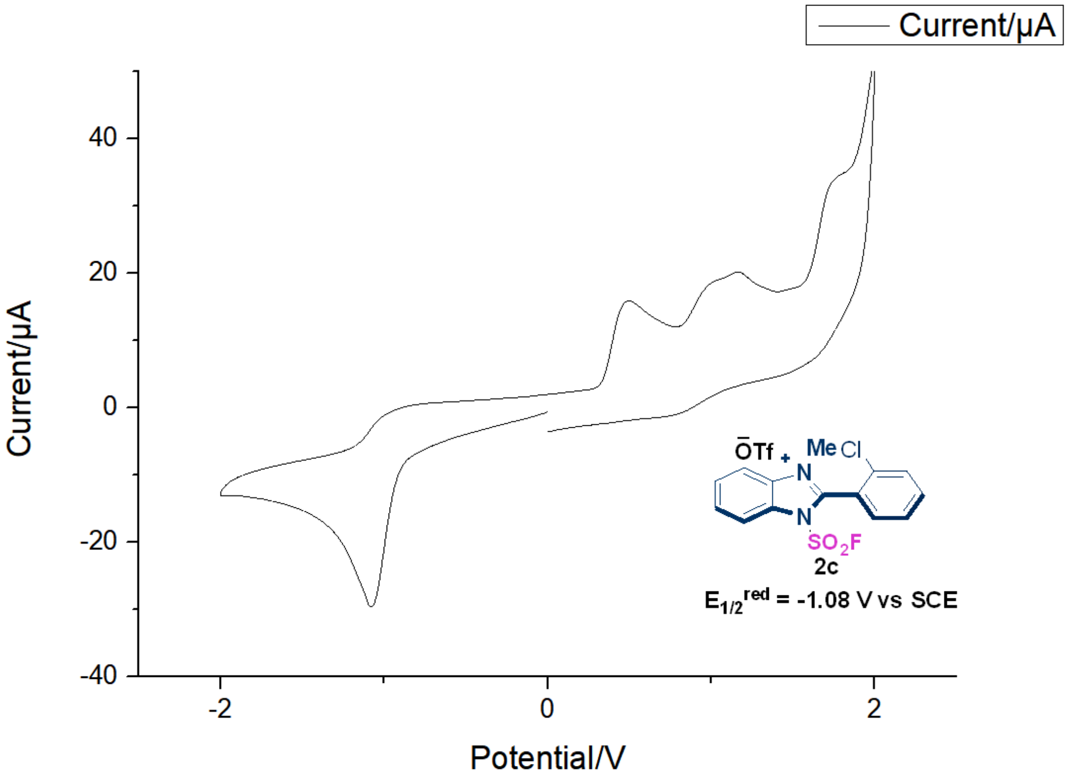

Supplementary Figure 6. Cyclic voltammograms of **2c**

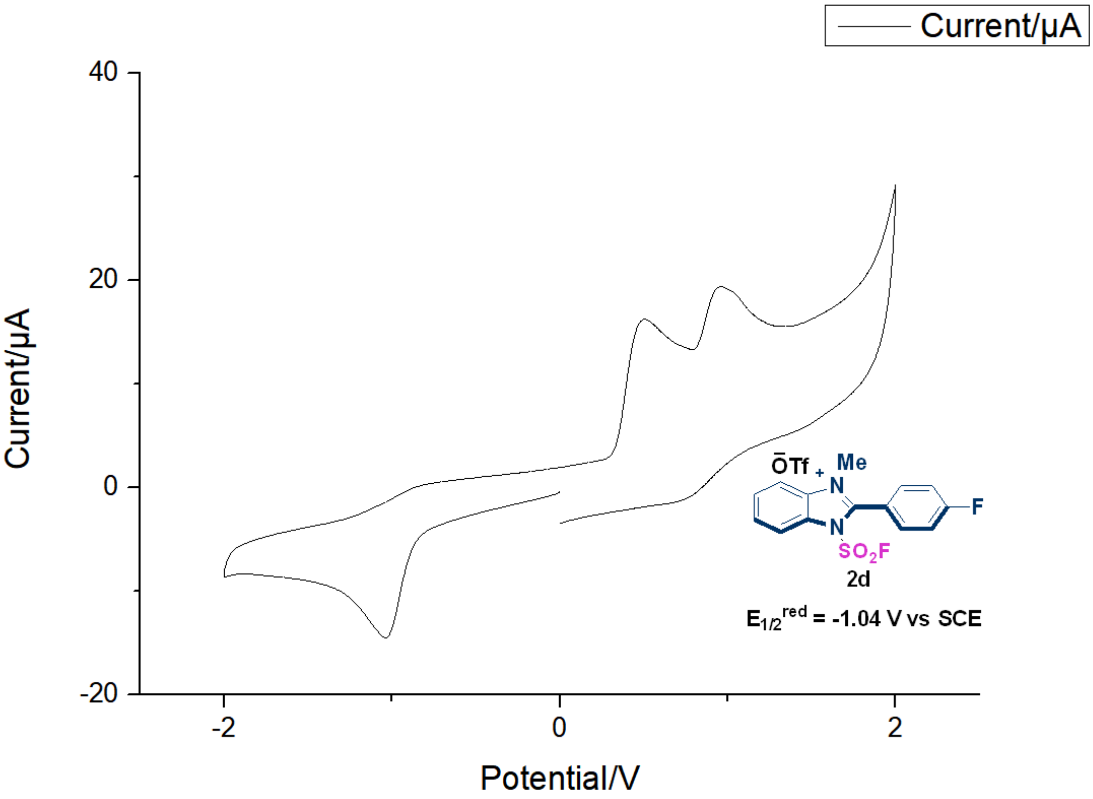

Supplementary Figure 7. Cyclic voltammograms of **2d**

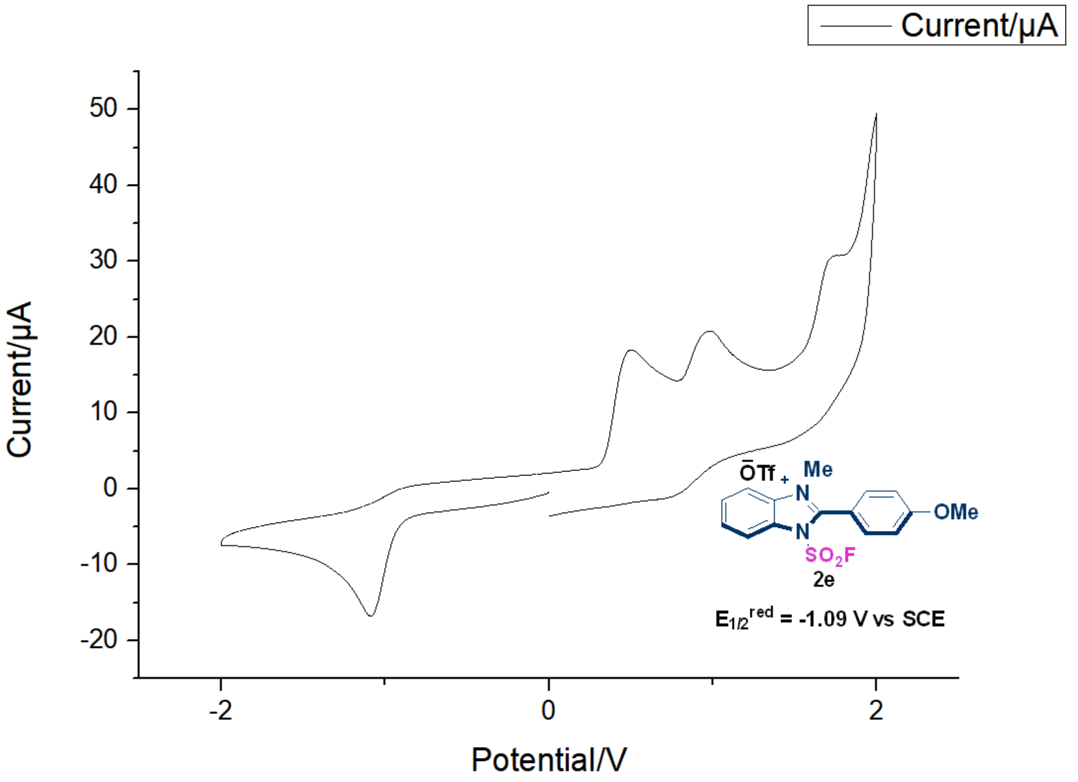

Supplementary Figure 8. Cyclic voltammograms of **2e**

V. Optimizations of the Reaction Conditions

Supplementary Table 1: Optimization of solvents[a]

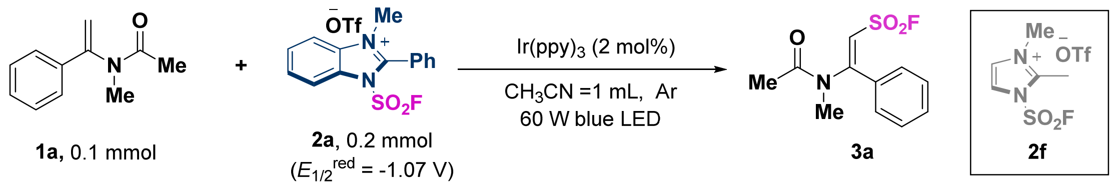

Entry	Change of conditions	Yield of **3a**[b]	*E/Z* ratio[c]
1	None	10%	> 20:1
2	EA(1mL)	41%	> 20:1
**3**	**DME(1mL)**	**45%**	> 20:1
4	NMP(1mL)	n.d.	-
5	2-Me-THF(1mL)	13.4%	> 20:1
6	hexane(1mL)	n.d.	-
7	DMSO(1mL)	n.d.	-
8	DMF(1mL)	n.d.	-
9	Acetone	8%	> 20:1
10	**2f** instead of **2a**	n.d.	-

[a] All reactions were carried out with **1a** (17.5 mg, 0.10 mmol), **2a** (0.20 mmol, 2equiv), *fac*-Ir(ppy)3 (2 mol%) in solution (1.0 mL) under Ar and 60 W blue LEDs. [b] Yields determined by GC using dodecane as an internal standard. [c] The *E/Z* ratio was determined by ^{1}H NMR.

Supplementary Table 2: Optimization of additives [a]

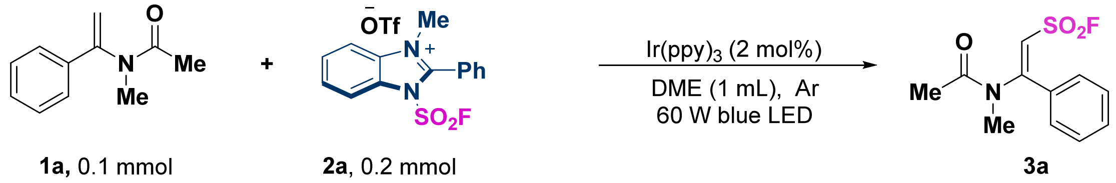

Entry	Change of additives	Yield of **3a**[b]	*E/Z* ratio[c]
1	None	45%	> 20:1
2	DMAP (2.5 equiv)	n.d.	-
3	Na_2HPO_4 (2.5 equiv)	46%	> 20:1
4	K_2HPO_4 (2.5 equiv)	27%	> 20:1
5	Na_3PO_4 (2.5 equiv)	32%	> 20:1
6	K_3PO_4 (2.5 equiv)	27%	> 20:1
**7**	**KH_2PO_4 (2.5 equiv)**	**59%**	> 20:1
8	CsF (2.5 equiv)	n.d.	-
9	LiCl (2.5 equiv)	n.d.	-
10	Pyridine (2.5 equiv)	12.5%	> 20:1

[a] All reactions were carried out with **1a** (17.5 mg, 0.10 mmol), **2a** (0.20 mmol, 2equiv), *fac*-Ir(ppy)$_3$ (2 mol%), additive (2.5 equiv) in DME (1.0 mL) under Ar and 60 W blue LEDs. [b] Yields determined by GC using dodecane as an internal standard. [c] The *E/Z* ratio was determined by ^{1}H NMR.

Supplementary Table 3: Optimization of photocatalysts and light sources[a]

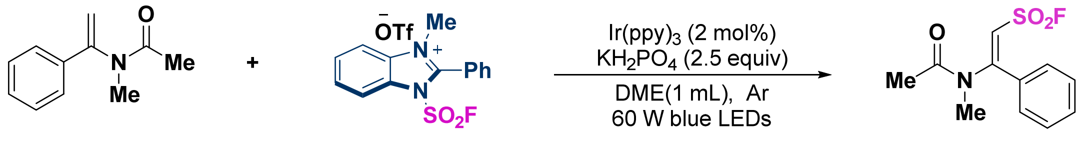

1a, 0.1 mmol **2a**, 0.2 mmol ($E_{1/2}^{red}$ = -1.07 V) **3a**

Entry	Change of conditions	Yield of **3a**[b]	*E/Z* ratio[c]
1	None	59%	> 20:1
**2**	**4CzIPN**	**62%**	> 20:1
3	Ir{[dF(CF_3)ppy]$_2$(dtbbpy)}PF_6	38%	> 20:1
4	4CzIPN (26 W visible light)	57%	> 20:1

5	4CzIPN (90 W blue LED)	55%	> 20:1
6	4CzIPN (30 W blue LED)	56%	> 20:1

[a] All reactions were carried out with **1a** (17.5 mg, 0.10 mmol), **2a** (0.20 mmol, 2equiv), photosensitizer (2 mol%), KH_2PO_4 (0.20 mmol, 2equiv) in DME (1.0 mL) under Ar and light irradiation. [b] Yields determined by GC using dodecane as an internal standard. [c] The *E/Z* ratio was determined by ^{1}H NMR.

Supplementary Table 4: Optimization of imidazolium reagents[a]

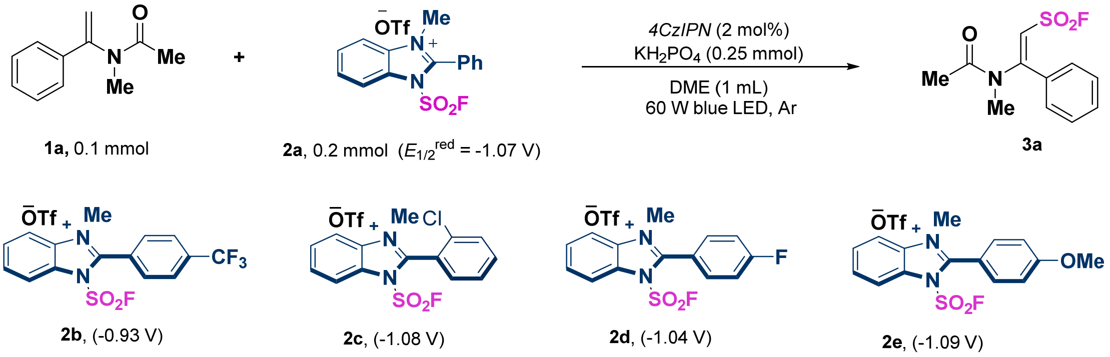

Entry	Change of reagents	Yield of **3a**[b]	*E/Z* ratio[d]
1	None	62%	> 20:1
**2**	**2b**	**71% (65%)[c]**	> 20:1
3	**2c**	58%	> 20:1
4	**2d**	64%	> 20:1
5	**2e**	16%	> 20:1

[a] All reactions were carried out with **1a** (16.0 mg, 0.10 mmol), **2** (0.20 mmol, 2equiv), 4CzIPN (2 mol%), in DME (1.0 mL) under Ar and 60 W blue LEDs. [b] Yields determined by GC using dodecane as an internal standard. [c] Isolated yields. [d] The *E/Z* ratio was determined by ^{1}H NMR.

Supplementary Table 5: Optimization of *Z*-alkenylsulfonyl fluoride reaction conditions[a]

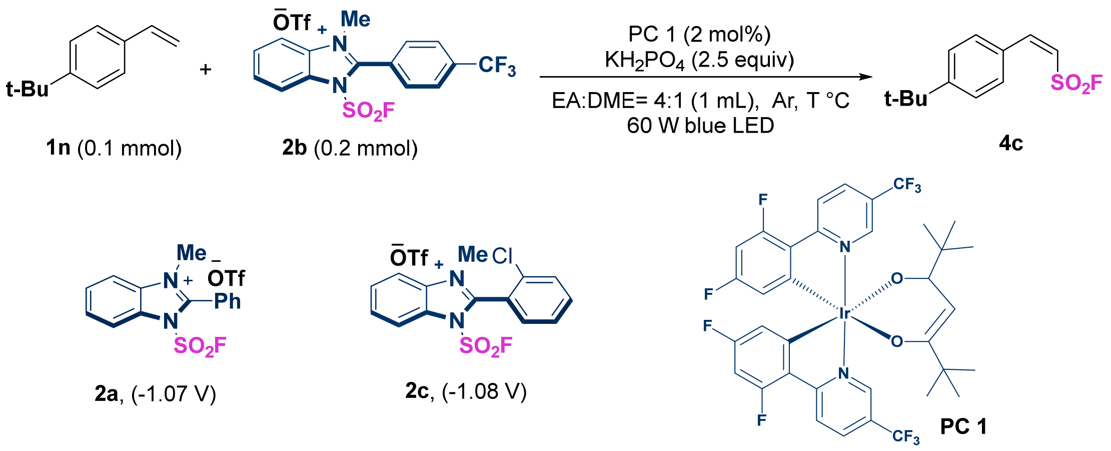

Entry	Change of conditions	Yield of **4c**[b]	*E/Z* ratio[d]
1	None	39	0.85:1
2	**2a** instead of **2b**	20	1.15:1
3	**2c** instead of **2b**	23	0.81:1
4	*fac*-Ir[*d*-F(*p*-*t*-Bu)ppy]$_3$	18.2	2.43:1
5	Ru(phen)$_3$(PF$_6$)$_2$	0	-
6	Fluorescein	0	-
7	w/o PC	0	-
8	w/o Light	0	-
9	30℃	40	0.86:1
10	40℃	37	0.84:1
11	50°C	44	0.78:1
12	50°C, **Then Ir{[dF(CF$_3$)ppy]$_2$(dtbbpy)}PF$_6$ in 1.5 mL MeCN was injected to the tube, 12h**	**50 (41)[c]**	**0.72:1**
**13**	50°C, Then Ir(dFppy)$_3$ in 1.5 mL MeCN was injected to the tube, 12h	33.3	1.10:1

14	50°C, Then *fac*-Ir[*d*-F(*p*-*t*-Bu)ppy]$_3$ in 1.5 mL MeCN was injected to the tube, 12h	29	1.15:1
15	50°C, Then *fac*-Ir[(3-*t*Bu-phenyl)-4-*t*Bu-ppy]$_3$ in 1.5 mL MeCN was injected to the tube, 12h	29	1.19:1
16	50°C, Then Ir[(bpy)$_2$dtbbpy]PF$_6$ in 1.5 mL MeCN was injected to the tube, 12h	36.7	1.05:1
17	50°C, Then Ru(phen)$_3$(PF$_6$)$_2$ in 1.5 mL MeCN was injected to the tube, 12h	47.8	0.89:1

[a] All reactions were carried out with **1n** (16.0 mg, 0.10 mmol), **2b** (0.20 mmol, 2 equiv), PC (2 mol%), in EA:DME = 4:1 (1.0 mL) under Ar and 60W blue LEDs. [b] Yields determined by GC using dodecane as an internal standard. [c] Isolated yields. [d] The *E/Z* ratio was determined by ^{1}H NMR.

Supplementary Table 6: Optimization of radical hydrofluorosulfonylation reaction [a]

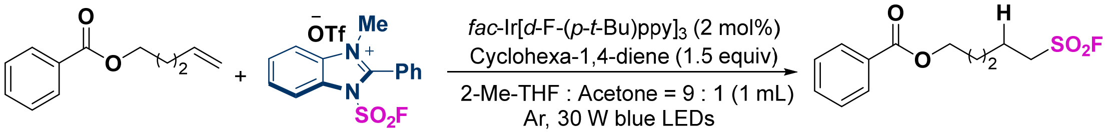

5a (0.1 mmol) **2a**, 0.2 mmol **6a**

Entry	Change of solvents	Yield of **6a**[b]
**1**	**None**	**69%**
2	MeCN	0%
3	EA	18%
4	DCM	trace
5	DME	37%
6	Acetone	22%
7	DMSO	0%
8	DMF	0%
9	MeOH	0%
10	THF:MeCN = 4:1	35%
11	2-Methyltetrahydrofuran:MeCN = 4:1	53%
12	2-Methyltetrahydrofuran:Acetone = 4:1	64%

13	2-Methyltetrahydrofuran:Acetone = 3:2	63%
14	2-Methyltetrahydrofuran:Acetone = 5:1	65%

[a] All reactions were carried out with **5a** (0.10 mmol), **2a** (0.20 mmol, 2.0 equiv), *fac*-Ir[*d*-F-(*p*-*t*-Bu)ppy]$_3$ (2 mol%) and cyclohexa-1,4-diene (1.5 equiv) in solution (1 mL) under Ar and 30 W blue LEDs. [b] Yields determined by GC using dodecane as an internal standard.

Supplementary Table 7: Optimization of photocatalysts and light sources [a]

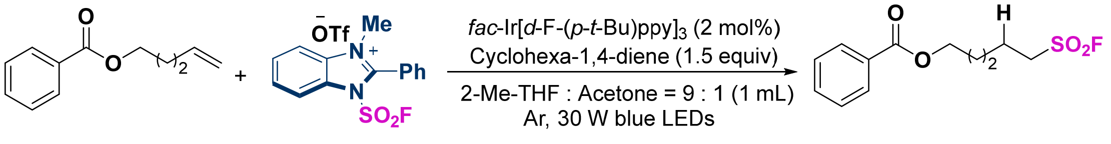

5a (0.1 mmol) **2a**, 0.2 mmol **6a**

Entry	PC	Light sources	Yield of **6a**[b]
**1**	*fac*-Ir[*d*-F-(*p*-*t*-Bu)ppy]$_3$	30 W Blue LEDs	69%
2	Ir{[dF(CF$_3$)ppy]$_2$(dtbbpy) }PF$_6$	30 W Blue LEDs	29%
3	4CzIPN	30 W Blue LEDs	44%
4	*fac*-Ir[*d*-F-(*p*-*t*-Bu)ppy]$_3$	10 W Blue LEDs	27%
**5**	***fac*-Ir[*d*-F-(*p*-*t*-Bu)ppy]$_3$**	**60 W Blue LEDs**	**72% (68)**[c]
6	4CzIPN	90 W Blue LEDs	67%

[a] All reactions were carried out with **5a** (0.10 mmol), **2a** (0.20 mmol, 2.0 equiv), PC (2 mol%) and cyclohexa-1,4-diene (1.5 equiv) in 2-methyltetrahydrofuran:acetone = 9:1 (1 mL) under Ar and light irradiation. [b] Yields determined by GC using dodecane as an internal standard. [c] Isolated yields.

Supplementary Table 8: Optimization of radical migration fluorosulfonylation reaction [a]

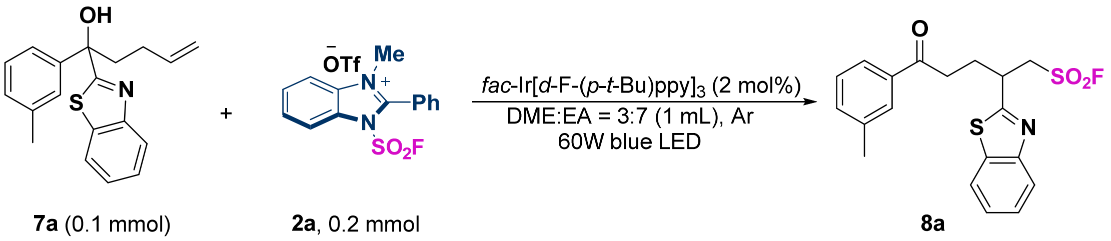

7a (0.1 mmol) **2a**, 0.2 mmol **8a**

Entry	Change of conditions	Yield of **8a**[b]
**1**	**None**	**66%**
2	EA	40%
3	DMSO	0%

4	DMF	0%
5	DMAc	0%
6	NMP	0%

[a] All reactions were carried out with **7a** (0.10 mmol), **2a** (0.20 mmol, 2.0 equiv), *fac*-Ir[*d*-F-(*p*-*t*-Bu)ppy]$_3$ (2 mol%) in DME:EA = 4:1 (1.0 mL) under Ar and 60 W blue LEDs. [b] Isolated yields.

VI. General Procedure for the Synthesis of the Products 3, 4, 6 and 8

General Procedure for the synthesis of product 3

Condition A: Underargon, to a solution of 4CzIPN (2 mol%), KH_2PO_4 (2.5 equiv) and IMSF reagent **2b** (0.2 mmol, 2 equiv.) in dried DME (1 mL) was added corresponding alkenes **1** (0.1 mmol) at room temperature. After that, the tube was exposed to a 60 W blue LEDs about 10 h until the reaction was completed as monitored by TLC analysis. The reaction mixturewas evaporated in *vacuo*.The crude products were directly purified by flash chromatography on silica gel to give the desired products.

General Procedure for the synthesis of product 4

Condition B: Underargon, to a solution of PC 1 (2 mol%), KH_2PO_4 (2.5 equiv) and IMSF reagent **2b** (0.2 mmol, 2 equiv.) in dried EA:DME = 4:1 (1 mL) was added corresponding alkenes **1** (0.1 mmol) at room temperature. After that, the tube was exposed to a 60 W blue LEDs about 12 h, then 1.5 ml of acetonitrile containing Ir{[dF(CF_3)ppy]$_2$(dtbbpy)}PF_6 (2 mol%) was injected into the reaction tube about 12 h until the reaction was completed as monitored by TLC analysis. The reaction mixturewas evaporated in *vacuo*.The crude products were directly purified by flash chromatography on silica gel to give the desired products.

General Procedure for the synthesis of product 6

Condition C: Underargon, to a solution of *fac*-Ir[*d*-F-(*p*-*t*-Bu)ppy]$_3$ (2 mol%), 1,4-cyclohexadiene (1.5 equiv) and IMSF reagent **2a** or **2b** (0.2 mmol, 2 equiv.) in dried 2-Methyltetrahydrofuran : Acetone = 9:1 (1 mL) was added corresponding alkenes **5** (0.1 mmol) at room temperature. After that, the tube was exposed to a 60 W blue LEDs about 12 h, then until the reaction was completed as monitored by TLC analysis. The reaction mixture was evaporated in *vacuo*.The crude products were directly purified by flash chromatography on silica gel to give the desired products.

General Procedure for the synthesis of product 8

Condition D: Underargon, to a solution of *fac*-Ir[*d*-F-(*p*-*t*-Bu)ppy]$_3$ (2 mol%), and IMSF reagent **2a** (0.2 mmol, 2 equiv) in dried EA:DME = 7:3 (1 mL) was added corresponding alkenes **7** (0.1 mmol) at room temperature. After that, the tube was exposed to a 60 W blue LEDs about 12 h, then until the reaction was completed as monitored by TLC analysis. The reaction mixture was evaporated in *vacuo*.The crude products were directly purified by flash chromatography on silica gel to give the desired products.

VII. Mechanistic studies and synthetic application

Synthetic application (a):

To a solution of 2-Pyrazolin-5-one **9** (0.2 mmol), $NaHCO_3$ (1.0 equiv) and DBU (5 mol%) in dried DCM (1 mL) was added corresponding alkenes **3m** (0.1 mmol) at room temperature about 24 h until the reaction was completed as monitored by TLC analysis. The reaction mixture was evaporated in *vacuo*.The crude products were directly purified by flash chromatography on silica gel to give the desired product **10** in 50% yield.

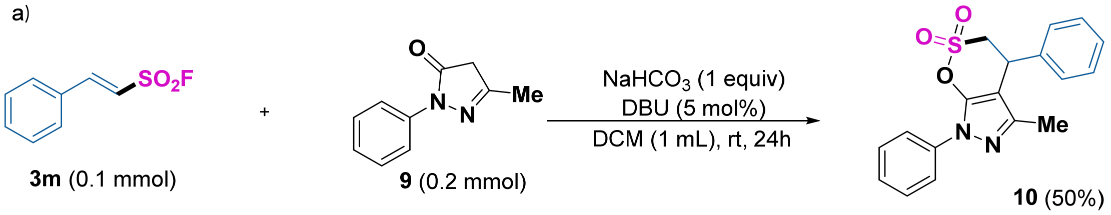

Synthetic application (b):

To a solution of 5,5-dimethylcyclohexane-1,3-dione **11** (0.2 mmol), NEt_3 (1.5 equiv) in dried DMSO (1 mL) was added corresponding alkenes **3m** (0.1 mmol) at room temperature about 1 h until the reaction was completed as monitored by TLC analysis. The reaction mixture was evaporated in *vacuo*.The crude products were directly purified by flash chromatography on silica gel to give the desired product **12** in 40% yield.

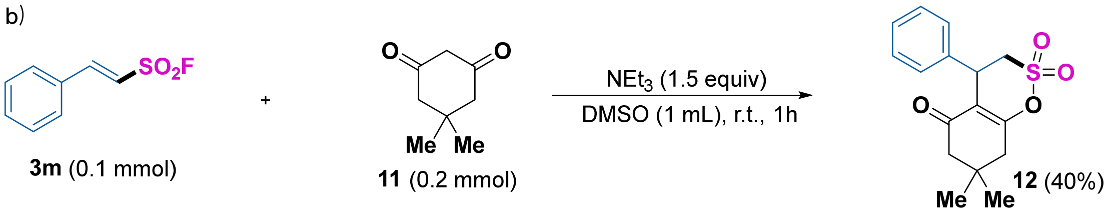

Synthetic application (c):

To a solution of Estrone **13** (0.2 mmol), KOH (2.0 equiv) in dried CH_3CN (1 mL) was added corresponding alkenes **3m** (0.1 mmol) at 50°C about 12 h until the reaction was completed as monitored by TLC analysis. The reaction mixture was evaporated in *vacuo*.The crude products were directly purified by flash chromatography on silica gel to give the desired product **14** in 60% yield.

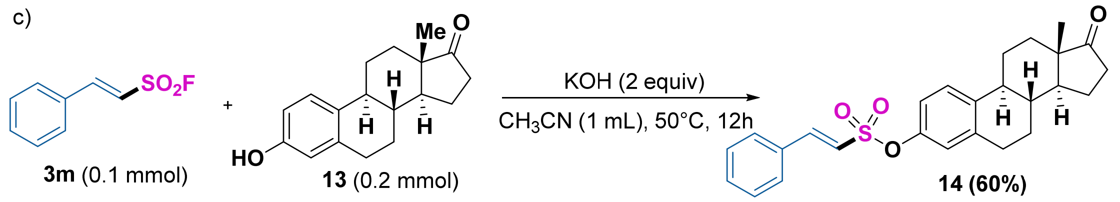

Synthetic application(d):

To a solution of Vitamin E **15** (0.05 mmol), KOH (2.0 equiv) in dried CH_3CN (1 mL) was added Lumacator intermediate derivative **6f** (0.025 mmol) at 50°C about 12 h until the reaction was completed as monitored by TLC analysis. The reaction mixture was evaporated in *vacuo*.The crude products were directly purified by flash chromatography on silica gel to give the desired product **16** in 70% yield.

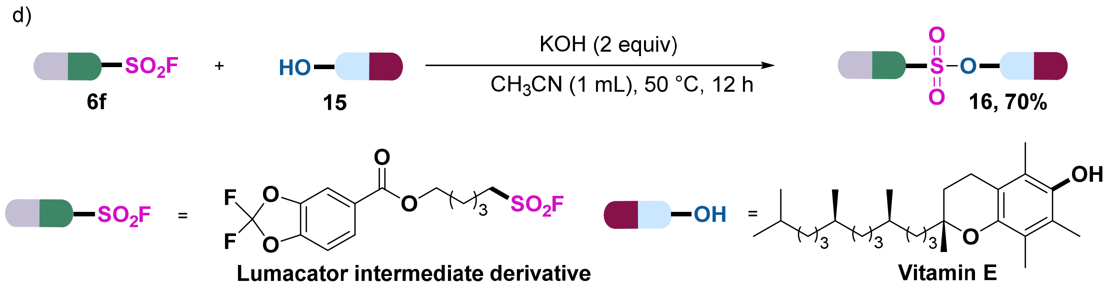

Mechanistic study (a):

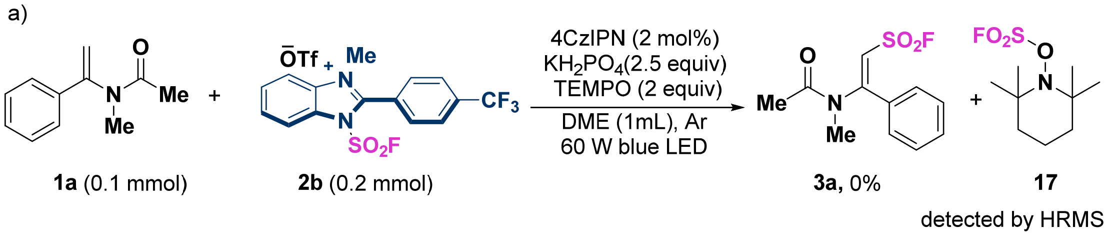

Underargon, to a solution of 4CzIPN (2 mol%), KH_2PO_4 (2.5 equiv), TEMPO (2 equiv) and IMSF reagent **2b** (0.2 mmol, 2 equiv.) in dried DME (1 mL) was added corresponding alkenes **1a** (0.1 mmol) at room temperature. After that, the tube was exposed to a 60 W blue LEDs about 10 h until the reaction was completed as monitored by TLC analysis. Subsequently, the reaction mixture was analyzed by GC. GC showed that no major product **3a** was formed after addition of 0.2 mmol of TEMPO. In addition, we were fortunate to detect product **17** in HRMS (ESI). HRMS (ESI): caled for $C_9H_{18}FNO_3SNa^+$ $[M+Na]^+$ 262.0883; found 262.0887.

Mechanistic study (b):

b)

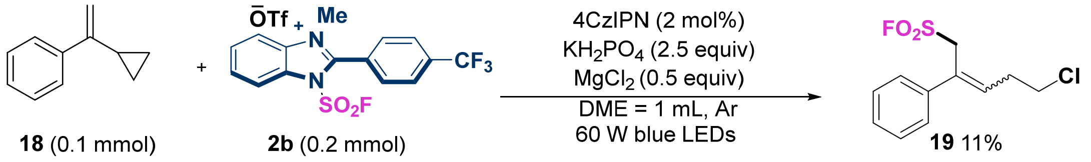

Underargon, to a solution of 4CzIPN (2 mol%), KH_2PO_4 (2.5 equiv), $MgCl_2$ (0.5 equiv) and IMSF reagent **2b** (0.2 mmol, 2 equiv.) in dried DME (1 mL) was added corresponding alkenes **18** (0.1 mmol) at room temperature. After that, the tube was exposed to a 60 W blue LEDs about 10 h until the reaction was completed as monitored by TLC analysis. We obtained the product **19** in an isolated yield of 11%.

VIII. Proposed Mechanism

(a) Proposed Mechanism for Alkenylsulfonyl fluoride reaction

As shown below, the mechanism for photocatalytic alkenylsulfonyl fluoride could be proposed. Starting from the oxidation of the excited state of 4CzIPN* by IMSF reagent **2b**, 4CzIPN$^+$ species was generated with imidazole **B** and fluorosulfonyl radical **A**, which can undergo addition reactions with alkenes **1** to produce intermediate **C**. Then the intermediate **C** was oxidized by 4CzIPN$^+$ to get intermediate **D** and regenerate 4CzIPN. The intermediate **D** underwent hydrogen eliminated to get product E-alkenylsulfonyl fluoride **3** in the presence of a weak base. Under photocatalytic conditions, the product **3** underwent isomerisation to produce *Z*-alkenylsulfonyl fluoride product **4**.

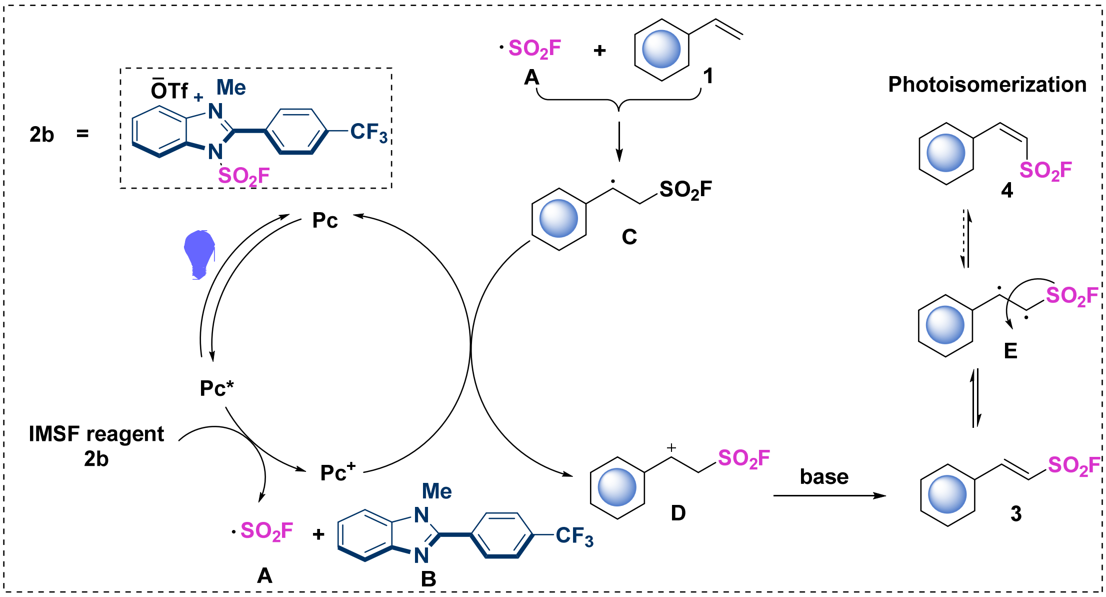

Supplementary Figure 9. Proposed Mechanism for Alkenylsulfonyl fluoride reaction

(b) Proposed Mechanism for hydrofluorosulfonylation reaction

As shown below, the mechanism for photocatalytic hydrofluorosulfonylation could be proposed. Starting from the oxidation of the excited state of iridium catalyst Ir(III)* by IMSF reagent **2a**, Ir(IV) species was generated with imidazole **F** and fluorosulfonyl radical **A**. The additon of SO_2F radical and olefin **5** afforded intermediate **G** followed by hydrogen atom transfer with cyclohexa-1,4-diene **H** to furnish the product **6**. The Ir(IV) species was reduced by intermediate **I** to regenerate Ir(III) catalyst and to give compound **J**.

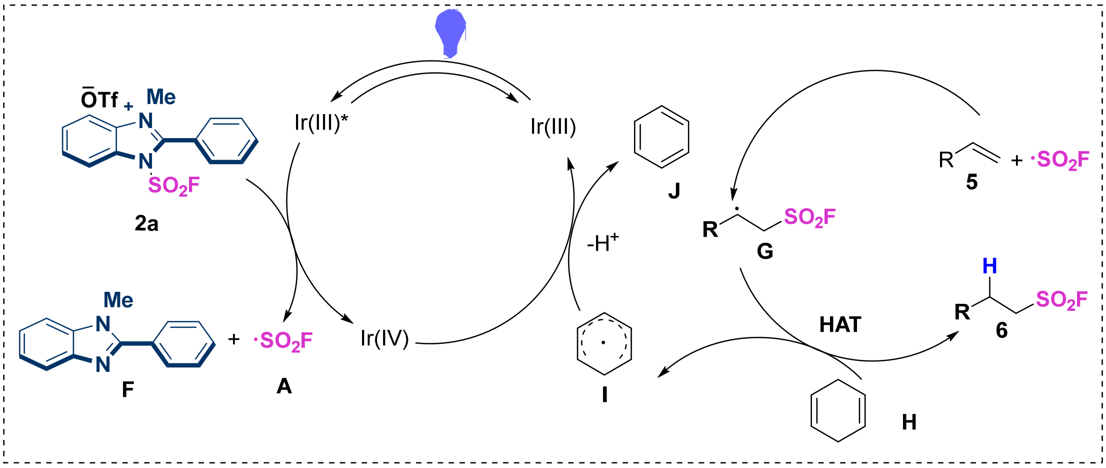

Supplementary Figure 10. Proposed Mechanism for hydrofluorosulfonylation reaction

(c) Proposed Mechanism for migration fluorosulfonylation reaction

As shown below, the mechanism for photocatalytic migration fluorosulfonylation could be proposed. Initially, the oxidation of the excited state of iridium catalyst Ir(III)* by IMSF reagent **2a**, Ir(IV) species was generated with sulfonyl fluoride radical **A**. The additon of SO_2F radical and olefins **7** afforded the carbon radical intermediate **K,** which rapidly attacks the heteroarene to generate cyclic nitrogen radical intermediate **L**. The fast ring opening followed by radical β-cleavage furnished a stabilized radical intermediate **M**, which is oxidized by Ir(IV) to carbocation intermediate **N**. Finally, deprotonation of intermediate **N** afforded the heteroaryl migrated products **8a-8h**.

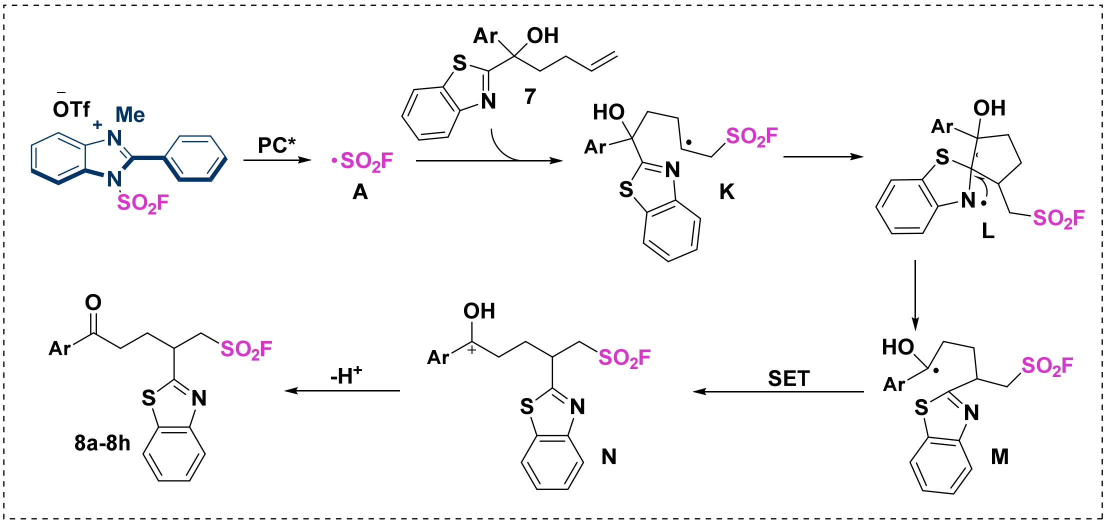

Supplementary Figure 11. Proposed Mechanism for migration fluorosulfonylation reaction

IX. Characteristic Data

(E)-2-(N-methylacetamido)-2-phenylethene-1-sulfonyl fluoride (3a)

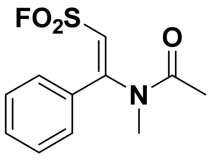

65% (16.7 mg); white solid: m.p. 95-96 °C; ^{1}H NMR (400 MHz, Chloroform-*d*) δ 7.62 – 7.53 (m, 1H), 7.54 – 7.43 (m, 4H), 6.45 (s, 1H), 3.08 (s, 3H), 2.13 (s, 3H). 13C NMR (101 MHz, Chloroform-*d*) δ 171.08, 159.33 (d, J = 5.0 Hz), 132.24, 132.01, 129.36, 129.05, 113.20 (d, J = 30.4 Hz), 36.83, 23.81. ^{19}F NMR (376 MHz, Chloroform-*d*) δ 69.87. HRMS (ESI): caled for $C_{11}H_{13}FNO_3S^+$ $[M + H]^+$ 258.0595; found 258.0595.

(E)-2-(N-isopropylacetamido)-2-phenylethene-1-sulfonyl fluoride (3b)

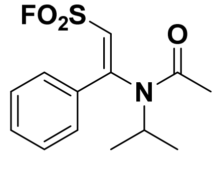

82% (23.5 mg); white solid: m.p. 100-101 °C; ^{1}H NMR (400 MHz, Chloroform-*d*) δ 7.62 – 7.44 (m, 5H), 6.34 (s, 1H), 4.37 (hept, J = 6.9 Hz, 1H), 2.16 (s, 3H), 1.16 (s, 3H), 1.14 (s, 3H). ^{13}C NMR (101 MHz, Chloroform-*d*) δ 169.7, 158.1 (d, J = 5.7 Hz), 132.9, 132.6, 129.9, 128.8, 117.6 (d, J = 29.7 Hz), 50.8, 24.2, 20.6. ^{19}F NMR (376 MHz, Chloroform-*d*) δ 67.54. HRMS (ESI): caled for $C_{13}H_{17}FNO_3S^+$ $[M + H]^+$ 286.0908; found 286.0907.

(E)-2-(N-benzylacetamido)-2-phenylethene-1-sulfonyl fluoride (3c)

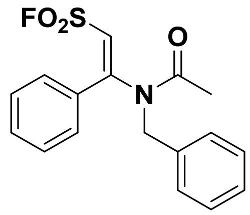

40% (13.2 mg); white solid: m.p. 103-104 °C; ^{1}H NMR (400 MHz, Chloroform-*d*) δ 7.62 – 7.53 (m, 1H), 7.48 (ddd, J = 8.0, 6.6, 1.2 Hz, 2H), 7.43 – 7.38 (m, 2H), 7.35 – 7.28 (m, 3H), 7.12 (dd, J = 7.8, 1.8 Hz, 2H), 6.26 (s, 1H), 4.68 (s, 2H), 2.20 (s, 4H). ^{13}C NMR (101 MHz, Chloroform-*d*) δ 170.7, 158.3 (d, J = 5.2 Hz), 135.9, 132.4, 131.7, 129.6, 129.0, 128.9, 128.1, 128.0, 115.5 (d, J = 30.0 Hz), 51.4, 23.6. ^{19}F NMR ^{19}F NMR (376 MHz, Chloroform-*d*) δ 69.01. HRMS (ESI): caled for $C_{17}H_{17}FNO_3S^+$ $[M + H]^+$ 334.0908; found 334.0908.

(E)-2-(N-(2-methylbenzyl)acetamido)-2-phenylethene-1-sulfonyl fluoride (3d)

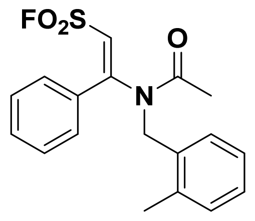

47% (16.5 mg); white solid: m.p. 114-115 °C; ^{1}H NMR (400 MHz, Chloroform-*d*) δ 7.60 – 7.52 (m, 1H), 7.46 (dd, *J* = 8.5, 7.0 Hz, 2H), 7.39 – 7.32 (m, 2H), 7.24 – 7.10 (m, 3H), 6.92 (dd, *J* = 6.8, 2.2 Hz, 1H), 6.37 (s, 1H), 4.76 (s, 2H), 2.19 (s, 3H), 2.09 (s, 3H). ^{13}C NMR (101 MHz, Chloroform-*d*) δ 170.8, 158.8 (d, *J* = 5.1 Hz), 135.6, 133.5, 132.3, 131.8, 130.8, 129.5, 128.9, 127.9, 127.1, 126.4, 114.7 (d, *J* = 30.1 Hz), 49.7, 23.9, 19.0. ^{19}F NMR (376 MHz, Chloroform-*d*) δ 69.47. HRMS (ESI): caled for $C_{18}H_{19}FNO_3S^+$ $[M + H]^+$ 348.1064; found 348.1064.

2,2-diphenylethene-1-sulfonyl fluoride (3e)

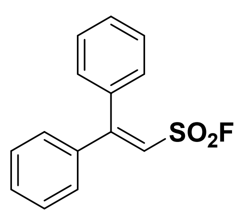

82% (21.5 mg); white solid: m.p. 77-78 °C; ^{1}H NMR (400 MHz, Chloroform-*d*) δ 7.52 – 7.42 (m, 4H), 7.39 (t, *J* = 7.7 Hz, 2H), 7.34 – 7.28 (m, 4H), 6.83 (s, 1H). ^{13}C NMR (101 MHz, Chloroform-*d*) δ 161.3 (d, *J* = 3.7 Hz), 138.1 (d, *J* = 2.1 Hz), 135.2, 131.5, 130.2, 129.3, 128.9, 128.9, 128.4, 117.7 (d, *J* = 28.1 Hz). ^{19}F NMR (376 MHz, Chloroform-*d*) δ 68.15. HRMS (ESI): caled for $C_{14}H_{12}FO_2S^+$ $[M + H]^+$ 263.0537; found 263.0537.

2,2-di-p-tolylethene-1-sulfonyl fluoride (3f)

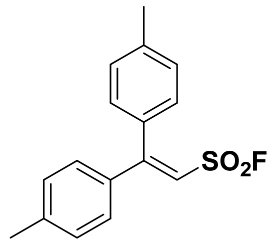

72% (21.0 mg); white solid: m.p. 82-83 °C; ^{1}H NMR (400 MHz, Chloroform-*d*) δ 7.27 (s, 1H), 7.25 (s, 1H), 7.21 (d, *J* = 10.3 Hz, 6H), 2.43 (s, 3H), 2.40 (s, 3H). ^{13}C NMR (101 MHz, Chloroform-*d*) δ 161.6 (d, *J* = 3.7 Hz), 142.1, 140.4, 135.5 (d, *J* = 2.2 Hz), 132.4, 129.5, 129.4, 129.0, 129.0, 116.0 (d, *J* = 27.6 Hz), 21.5, 21.4. ^{19}F NMR (376 MHz, Chloroform-*d*) δ 68.54. HRMS (ESI): caled for $C_{16}H_{16}FO_2S^+$ $[M + H]^+$ 291.0850; found 291.0850.

2,2-bis(4-fluorophenyl)ethene-1-sulfonyl fluoride (3g)

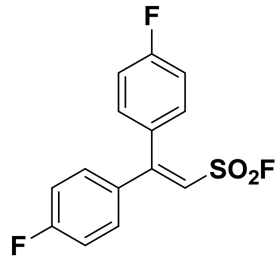

74% (22.0 mg); white solid: m.p. 86-87 °C; ^{1}H NMR (400 MHz, Chloroform-*d*) δ 7.31 (ddd, *J* = 9.0, 5.3, 3.9 Hz, 4H), 7.21 – 7.05 (m, 4H), 6.78 (s, 1H). ^{13}C NMR (101 MHz, Chloroform-*d*) δ 166.1, 165.2, 163.5, 162.7, 159.0 (d, *J* = 4.1 Hz), 134.1, 131.5 (d, *J* = 8.2 Hz), 131.0 (d, *J* = 8.9 Hz), 117.7 (d, *J* = 28.4 Hz), 116.0 (dd, *J* = 43.1, 21.9 Hz). ^{19}F NMR (376 MHz, Chloroform-*d*) δ 68.16, -107.36 (td, *J* = 8.6, 3.9 Hz), -109.34 (tt, *J* = 8.6, 5.3 Hz). HRMS (ESI): caled for $C_{14}H_{10}F_3O_2S^+$ [M + H]$^+$ 299.0348; found 299.0349.

2-(thiophen-2-yl)ethyl 3-(fluorosulfonyl)-2-phenylacrylate (3h)

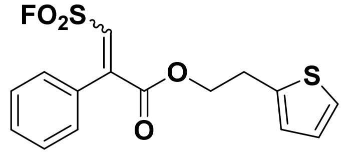

32% (E/Z = 1:1) (11.0 mg); yellow oil; ^{1}H NMR (400 MHz, Chloroform-*d*), (*E/Z* mixture of isomer), δ 7.57 – 7.39 (m, 4H), 7.31 (dt, *J* = 6.8, 1.5 Hz, 1H), 7.17 (ddd, *J* = 6.7, 5.1, 1.3 Hz, 1H), 7.01 – 6.84 (m, 1H), 6.81 – 6.68 (m, 1H), 4.63 – 4.46 (m, 2H), 3.31 – 3.19 (m, 2H). ^{13}C NMR (101 MHz, Chloroform-d), (*E/Z* mixture of isomer), δ 164.0, 151.8, 148.1, 139.0, 138.8, 132.4, 130.8, 130.4, 130.1, 130.0 (d, J = 31.9 Hz), 129.5, 128.8, 128.3, 127.1, 127.0, 117.6 (d, J = 30.8 Hz), 67.2, 67.0, 29.0, 28.8. ^{19}F NMR (376 MHz, Chloroform-d), (*E/Z* mixture of isomer), δ 66.05, 65.87. HRMS (ESI): caled for $C_{15}H_{13}FO_4S_2Na^+$ [M + Na]$^+$ 363.0131; found 363.0130.

pentyl (E)-3-(fluorosulfonyl)-2-phenylacrylate (3i)

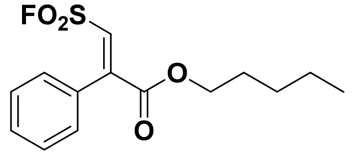

53% (16.0 mg); colorless oil; ^{1}H NMR (500 MHz, Chloroform-*d*) δ 7.50 – 7.42 (m, 4H), 7.38 – 7.32 (m, 2H), 4.26 (t, *J* = 6.7 Hz, 2H), 1.68 (p, *J* = 6.8 Hz, 2H), 1.39 – 1.14 (m, 4H), 0.90 (t, *J* = 6.9 Hz, 3H). ^{13}C NMR (126 MHz, Chloroform-*d*) δ 163.8 (d, *J* = 2.8 Hz), 148.5 (d, *J* = 4.7 Hz), 130.3, 130.3, 129.5 (d, *J* = 31.5 Hz), 128.7, 128.2, 67.4, 28.0, 27.8, 22.1, 13.9. ^{19}F NMR (471 MHz, Chloroform-*d*) δ 65.92. HRMS (ESI): caled for $C_{14}H_{17}FNO_4SNa^+$ [M + Na]$^+$ 323.0724; found 323.0721.

(E)-1,2-diphenylethene-1-sulfonyl fluoride (3j)

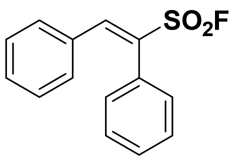

23% (6.0 mg); white solid: m.p. 107-108 °C; ^{1}H NMR (400 MHz, Chloroform-*d*) δ 7.94 (s, 1H), 7.59 – 7.46 (m, 3H), 7.43 (dd, *J* = 7.6, 2.0 Hz, 2H), 7.38 – 7.30 (m, 1H), 7.23 (t, *J* = 7.8 Hz, 2H), 7.11 (d, *J* = 7.2 Hz, 2H). ^{13}C NMR (126 MHz, Chloroform-*d*) δ 143.3 (d, *J* = 2.2 Hz), 133.9 (d, *J* = 22.5 Hz), 131.5, 131.3, 131.1, 130.4, 130.3, 129.6, 129.4, 128.8. ^{19}F NMR (376 MHz, Chloroform-*d*) δ 53.03. HRMS (ESI): caled for $C_{14}H_{11}FO_2SNa^+$ $[M + Na]^+$ 285.0356; found 285.0355.

(E)-1-phenylprop-1-ene-2-sulfonyl fluoride (3k)

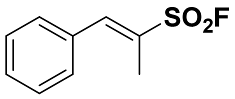

30% (6.0 mg); white solid: m.p. 50-51 °C; ^{1}H NMR (500 MHz, Chloroform-*d*) δ 7.78 (s, 1H), 7.51 – 7.41 (m, 5H), 2.40 (s, 3H). ^{13}C NMR (126 MHz, Chloroform-*d*) δ 142.2 (d, *J* = 2.7 Hz), 132.3, 130.7 (d, *J* = 21.4 Hz) 130.5, 129.9, 129.0, 13.7. ^{19}F NMR (471 MHz, Chloroform-*d*) δ 50.53. HRMS (ESI): caled for $C_9H_{10}FO_2S^+$ $[M + H]^+$ 201.0380; found 201.0381.

(E)-2-phenylprop-1-ene-1-sulfonyl fluoride (3l)

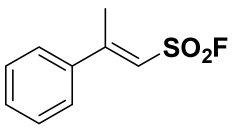

69% (13.7 mg); white solid: m.p. 55-56 °C; ^{1}H NMR (500 MHz, Chloroform-*d*) δ 7.55 – 7.43 (m, 5H), 6.62 (s, 1H), 2.64 (s, 3H). ^{13}C NMR (126 MHz, Chloroform-*d*) δ 160.1 (d, *J* = 2.4 Hz), 138.7, 131.0, 129.1, 126.5, 118.3 (d, *J* = 25.5 Hz), 18.59. ^{19}F NMR ^{19}F NMR (471 MHz, Chloroform-*d*) δ 66.09. HRMS (ESI): caled for $C_9H_{10}FO_2S^+$ $[M + H]^+$ 201.0380; found 201.0382.

(E)-2-phenylethene-1-sulfonyl fluoride (3m)

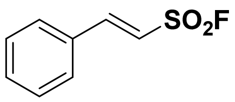

81% (15.1 mg); white solid: m.p. 86-87 °C; ^{1}H NMR (400 MHz, Chloroform-d) δ 7.82 (d, J = 15.6 Hz, 1H), 7.59 – 7.43 (m, 5H), 6.88 (d, J = 15.5 Hz, 1H). ^{13}C NMR (101 MHz, Chloroform-*d*) δ 148.9 (d, *J* = 2.7 Hz),

132.7, 131.0, 129.4, 129.1, 117.9 (d, *J* = 28.1 Hz). ^{19}F NMR (376 MHz, Chloroform-*d*) δ 62.30. HRMS (ESI): caled for $C_8H_8FO_2S^+$ $[M + H]^+$ 187.0224; found 187.0231.

(E)-2-(4-(tert-butyl)phenyl)ethene-1-sulfonyl fluoride (3n)

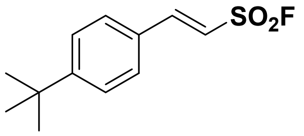

82% (19.1 mg); white solid: m.p. 46-47 °C; ^{1}H NMR (400 MHz, Chloroform-*d*) δ 7.79 (d, *J* = 16.7 Hz, 1H), 7.49 (s, 4H), 6.82 (d, *J* = 15.5 Hz, 1H), 1.34 (s, 9H). ^{13}C NMR (101 MHz, Chloroform-*d*) δ 156.71, 148.83 (d, *J* = 2.7 Hz), 129.0, 128.2, 126.4, 116.7 (d, *J* = 27.7 Hz), 35.2, 31.0. ^{19}F NMR (376 MHz, Chloroform-*d*) δ 62.59. HRMS (ESI): caled for $C_{12}H_{16}FO_2S^+$ $[M + H]^+$ 243.0850; found 243.0850.

(E)-2-(4-fluorophenyl)ethene-1-sulfonyl fluoride (3o)

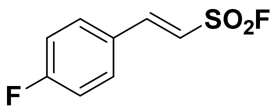

64% (13 mg); white solid: m.p. 79-80 °C; ^{1}H NMR (400 MHz, Chloroform-*d*) δ 7.78 (d, *J* = 15.5 Hz, 1H), 7.67 – 7.51 (m, 2H), 7.17 (t, *J* = 8.5 Hz, 2H), 6.81 (d, *J* = 15.5 Hz, 1H).^{13}C NMR (101 MHz, Chloroform-*d*) δ 165.2 (d, *J* = 255.6 Hz), 147.5 (d, *J* = 2.7 Hz), 131.3 (d, *J* = 9.1 Hz), 127.3 (d, *J* = 3.6 Hz), 117.7 (dd, *J* = 28.3, 2.6 Hz), 116.8 (d, *J* = 22.3 Hz). ^{19}F NMR ^{19}F NMR (376 MHz, Chloroform-*d*) δ 62.39, -104.98. HRMS (ESI): caled for $C_8H_7F_2O_2S^+$ $[M + H]^+$ 205.0130; found 205.0130.

(E)-2-(4-chlorophenyl)ethene-1-sulfonyl fluoride (3p)

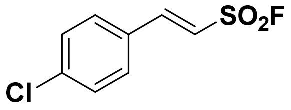

82% (18.1 mg); white solid: m.p. 114-115 °C; ^{1}H NMR (400 MHz, Chloroform-*d*) δ 7.77 (d, *J* = 15.5 Hz, 1H), 7.48 (q, *J* = 8.7 Hz, 4H), 6.86 (d, *J* = 15.5 Hz, 1H). ^{13}C NMR (101 MHz, Chloroform-*d*) δ 147.4 (d, *J* = 2.8 Hz), 138.9, 130.2, 129.8, 129.4, 118.5 (d, *J* = 28.4 Hz).. ^{19}F NMR (376 MHz, Chloroform-*d*) δ 62.33. HRMS (ESI): caled for $C_8H_7ClFO_2S^+$ $[M + H]^+$ 220.9834; found 220.9832.

(E)-2-(m-tolyl)ethene-1-sulfonyl fluoride (3q)

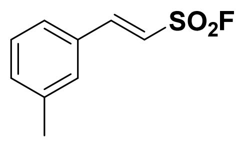

67% (12.4 mg); white solid: m.p. 32-33 °C; ^{1}H NMR (400 MHz, Chloroform-*d*) δ 7.78 (d, *J* = 15.6 Hz, 1H), 7.41 – 7.30 (m, 4H), 6.85 (d, *J* = 15.5 Hz, 1H), 2.40 (s, 3H).^{13}C NMR (101 MHz, Chloroform-*d*) δ 149.1 (d, *J* = 2.8 Hz), 139.3, 133.5, 130.9, 129.6, 129.3, 126.3, 117.6 (d, *J* = 28.0 Hz), 21.3. ^{19}F NMR (376 MHz, Chloroform-*d*) δ 62.35. HRMS (ESI): caled for $C_9H_{10}FO_2S^+$ $[M + H]^+$ 201.0380; found 201.0379.

(E)-2-(2,6-dimethylphenyl)ethene-1-sulfonyl fluoride (3r)

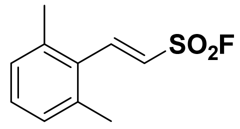

70% (15.0 mg); slight yellow oil; ^{1}H NMR (400 MHz, Chloroform-*d*) δ 7.79 (d, *J* = 15.5 Hz, 1H), 7.49 (s, 3H), 6.82 (d, *J* = 15.5 Hz, 1H), 1.34 (s, 6H). ^{13}C NMR (101 MHz, Chloroform-*d*) δ 156.7, 148.8 (d, *J* = 2.6 Hz), 129.0, 128.2, 126.4, 116.7 (d, *J* = 27.7 Hz), 31.0. ^{19}F NMR (376 MHz, Chloroform-*d*) δ 62.58. HRMS (ESI): caled for $C_{10}H_{12}FO_2S^+$ $[M + H]^+$ 215.0537; found 215.0539.

3,4-dihydronaphthalene-2-sulfonyl fluoride (3s)

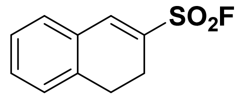

35% (35.5 mg); colorless oil; ^{1}H NMR (400 MHz, Chloroform-*d*) δ 7.62 (s, 1H), 7.38 (dt, *J* = 7.4, 4.3 Hz, 1H), 7.29 (d, *J* = 6.2 Hz, 2H), 7.23 (d, *J* = 8.3 Hz, 1H), 3.05 ((t, *J* = 8.0 Hz, 2H), 2.79 (t, *J* = 8.0 Hz, 2H). ^{13}C NMR (101 MHz, Chloroform-*d*) δ 140.20 (d, *J* = 3.0 Hz), 136.03, 131.79, 130.30 (d, *J* = 25.0 Hz), 129.77, 129.67, 128.15, 127.47, 27.20, 21.98. ^{19}F NMR (376 MHz, Chloroform-*d*) δ 54.17. HRMS (ESI): caled for $C_{10}H_{10}FO_2S^+$ $[M + H]^+$ 213.0380; found 213.0381.

(E)-2-(thiophen-2-yl)ethene-1-sulfonyl fluoride (3t)

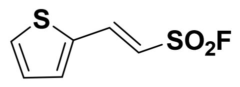

65% (12.5 mg); white solid: m.p. 64-65 °C; ^{1}H NMR (400 MHz, Chloroform-*d*) δ 7.79 (d, *J* = 16.6 Hz, 1H), 7.73 (d, *J* = 2.9 Hz, 1H), 7.44 (dd, *J* = 5.2, 2.9 Hz, 1H), 7.30 (d, *J* = 5.1 Hz, 1H), 6.69 (d, *J* = 15.4 Hz, 1H). ^{13}C NMR (101 MHz, Chloroform-*d*) δ 142.0 (d, *J* = 2.7 Hz), 134.0, 132.5, 128.3, 125.0,

117.1 (d, J = 27.9 Hz). ^{19}F NMR (376 MHz, Chloroform-*d*) δ 62.86. HRMS (ESI): caled for $C_6H_6FO_2S_2^+$ [M + H]$^+$ 192.9788; found 192.9786.

(E)-2-(pyridin-2-yl)ethene-1-sulfonyl fluoride (3u)

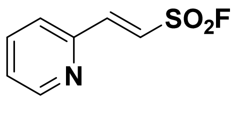

53% (11.6 mg); yellow solid: m.p. 48-49 °C; ^{1}H NMR (500 MHz, Chloroform-*d*) δ 8.73 (d, J = 7.2 Hz, 1H), 7.83 (td, J = 7.7, 1.8 Hz, 1H), 7.80 (d, J = 16.1 Hz, 1H), 7.55 (d, J = 17.1 Hz, 1H), 7.49 (d, J = 7.7 Hz, 1H), 7.43 (dd, J = 7.7, 4.7 Hz, 1H). ^{13}C NMR (126 MHz, Chloroform-*d*) δ 150.7, 149.4, 146.7 (d, J = 3.3 Hz), 137.3, 126.4, 126.2, 122.7 (d, J = 28.9 Hz). ^{19}F NMR (471 MHz, Chloroform-*d*) δ 61.59. HRMS (ESI): caled for $C_7H_7FNO_2S^+$ [M + H]$^+$ 188.0176; found 188.0176.

phenethyl (E)-4-(2-(fluorosulfonyl)vinyl)benzoate (3v)

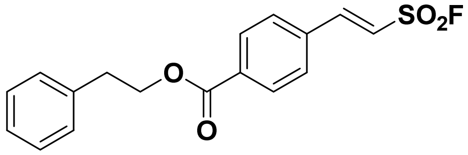

33% (11.0 mg); white solid: m.p. 92-93 °C; ^{1}H NMR (400 MHz, Chloroform-*d*) δ 8.13 – 8.04 (m, 2H), 7.84 (d, J = 15.6 Hz, 1H), 7.65 – 7.58 (m, 2H), 7.38 – 7.21 (m, 4H), 6.96 (dd, J = 15.6, 2.5 Hz, 1H), 4.57 (t, J = 7.0 Hz, 2H), 3.10 (t, J = 6.9 Hz, 2H). ^{13}C NMR (101 MHz, Chloroform-*d*) δ 165.3, 147.3 (d, J = 2.8 Hz), 137.6, 134.8, 133.6, 130.4, 128.9, 128.9, 128.6, 126.7, 120.3 (d, J = 28.8 Hz), 66.0, 35.1. ^{19}F NMR (376 MHz, Chloroform-*d*) δ 62.10. HRMS (ESI): caled for $C_{17}H_{15}FO_4SNa^+$ [M + Na]$^+$ 357.0567; found 357.0567.

(2R,8R,9R,10S,13S,14R,17S)-10,13-dimethyl-17-((S)-6-methylheptan-2-yl)-2,3,4,7,8,9,10,11,12,13,14,15,16,17-tetradecahydro-1H-cyclopenta[a]phenanthren-2-yl 4-((E)-2-(fluorosulfonyl)vinyl)benzoate (3w)

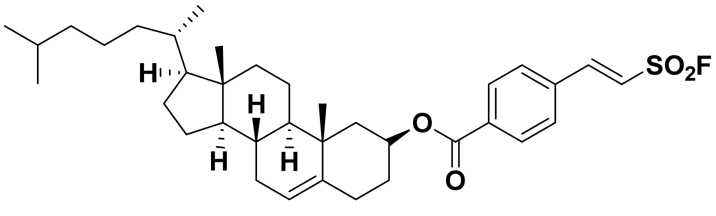

42% (25.0 mg); white solid: m.p. 176-177 °C; ^{1}H NMR (400 MHz, Chloroform-*d*) δ 8.13 (d, J = 8.4 Hz, 2H), 7.84

(d, J = 15.6 Hz, 1H), 7.62 (d, J = 8.5 Hz, 2H), 6.96 (dd, J = 15.6, 2.4 Hz, 1H), 5.43 (d, J = 4.9 Hz, 1H), 4.94 – 4.82 (m, 1H), 2.47 (d, J = 7.7 Hz, 2H), 2.07 – 1.91 (m, 4H), 1.87 – 1.73 (m, 2H), 1.56 – 1.29 (m, 9H), 1.25 – 0.98 (m, 15H), 0.92 (d, J = 6.5 Hz, 3H), 0.87 (dd, J = 6.7, 1.8 Hz, 6H), 0.69 (s, 3H). ^{13}C NMR (101 MHz, Chloroform-*d*) δ 164.8, 147.4 (d, J = 2.8 Hz), 139.4, 134.6, 134.2, 130.5, 128.8, 123.1, 120.2 (d, J = 28.8 Hz), 75.3, 56.7, 56.1, 50.0, 42.3, 39.7, 39.5, 38.1, 37.0, 36.6, 36.2, 35.8, 31.9, 31.9, 28.2, 28.0, 27.8, 24.3, 23.8, 22.8, 22.6, 21.0, 19.4, 18.7, 11.9. ^{19}F NMR (376 MHz, Chloroform-*d*) δ 62.12. HRMS (ESI): caled for $C_{36}H_{52}FO_4S^+$ $[M + H]^+$ 599.3565; found 599.3617.

(E)-2-((8R,9S,13S,14S)-13-methyl-17-oxo-7,8,9,11,12,13,14,15,16,17-decahydro-6H-cyclopenta[a]phenanthren-3-yl)ethene-1-sulfonyl fluoride (3x)

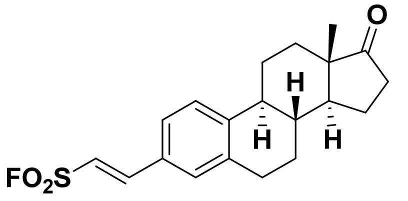

33% (12.0 mg); white solid: m.p. 81-82 °C; ^{1}H NMR (500 MHz, Chloroform-*d*) δ 7.76 (d, J = 15.5 Hz, 1H), 7.39 (d, J = 8.2 Hz, 1H), 7.34 (d, J = 10.1 Hz, 1H), 7.28 (s, 1H), 6.81 (dd, J = 15.5, 2.4 Hz, 1H), 2.98 – 2.87 (m, 2H), 2.53 (dd, J = 18.9, 9.4 Hz, 1H), 2.47 – 2.41 (m, 1H), 2.38 – 2.32 (m, 1H), 2.25 – 2.09 (m, 1H), 2.12 – 2.03 (m, 2H), 2.00 (dt, J = 12.6, 2.8 Hz, 1H), 1.68 – 1.61 (m, 2H), 1.57 – 1.45 (m, 4H), 0.93 (s, 3H). ^{13}C NMR (126 MHz, Chloroform-*d*) δ 220.4, 148.9 (d, J = 2.6 Hz), 145.3, 137.9, 129.8, 128.5, 126.5, 126.4, 116.9, 50.5, 47.9, 44.7, 37.8, 35.8, 31.5, 29.2, 26.1, 25.5, 21.6, 13.8. ^{19}F NMR (471 MHz, Chloroform-*d*) δ 62.55. HRMS (ESI): caled for $C_{20}H_{24}FO_3S^+$ $[M + H]^+$ 363.1425; found 363.1425.

(Z)-2-phenylethene-1-sulfonyl fluoride (4a)

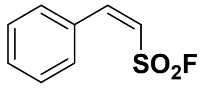

42% (7.8 mg); white solid: m.p. 56-57 °C; ^{1}H NMR (400 MHz, Chloroform-*d*) δ 7.65 – 7.56 (m, 2H), 7.51 – 7.41 (m, 3H), 7.39 (dd, J = 11.9, 5.8 Hz, 1H), 6.51 (dd, J = 11.9, 2.6 Hz, 1H). ^{13}C NMR (101 MHz, Chloroform-*d*) δ 146.68 (d, J = 3.8 Hz), 131.25, 131.06, 130.05 (d, J = 1.7 Hz), 128.69, 120.27 (d,

J = 28.6 Hz). ^{19}F NMR (376 MHz, Chloroform-*d*) δ 64.00; HRMS (ESI): caled for $C_8H_7FO_2SNa^+$ $[M + Na]^+$ 209.0043; found 209.0039.

(Z)-2-(m-tolyl)ethene-1-sulfonyl fluoride (4b)

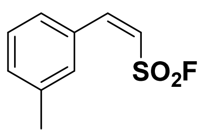

45% (9.0 mg); slight yellow solid: m.p. 47-48 °C; ^{1}H NMR (400 MHz, Chloroform-*d*) δ 7.42 (d, J = 7.6 Hz, 1H), 7.39 – 7.31 (m, 3H), 7.28 (d, J = 7.7 Hz, 1H), 6.48 (dd, J = 11.9, 2.6 Hz, 1H), 2.39 (s, 3H). ^{13}C NMR (101 MHz, Chloroform-*d*) δ 146.9 (d, J = 3.9 Hz), 138.5, 132.1, 131.0, 130.7 (d, J = 1.6 Hz), 128.6, 127.2 (d, J = 1.8 Hz), 120.0 (d, J = 28.7 Hz), 21.3. ^{19}F NMR (376 MHz, Chloroform-*d*) δ 63.98. HRMS (ESI): caled for $C_9H_{10}FO_2S^+$ $[M + H]^+$ 201.0380; found 201.0381.

(Z)-2-(4-(tert-butyl)phenyl)ethene-1-sulfonyl fluoride (4c)

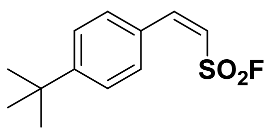

41% (10.0 mg); colorless oil; ^{1}H NMR (400 MHz, Chloroform-*d*) δ 7.59 (d, J = 8.4 Hz, 2H), 7.47 (d, J = 8.5 Hz, 2H), 7.31 (dd, J = 12.0, 5.6 Hz, 1H), 6.43 (dd, J = 12.0, 3.0 Hz, 1H), 1.34 (s, 9H). ^{13}C NMR ^{13}C NMR (101 MHz, Chloroform-*d*) δ 155.2, 146.6 (d, J = 3.6 Hz), 130.5, 128.1, 125.8, 118.6 (d, J = 28.4 Hz), 35.0, 31.0. ^{19}F NMR (376 MHz, Chloroform-*d*) δ 63.77. HRMS (ESI): caled for $C_{12}H_{15}FO_2SNa^+$ $[M + Na]^+$ 265.0669; found 265.0669.

(Z)-2-(2,6-dimethylphenyl)ethene-1-sulfonyl fluoride (4d)

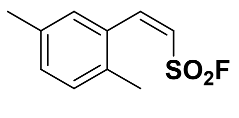

50% (10.7 mg); slight yellow oil; ^{1}H NMR (500 MHz, Chloroform-*d*) δ 7.56 (dd, J = 11.4, 6.4 Hz, 1H), 7.25 (s, 1H), 7.14 (q, J = 7.8 Hz, 2H), 6.57 (dd, J = 11.4, 1.1 Hz, 1H), 2.35 (s, 3H), 2.28 (s, 3H). ^{13}C NMR (126 MHz, Chloroform-*d*) δ 146.9 (d, J = 3.9 Hz), 135.5, 133.2, 131.3, 130.8, 130.0, 129.4 (d, J = 2.3 Hz), 122.0 (d, J = 27.8 Hz), 20.8, 19.4. ^{19}F NMR (471 MHz, Chloroform-*d*) δ 64.96. HRMS (ESI): caled for $C_{10}H_{12}FO_2S^+$ $[M + H]^+$ 215.0537; found 215.0536.

(Z)-2-mesitylethene-1-sulfonyl fluoride (4e)

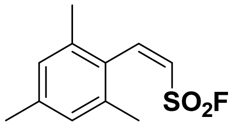

57% (12.9 mg); white solid: m.p. 115-116 °C; ^{1}H NMR (400 MHz, Chloroform-*d*) δ 7.53 (dd, *J* = 11.2, 6.3 Hz, 1H), 6.91 (s, 2H), 6.71 (d, *J* = 11.1 Hz, 1H), 2.30 (s, 3H), 2.22 (s, 6H). ^{13}C NMR (101 MHz, Chloroform-*d*) δ 148.2, 148.1, 138.6, 134.4, 128.2, 124.7 (d, *J* = 27.2 Hz), 21.1, 20.0. ^{19}F NMR (376 MHz, Chloroform-*d*) δ 62.49. HRMS (ESI): caled for $C_{11}H_{14}FO_2S^+$ $[M + H]^+$ 229.0693; found 229.0680.

(Z)-2-(4-chlorophenyl)prop-1-ene-1-sulfonyl fluoride (4f)

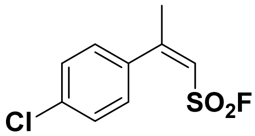

33% (7.7 mg); colorless oil; ^{1}H NMR (400 MHz, Chloroform-d) δ 7.44 – 7.35 (m, 2H), 7.27 – 7.13 (m, 2H), 6.49 (s, 1H), 2.31 (s, 3H). ^{13}C NMR (101 MHz, Chloroform-d) δ 159.6 (d, J = 3.6 Hz), 135.9, 135.0, 128.9, 128.1, 119.5 (d, J = 27.5 Hz), 27.3 (d, J = 2.3 Hz). ^{19}F NMR (376 MHz, Chloroform-d) δ 66.36. HRMS (ESI): caled for $C_9H_9ClFO_2S^+$ $[M + H]^+$ 234.9991; found 234.9989.

(Z)-2-(4-bromophenyl)ethene-1-sulfonyl fluoride (4g)

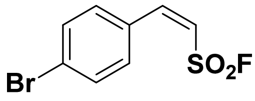

25% (6.5 mg); yellow solid: m.p. 53-54 °C; ^{1}H NMR (500 MHz, Chloroform-*d*) δ 7.59 (d, *J* = 8.6 Hz, 2H), 7.46 (d, *J* = 8.4 Hz, 2H), 7.31 (dd, *J* = 11.9, 5.7 Hz, 1H), 6.54 (dd, *J* = 11.9, 2.4 Hz, 1H). ^{13}C NMR (126 MHz, Chloroform-*d*) δ 145.3 (d, *J* = 3.9 Hz), 132.0, 131.5, 129.9, 126.1, 121.0 (d, *J* = 28.9 Hz). ^{19}F NMR (471 MHz, Chloroform-*d*) δ 64.14. HRMS (ESI): caled for $C_9H_7FO_2SNa+$ $[M + Na]^+$ 286.9148; found 286.9146.

(3R,5R)-3-isopropyl-5-methylcyclohexyl (Z)-3-(fluorosulfonyl)-2-phenylacrylate (4h)

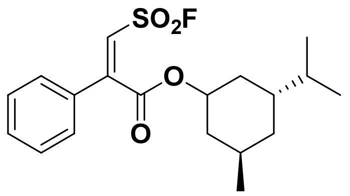

41% (15.0 mg); white solid: m.p. 124-125 °C; ^{1}H NMR (500 MHz, Chloroform-*d*) δ 7.60 – 7.45 (m, 5H), 6.71 (s, 1H), 4.97 (td, *J* = 10.9, 4.4 Hz, 1H), 2.36 – 2.29 (m, 1H), 1.87 (td, *J* = 7.0, 2.7 Hz, 1H), 1.77 – 1.69 (m, 2H), 1.62 (s, 1H), 1.56 (tdt, *J* = 12.0, 6.6, 3.4 Hz, 1H), 1.46 (ddt, *J* = 12.5, 10.8, 3.1 Hz, 1H), 1.10 (tdd, *J* = 12.1, 10.4, 7.9 Hz, 2H), 0.97 (d, *J* = 6.6 Hz, 3H), 0.85 (d, *J* = 7.0 Hz, 3H), 0.80 (d, *J* = 6.9 Hz, 3H). ^{13}C NMR (126 MHz, Chloroform-*d*) δ 163.8, 152.5 (d, *J* = 3.8 Hz), 132.3, 131.3, 129.4, 127.4, 117.0 (d, *J* = 30.7 Hz), 78.2, 46.9, 39.8, 34.1, 31.5, 25.6, 23.0, 22.0, 20.8, 15.7. ^{19}F NMR (471 MHz, Chloroform-*d*) δ 66.10. HRMS (ESI): caled for $C_{19}H_{25}FO_4SNa^+$ $[M + Na]^+$ 391.1350; found 391.1348.

Methyl (Z)-4-(2-(fluorosulfonyl)-1-(3,5,5,8,8-pentamethyl-5,6,7,8-tetrahydronaphthalen-2-yl)vinyl)benzoate (4i)

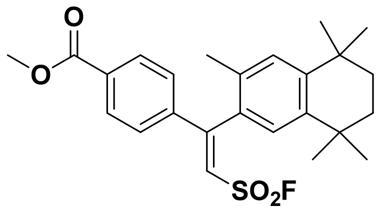

69% (*Z*:*E*=1.6:1) (31.0 mg); yellow oil; ^{1}H NMR (500 MHz, Chloroform-*d*) δ 8.09 – 8.01 (m, 1H), 7.42 – 7.36 (m, 1H), 7.17 (s, 0H), 7.12 (s, 1H), 6.99 (s, 0H), 3.93 (s, 1H), 1.90 (s, 1H), 1.71 (s, 2H), 1.29 (d, *J* = 9.2 Hz, 6H). ^{13}C NMR (126 MHz, Chloroform-*d*) δ 166.2, 160.6 (d, *J* = 3.6 Hz), 146.8, 142.4, 141.1 (d, *J* = 2.1 Hz), 132.4, 132.2, 130.8, 130.2, 128.5, 127.8, 127.6, 120.2 (d, *J* = 27.7 Hz), 52.5, 35.1, 35.0, 34.2, 34.0, 31.8, 31.7, 19.4. ^{19}F NMR (471 MHz, Chloroform-*d*) δ 67.04. HRMS (ESI): caled for $C_{25}H_{30}FO_4S^+$ $[M + H]^+$ 445.1844; found 445.1841.

5-(fluorosulfonyl)pentyl benzoate (6a)

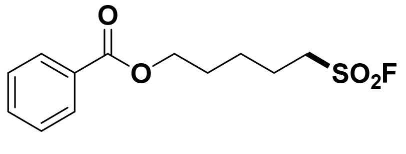

69% (18.8 mg); colorless oil; ^{1}H NMR (400 MHz, Chloroform-*d*) δ 8.06 – 8.00 (m, 1H), 7.61 – 7.52 (m, 2H), 7.45 (t, *J* = 7.7 Hz, 1H), 4.35 (t, *J* = 6.3 Hz, 2H), 3.45 – 3.35 (m, 2H), 2.04 (p, *J* = 7.7 Hz, 2H), 1.92 – 1.79 (m, 2H), 1.66 (p, *J* = 7.8, 7.3 Hz, 2H). 13C NMR (101 MHz, Chloroform-*d*) δ 166.5, 133.0, 130.1, 129.5, 128.4, 64.1,

50.7 (d, J = 16.5 Hz), 28.1, 24.6, 23.5. ^{19}F NMR (376 MHz, Chloroform-d) δ 59.62 (t, J = 4.7 Hz). HRMS (ESI): caled for $C_{12}H_{16}FO_4S^+$ [M + H]$^+$ 275.0748; found 275.0747.

5-(fluorosulfonyl)pentyl benzo[d][1,3]dioxole-5-carboxylate (6b)

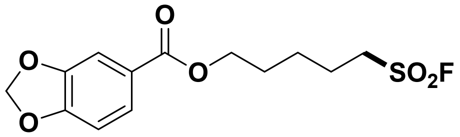

46% (14.5 mg); yellow solid: m.p. 48-49 °C; ^{1}H NMR (400 MHz, Chloroform-d) δ 7.63 (dd, J = 8.2, 1.7 Hz, 1H), 7.45 (d, J = 1.7 Hz, 1H), 6.84 (d, J = 8.2 Hz, 1H), 6.04 (s, 2H), 4.31 (t, J = 6.3 Hz, 2H), 3.44 – 3.35 (m, 2H), 2.10 – 1.98 (m, 2H), 1.88 – 1.77 (m, 2H), 1.71 – 1.60 (m, 2H). ^{13}C NMR (101 MHz, Chloroform-d) δ 165.8, 151.7, 147.8, 125.3, 124.1, 109.4, 108.0, 101.8, 64.0, 50.7 (d, J = 16.6 Hz), 28.1, 24.6, 23.2. ^{19}F NMR (376 MHz, Chloroform-d) δ 53.67 (t, J = 3.6 Hz.). HRMS (ESI): caled for $C_{13}H_{16}FO_6S^+$ [M + H]$^+$ 319.0646; found 319.0646.

5-(fluorosulfonyl)pentyl 2-(4-isobutylphenyl)propanoate (6c)

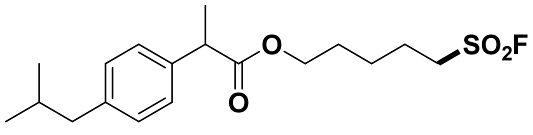

60% (21.5 mg); colorless oil; ^{1}H NMR (400 MHz, Chloroform-d) δ 7.19 (d, J = 8.2 Hz, 2H), 7.10 (d, J = 8.2 Hz, 2H), 4.19 – 3.99 (m, 2H), 3.68 (q, J = 7.1 Hz, 1H), 3.24 (ddd, J = 9.5, 6.9, 4.2 Hz, 2H), 2.45 (d, J = 7.2 Hz, 2H), 1.94 – 1.77 (m, 3H), 1.62 (dq, J = 8.2, 6.1 Hz, 2H), 1.49 (d, J = 7.2 Hz, 3H), 1.45 – 1.33 (m, 2H), 0.90 (d, J = 6.7 Hz, 6H). ^{13}C NMR (101 MHz, Chloroform-d) δ 174.7, 140.6, 137.8, 129.3, 127.1, 63.7, 50.6 (d, J = 16.5 Hz), 45.2, 45.0, 30.2, 27.8, 24.3, 23.0, 22.4, 18.3. ^{19}F NMR (376 MHz, Chloroform-d) δ 53.53 (t, J = 4.0 Hz). HRMS (ESI): caled for $C_{18}H_{28}FO_4S^+$ [M + H]$^+$ 359.1687; found 359.1687.

(4-methyl-1-tosylpyrrolidin-3-yl)methanesulfonyl fluoride (6d)

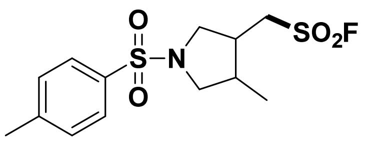

40% (13.4 mg); slight yellow solid: m.p. 94-95 °C; ^{1}H NMR (400 MHz, Chloroform-d) δ 7.73 (d, J = 8.3 Hz, 2H), 7.35 (d, J = 8.0 Hz, 2H), 3.56 (dd, J = 10.7, 7.2 Hz, 1H), 3.44 – 3.31 (m, 2H), 3.27 (dd, J = 10.5, 7.7 Hz, 1H), 3.09 (tt, J = 8.6, 4.0 Hz, 2H),

2.70 – 2.57 (m, 1H), 2.41 – 2.47 (m, 4H), 0.87 (d, *J* = 7.1 Hz, 3H). ^{13}C NMR (101 MHz, Chloroform-*d*) δ 144.0, 133.5, 129.9, 127.4, 53.9, 50.0 (d, *J* = 2.0 Hz), 49.6 (d, *J* = 17.2 Hz), 36.9, 35.2, 21.5, 13.2. ^{19}F NMR (376 MHz, Chloroform-*d*) δ 56.56 (t, *J* = 3.7 Hz.). HRMS (ESI): caled for $C_{13}H_{19}FNO_4S_2^+$ [M + H]$^+$ 336.0734; found 336.0735.

5-(fluorosulfonyl)pentyl 4-(N,N-dipropylsulfamoyl)benzoate (6e)

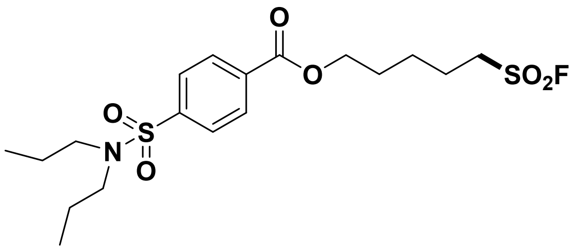

42% (18.4 mg); slight yellow oil; ^{1}H NMR (400 MHz, Chloroform-*d*) δ 8.14 (d, *J* = 8.4 Hz, 2H), 7.87 (d, *J* = 8.5 Hz, 2H), 4.38 (t, *J* = 6.4 Hz, 2H), 3.41 (td, *J* = 7.7, 4.0 Hz, 2H), 3.14 – 3.06 (m, 4H), 2.05 (p, *J* = 7.7 Hz, 2H), 1.92 – 1.81 (m, 2H), 1.73 – 1.61 (m, 2H), 1.55 (h, *J* = 7.4 Hz, 4H), 0.87 (t, *J* = 7.4 Hz, 6H). ^{13}C NMR (101 MHz, Chloroform-*d*) δ 165.2, 144.4, 133.4, 130.2, 127.0, 64.7, 50.7 (d, *J* = 16.7 Hz), 49.9, 28.0, 24.5, 23.2, 21.9, 11.1. ^{19}F NMR (376 MHz, Chloroform-*d*) δ 53.89 (t, *J* = 4.6 Hz.). HRMS (ESI): caled for $C_{18}H_{29}FNO_6S_2^+$ [M + H]$^+$ 438.1415; found 438.1414.

5-(fluorosulfonyl)pentyl 1-(2,2-difluorobenzo[d][1,3]dioxol-5-yl)cyclopropane-1-carboxylate (6f)

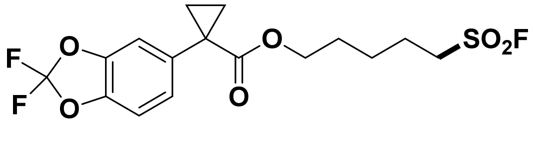

61% (24.1 mg); colorless oil; ^{1}H NMR (400 MHz, Chloroform-*d*) δ 7.06 (s, 1H), 7.04 (d, *J* = 1.7 Hz, 1H), 7.00 – 6.97 (m, 1H), 4.06 (t, *J* = 6.3 Hz, 2H), 3.34 – 3.27 (m, 2H), 1.98 – 1.85 (m, 2H), 1.66 – 1.54 (m, 4H), 1.51 – 1.38 (m, 2H), 1.19 (q, *J* = 4.0 Hz, 2H). ^{13}C NMR (101 MHz, Chloroform-*d*) δ 173.9, 143.4, 142.8, 135.7, 131.7 (t, *J* = 255.0 Hz), 125.6, 112.0, 108.9, 64.4, 50.6 (d, *J* = 16.6 Hz), 29.0, 27.8, 24.3, 23.0, 16.9. ^{19}F NMR (376 MHz, Chloroform-*d*) δ 53.89 (t, *J* = 3.9 Hz.). HRMS (ESI): caled for $C_{16}H_{18}F_3O_6S^+$ [M + H]$^+$ 395.0771; found 395.0772.

5-((4-methyl-2-oxo-2H-chromen-7-yl)oxy)pentane-1-sulfonyl fluoride (6g)

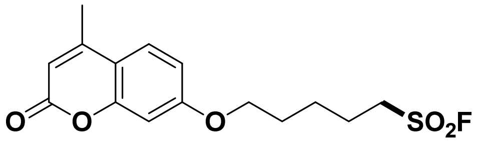

45% (14.8 mg); colorless oil; ^{1}H NMR (400 MHz, Chloroform-*d*) δ 7.49 (d, *J* = 8.8 Hz, 1H), 6.84 (dd, *J* = 8.8, 2.5 Hz, 1H), 6.79 (d, *J* = 2.6 Hz, 1H), 6.14 (q, *J* = 1.2 Hz, 1H), 4.05 (t, *J* = 6.0 Hz, 2H), 3.47 – 3.38 (m, 2H), 2.40 (d, *J* = 1.2 Hz, 3H), 2.06 (p, *J* = 7.7 Hz, 2H), 1.95 – 1.82 (m, 2H), 1.72 (ddd, *J* = 15.3, 9.1, 6.1 Hz, 2H). ^{13}C NMR (101 MHz, Chloroform-*d*) δ 161.8, 161.2, 155.3, 152.5, 125.6, 113.7, 112.5, 112.1, 101.3, 67.7, 50.7 (d, *J* = 16.6 Hz), 28.3, 24.7, 23.3, 18.6. ^{19}F NMR (376 MHz, Chloroform-*d*) δ 53.74 (t, *J* = 4.6 Hz.). HRMS (ESI): caled for $C_{15}H_{18}FO_5S^+$ $[M + H]^+$ 329.0854; found 329.0855.

(8R,9S,10R,13S,14S,17S)-10,13-dimethyl-3-oxo-2,3,6,7,8,9,10,11,12,13,14,15,16,17-tetradecahydro-1H-cyclopenta[a]phenanthren-17-yl 5-(fluorosulfonyl)pentanoate (6h)

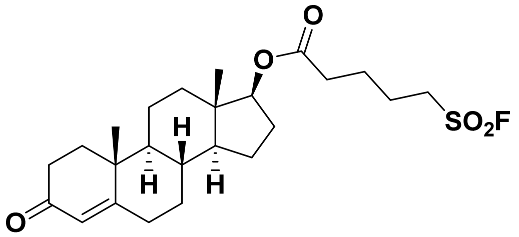

34% (16.0 mg); yellow solid: m.p. 96-97 °C; ^{1}H NMR (400 MHz, Chloroform-*d*) δ 5.73 (s, 1H), 4.62 (dd, *J* = 9.2, 7.7 Hz, 1H), 3.44 – 3.35 (m, 2H), 2.45 – 2.33 (m, 3H), 2.33 – 2.24 (m, 1H), 2.24 – 2.14 (m, 1H), 2.01 (ddt, *J* = 11.9, 9.5, 6.4 Hz, 2H), 1.89 – 1.64 (m, 5H), 1.63 – 1.52 (m, 3H), 1.45 – 1.32 (m, 2H), 1.25 (s, 8H), 1.19 (s, 3H), 0.84 (s, 3H). ^{13}C NMR (101 MHz, Chloroform-*d*) δ 172.4, 170.8, 124.0, 82.8, 53.7, 50.6 (d, *J* = 16.7 Hz), 50.2, 42.5, 38.6, 36.7, 35.7, 35.4, 33.9, 33.4, 32.7, 31.5, 29.7, 27.5, 23.5, 23.2, 22.9, 20.5, 17.4, 12.1. ^{19}F NMR (376 MHz, Chloroform-*d*) δ 53.74 (t, *J* = 4.7 Hz.). HRMS (ESI): caled for $C_{24}H_{36}FO_5S^+$ $[M + H]^+$ 455.2262; found 455.2261.

(8S,9R,13R,14R)-13-methyl-17-oxo-7,8,9,11,12,13,14,15,16,17-decahydro-6H-cyclopenta[a]phenanthren-3-yl 5-(fluorosulfonyl)pentanoate (6i)

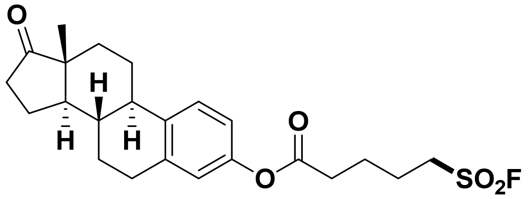

56% (24.5 mg); slight yellow solid: m.p. 97-98 °C; ^{1}H NMR (400 MHz, Chloroform-*d*) δ 7.29 (d, *J* = 8.6 Hz, 1H), 6.88 – 6.78 (m, 2H), 3.44 (td, *J* = 7.5, 4.2 Hz, 2H), 2.91 (dd, *J* = 8.7, 3.9 Hz, 2H), 2.64 (t, *J* = 7.1 Hz, 2H), 2.51 (dd, *J* = 18.8, 8.6 Hz, 1H), 2.45 – 2.36 (m, 1H), 2.29 (td, *J* = 10.7, 4.1 Hz, 1H), 2.22 – 2.03 (m, 4H), 1.96 (ddt, *J* = 12.6, 8.5, 4.7 Hz, 3H), 1.69 – 1.54 (m, 3H), 1.57 – 1.40 (m, 4H), 0.91 (s, 3H). ^{13}C NMR (101 MHz, Chloroform-*d*) δ 220.7, 171.3, 148.3, 138.1, 137.6, 126.5, 121.4, 118.6, 50.6, 50.4 (d, *J* = 2.8 Hz), 47.9, 44.1, 38.0, 35.8, 33.3, 31.5, 29.4, 26.3, 25.7, 23.0, 22.9, 21.6, 13.8. ^{19}F NMR (376 MHz, Chloroform-*d*) δ 53.80 (t, *J* = 4.7 Hz.). HRMS (ESI): caled for $C_{23}H_{30}FO_5S^+$ $[M + H]^+$ 437.1793; found 437.1794.

(R)-2,5,7,8-tetramethyl-2-((4R,8R)-4,8,12-trimethyltridecyl)chroman-6-yl 5-(fluorosulfonyl)pentanoate (6j)

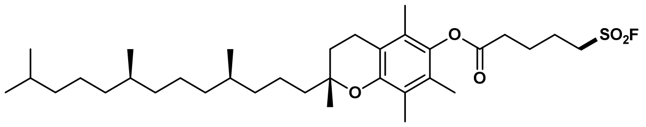

25% (15.0 mg); slight yellow oil; ^{1}H NMR (400 MHz, Chloroform-*d*) δ 3.44 (td, *J* = 7.6, 4.2 Hz, 2H), 2.69 (t, *J* = 7.1 Hz, 2H), 2.59 (t, *J* = 6.8 Hz, 2H), 2.09 (s, 2H), 1.98 (d, *J* = 16.5 Hz, 7H), 1.78 (ddq, *J* = 20.1, 13.4, 6.8 Hz, 2H), 1.62 – 1.49 (m, 4H), 1.43 (s, 1H), 1.26 (s, 16H), 1.24 (s, 4H), 0.86 (tt, *J* = 7.6, 4.6 Hz, 15H). ^{13}C NMR (101 MHz, Chloroform-*d*) δ 171.1, 149.5, 140.3, 126.5, 124.7, 123.2, 117.5, 75.1, 50.6 (d, *J* = 17.0 Hz), 39.4, 37.4 (d, *J* = 3.2 Hz), 37.3, 33.0, 32.8, 32.7, 31.9, 30.3, 29.7, 28.0, 24.8, 24.5, 23.2, 23.1, 22.7 (d, *J* = 2.7 Hz), 22.6, 21.0, 20.6, 19.8, 19.7, 14.1, 13.0, 12.2, 11.8. ^{19}F NMR (376 MHz, Chloroform-*d*) δ 53.73 (t, *J* = 4.9 Hz.). HRMS (ESI): caled for $C_{34}H_{57}FO_5SNa^+$ $[M + Na]^+$ 619.3803; found 619.3805.

((10R,13S)-10,13-dimethyl-3-oxo-2,3,6,7,8,9,10,11,12,13,14,15,16,17-tetradecahydro-1H-cyclopenta[a]phenanthren-17-yl)methyl 5-(fluorosulfonyl)pentanoate (6k)

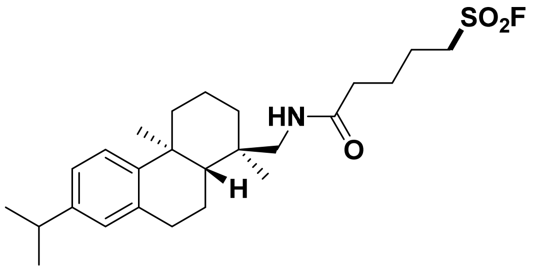

31% (14.0 mg); colorless oil; ^{1}H NMR (400 MHz, Chloroform-*d*) δ 7.17 (d, *J* = 8.2 Hz, 1H), 7.00 (dd, *J* = 8.2, 2.1 Hz, 1H), 6.90 (s, 1H), 5.43 (t, *J* = 6.6 Hz, 1H), 3.34 (td, *J* = 7.6, 4.3 Hz, 2H), 3.25 (dd, *J* = 13.7, 6.4 Hz, 1H), 3.10 (dd, *J* = 13.7, 6.5 Hz, 1H), 2.93 (ddd, *J* = 17.3, 6.9, 1.9 Hz, 1H), 2.81 (dtd, *J* = 17.8, 11.2, 10.0, 4.8 Hz, 2H), 2.34 – 2.26 (m, 1H), 2.22 (t, *J* = 7.1 Hz, 2H), 2.01 – 1.91 (m, 2H), 1.88 – 1.62 (m, 7H), 1.45 – 1.31 (m, 3H), 1.23 (d, *J* = 7.1 Hz, 9H), 0.94 (s, 3H). ^{13}C NMR (101 MHz, Chloroform-*d*) δ 171.5, 147.1, 145.7, 134.7, 126.9, 124.1, 123.9, 50.5 (d, *J* = 16.5 Hz), 49.7, 45.2, 38.3, 37.4, 37.3, 36.2, 35.5, 33.4, 30.1, 25.2, 23.9 (d, *J* = 2.3 Hz), 23.7, 23.0, 18.9, 18.7, 18.5. ^{19}F NMR (376 MHz, Chloroform-*d*) δ 53.67 (t, *J* = 4.6 Hz.). HRMS (ESI): caled for $C_{25}H_{39}FNO_3S^+$ $[M + H]^+$ 452.2629; found 452.2627.

2-(benzo[d]thiazol-2-yl)-5-oxo-5-(m-tolyl)pentane-1-sulfonyl fluoride (8a)

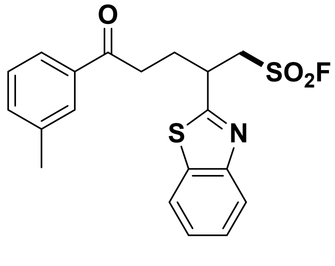

66% (26.0 mg); yellow solid: m.p. 107-108 °C; ^{1}H NMR (400 MHz, Chloroform-*d*) ^{1}H NMR (400 MHz, Chloroform-*d*) δ 8.00 (dt, *J* = 8.3, 0.9 Hz, 1H), 7.91 – 7.84 (m, 1H), 7.65 (d, *J* = 11.1 Hz, 2H), 7.50 (ddd, *J* = 8.3, 7.2, 1.3 Hz, 1H), 7.41 (ddd, *J* = 8.2, 7.2, 1.2 Hz, 1H), 7.37 – 7.28 (m, 2H), 4.32 (ddd, *J* = 14.8, 7.6, 4.1 Hz, 1H), 4.04 (ddt, *J* = 8.8, 7.5, 5.2 Hz, 1H), 3.89 (dt, *J* = 14.8, 5.0 Hz, 1H), 3.11 – 2.93 (m, 2H), 2.63 – 2.38 (m, 2H), 2.37 (s, 3H). ^{13}C NMR (101 MHz, Chloroform-*d*) δ 198.3, 169.0, 152.9, 138.5, 136.4, 134.8, 134.1, 131.6, 128.5 (d, *J* = 4.6 Hz), 126.5, 125.6, 125.2, 123.2, 121.8, 54.9 (d, *J* = 16.1 Hz), 38.8, 34.8, 29.4, 21.3. ^{19}F NMR (376 MHz, Chloroform-*d*) δ 58.23. (t, *J* = 4.2 Hz). HRMS (ESI): caled for $C_{19}H_{19}FNO_3S_2^+$ $[M + H]^+$ 392.0785; found 392.0786.

2-(benzo[d]thiazol-2-yl)-5-(4-fluorophenyl)-5-oxopentane-1-sulfonyl fluoride (8b)

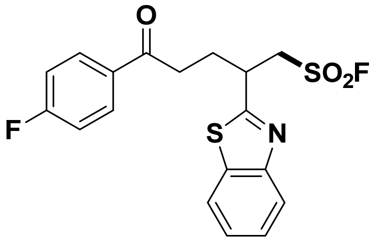

75% (29.5 mg); yellow solid: m.p. 105-106 °C; ^{1}H NMR (400 MHz, Chloroform-*d*) δ 8.03 – 7.96 (m, 1H), 7.88 (m, 3H), 7.46 (m, 2H), 7.14 – 7.04 (m, 2H), 4.31 (m, 1H), 4.04 (m, 1H), 3.89 (m, 1H), 3.09 – 2.91 (m, 2H), 2.49 (m, 2H). ^{13}C NMR (101 MHz, Chloroform-*d*) δ 196.5, 168.0 (d, *J* = 172.0 Hz), 164.6, 152.9, 134.7, 132.8, 130.6 (d, *J* = 9.2 Hz), 126.5, 125.7, 123.2, 121.8, 115.8 (d, *J* = 21.8 Hz), 54.9 (d, *J* = 16.3 Hz), 38.8, 34.7, 29.3. ^{19}F NMR (376 MHz, Chloroform-*d*) δ 58.28 (t, *J* = 4.8 Hz), -95.97 – -112.81 (m). HRMS (ESI): caled for $C_{18}H_{16}F_2NO_3S_2^+$ $[M + H]^+$ 396.0489; found 396.0488.

2-(benzo[d]thiazol-2-yl)-5-(furan-2-yl)-5-oxopentane-1-sulfonyl fluoride (8c)

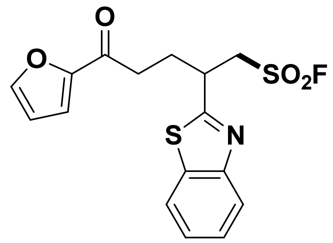

27% (10.0 mg); yellow solid: m.p. 123-124 °C; ^{1}H NMR (400 MHz, Chloroform-*d*) δ 8.20 – 8.13 (m, 1H), 8.00 – 7.95 (m, 1H), 7.62 – 7.49 (m, 2H), 7.39 (dd, *J* = 1.8, 0.8 Hz, 1H), 6.27 (ddd, *J* = 19.8, 3.3, 1.3 Hz, 2H), 3.86 (m, 1H), 3.78 – 3.60 (m, 2H), 3.26 (t, *J* = 7.2 Hz, 2H), 2.43 – 2.18 (m, 2H). ^{13}C NMR (101 MHz, Chloroform-*d*) δ 193.7, 165.7, 153.4, 151.0, 142.7, 137.2, 127.8, 127.1, 125.4, 122.5, 110.5, 108.5, 54.6 (d, *J* = 14.7 Hz), 35.6, 34.3, 27.3 (d, *J* = 2.0 Hz). ^{19}F NMR (376 MHz, Chloroform-*d*) δ 58.17 (d, *J* = 5.4 Hz). HRMS (ESI): caled for $C_{16}H_{15}FNO_4S_2^+$ $[M + H]^+$ 368.0421; found 368.0420.

2-(benzo[d]thiazol-2-yl)-5-oxo-5-(thiophen-2-yl)pentane-1-sulfonyl fluoride (8d)

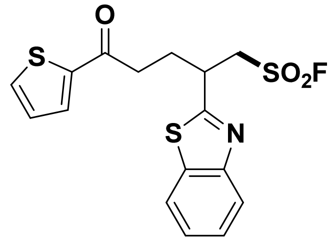

26% (10.0 mg); yellow oil; ^{1}H NMR (400 MHz, Chloroform-*d*) δ 8.03 – 7.97 (m, 1H), 7.91 – 7.84 (m, 1H), 7.65 – 7.58 (m, 2H), 7.46 (m, 2H), 7.08 (dd, *J* = 4.9, 3.8 Hz, 1H), 4.31 (m, 1H), 4.04 (m, 1H), 3.88 (ddd, *J* = 14.8, 5.5, 4.6 Hz, 1H), 3.10 – 2.88 (m, 2H), 2.59 – 2.38 (m, 2H). ^{13}C NMR (101 MHz, Chloroform-*d*) δ 190.9, 168.8, 152.9, 143.5, 134.8, 134.0, 132.0, 128.1, 126.5, 125.6, 123.2, 121.8, 54.8 (d, *J* = 16.3 Hz), 38.8, 35.4, 29.5. ^{19}F NMR (376

MHz, Chloroform-*d*) δ 58.27 (t, *J* = 3.2 Hz). HRMS (ESI): caled for $C_{16}H_{15}FNO_3S_3^+$ [M + H]$^+$ 384.0193; found 384.0192.

5-oxo-5-phenyl-2-(thiazol-2-yl)pentane-1-sulfonyl fluoride (8e)

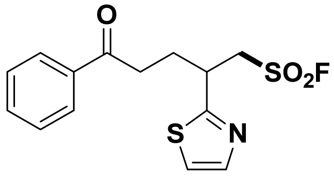

70% (23.0 mg); white solid: m.p. 102-103 °C; ^{1}H NMR (400 MHz, Chloroform-*d*) δ 7.90 – 7.85 (m, 2H), 7.79 (d, *J* = 3.3 Hz, 1H), 7.63 – 7.50 (m, 1H), 7.44 (m, 2H), 7.30 (d, *J* = 3.3 Hz, 1H), 4.19 (m, 1H), 3.99 (m, 1H), 3.83 (dt, *J* = 14.6, 5.0 Hz, 1H), 3.06 – 2.88 (m, 2H), 2.52 – 2.29 (m, 2H). ^{13}C NMR (101 MHz, Chloroform-*d*) δ 198.1, 168.0, 143.1, 136.4, 133.4, 128.7, 127.9, 119.3, 55.4 (d, *J* = 15.8 Hz), 37.9, 34.7, 29.6 (d, *J* = 1.6 Hz). ^{19}F NMR (376 MHz, Chloroform-*d*) δ 58.21 (t, *J* = 4.7 Hz). HRMS (ESI): caled for $C_{14}H_{14}FNO_3S_2^+$ [M + H]$^+$ 328.0472; found 328.0471.

2-(benzo[d]thiazol-2-yl)-6,6-dimethyl-5-oxoheptane-1-sulfonyl fluoride (8f)

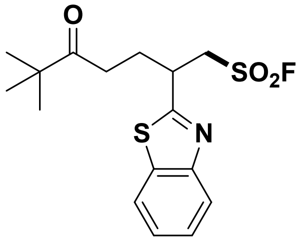

57% (20.5 mg); slight yellow oil; ^{1}H NMR (400 MHz, Chloroform-*d*) δ 7.94 (dd, *J* = 46.6, 7.3 Hz, 2H), 7.47 (m, 2H), 4.27 (m, 1H), 3.93 (m, 1H), 3.87 – 3.77 (m, 1H), 2.61 – 2.44 (m, 2H), 2.38 – 2.17 (m, 2H), 1.09 (s, 9H). ^{13}C NMR (101 MHz, Chloroform-*d*) δ 214.2, 169.1, 152.9, 147.5, 126.5, 125.6, 123.2, 121.8, 54.9 (d, *J* = 16.2 Hz), 44.1, 38.7, 32.7, 29.4, 26.5. ^{19}F NMR (376 MHz, Chloroform-*d*) δ 58.08 (t, *J* = 4.2 Hz) HRMS (ESI): caled for $C_{16}H_{21}FN_2O_3S_2^+$ [M + H]$^+$ 358.0942; found 358.0941.

2-(benzo[d]thiazol-2-yl)-5-cyclopropyl-5-oxopentane-1-sulfonyl fluoride (8g)

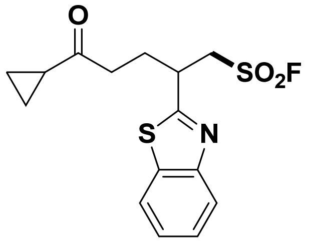

73% (25.0 mg); slight yellow oil; ^{1}H NMR (400 MHz, Chloroform-*d*) δ 7.95 (dd, *J* = 50.0, 7.5 Hz, 2H), 7.60 – 7.38 (m, 2H), 4.28 (m, 1H), 3.97 – 3.88 (m, 1H), 3.83 (dt, *J* = 14.7, 5.0 Hz, 1H), 2.76 – 2.52 (m, 2H), 2.42 – 2.20 (m, 2H), 1.83 (ddd, *J* = 12.4, 7.8, 4.5 Hz, 1H), 1.05 – 0.96 (m, 2H), 0.91 – 0.81 (m, 2H). ^{13}C NMR (101 MHz, Chloroform-*d*) δ 208.7, 169.0, 152.9, 134.7, 126.4, 125.6, 123.1, 121.8, 54.7 (d, *J* = 16.1 Hz), 39.4, 38.6, 29.1, 20.6, 11.1 (d, *J* = 3.2 Hz). ^{19}F NMR (376 MHz, Chloroform-*d*) δ 58.10 (t, *J* = 4.3 Hz). HRMS (ESI): caled for $C_{15}H_{17}FNO_3S_2^+$ $[M + H]^+$ 342.0629; found 342.0628.

2-(benzo[d]thiazol-2-yl)-5-oxohexane-1-sulfonyl fluoride (8h)

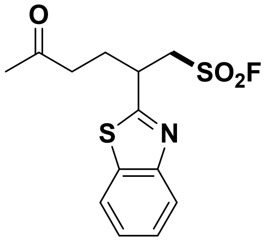

48% (15.0 mg); slight yellow oil; ^{1}H NMR (400 MHz, Chloroform-*d*) δ 7.94 (dd, *J* = 47.4, 7.9 Hz, 2H), 7.47 (m, 2H), 4.26 (m, 1H), 3.97 – 3.87 (m, 1H), 3.82 (ddd, *J* = 14.7, 5.6, 4.6 Hz, 1H), 2.58 – 2.45 (m, 2H), 2.38 – 2.21 (m, 2H), 2.10 (s, 3H). ^{13}C NMR (101 MHz, Chloroform-*d*) δ 206.5, 168.8, 152.9, 134.7, 126.5, 125.6, 123.2, 121.8, 54.7 (d, *J* = 16.5 Hz), 39.6, 38.6, 30.0, 28.9. ^{19}F NMR (376 MHz, Chloroform-*d*) δ 58.14 (t, *J* = 4.5 Hz). HRMS (ESI): caled for $C_{13}H_{15}FNO_3S_2^+$ $[M + H]^+$ 316.0472; found 316.0471.

2-cyano-2-methyl-5-oxo-5-phenylpentane-1-sulfonyl fluoride (8i)

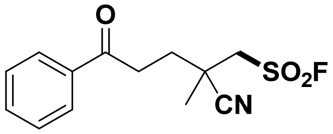

42% (12.0 mg); slight yellow oil; ^{1}H NMR (400 MHz, Chloroform-*d*) δ 7.99 (d, *J* = 7.2 Hz, 2H), 7.62 (t, *J* = 7.4 Hz, 1H), 7.50 (t, *J* = 7.7 Hz, 1H), 3.71 (ddd, *J* = 55.4, 15.0, 3.2 Hz, 2H), 3.29 (t, *J* = 7.9 Hz, 2H), 2.43 – 2.20 (m, 2H), 1.71 (s, 3H). ^{13}C NMR (101 MHz, Chloroform-*d*) δ 197.0, 136.0, 133.8, 128.9, 128.1, 119.9, 57.8 (d, *J* = 17.2 Hz), 34.6, 33.8, 33.1 (d, *J* = 1.9 Hz), 23.7. ^{19}F NMR (376 MHz, Chloroform-*d*) δ 65.35 (t, *J* = 3.8 Hz). HRMS (ESI): caled for $C_{13}H_{15}FNO_3S^+$ $[M + H]^+$ 284.0751; found 284.0750.

2-cyano-5-oxo-5-(p-tolyl)pentane-1-sulfonyl fluoride (8j)

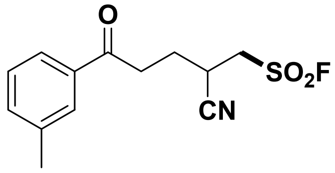

71% (20.0 mg); slight yellow solid: m.p. 88-89 °C; ^{1}H NMR (400 MHz, Chloroform-*d*) δ 7.77 (d, *J* = 7.2 Hz, 2H), 7.46 – 7.34 (m, 2H), 3.80 (m, 1H), 3.68 (dt, *J* = 15.0, 5.3 Hz, 1H), 3.54 (ddt, *J* = 10.0, 8.5, 4.8 Hz, 1H), 3.35 – 3.27 (m, 2H), 2.43 (s, 3H), 2.36 (m, 1H), 2.22 – 2.09 (m, 1H). ^{13}C NMR (101 MHz, Chloroform-*d*) δ 197.5, 138.8, 136.0, 134.7, 128.7 (d, *J* = 17.7 Hz), 125.3, 117.6, 52.3 (d, *J* = 19.1 Hz), 34.8, 26.9, 26.0, 21.4. ^{19}F NMR (376 MHz, Chloroform-*d*) δ 58.03 (t, *J* = 6.4 Hz). HRMS (ESI): caled for $C_{13}H_{15}FNO_3S^+$ $[M + H]^+$ 284.0751; found 284.0750.

5-(4-bromophenyl)-2-cyano-5-oxopentane-1-sulfonyl fluoride (8k)

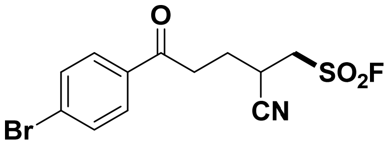

58% (20.1 mg); slight yellow solid: m.p. 107-108 °C; ^{1}H NMR ^{1}H NMR (400 MHz, Chloroform-*d*) δ 7.83 (d, *J* = 8.6 Hz, 2H), 7.65 (d, *J* = 8.6 Hz, 2H), 3.81 (ddd, *J* = 15.0, 8.3, 3.2 Hz, 1H), 3.68 (dt, *J* = 15.0, 5.3 Hz, 1H), 3.54 (ddt, *J* = 10.1, 8.2, 4.9 Hz, 1H), 3.37 – 3.18 (m, 2H), 2.44 – 2.31 (m, 1H), 2.26 – 2.09 (m, 1H). ^{13}C NMR (101 MHz, Chloroform-*d*) δ 196.3, 134.6, 132.2, 129.5, 129.2, 52.2 (d, *J* = 19.1 Hz), 34.7, 26.8, 25.8. ^{19}F NMR (376 MHz, Chloroform-*d*) δ 59.13 (t, *J* = 5.6 Hz). HRMS (ESI): caled for $C_{12}H_{12}BrFNO_3S^+$ $[M + H]^+$ 347.9700; found 347.9703.

2-cyano-5-(furan-2-yl)-5-oxopentane-1-sulfonyl fluoride (8l)

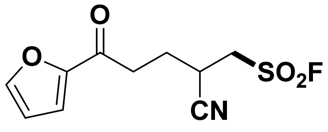

69% (18.0 mg); slight yellow oil; ^{1}H NMR (400 MHz, Chloroform-*d*) δ 7.78 (dd, *J* = 3.8, 1.2 Hz, 1H), 7.71 (dd, *J* = 4.9, 1.1 Hz, 1H), 7.18 (dd, *J* = 4.9, 3.8 Hz, 1H), 3.80 (ddd, *J* = 15.0, 8.5, 3.3 Hz, 1H), 3.68 (dt, *J* = 15.1, 5.3 Hz, 1H), 3.54 (ddt, *J* = 9.9, 8.4, 4.9 Hz, 1H), 3.36 – 3.16 (m, 2H), 2.44 – 2.31 (m, 1H), 2.26 – 2.07 (m, 1H). ^{13}C NMR (101 MHz, Chloroform-*d*) δ 190.1, 143.0, 134.6, 132.5, 128.4, 117.4, 52.1 (d, *J* = 19.1 Hz), 35.2, 26.8, 25.90. ^{19}F NMR (376 MHz, Chloroform-*d*) δ 59.10 (t, *J* = 4.8 Hz). HRMS (ESI): caled for $C_{10}H_{11}FNO_4S^+$ $[M + H]^+$ 260.0388; found 260.0363.

5-methyl-4,7-diphenyl-4,7-dihydro-3H-[1,2]oxathiino[6,5-c]pyrazole 2,2-dioxide (10)

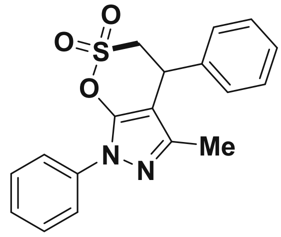

50% (17.2 mg); White solid; ^{1}H NMR (400 MHz, Chloroform-*d*) δ 7.69 – 7.63 (m, 2H), 7.51 – 7.30 (m, 8H), 4.53 (dd, *J* = 11.4, 5.9 Hz, 1H), 3.74 (dd, *J* = 14.2, 5.9 Hz, 1H), 3.41 (dd, *J* = 14.2, 11.4 Hz, 1H), 1.72 (s, 3H). ^{13}C NMR (101 MHz, Chloroform-*d*) δ 147.0, 144.0, 138.1, 137.1, 129.4, 129.3, 128.5, 128.0, 127.2, 99.0, 52.1, 37.9, 13.6. HRMS (ESI): caled for $C_{18}H_{17}N_2O_3S^+$ $[M + H]^+$ 341.0955; found 341.0953.

7,7-dimethyl-4-phenyl-4,6,7,8-tetrahydrobenzo[e][1,2]oxathiin-5(3H)-one 2,2-dioxide (12)

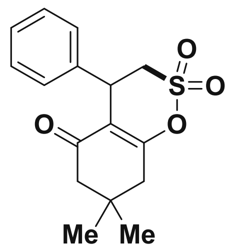

40% (12.0 mg); white solid; ^{1}H NMR (400 MHz, Chloroform-*d*) δ 7.32 (t, *J* = 7.3 Hz, 2H), 7.26 (d, *J* = 7.1 Hz, 1H), 7.19 (dd, *J* = 7.1, 1.8 Hz, 2H), 4.47 (ddt, *J* = 9.2, 7.2, 2.2 Hz, 1H), 3.70 (dd, *J* = 14.4, 7.3 Hz, 1H), 3.45 (dd, *J* = 14.4, 8.9 Hz, 1H), 2.59 – 2.47 (m, 2H), 2.28 (d, *J* = 3.3 Hz, 2H), 1.18 (s, 3H), 1.13 (s, 3H). ^{13}C NMR (101 MHz, Chloroform-*d*) δ 195.5, 165.6, 139.6, 129.0, 127.6, 116.9, 52.0, 50.7, 42.4, 39.8, 28.6, 27.9. HRMS (ESI): caled for $C_{16}H_{19}O_4S^+$ $[M + H]^+$ 307.0999; found 307.0995.

(8R,9S,13S,14S)-13-methyl-17-oxo-7,8,9,11,12,13,14,15,16,17-decahydro-6H-cyclopenta[a]phenanthren-3-yl (E)-2-phenylethene-1-sulfonate (14)

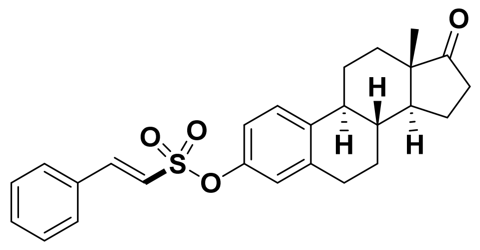

60% (26.3 mg); white solid; ^{1}H NMR (400 MHz, Chloroform-*d*) δ 7.56 (d, *J* = 15.6 Hz, 1H), 7.51 – 7.39 (m, 5H), 7.26 (d, *J* = 9.3 Hz, 1H), 7.01 (dd, *J* = 6.1, 2.8 Hz, 2H), 6.88 (d, *J* = 15.5 Hz, 1H), 2.90 (dd, *J* = 9.0, 4.3 Hz, 2H), 2.50 (dd, *J* = 18.9, 8.7 Hz, 1H), 2.37 (dt, *J* = 9.0, 3.2 Hz, 1H), 2.32 – 2.23 (m, 1H), 2.21 – 1.92 (m, 4H), 1.70 – 1.36 (m, 6H), 0.90 (s, 3H). ^{13}C NMR (101 MHz, Chloroform-d) δ 220.6, 147.4, 145.8, 139.0, 138.7, 131.9, 131.7, 129.3, 128.6, 126.7, 122.5, 121.0, 119.3, 50.4,

47.9, 44.1, 37.9, 35.8, 31.5, 29.4, 26.2, 25.7, 21.6, 13.8. HRMS (ESI): $C_{26}H_{29}O_4S^+$ [M + H]$^+$ 437.1781, found 437.1780.

5-((((R)-2,5,7,8-tetramethyl-2-((4R,8R)-4,8,12-trimethyltridecyl)chroman-6-yl)oxy)sulfonyl)pentyl 2,2-difluorobenzo[d][1,3]dioxole-5-carboxylate (16)

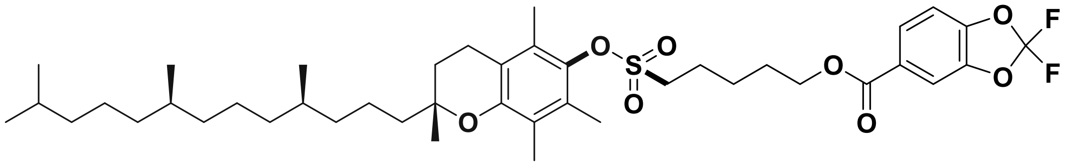

70% (0.025 mmol) (13.4 mg); colorless oil; ^{1}H NMR (400 MHz, Chloroform-*d*) δ 7.09 – 7.02 (m, 2H), 6.99 – 6.95 (m, 1H), 4.07 (t, *J* = 6.4 Hz, 2H), 3.34 – 3.26 (m, 2H), 2.59 (t, *J* = 6.8 Hz, 2H), 2.21 (d, *J* = 12.8 Hz, 6H), 2.09 (s, 3H), 2.06 – 1.97 (m, 2H), 1.79 (dh, *J* = 20.0, 6.7 Hz, 2H), 1.70 – 1.58 (m, 4H), 1.58 – 1.46 (m, 7H), 1.34 – 1.22 (m, 10H), 1.22 – 1.07 (m, 5H), 0.90 – 0.81 (m, 14H). ^{13}C NMR (101 MHz, Chloroform-*d*) δ 173.9, 150.2, 143.4, 142.8, 139.0, 135.8, 131.7, 128.7, 127.3, 125.7, 123.8, 118.0, 112.0, 108.9, 75.4, 64.6, 51.5, 40.0, 39.4, 37.5, 37.3, 32.8, 32.7, 31.0, 29.0, 28.1, 28.0, 24.8, 24.5, 23.9, 23.3, 22.7, 22.6, 21.0, 20.7, 19.8, 19.7, 16.9, 14.5, 13.6, 12.0. ^{19}F NMR (376 MHz, Chloroform-*d*) δ -49.92. HRMS (ESI): caled for $C_{42}H_{63}F_2O_8S^+$ [M + H]$^+$ 765.4206; found 765.4205.

5-chloro-2-phenylpent-2-ene-1-sulfonyl fluoride (19)

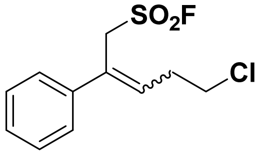

11% (2.9 mg); colorless oil; ^{1}H NMR (400 MHz, Chloroform-*d*) δ 7.38 – 7.21 (m, 5H), 6.18 (t, *J* = 7.4 Hz, 1H), 4.53 (d, *J* = 2.5 Hz, 2H), 3.63 (t, *J* = 6.4 Hz, 2H), 2.76 (q, *J* = 6.7 Hz, 2H). ^{13}C NMR (101 MHz, Chloroform-*d*) δ 135.3, 128.8, 128.8, 128.5, 128.4, 126.5, 52.5 (d, *J* = 16.8 Hz), 43.2, 32.6. ^{19}F NMR (376 MHz, Chloroform-*d*) δ 56.81. HRMS (ESI): caled for $C_{11}H_{12}ClFO_2SNa^+$ [M + Na]$^+$ 285.0123; found 285.0127.

X. X-ray crystallography data for 2b

Table 9 Crystal data and structure refinement for 2b.

Identification code	2b
Empirical formula	$C_{16}H_{11}F_{6.96}N_2O_5S_2$
Formula weight	507.53
Temperature/K	193.0
Crystal system	orthorhombic
Space group	Pnma
a/Å	17.4014(3)
b/Å	8.0234(2)
c/Å	14.1405(3)
α/°	90
β/°	90
γ/°	90
Volume/Å3	1974.27(7)
Z	4
ρcalcg/cm3	1.708
μ/mm-1	2.191
F(000)	1022.0
Crystal size/mm3	0.17 × 0.13 × 0.11
Radiation	GaKα (λ = 1.34139)
2Θ range for data collection/°	7.008 to 107.992
Index ranges	-21 ≤ h ≤ 19, -9 ≤ k ≤ 9, -17 ≤ l ≤ 17
Reflections collected	17641
Independent reflections	1946 [Rint = 0.0560, Rsigma = 0.0329]
Data/restraints/parameters	1946/1/177
Goodness-of-fit on F2	1.038
Final R indexes [I>=2σ (I)]	R1 = 0.0640, wR2 = 0.1637
Final R indexes [all data]	R1 = 0.0830, wR2 = 0.1765
Largest diff. peak/hole / e Å-3	0.96/-0.90

Crystal structure determination of 2b

Crystal Data for $C_{16}H_{11}F_{6.955}N_2O_5S_2$ (M = 507.53 g/mol): orthorhombic, space group Pnma (no. 62), a = 17.4014 (3) Å, b= 8.0234 (2) Å, c= 14.1405(3) Å, V= 1974.27(7)

Å^3, Z= 4, T= 193.0 K, μ(GaKα)= 2.191 mm^{-1}, $Dcalc$= 1.708 g/cm^3, 17641 reflections measured (7.008° ≤ 2Θ ≤ 107.992°), 1946 unique (R_{int} = 0.0560, R_{sigma} = 0.0329) which were used in all calculations. The final R_1 was 0.0640 (I > 2σ(I)) and wR_2 was 0.1765 (all data).

XI. NMR Spectra of 2, 3, 4, 6, 8, 10, 12, 14, 16, 19

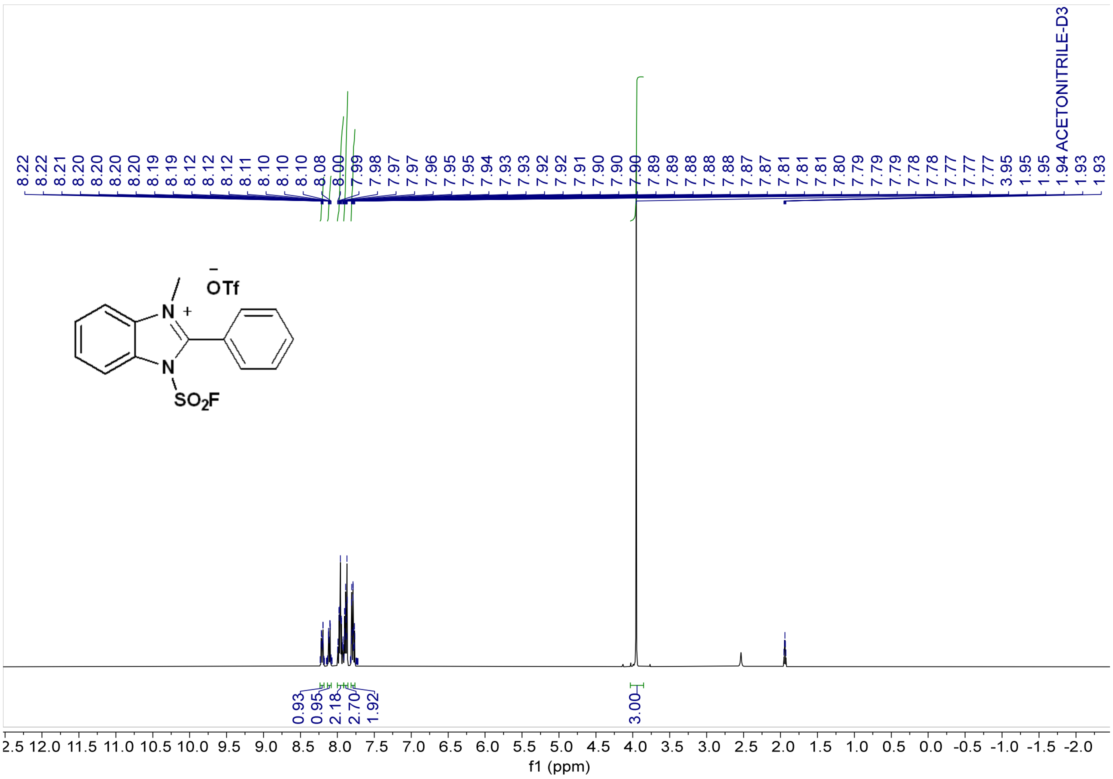

Supplementary Figure 12. ^{1}H NMR (400 MHz, room temperature, CD_3CN) spectra of product **2a**

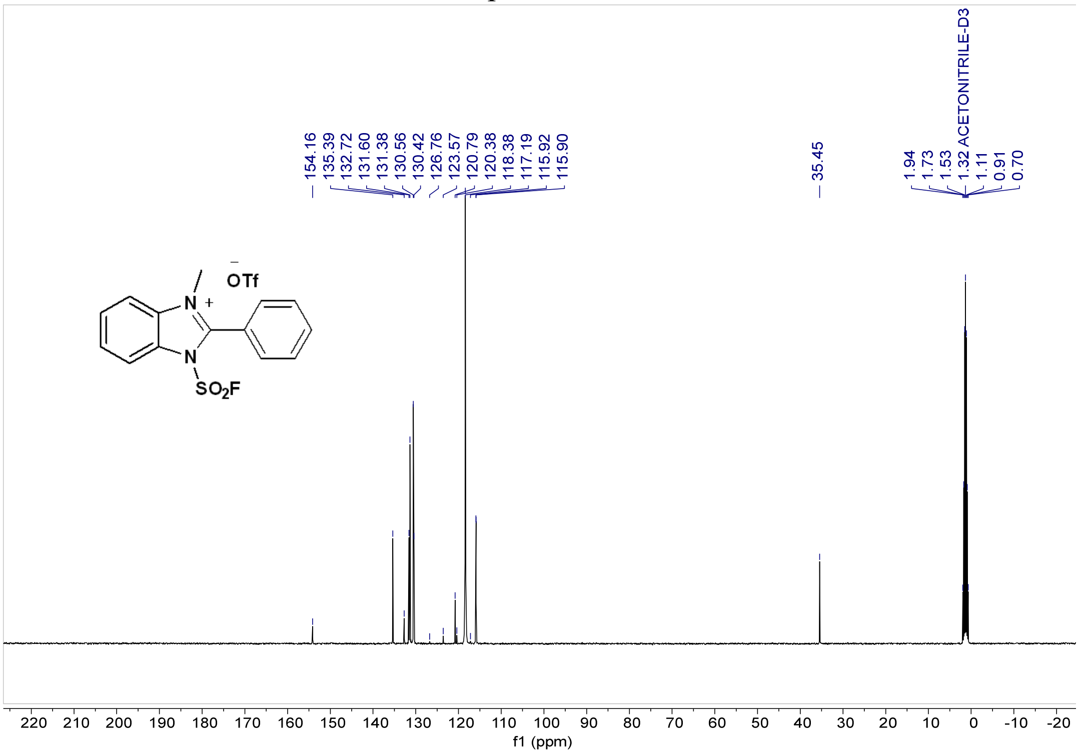

Supplementary Figure 13. ^{13}C NMR (101 MHz, room temperature, CD_3CN) spectra of product **2a**

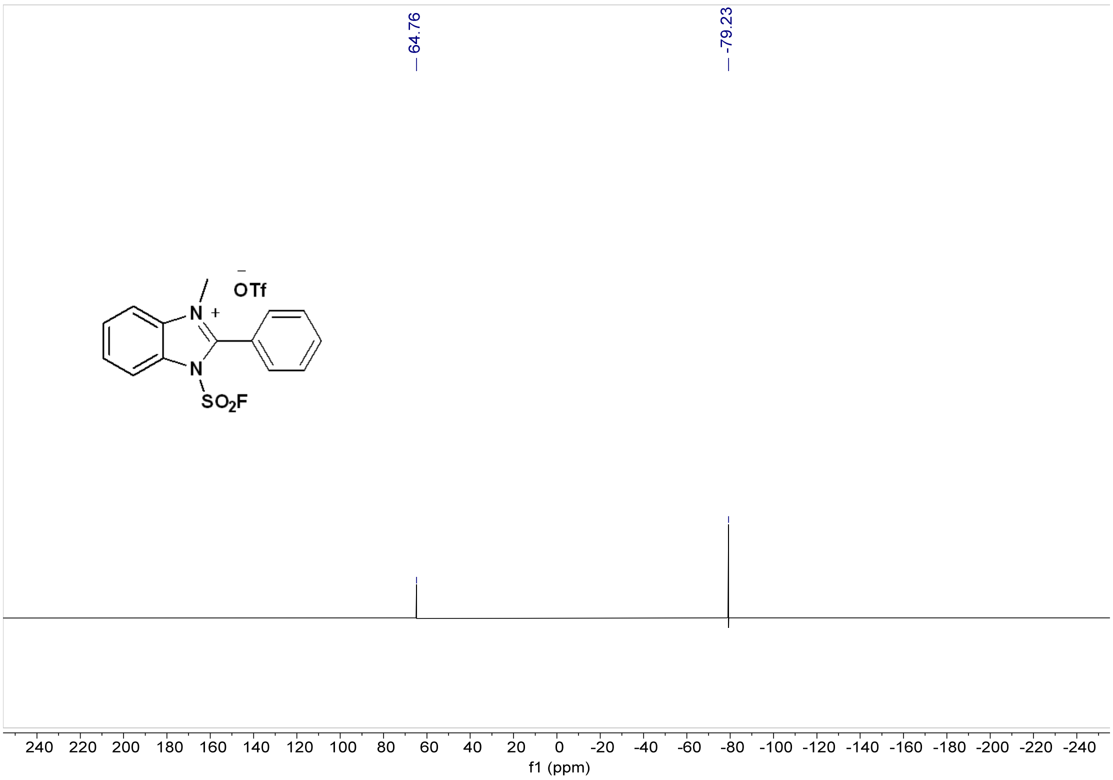

Supplementary Figure 14. ^{19}F NMR (376 MHz, room temperature, CD_3CN) spectra of product **2a**

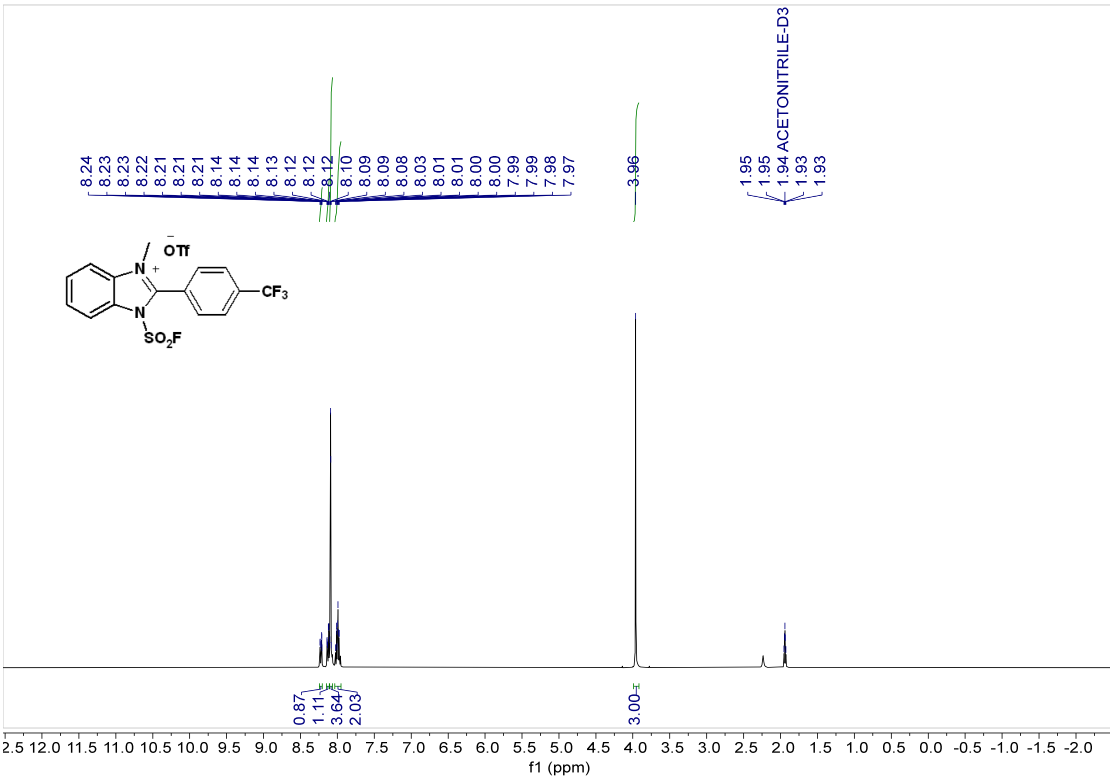

Supplementary Figure 15. ^{1}H NMR (400 MHz, room temperature, CD_3CN) spectra of product **2b**

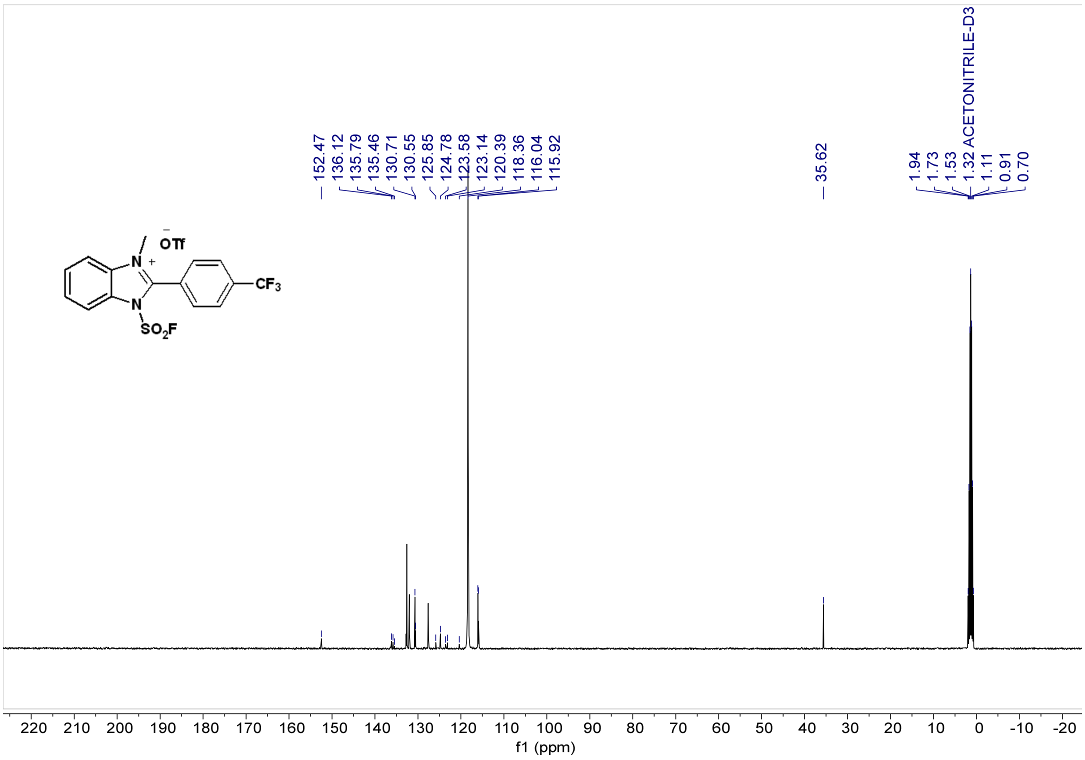

Supplementary Figure 16. ^{13}C NMR (101 MHz, room temperature, CD_3CN) spectra of product **2b**

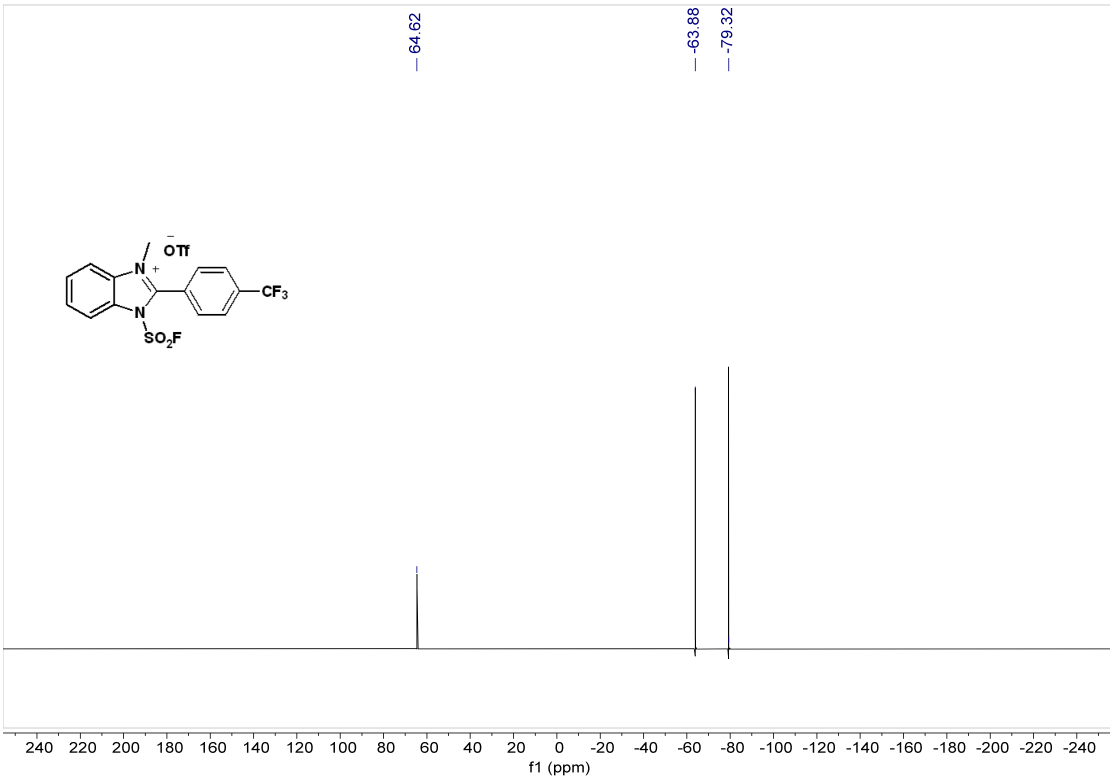

Supplementary Figure 17. ^{19}F NMR (376 MHz, room temperature, CD_3CN) spectra of product **2b**

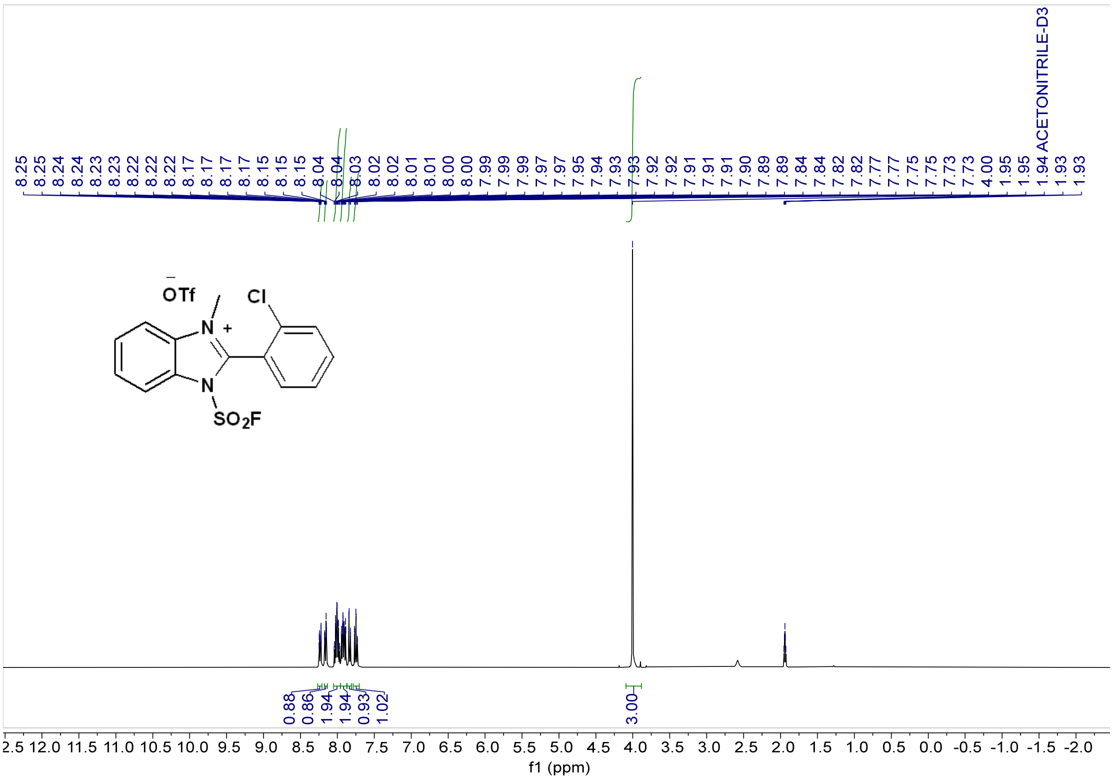

Supplementary Figure 18. ^{1}H NMR (400 MHz, room temperature, CD_3CN) spectra of product **2c**

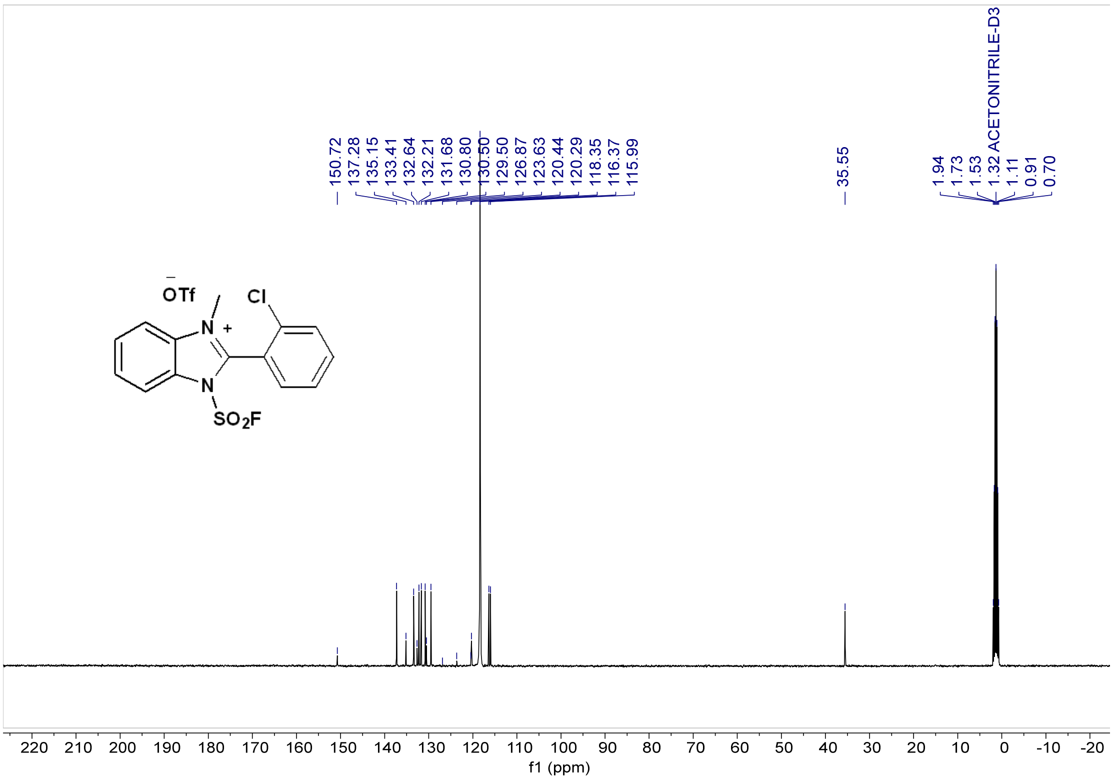

Supplementary Figure 19. ^{13}C NMR (101 MHz, room temperature, CD_3CN) spectra of product **2c**

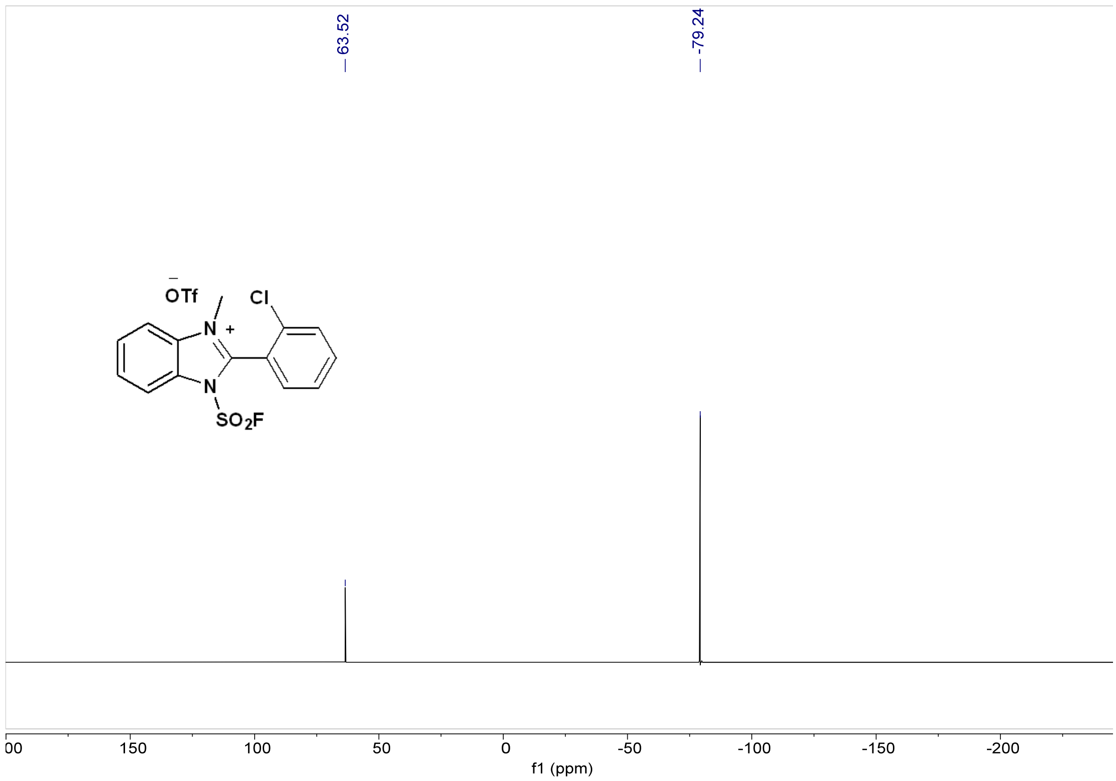

Supplementary Figure 20. ^{19}F NMR (376 MHz, room temperature, CD_3CN) spectra of product **2c**

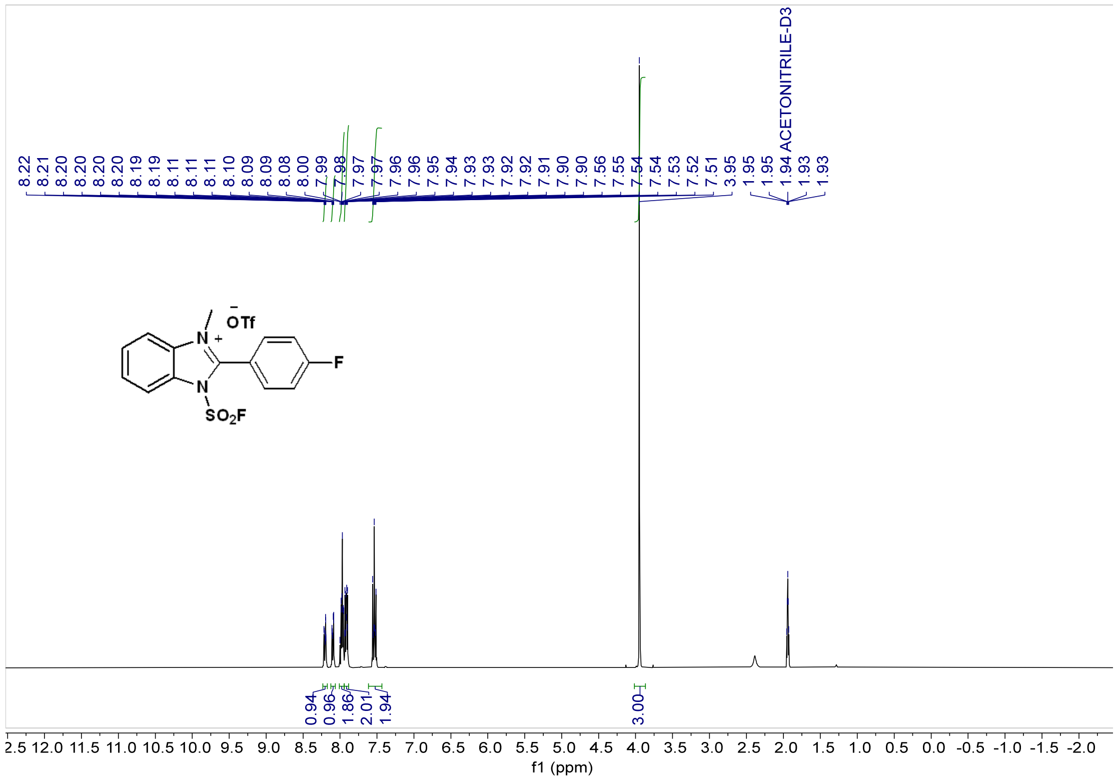

Supplementary Figure 21. ^{1}H NMR (400 MHz, room temperature, CD_3CN) spectra of product **2d**

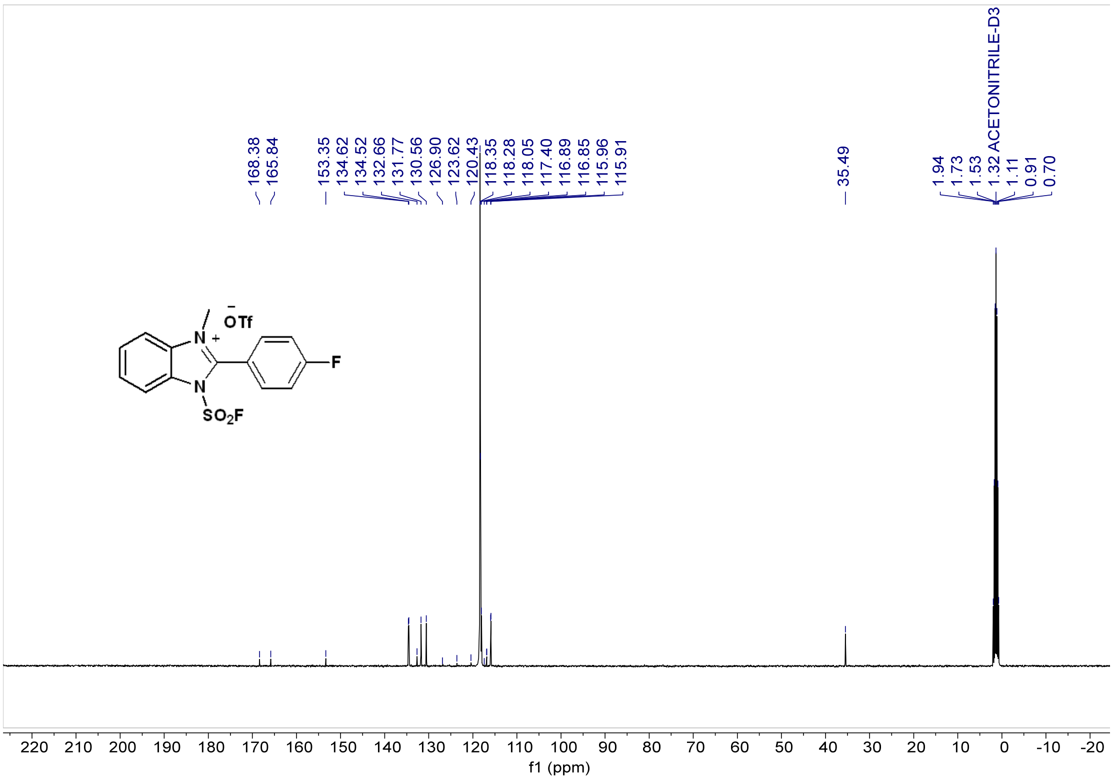

Supplementary Figure 22. ^{13}C NMR (101 MHz, room temperature, CD_3CN)spectra of product **2d**

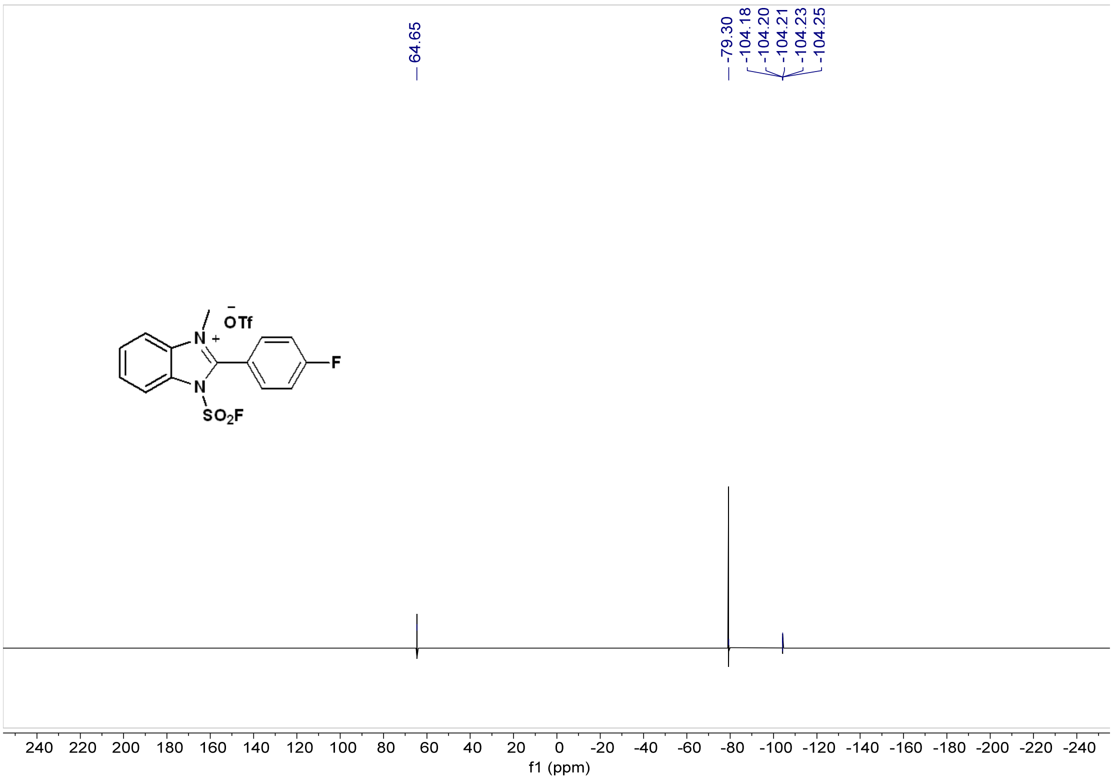

Supplementary Figure 23. ^{19}F NMR (376 MHz, room temperature, CD_3CN) spectra of product **2d**

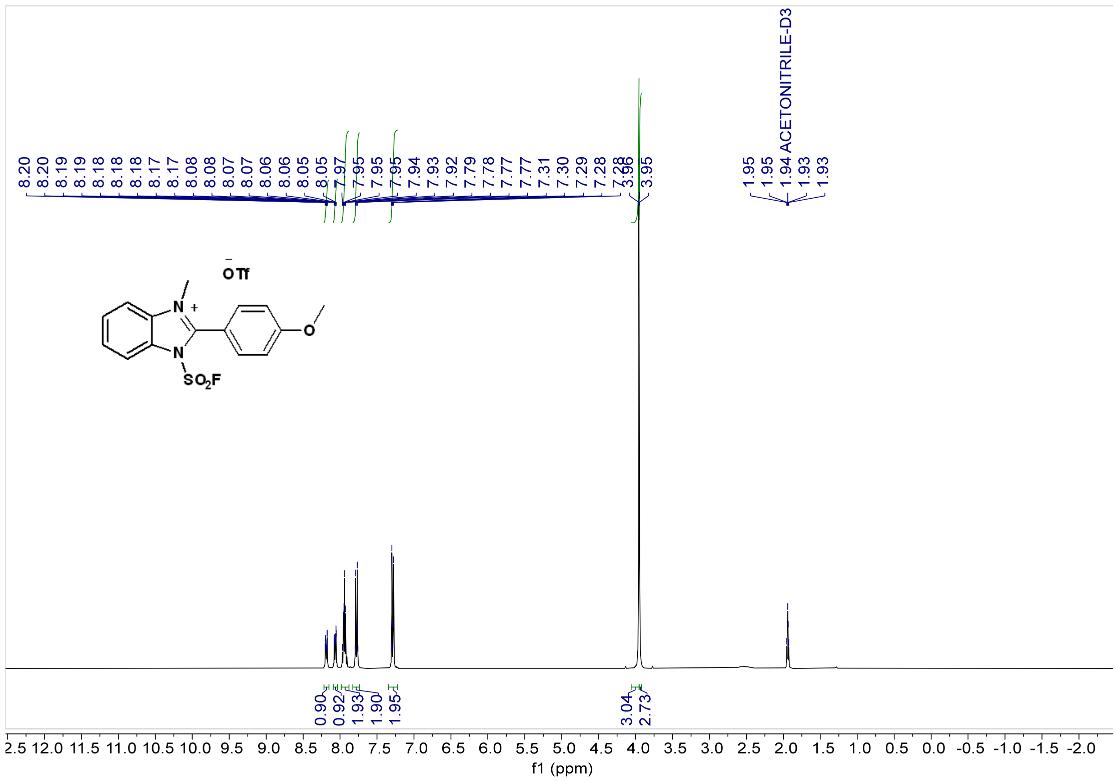

Supplementary Figure 24. ^{1}H NMR (400 MHz, room temperature, CD_3CN) spectra of product **2e**

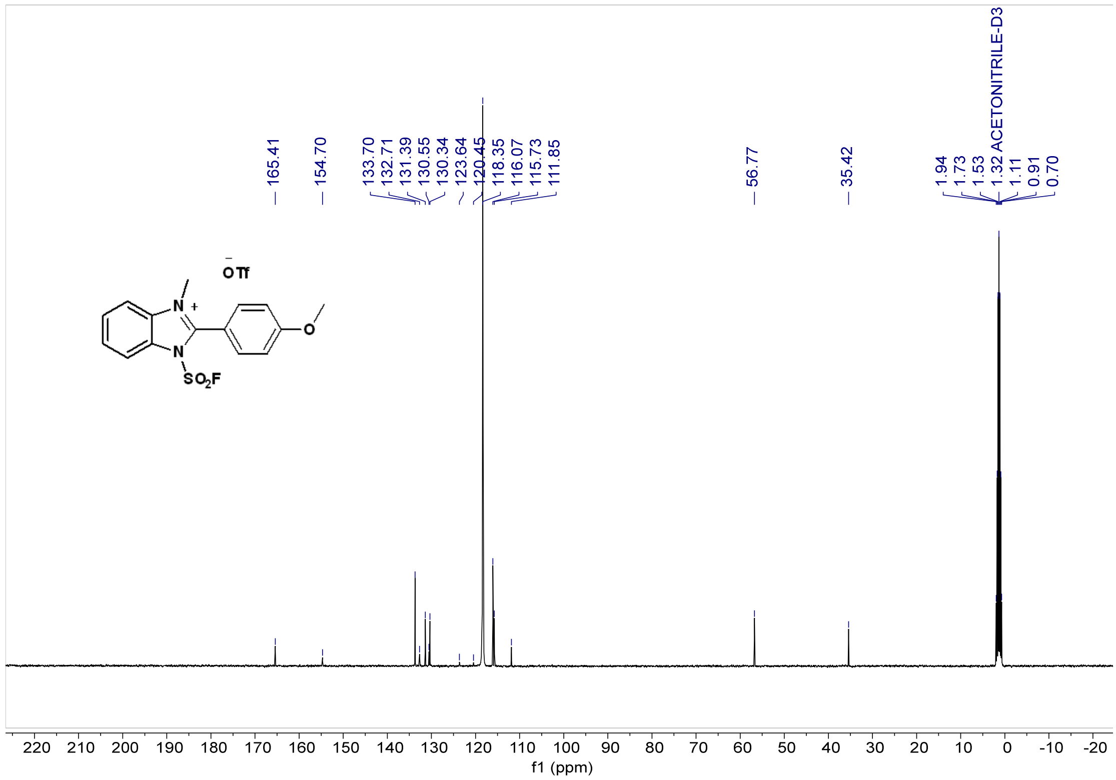

Supplementary Figure 25. ^{13}C NMR (101 MHz, room temperature, CD_3CN) spectra of product **2e**

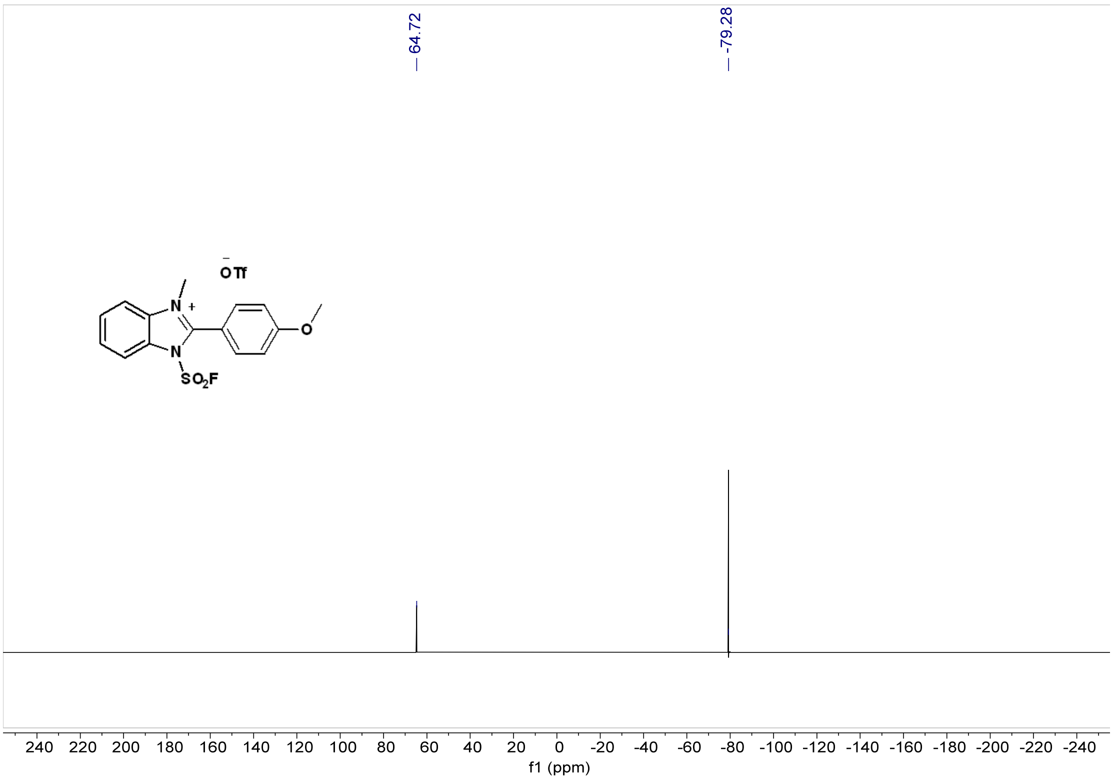

Supplementary Figure 26. ^{19}F NMR (376 MHz, room temperature, CD_3CN) spectra of product **2e**

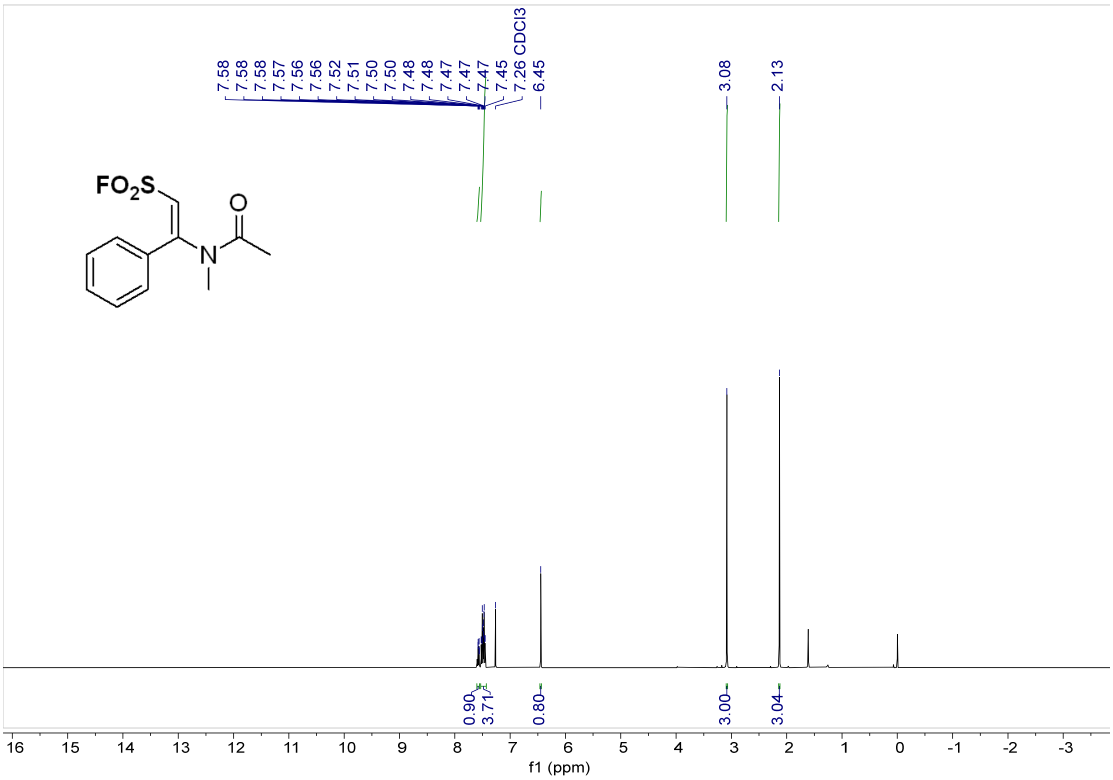

Supplementary Figure 27. ^{1}H NMR (400 MHz, room temperature, $CDCl_3$) spectra of product **3a**

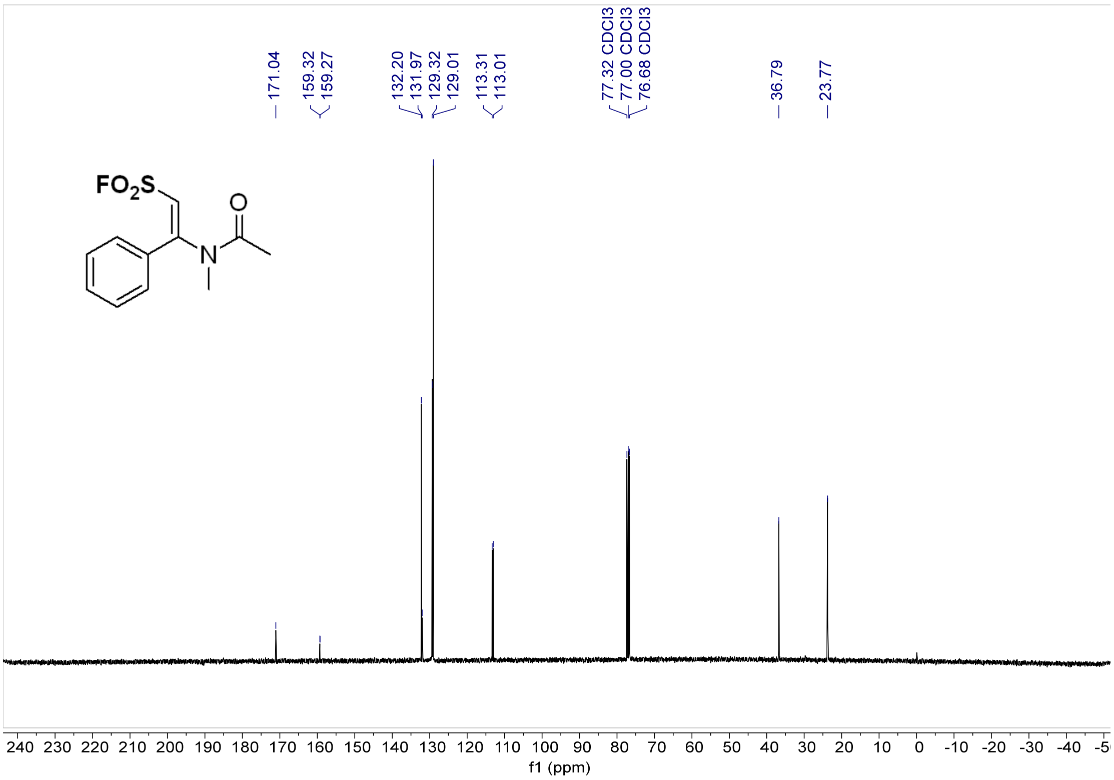

Supplementary Figure 28. ^{13}C NMR (101 MHz, room temperature, $CDCl_3$) spectra of product **3a**

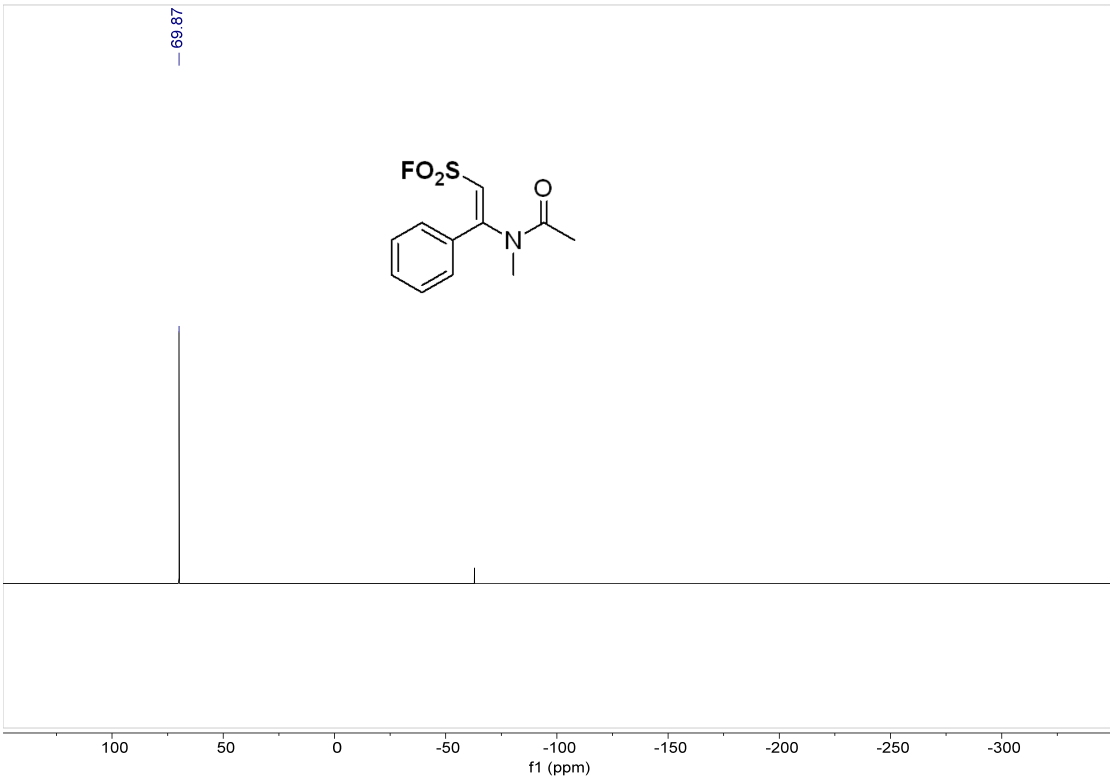

Supplementary Figure 29. ^{19}F NMR (101 MHz, room temperature, $CDCl_3$) spectra of product **3a**

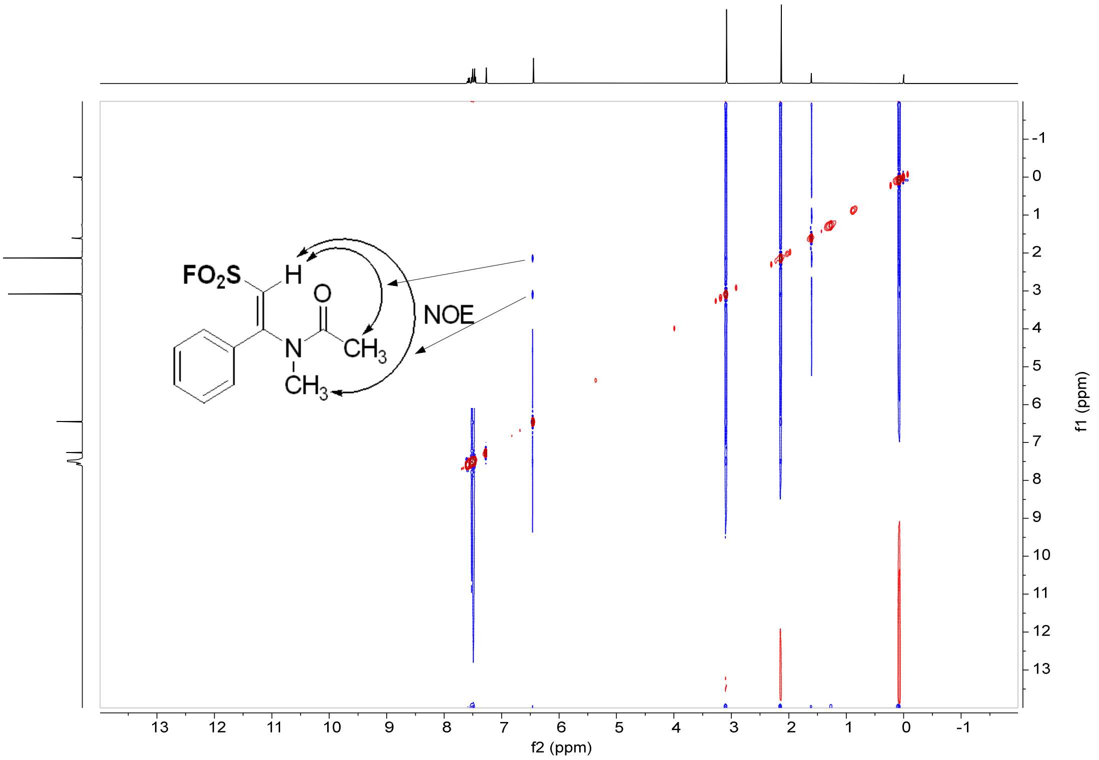

Supplementary Figure 30. NOESY (400 MHz, room temperature, $CDCl_3$) spectra of product **3a**

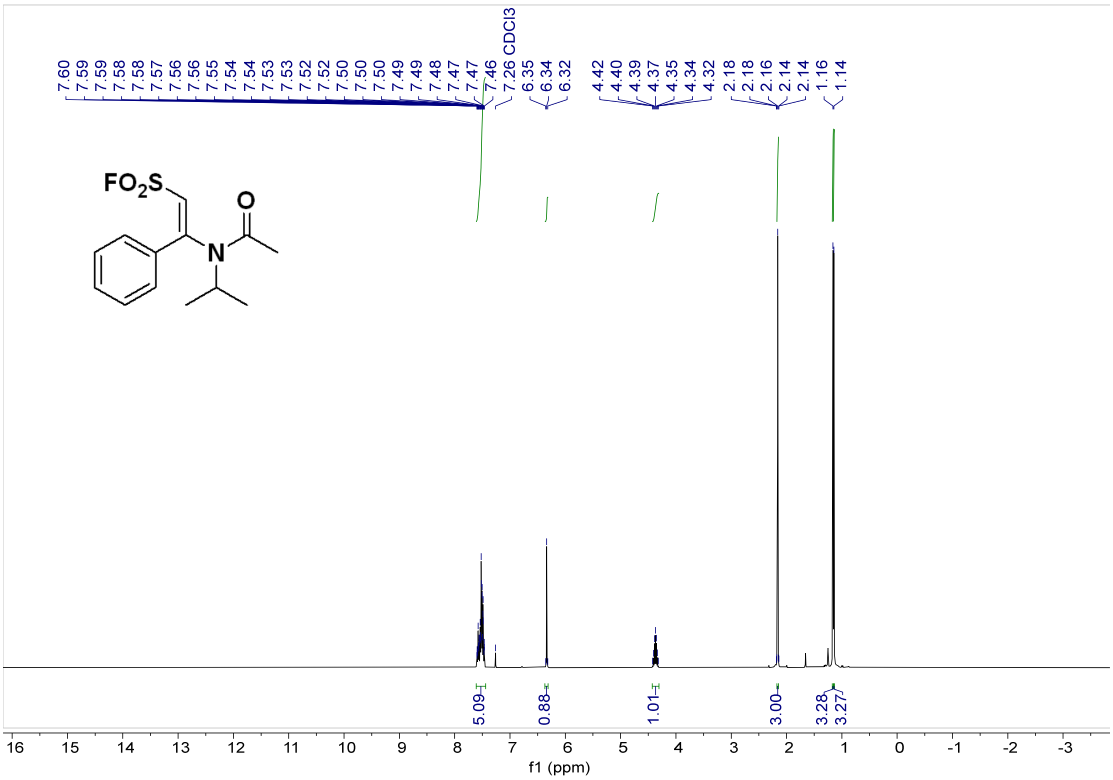

Supplementary Figure 31. ^{1}H NMR (400 MHz, room temperature, $CDCl_3$) spectra of product **3b**

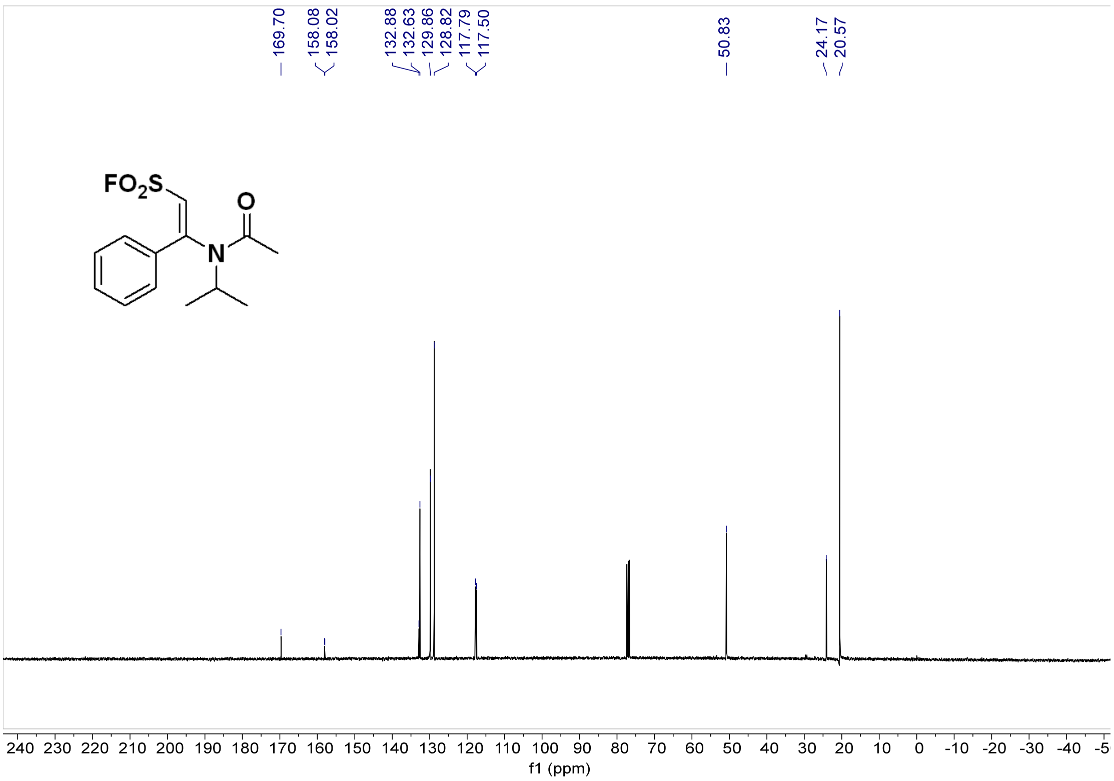

Supplementary Figure 32. ^{13}C NMR (101 MHz, room temperature, $CDCl_3$) spectra of product **3b**

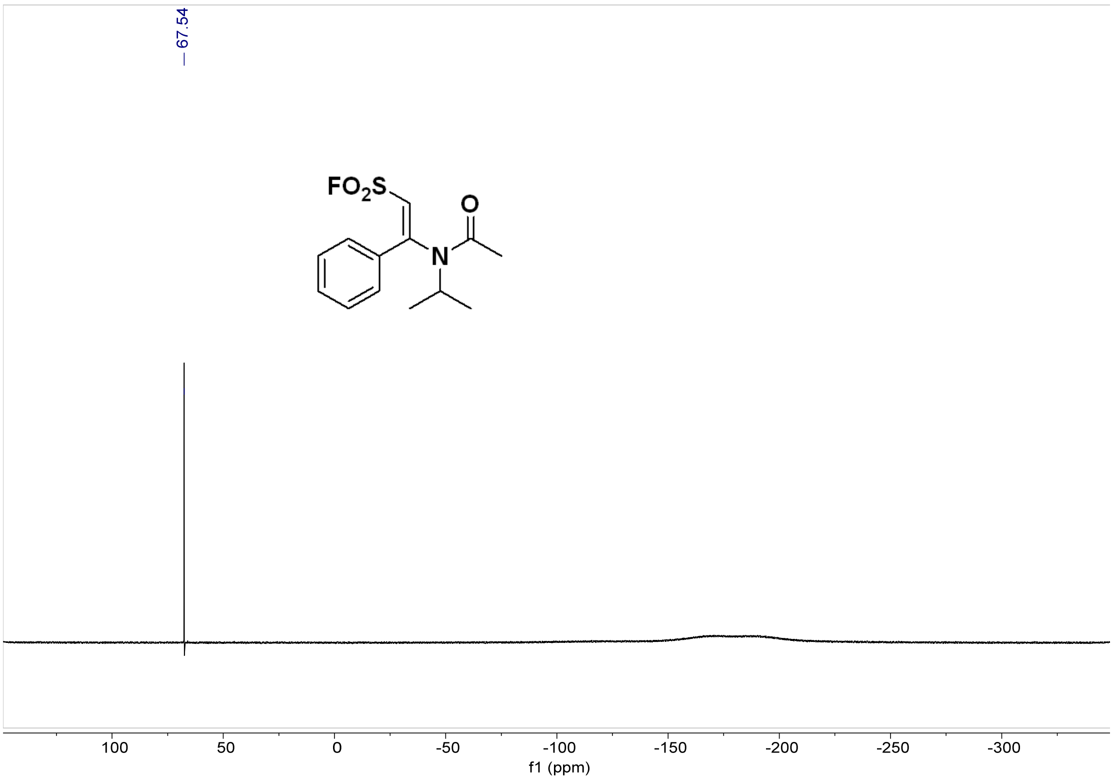

Supplementary Figure 33. ^{19}F NMR (376 MHz, room temperature, $CDCl_3$) spectra of product **3b**

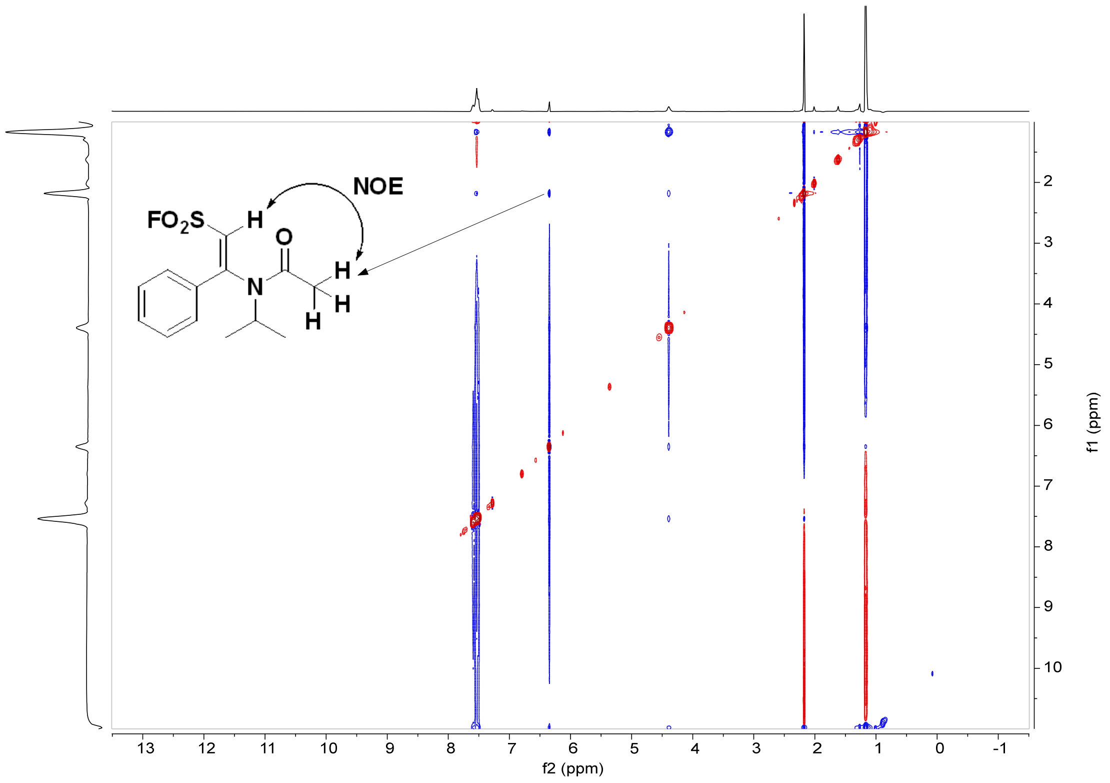

Supplementary Figure 34. NOESY (400 MHz, room temperature, CDCl3) spectra of product **3b**

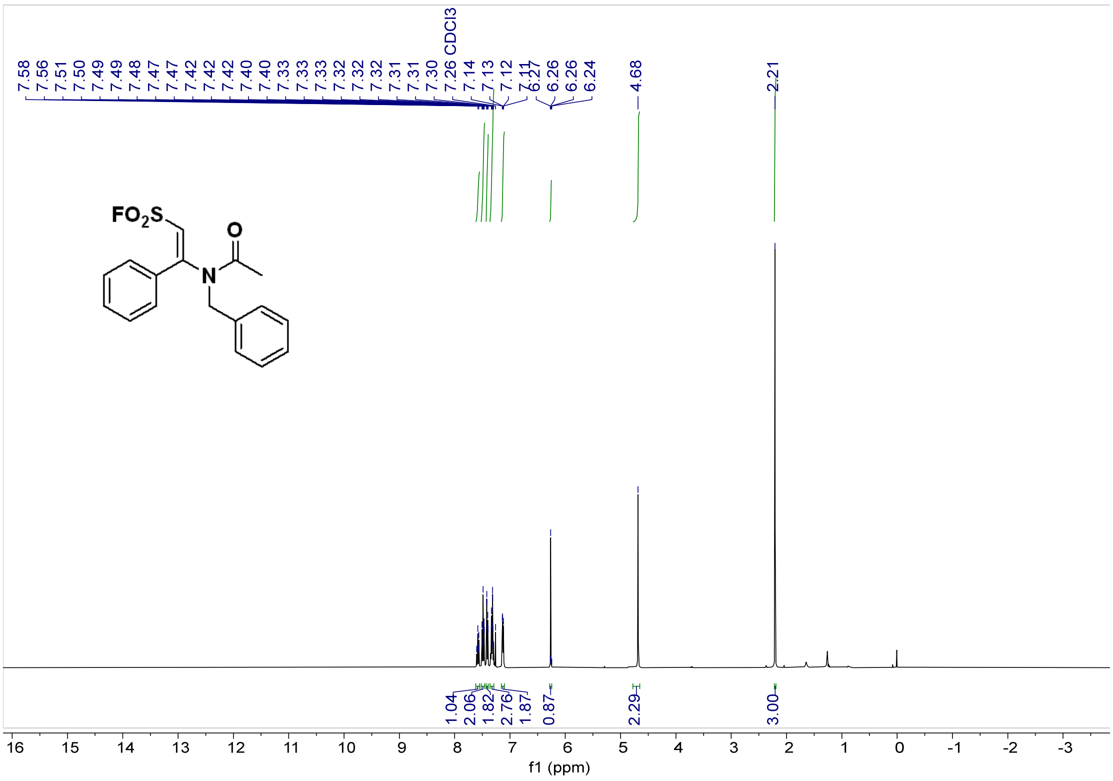

Supplementary Figure 35. ^{1}H NMR (400 MHz, room temperature, CDCl3) spectra of product **3c**

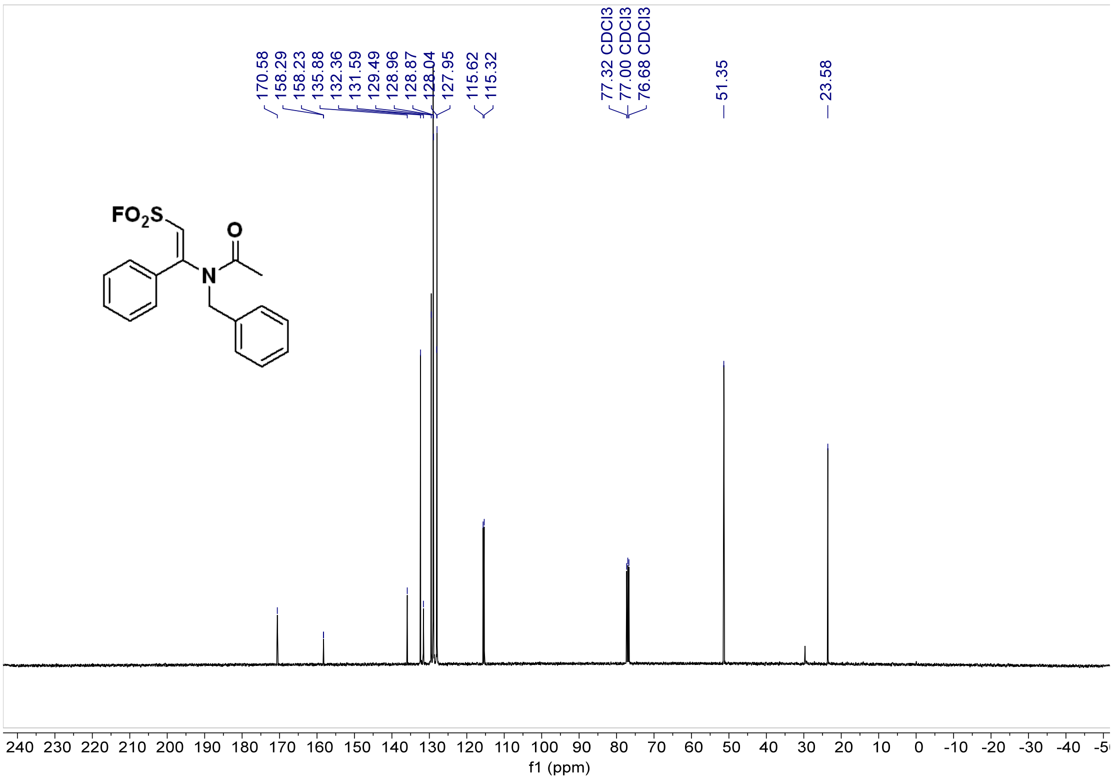

Supplementary Figure 36. ^{13}C NMR (101 MHz, room temperature, $CDCl_3$) spectra of product **3c**

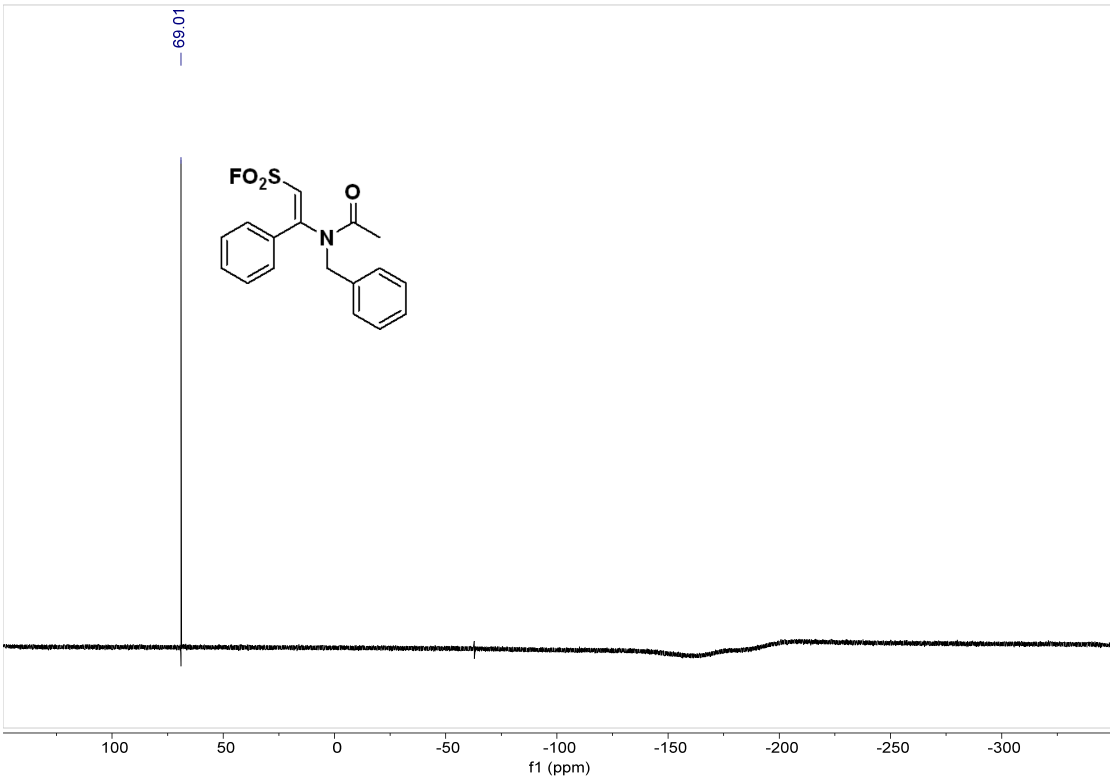

Supplementary Figure 37. ^{19}F NMR (376 MHz, room temperature, $CDCl_3$) spectra of product **3c**

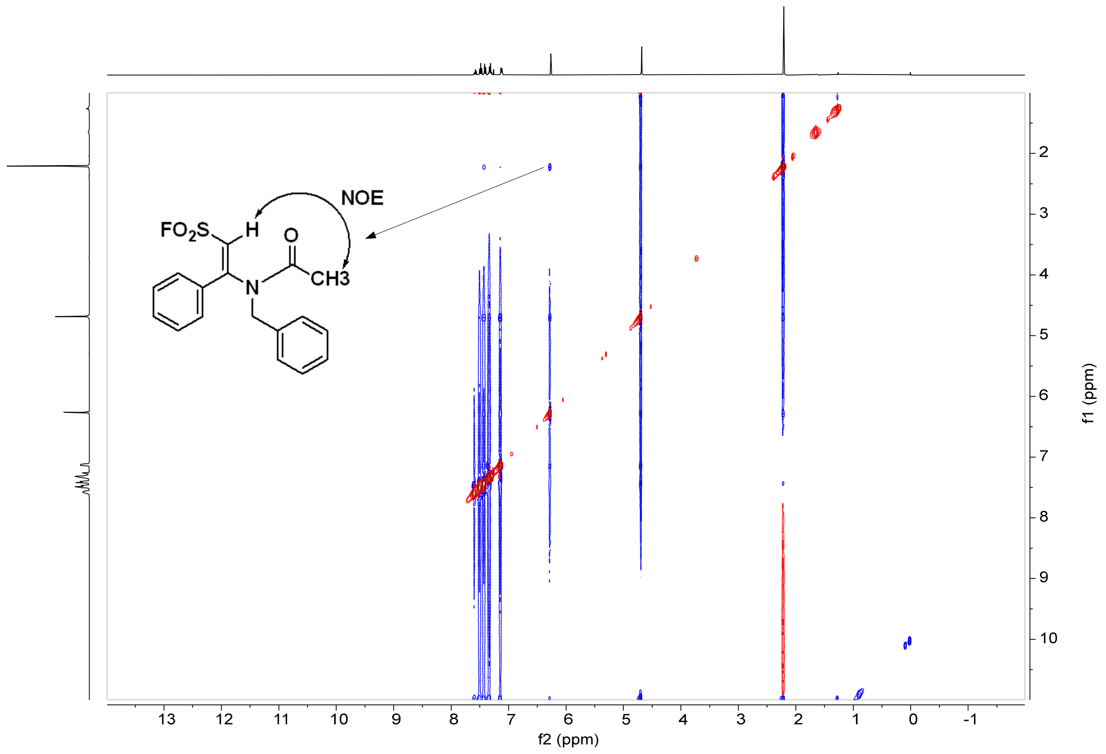

Supplementary Figure 38. NOESY (400 MHz, room temperature, $CDCl_3$) spectra of product **3c**

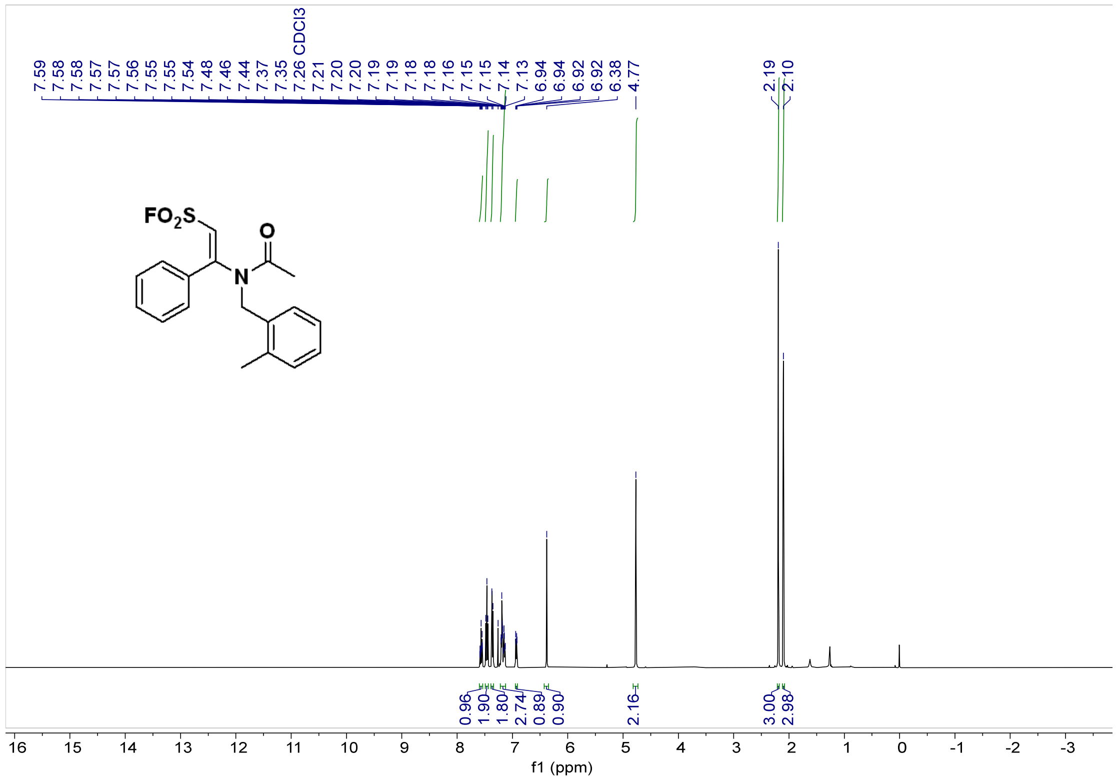

Supplementary Figure 39. ^{1}H NMR (400 MHz, room temperature, $CDCl_3$) spectra of product **3d**

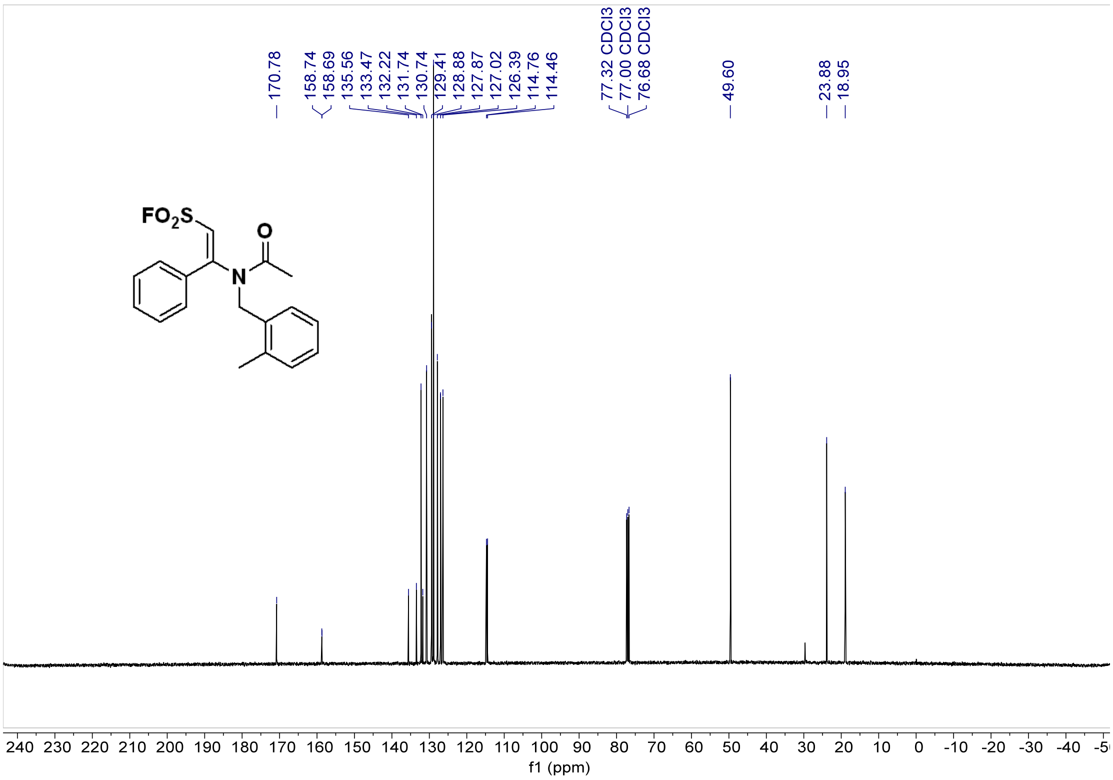

Supplementary Figure 40. ^{13}C NMR (101 MHz, room temperature, $CDCl_3$) spectra of product **3d**

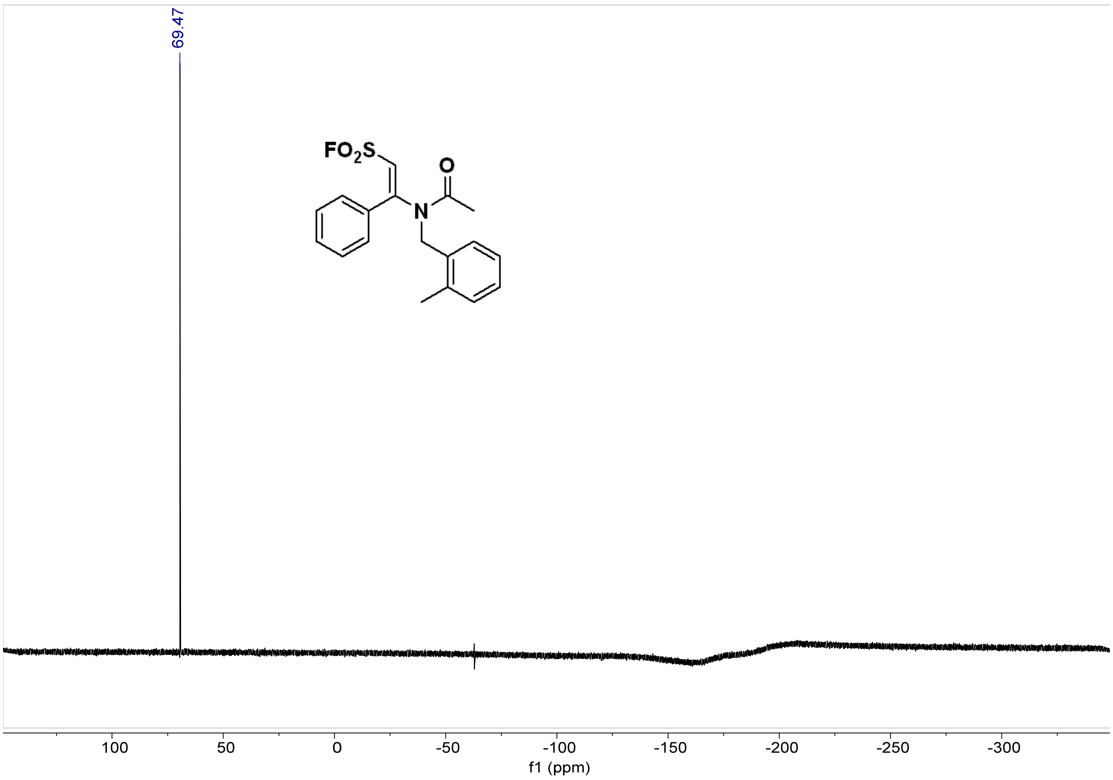

Supplementary Figure 41. ^{19}F NMR (376 MHz, room temperature, $CDCl_3$) spectra of product **3d**

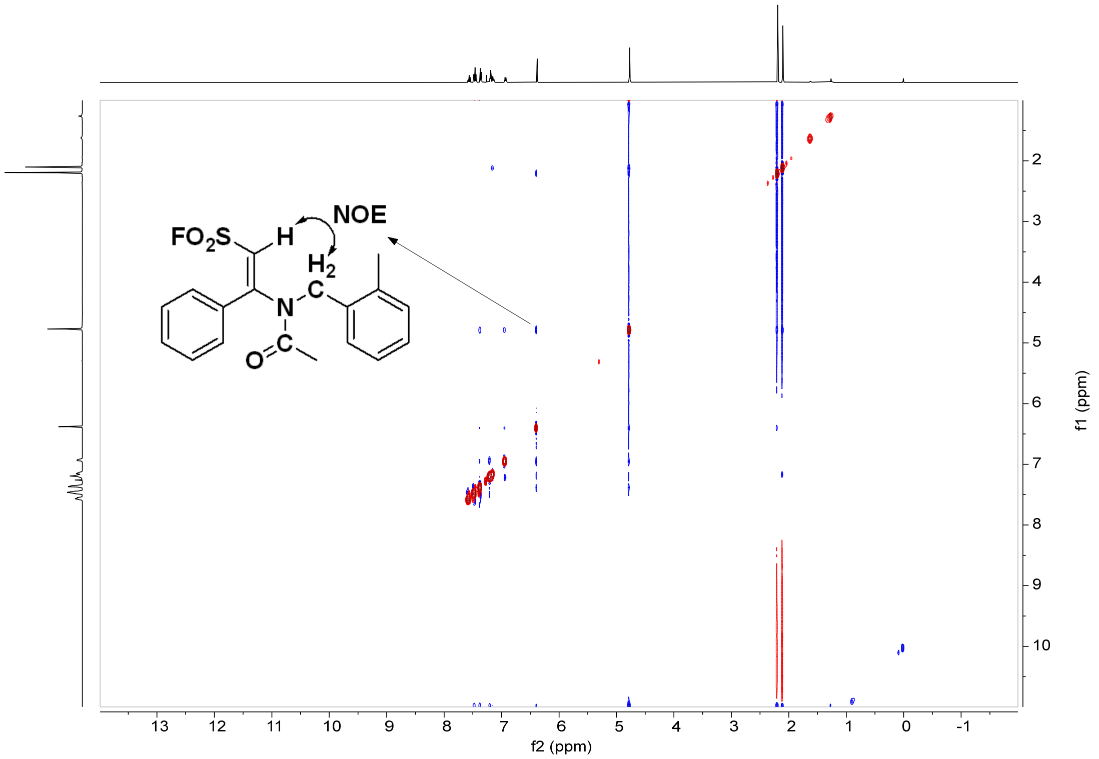

Supplementary Figure 42. NOESY (400 MHz, room temperature, $CDCl_3$) spectra of product **3d**

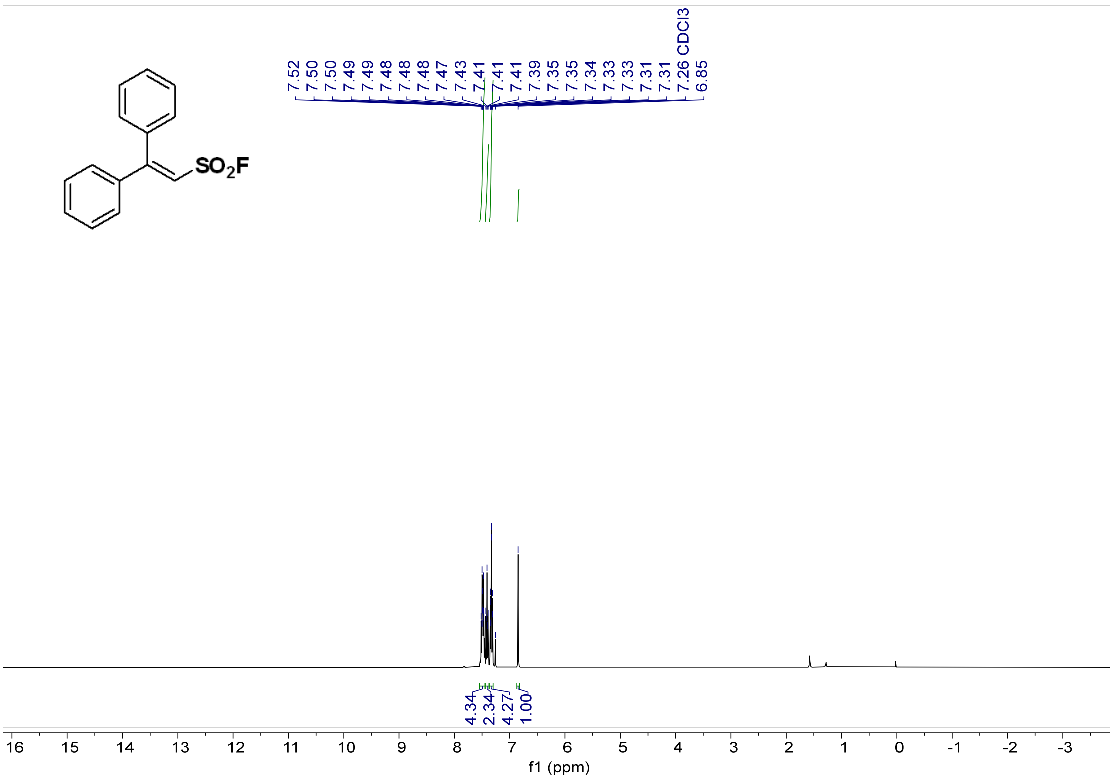

Supplementary Figure 43. ^{1}H NMR (400 MHz, room temperature, $CDCl_3$) spectra of product **3e**

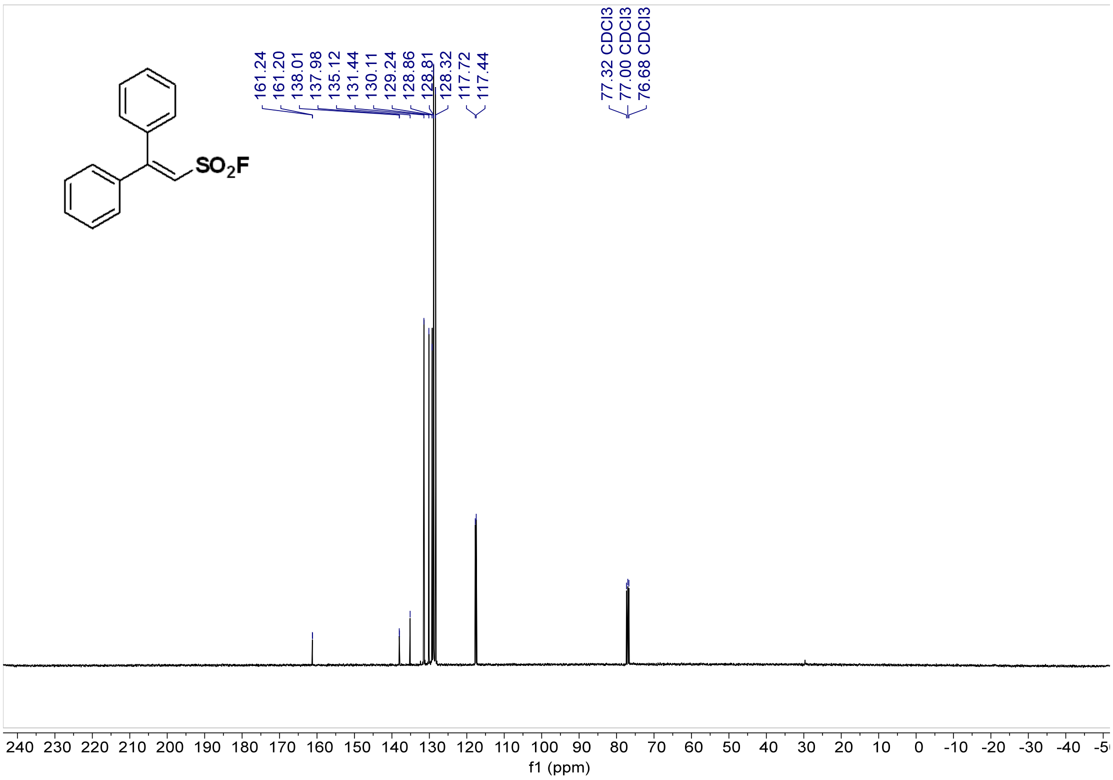

Supplementary Figure 44. ^{13}C NMR (101 MHz, room temperature, $CDCl_3$) spectra of product **3e**

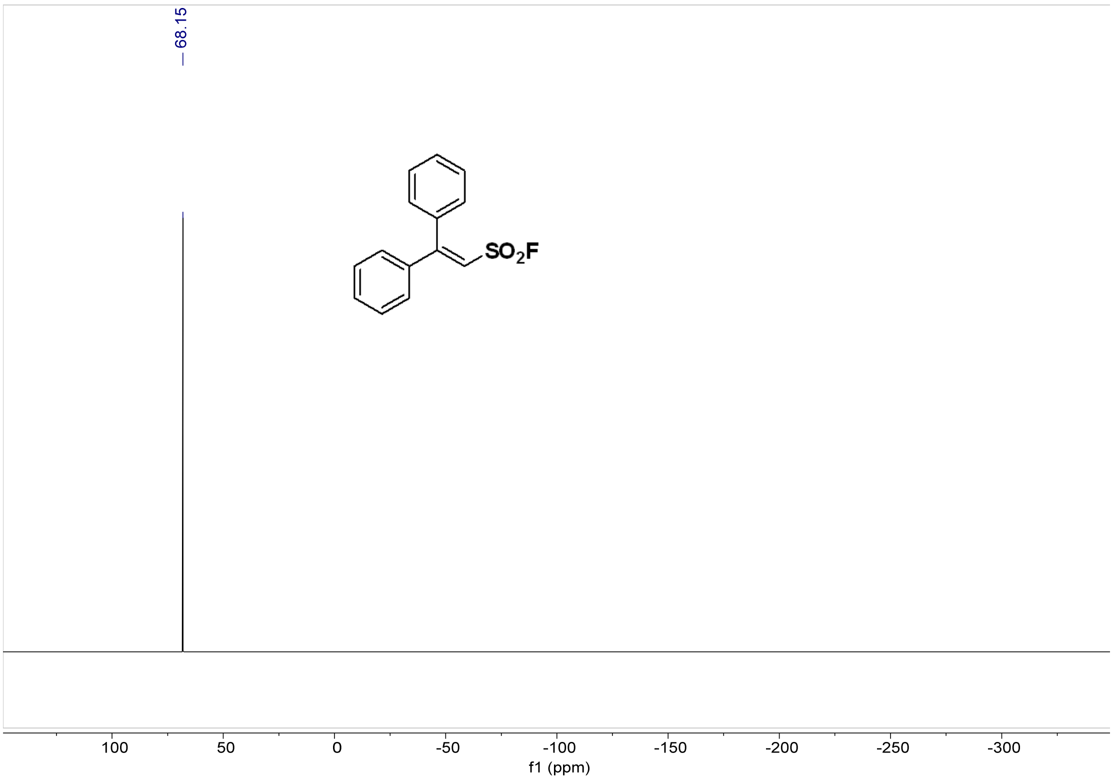

Supplementary Figure 45. ^{19}F NMR (376 MHz, room temperature, $CDCl_3$) spectra of product **3e**

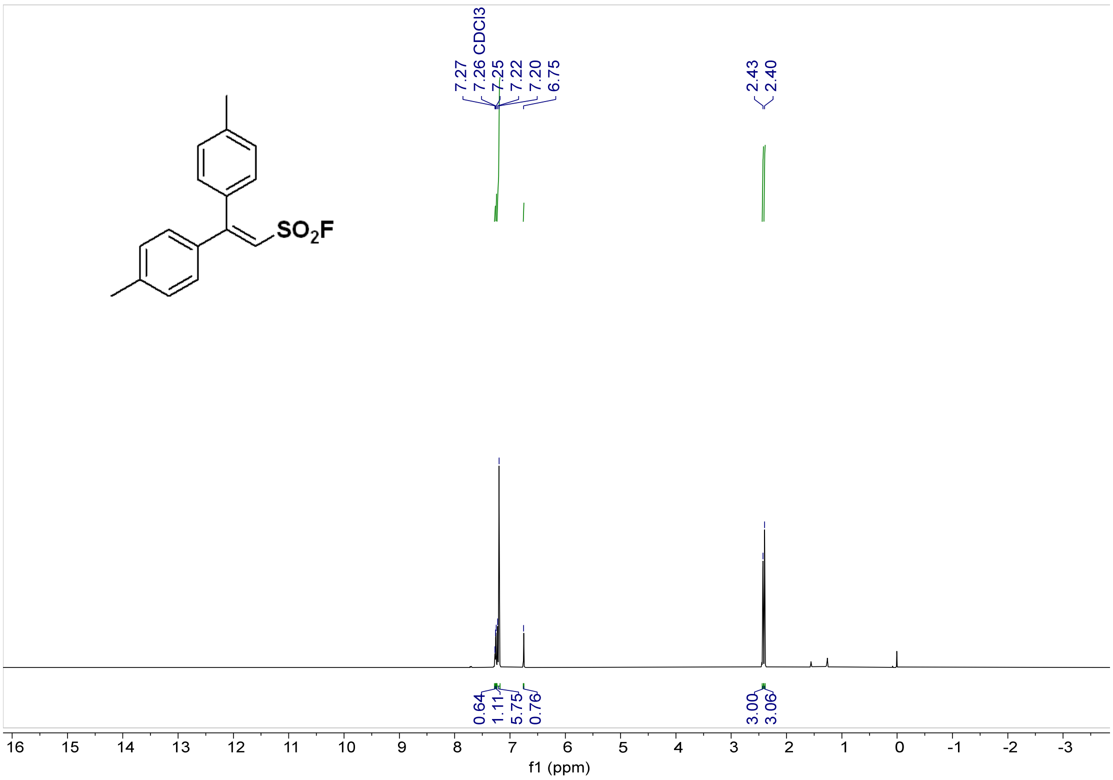

Supplementary Figure 46. ^{1}H NMR (400 MHz, room temperature, $CDCl_3$) spectra of product **3f**

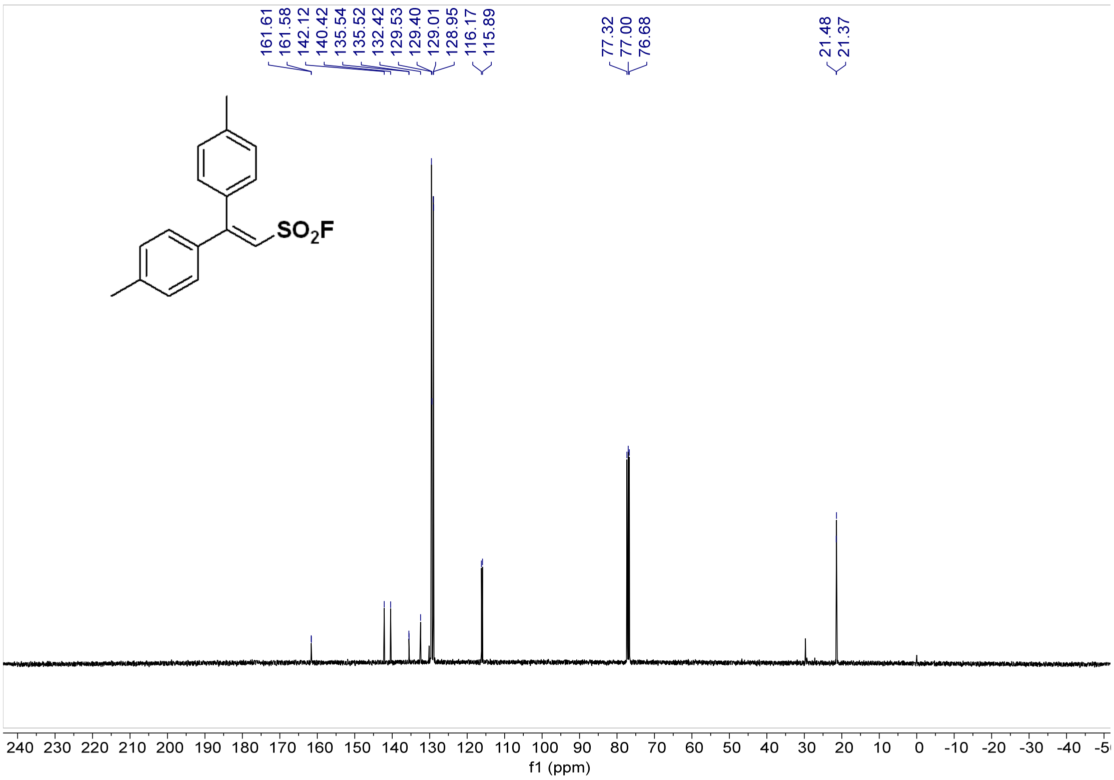

Supplementary Figure 47. ^{13}C NMR (101 MHz, room temperature, $CDCl_3$) spectra of product **3f**

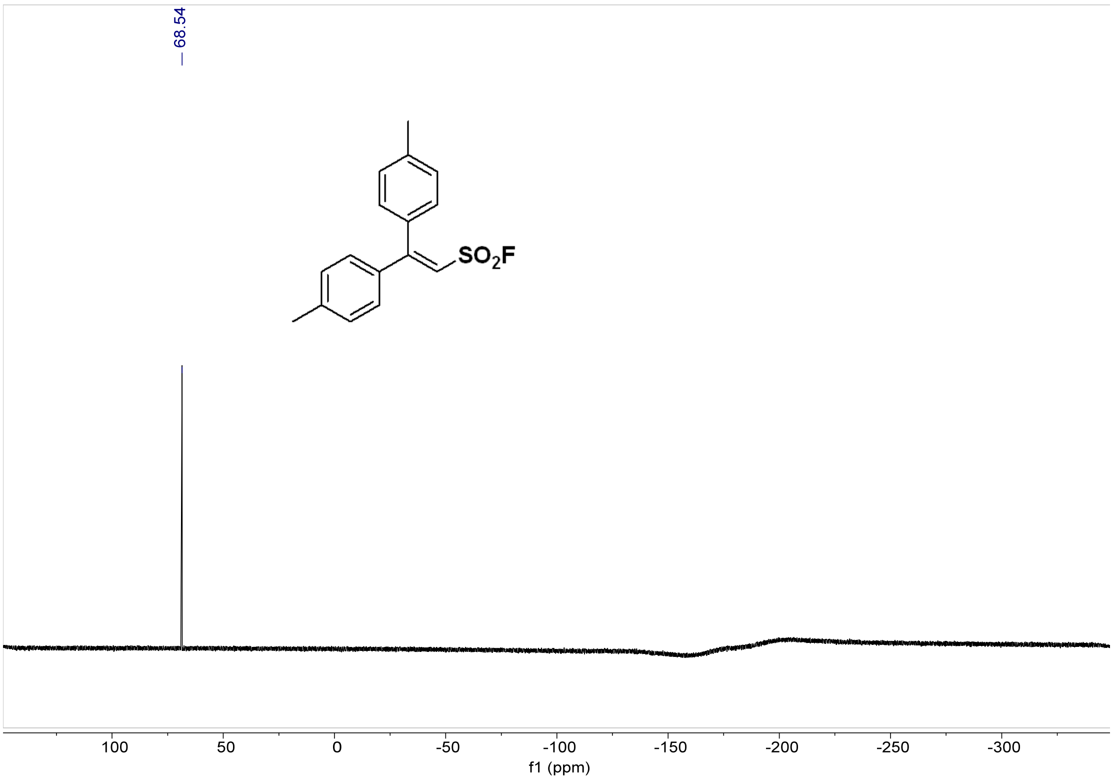

Supplementary Figure 48. ^{19}F NMR (376 MHz, room temperature, $CDCl_3$) spectra of product **3f**

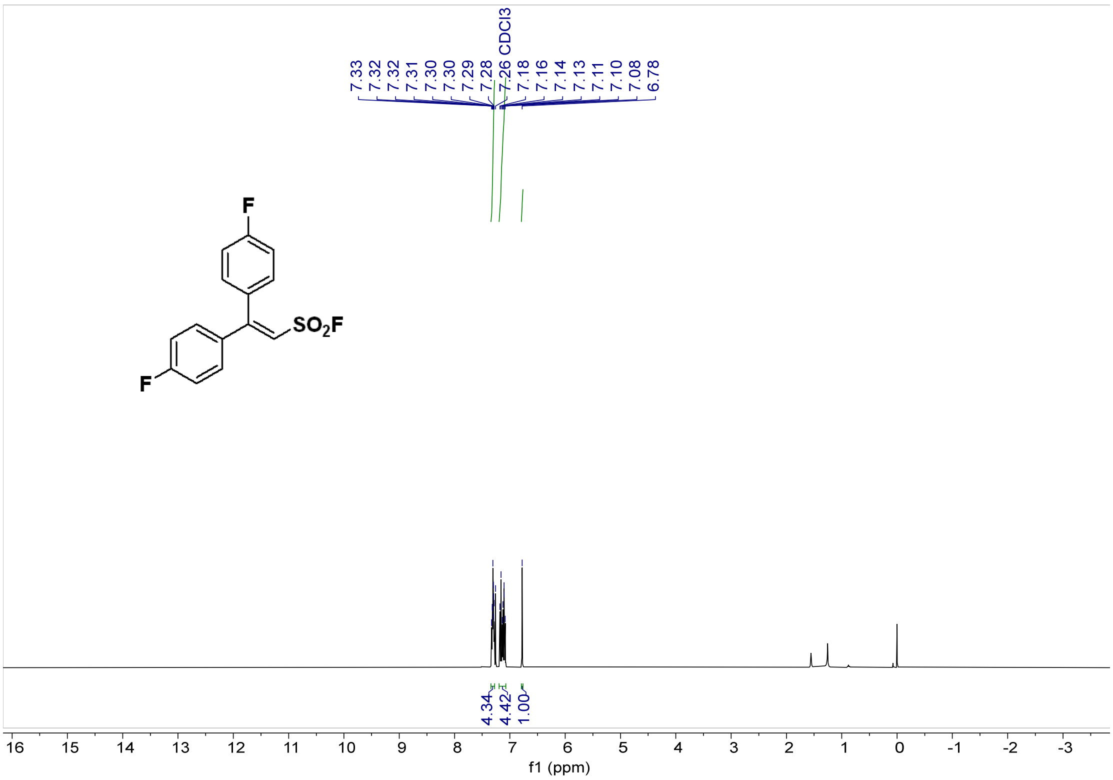

Supplementary Figure 49. ^{1}H NMR (400 MHz, room temperature, $CDCl_3$) spectra of product **3g**

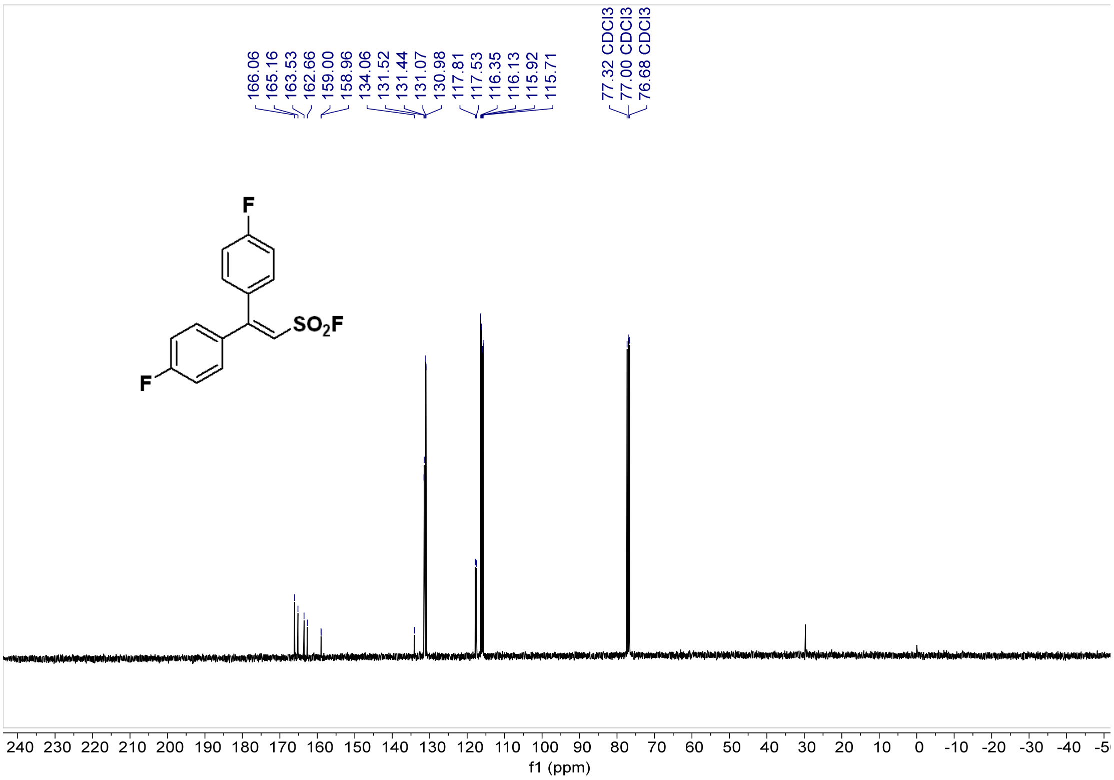

Supplementary Figure 50. ^{13}C NMR (101 MHz, room temperature, $CDCl_3$) spectra of product **3g**

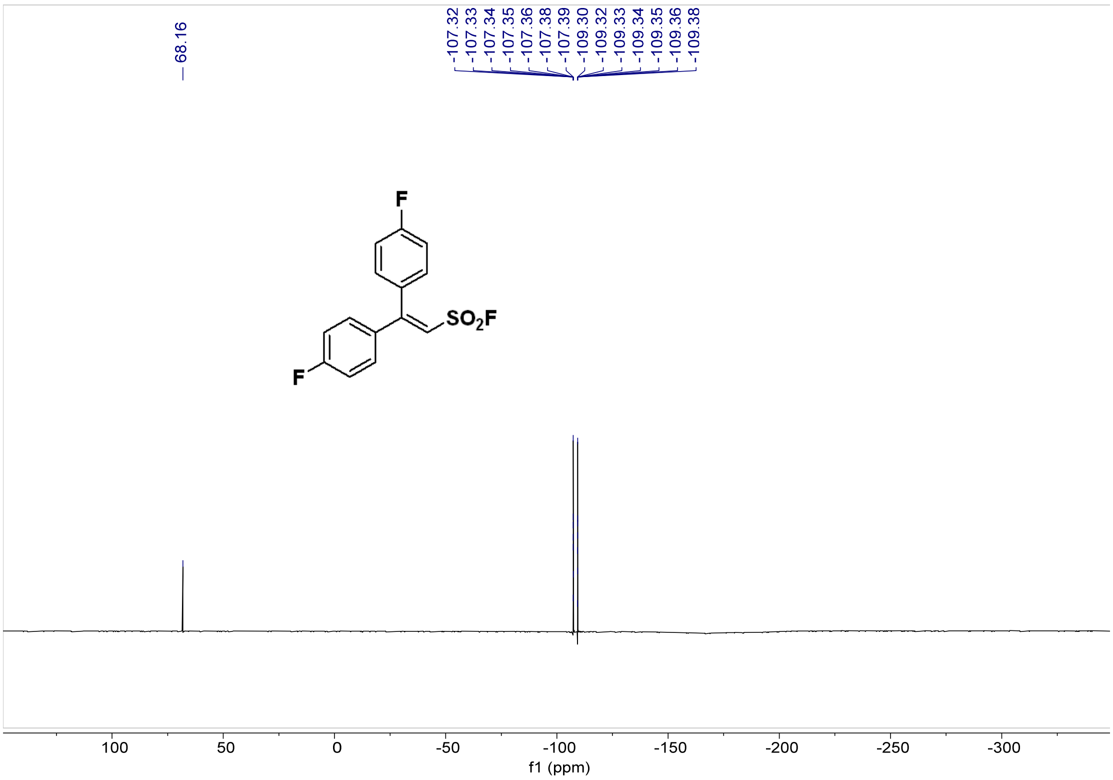

Supplementary Figure 51. ^{19}F NMR (376 MHz, room temperature, $CDCl_3$) spectra of product **3g**

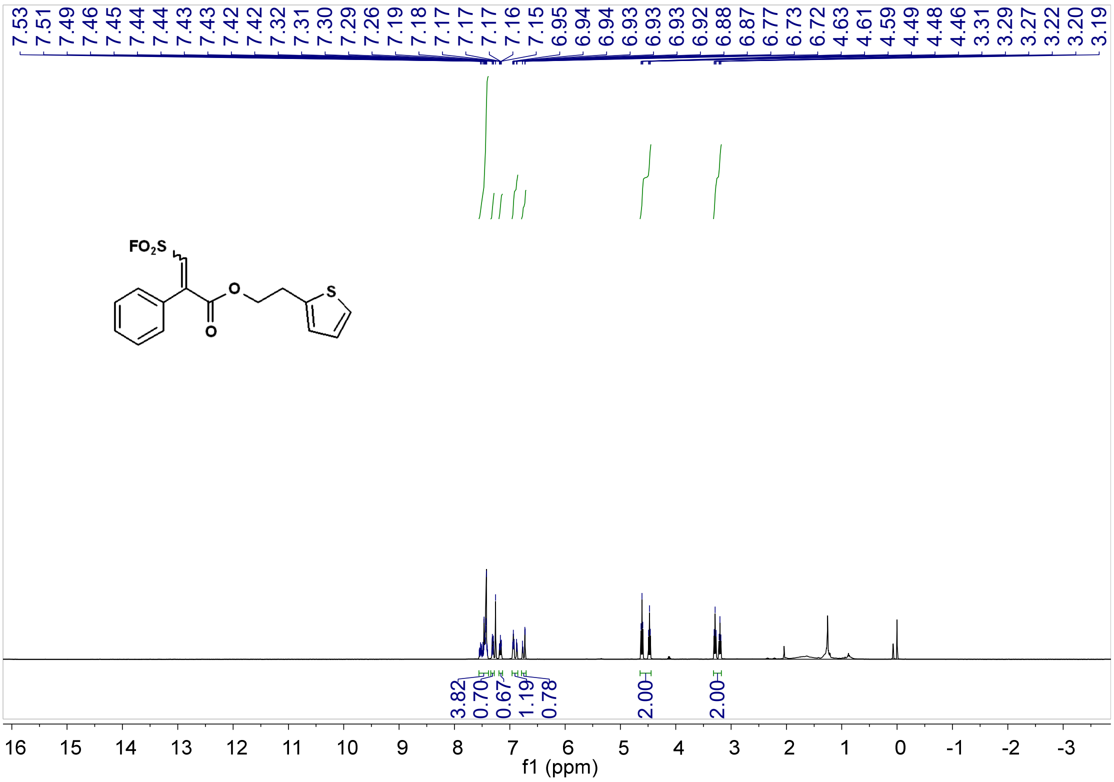

Supplementary Figure 52. ^{1}H NMR (400 MHz, room temperature, $CDCl_3$) spectra of product **3h**

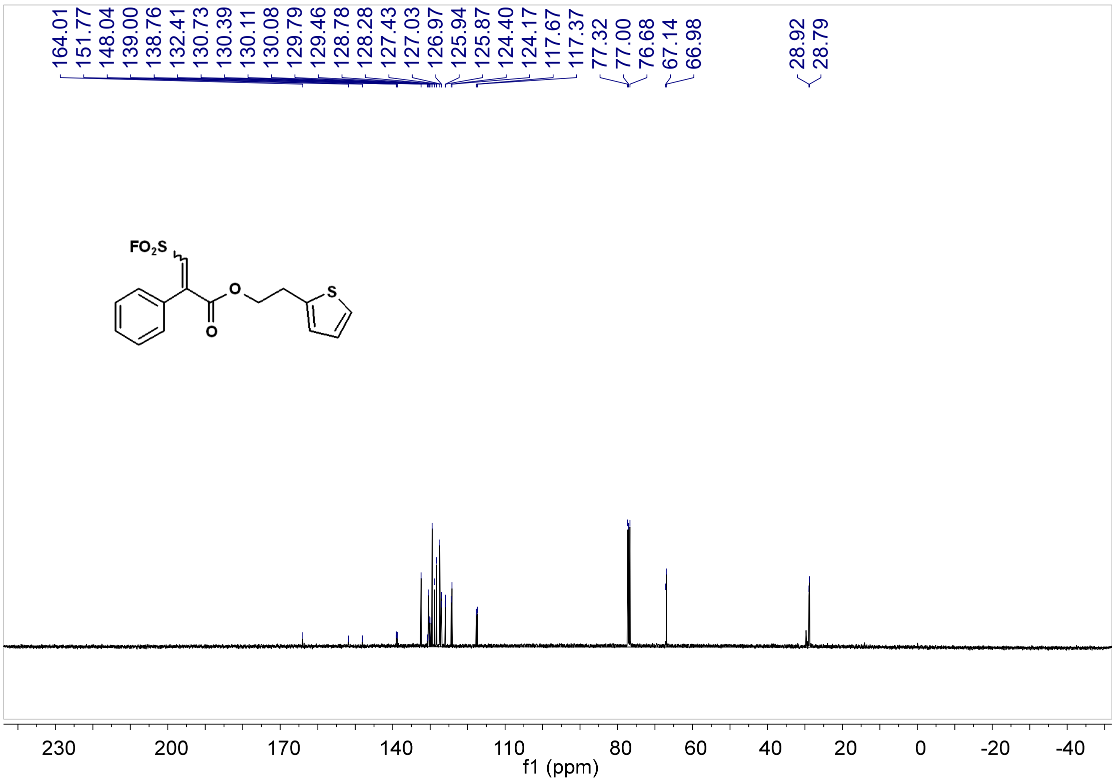

Supplementary Figure 53. ^{13}C NMR (101 MHz, room temperature, $CDCl_3$) spectra of product **3h**

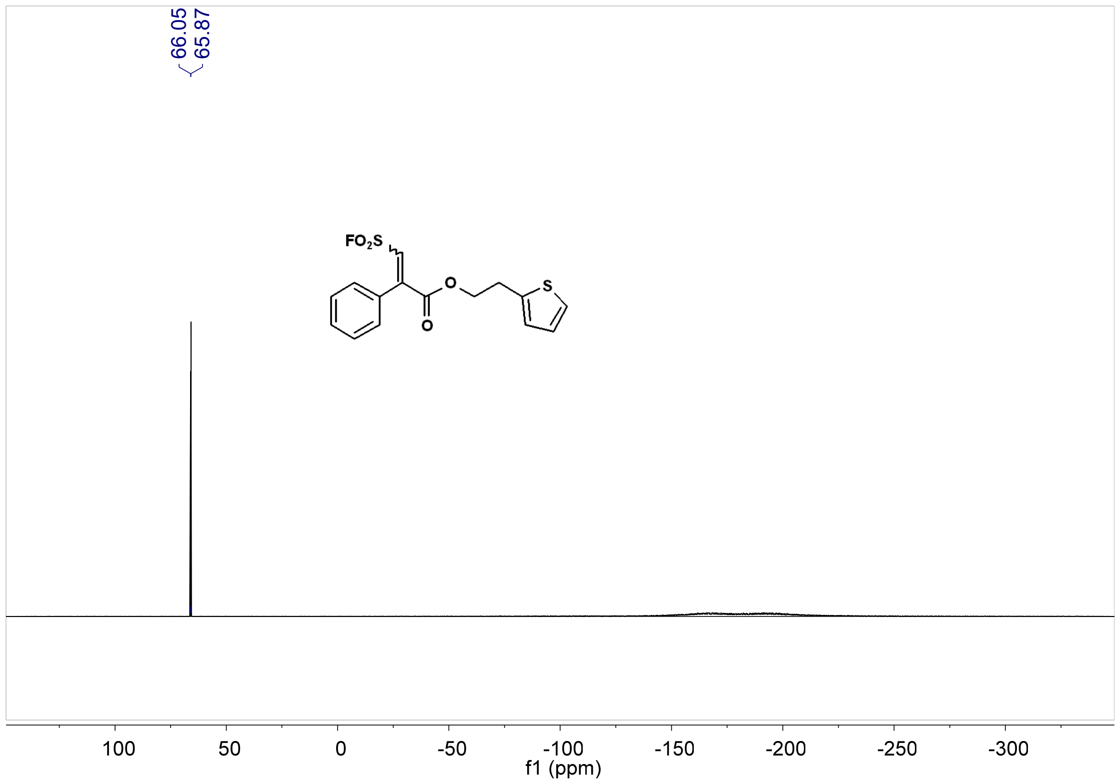

Supplementary Figure 54. ^{19}F NMR (376 MHz, room temperature, $CDCl_3$) spectra of product **3h**

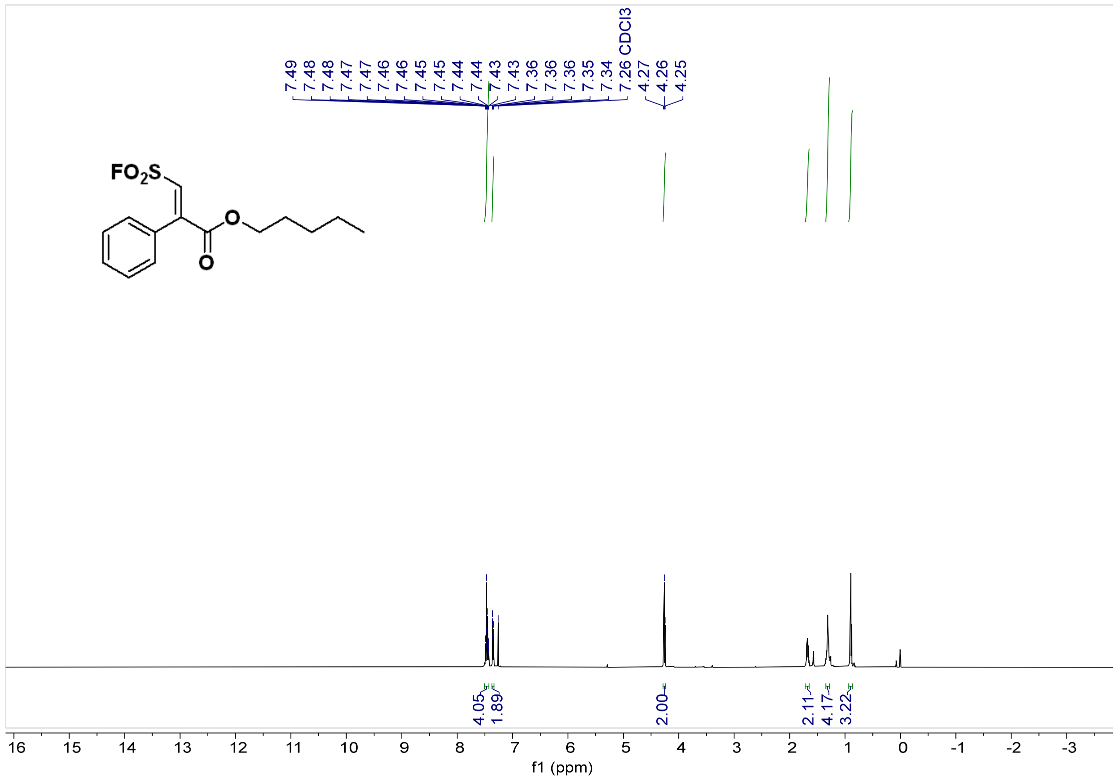

Supplementary Figure 55. ^{1}H NMR (500 MHz, room temperature, $CDCl_3$) spectra of product **3i**

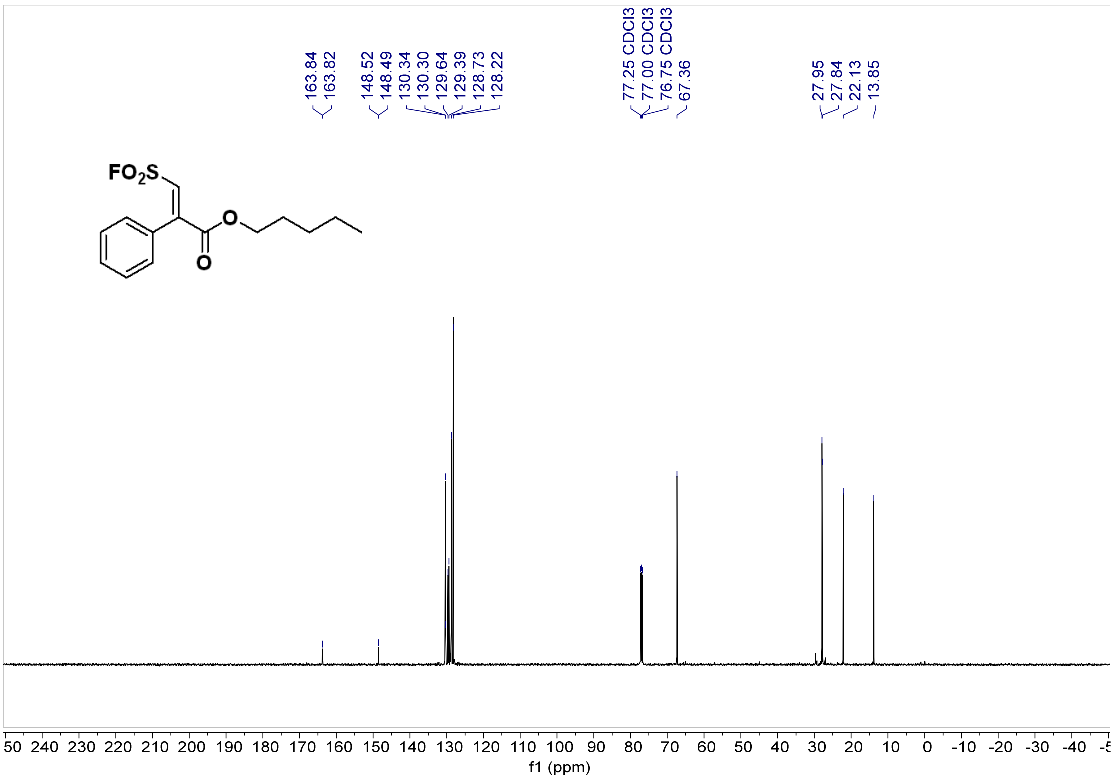

Supplementary Figure 56. ^{13}C NMR (126 MHz, room temperature, $CDCl_3$) spectra of product **3i**

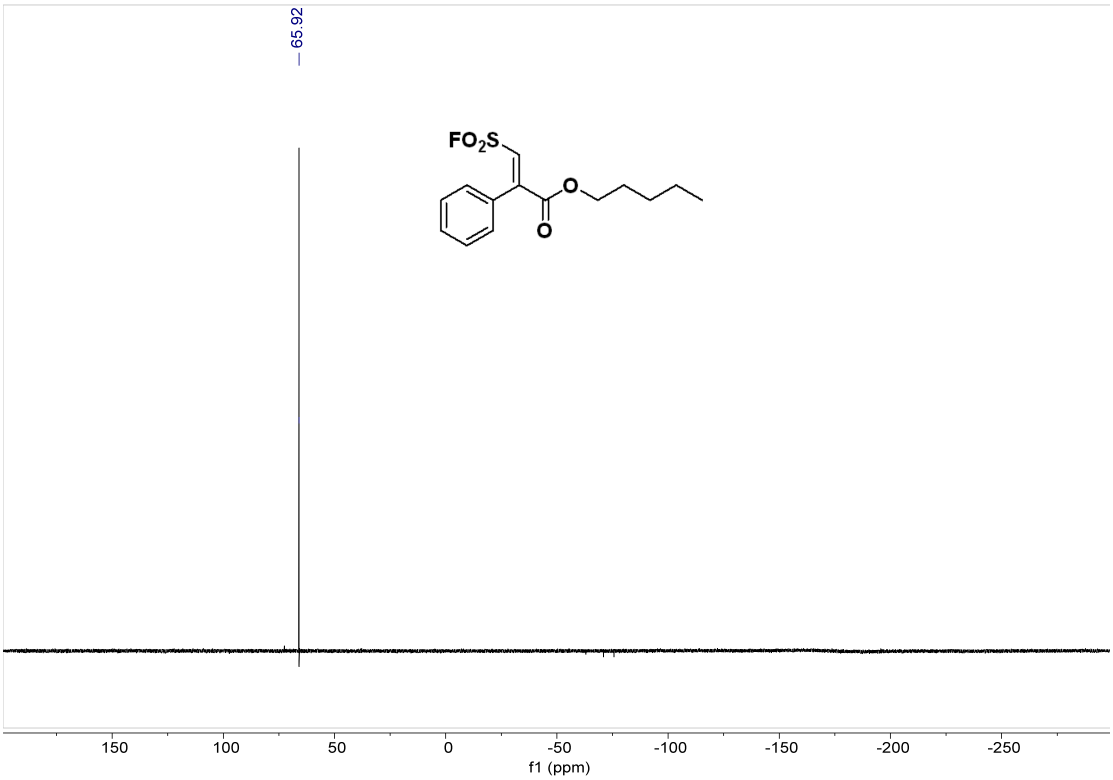

Supplementary Figure 57. ^{19}F NMR (471 MHz, room temperature, $CDCl_3$) spectra of product **3i**

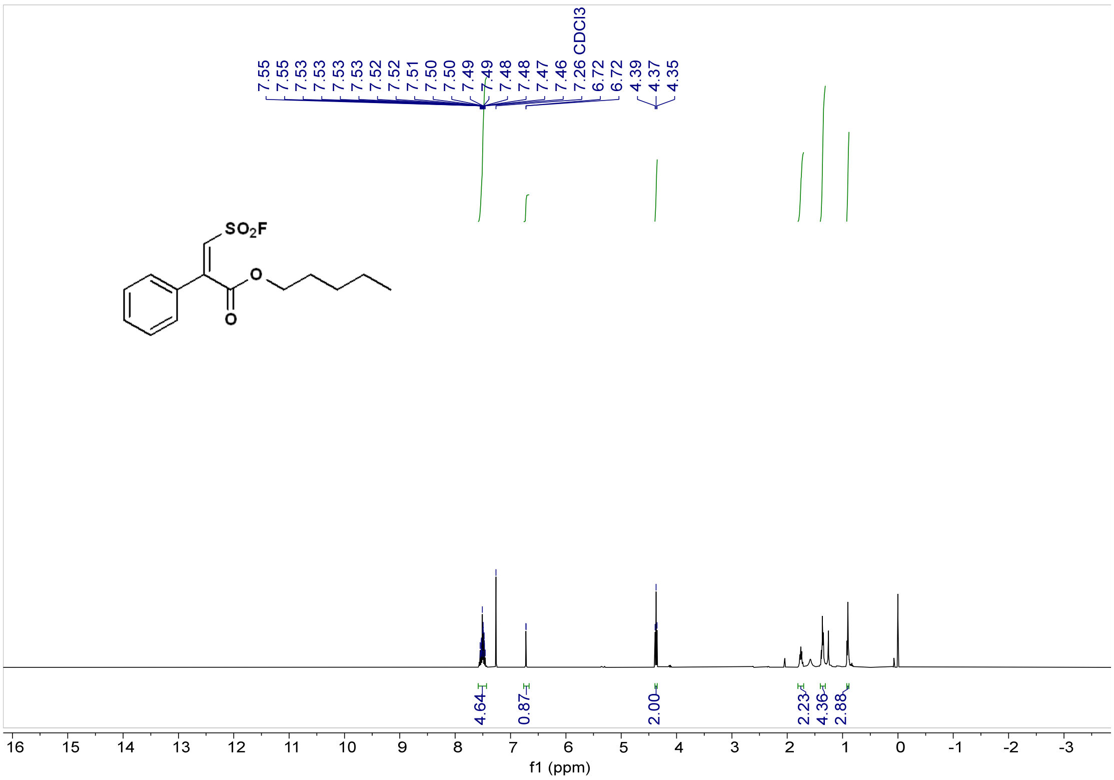

Supplementary Figure 58. ^{1}H NMR (400 MHz, room temperature, $CDCl_3$) spectra of product **3i'**

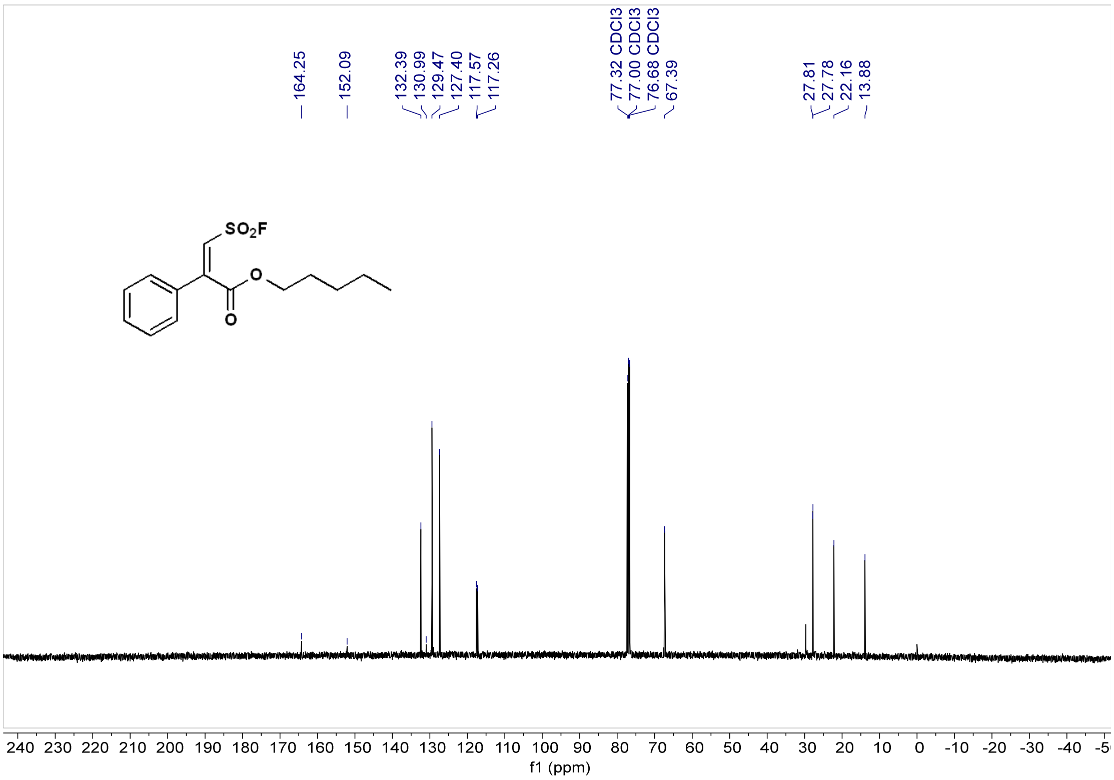

Supplementary Figure 59. ^{13}C NMR (101 MHz, room temperature, $CDCl_3$) spectra of product **3i'**

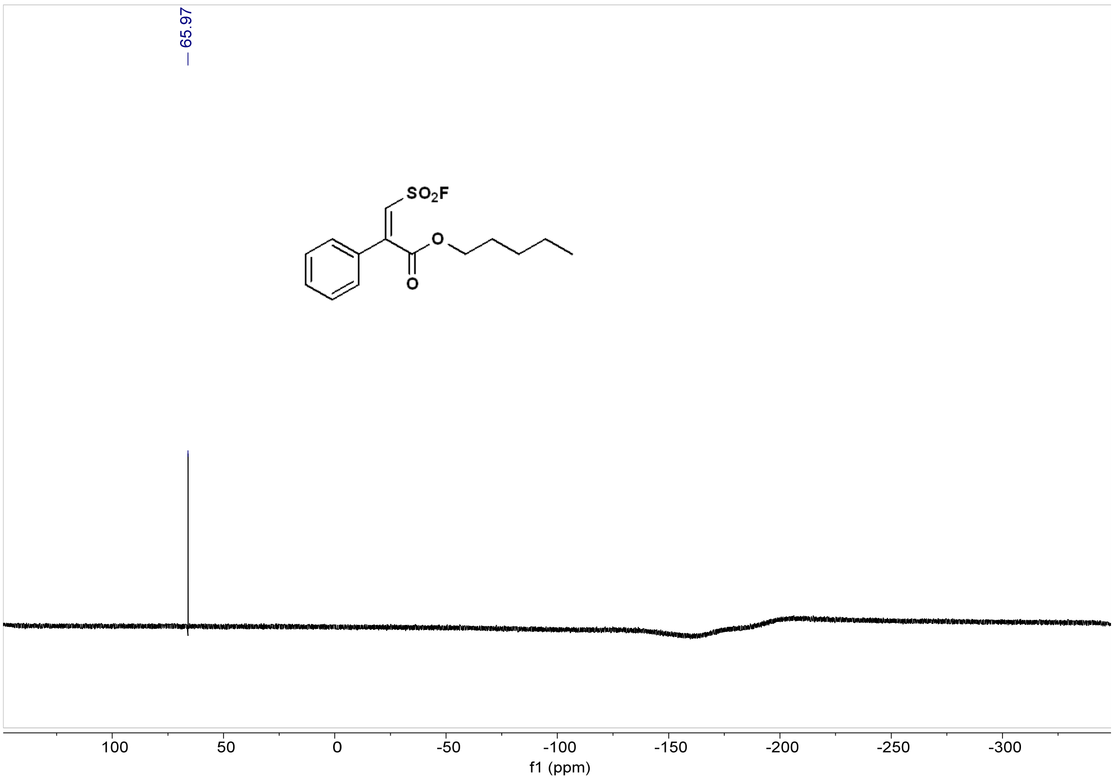

Supplementary Figure 60. ^{19}F NMR (376 MHz, room temperature, $CDCl_3$) spectra of product **3i'**

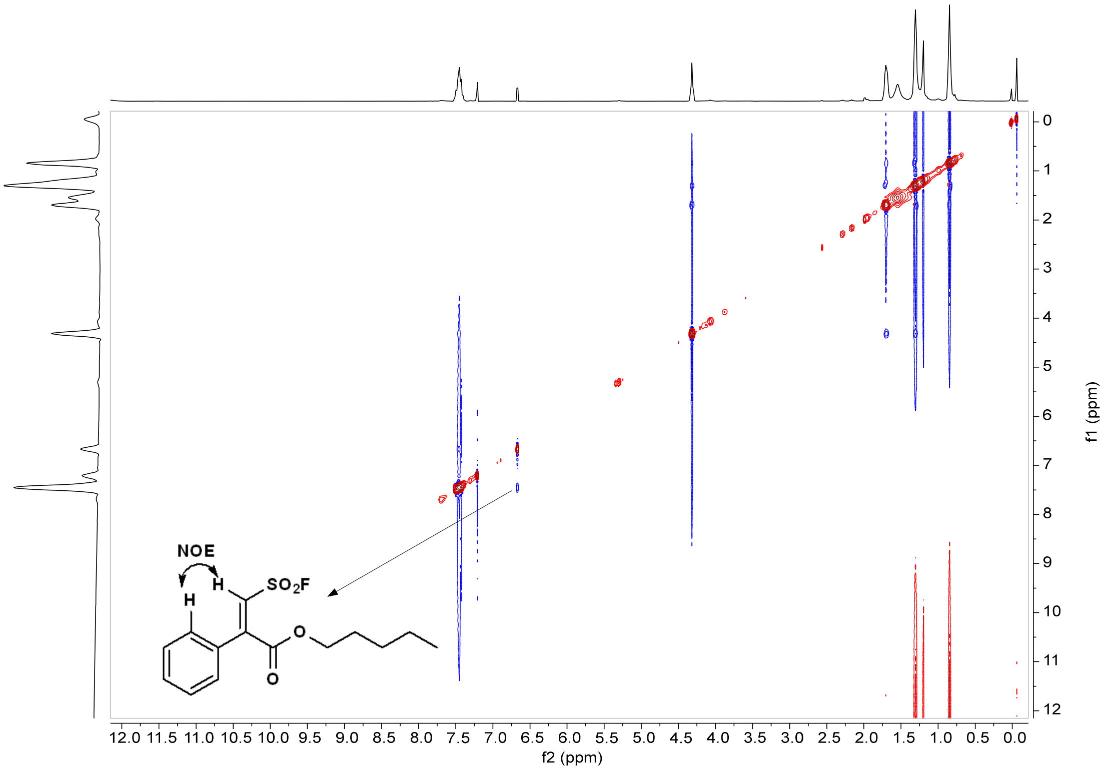

Supplementary Figure 61. NOESY (400 MHz, room temperature, $CDCl_3$) spectra of product **3i'**

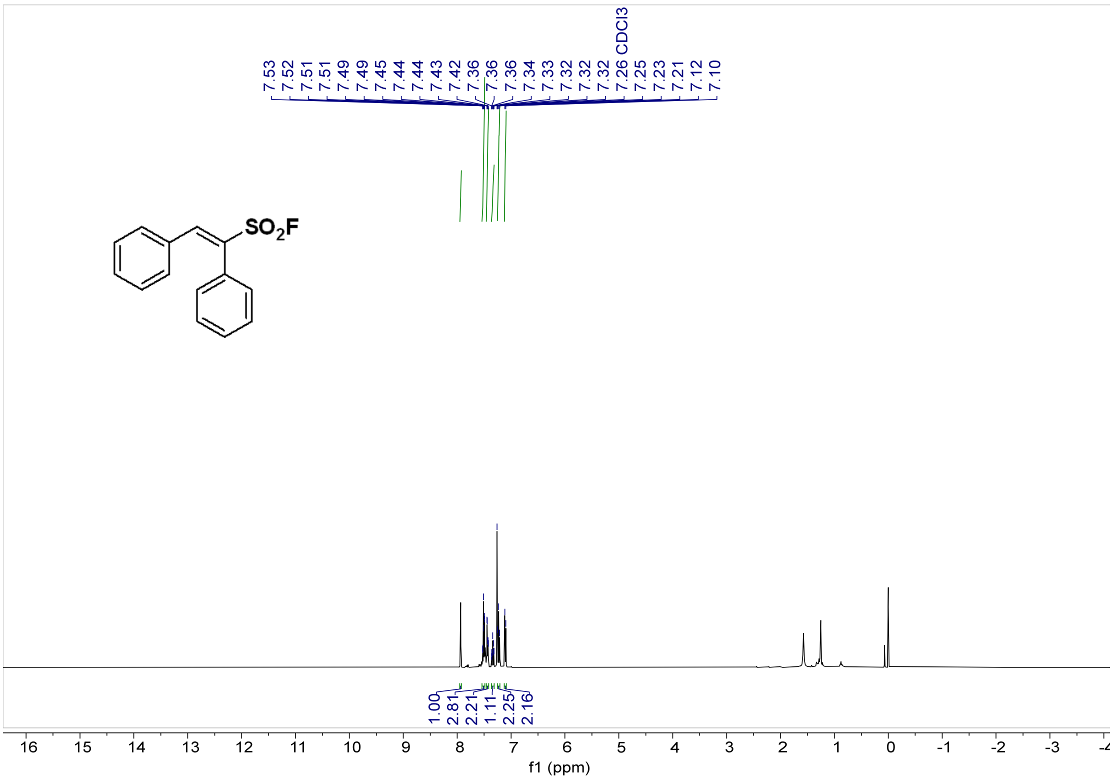

Supplementary Figure 62. ^{1}H NMR (400 MHz, room temperature, $CDCl_3$) spectra of product **3j**

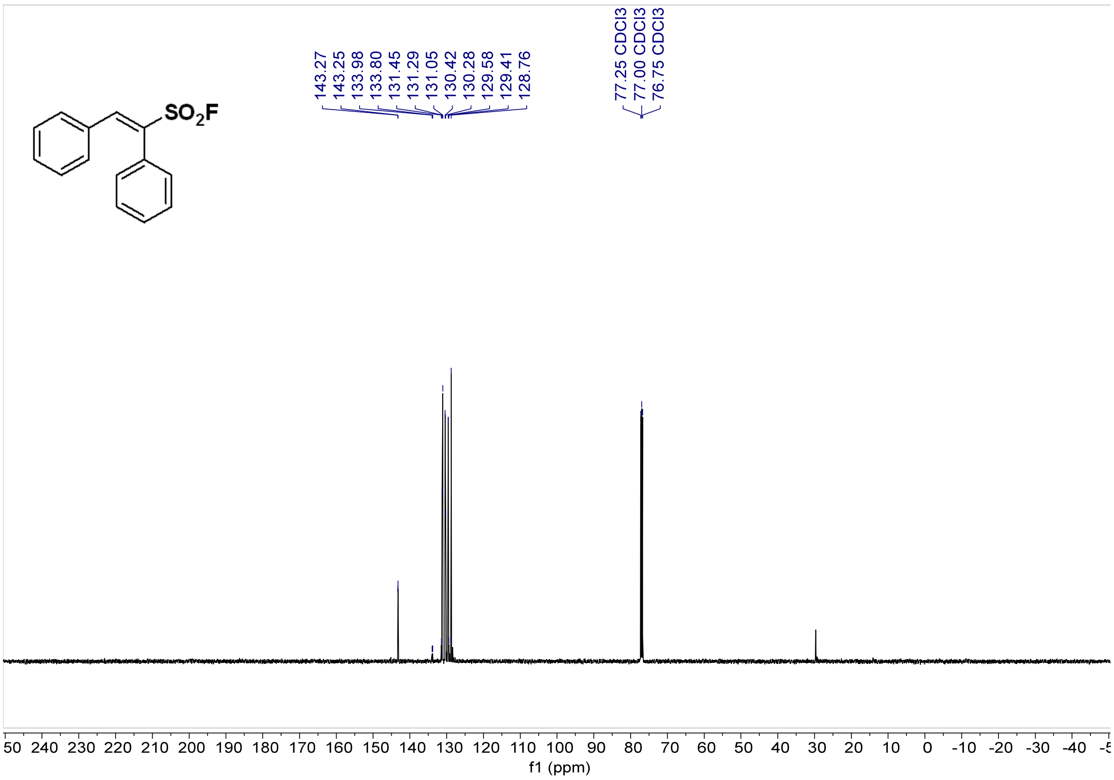

Supplementary Figure 63. ^{13}C NMR (101 MHz, room temperature, $CDCl_3$) spectra of product **3j**

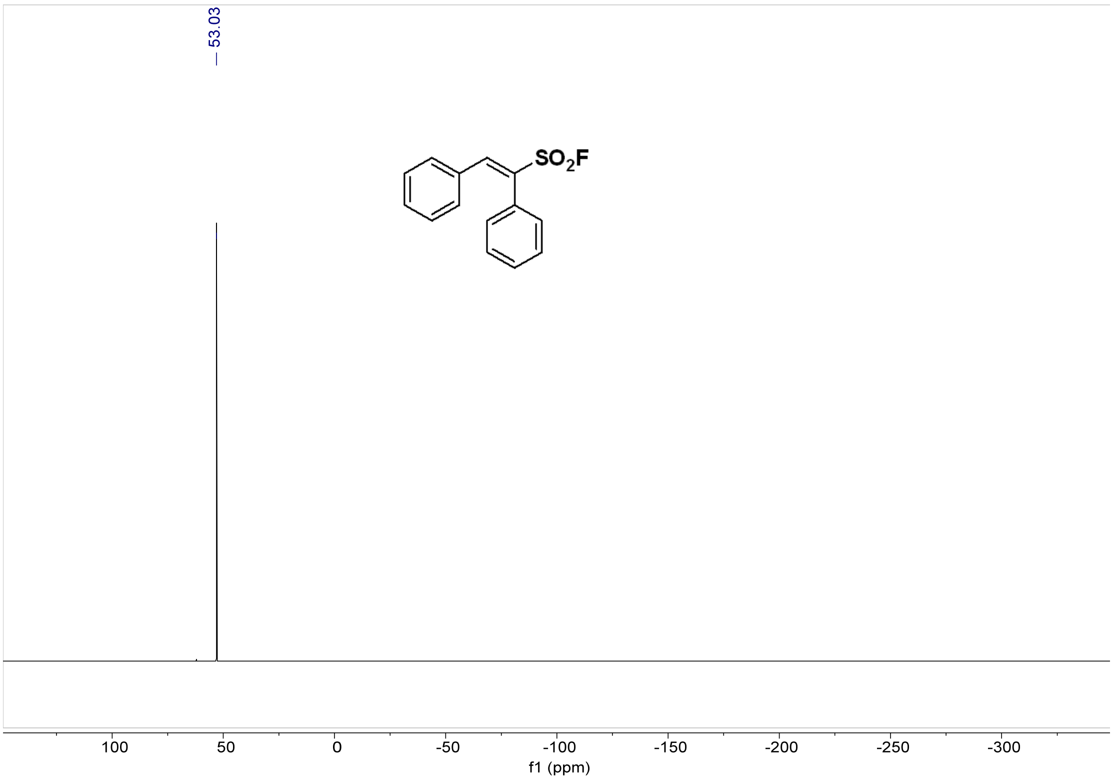

Supplementary Figure 64. ^{19}F NMR (376 MHz, room temperature, $CDCl_3$) spectra of product **3j**

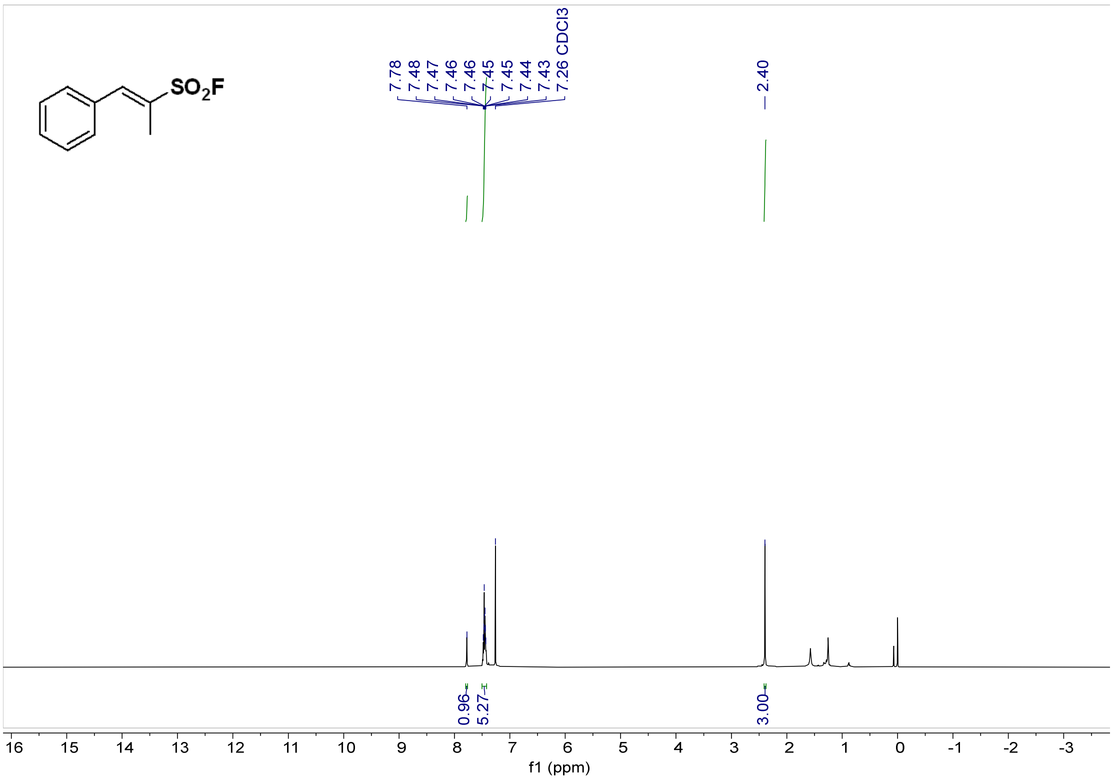

Supplementary Figure 65. ^{1}H NMR (500 MHz, room temperature, $CDCl_3$) spectra of product **3k**

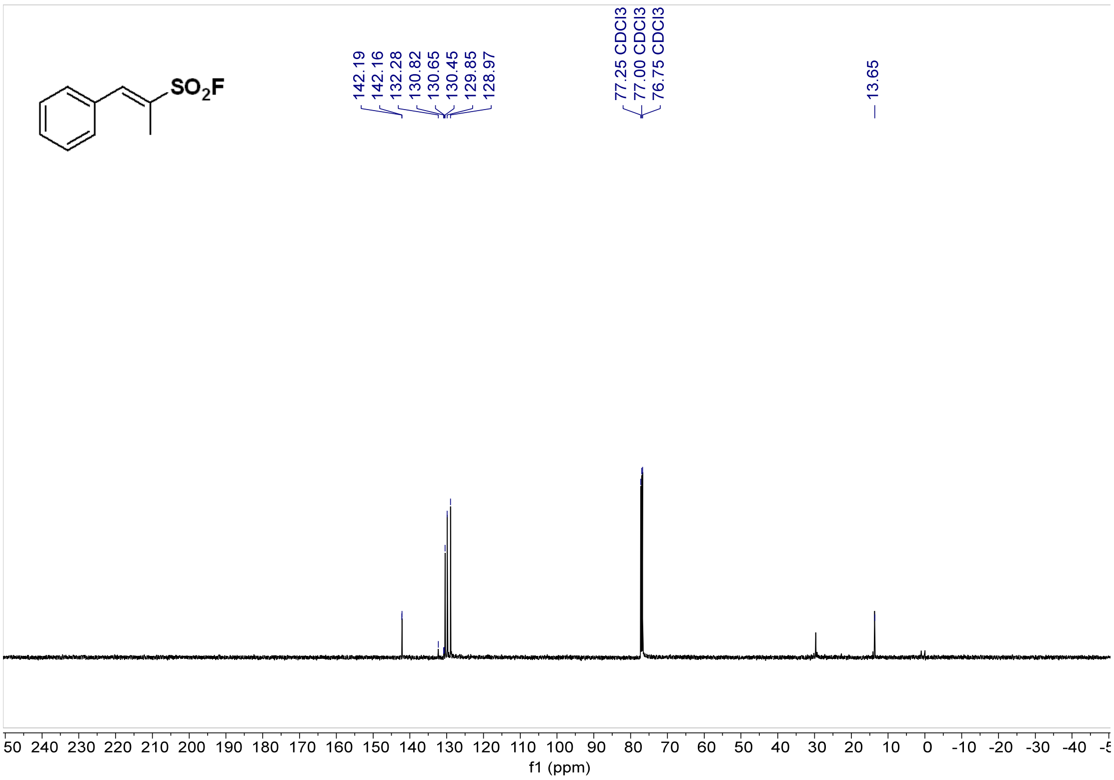

Supplementary Figure 66. ^{13}C NMR (126 MHz, room temperature, $CDCl_3$) spectra of product **3k**

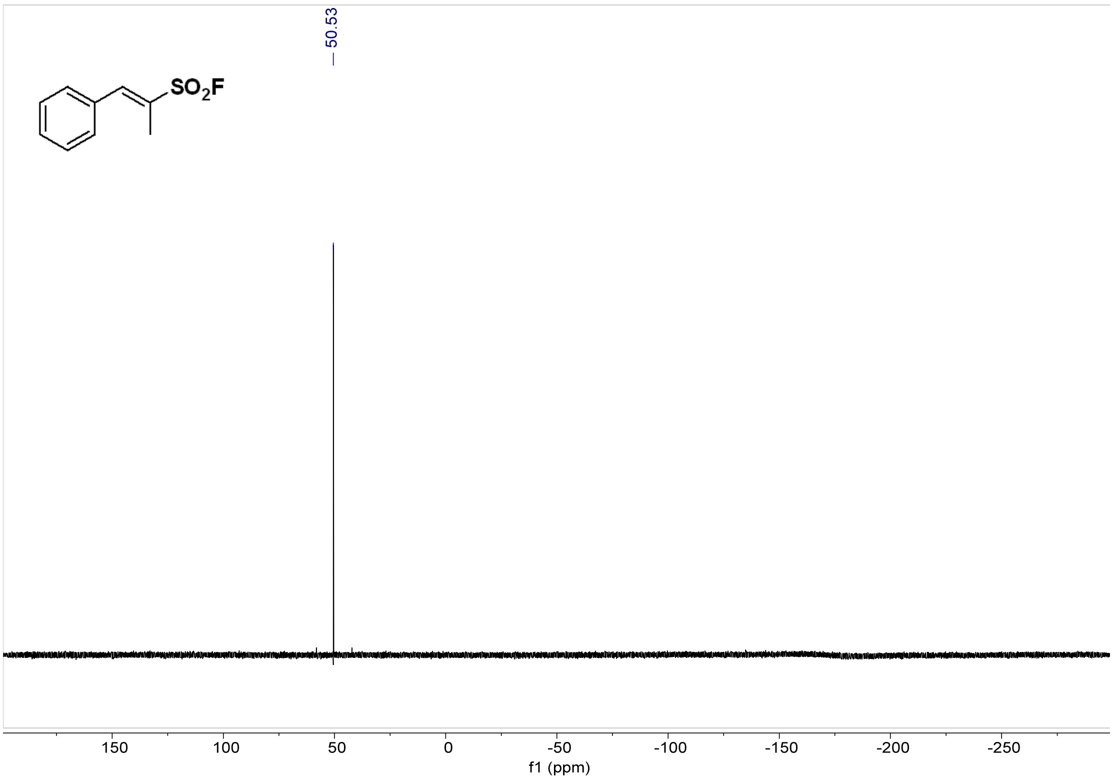

Supplementary Figure 67. ^{19}F NMR (471 MHz, room temperature, $CDCl_3$) spectra of product **3k**

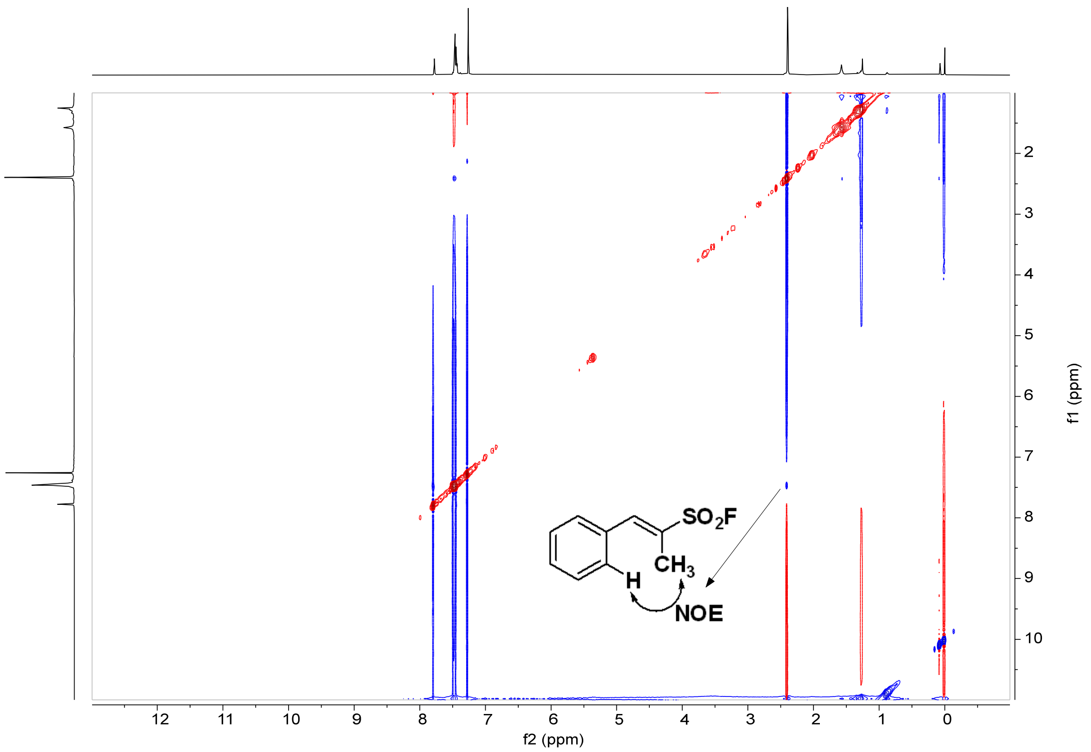

Supplementary Figure 68. NOESY (400 MHz, room temperature, $CDCl_3$) NMR spectra of product **3k**

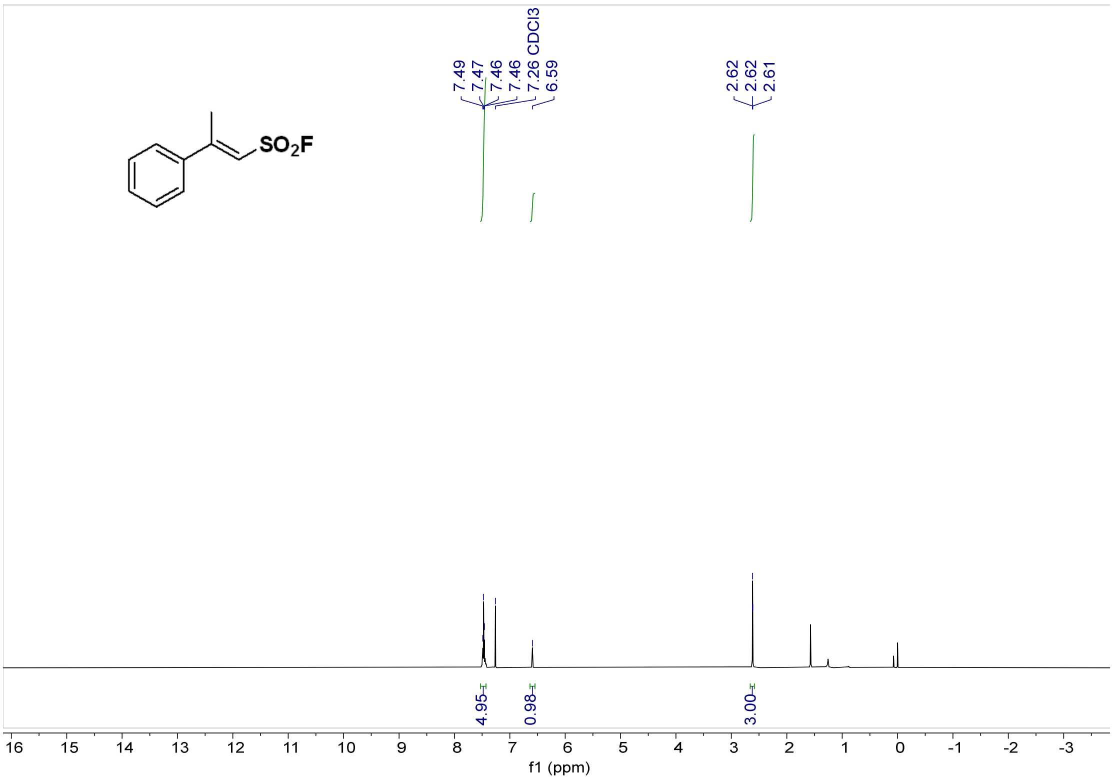

Supplementary Figure 69. ^1H NMR (500 MHz, room temperature, $CDCl_3$) spectra of product **3l**

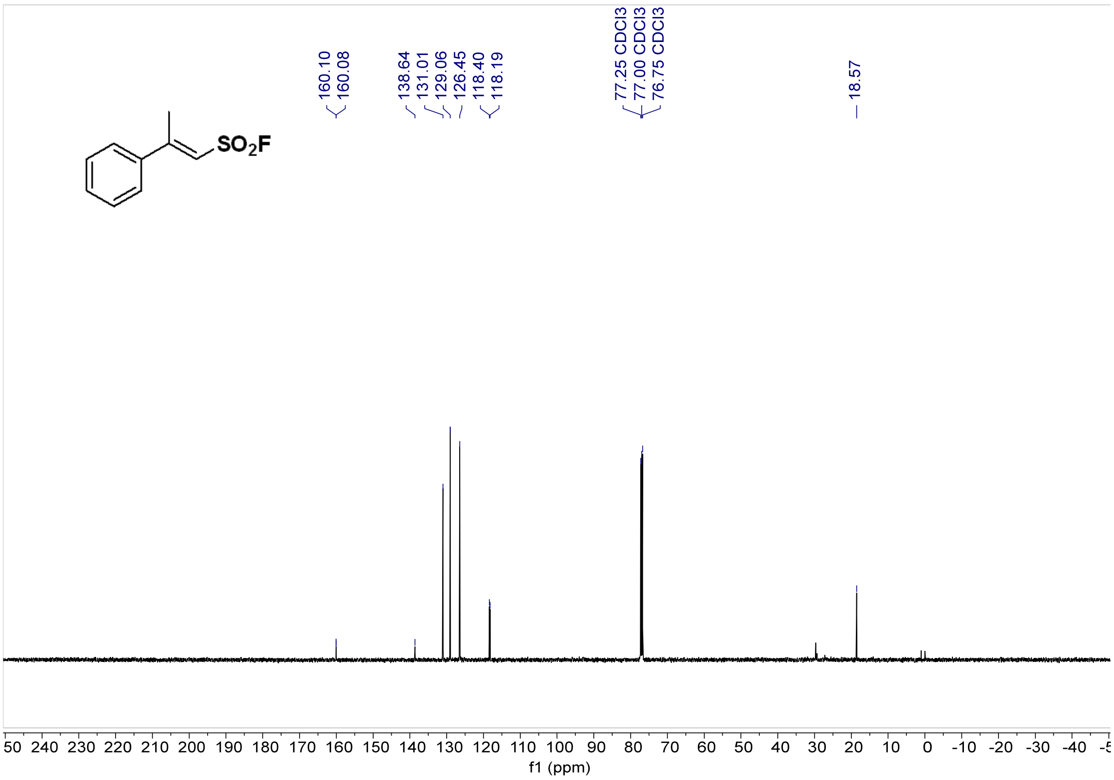

Supplementary Figure 70. ^{13}C NMR (126 MHz, room temperature, $CDCl_3$) spectra of product **3l**

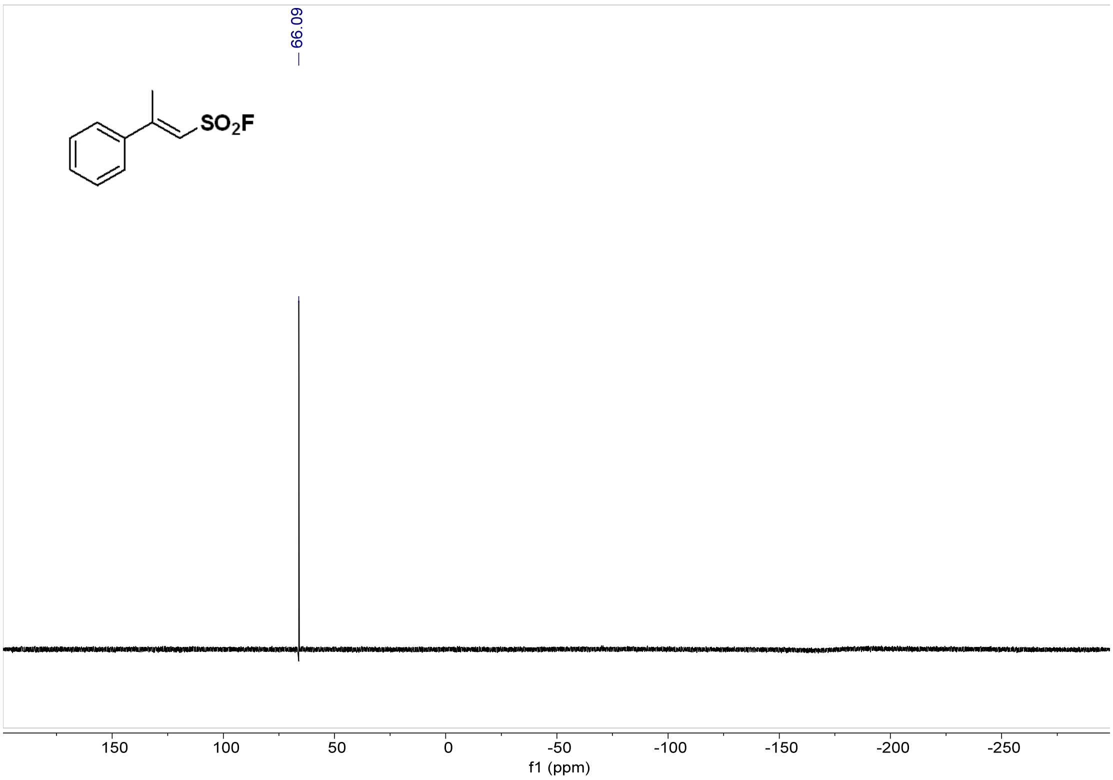

Supplementary Figure 71. ^{19}F NMR (471 MHz, room temperature, $CDCl_3$) spectra of product **3j**

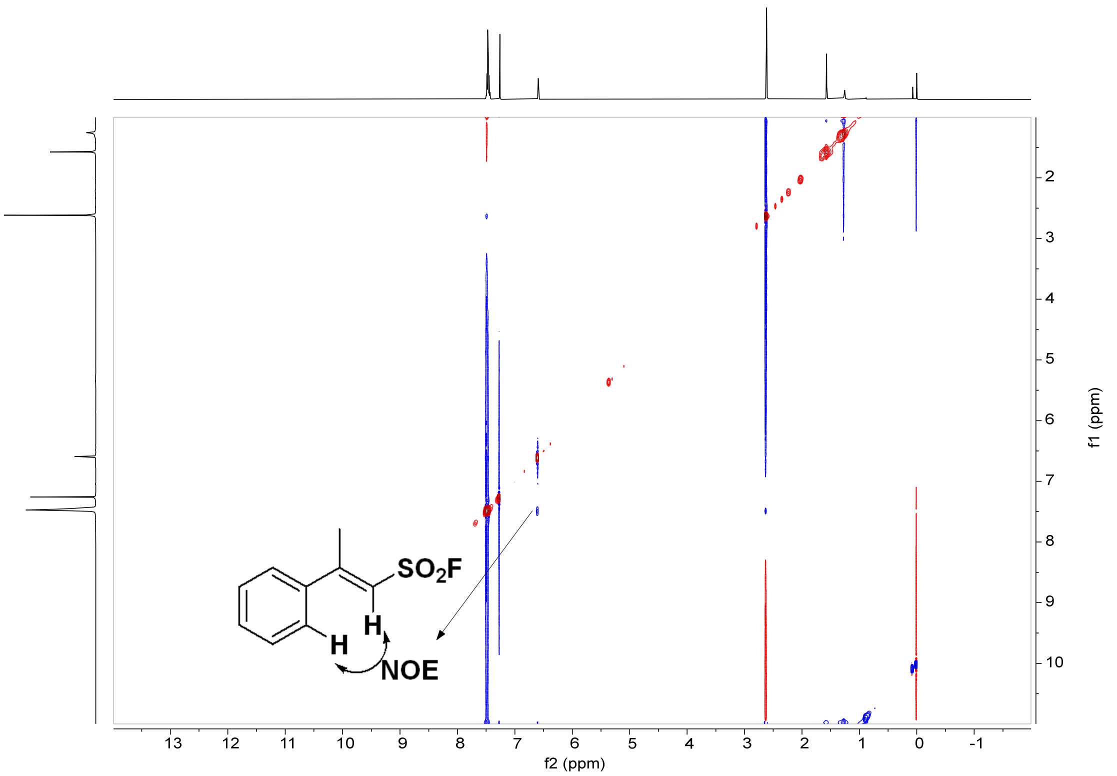

Supplementary Figure 72. NOESY (400 MHz, room temperature, CDCl3) spectra of product **3j**

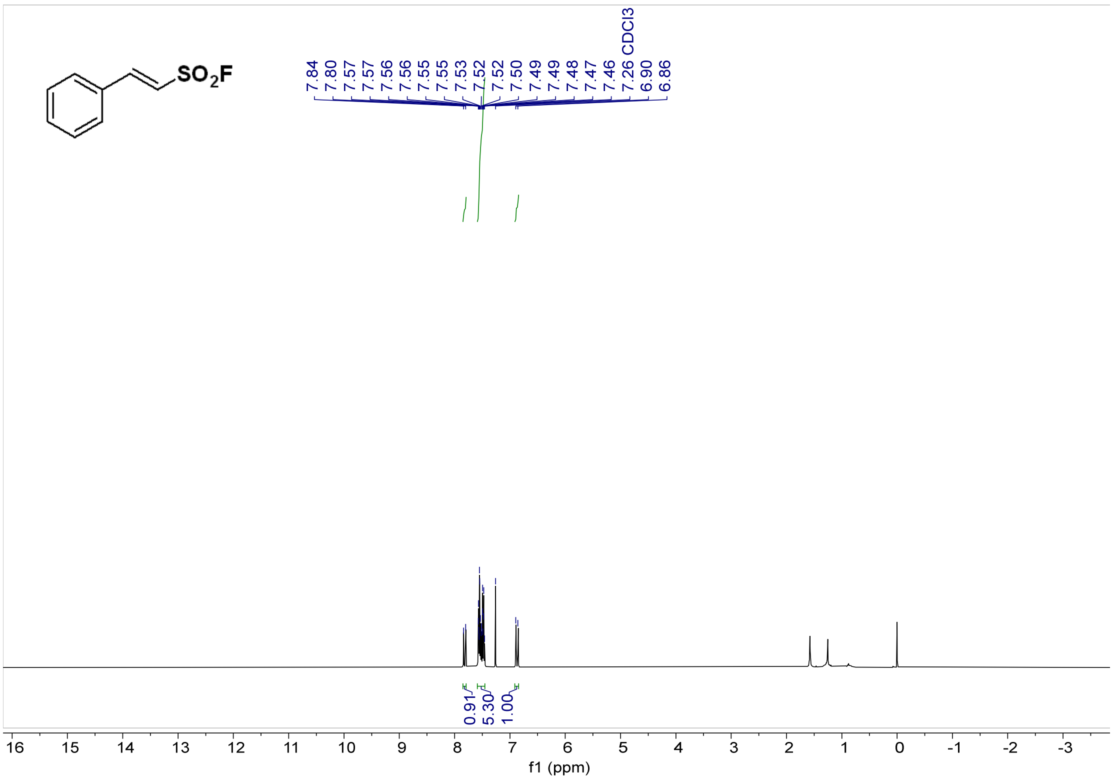

Supplementary Figure 73. ^{1}H NMR (400 MHz, room temperature, CDCl3) spectra of product **3m**

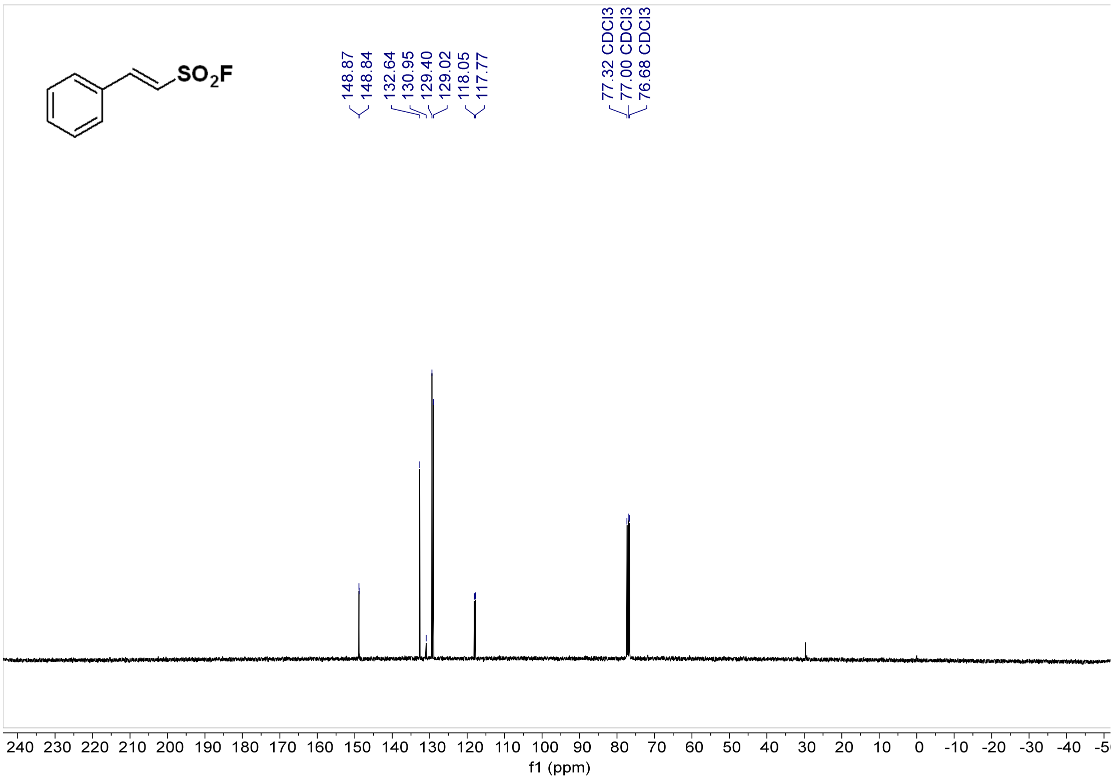

Supplementary Figure 74. ^{13}C NMR (101 MHz, room temperature, $CDCl_3$) spectra of product **3m**

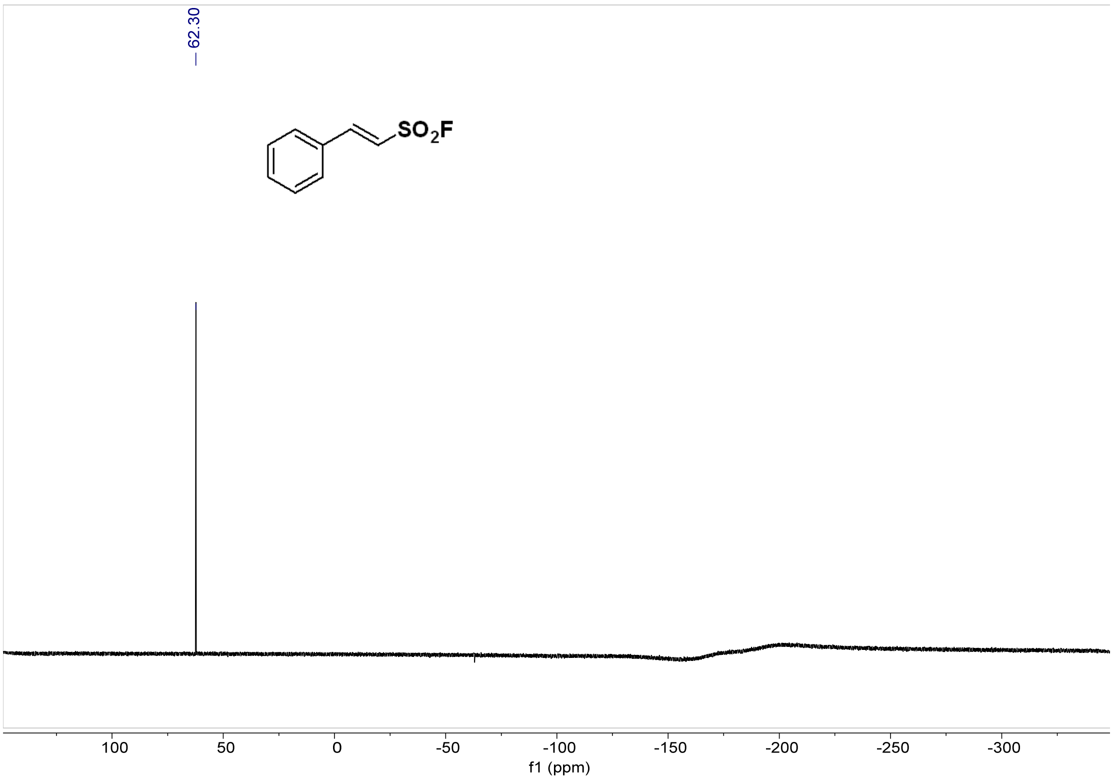

Supplementary Figure 75. ^{19}F NMR (376 MHz, room temperature, $CDCl_3$) spectra of product **3m**

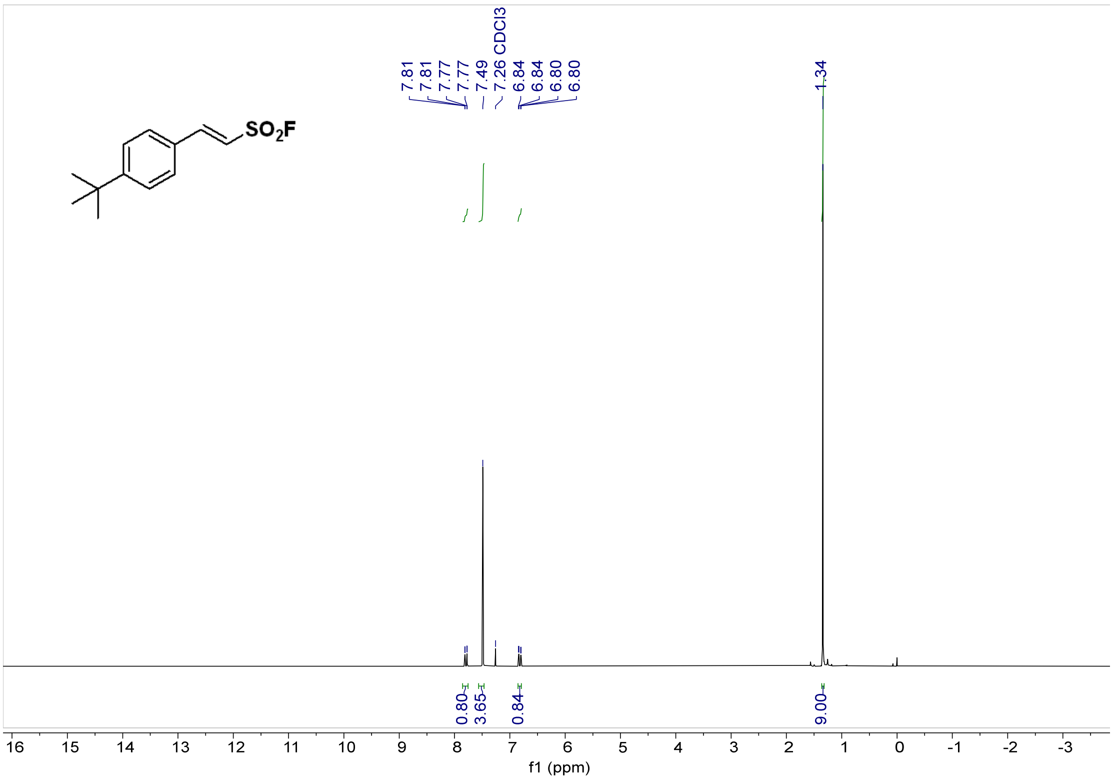

Supplementary Figure 76. ^{1}H NMR (400 MHz, room temperature, $CDCl_3$) spectra of product **3n**

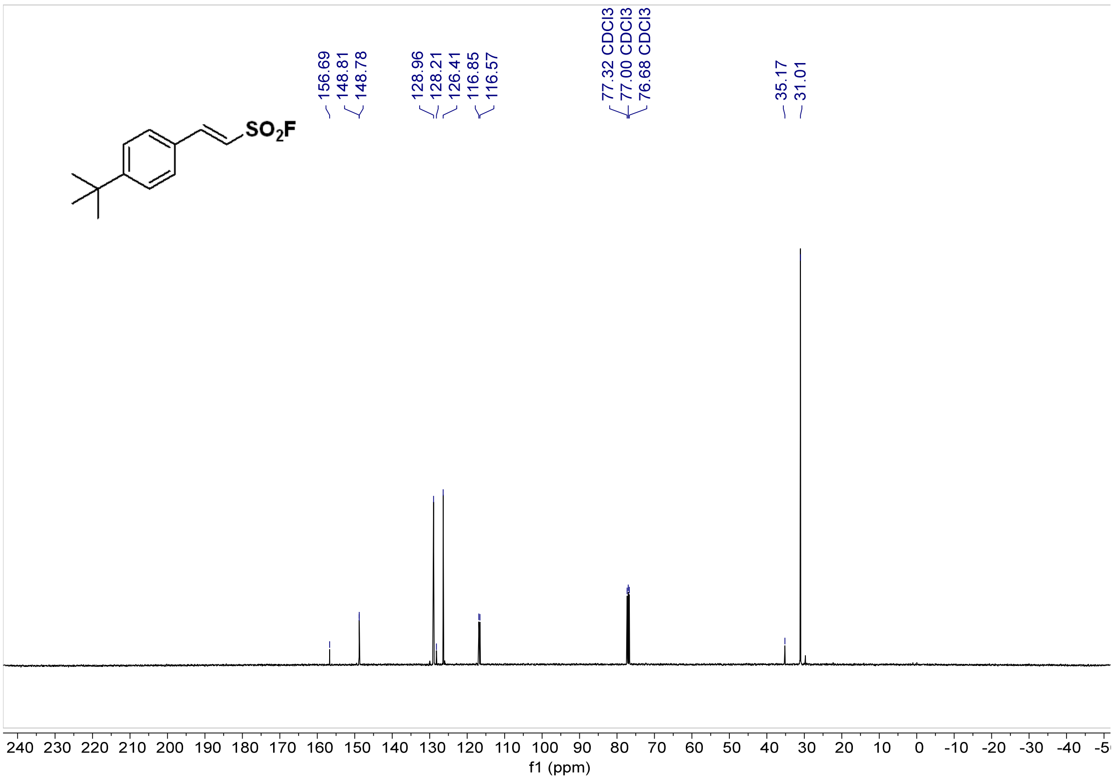

Supplementary Figure 77. ^{13}C NMR (101 MHz, room temperature, $CDCl_3$) spectra of product **3n**

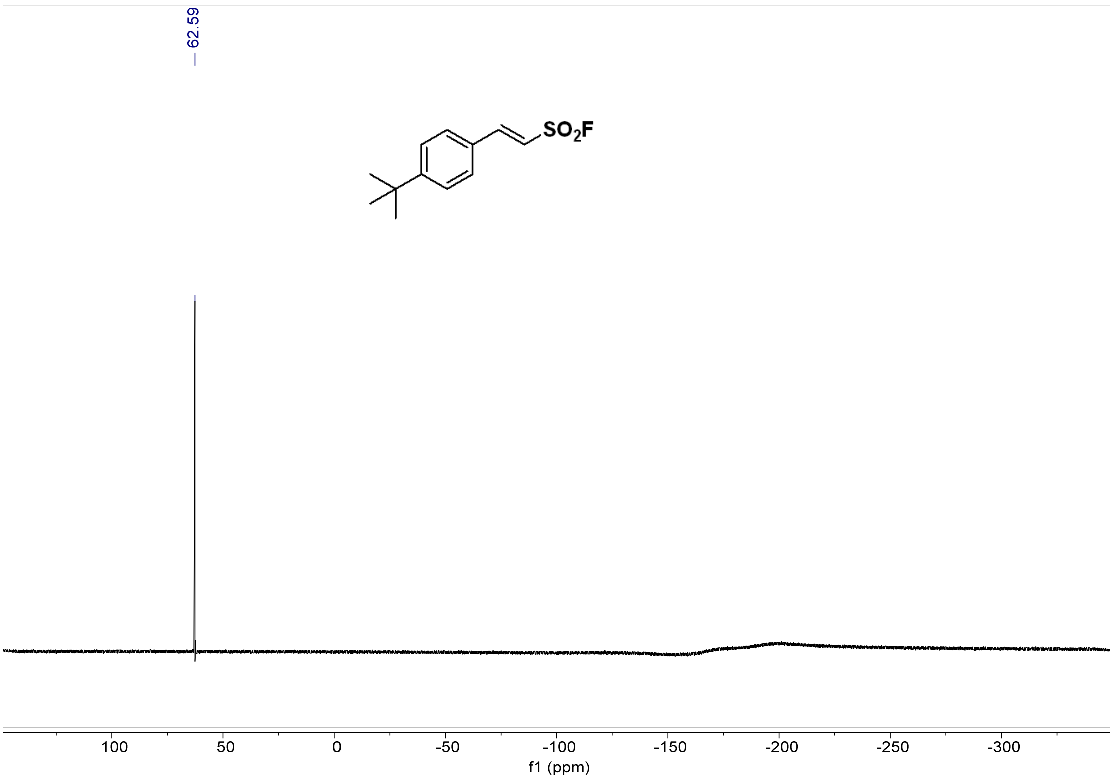

Supplementary Figure 78. ^{19}F NMR (376 MHz, room temperature, $CDCl_3$) spectra of product **3n**

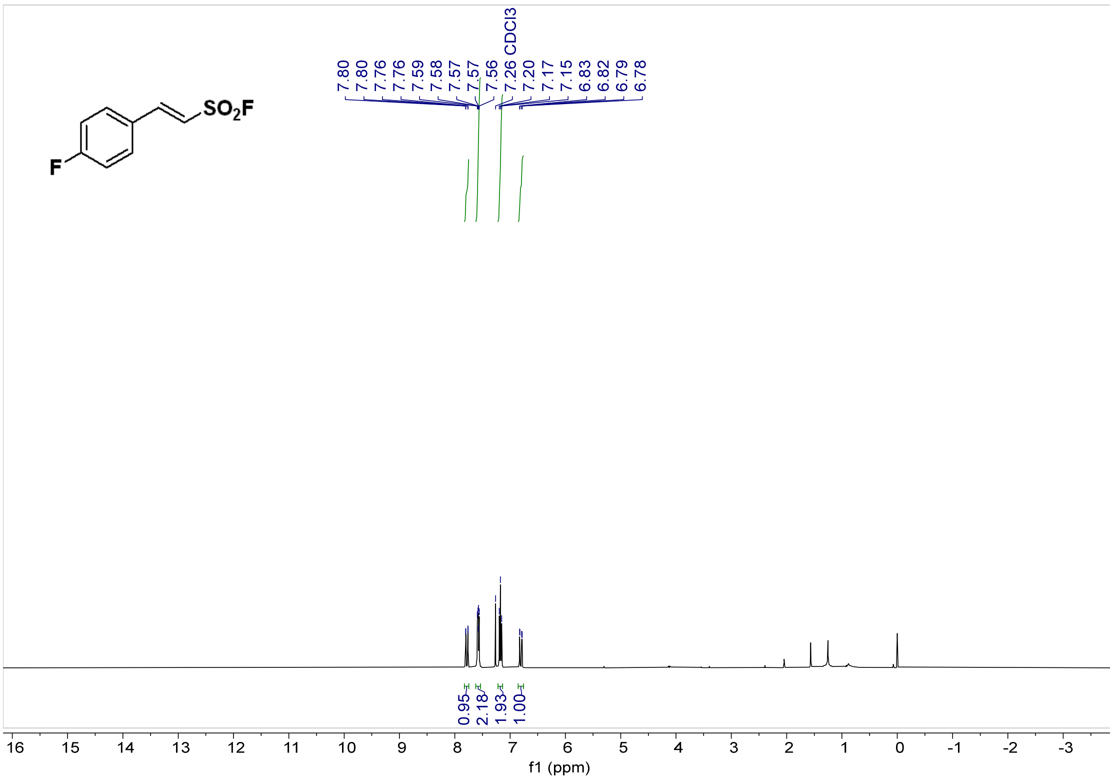

Supplementary Figure 79. ^{1}H NMR (400 MHz, room temperature, $CDCl_3$) spectra of product **3o**

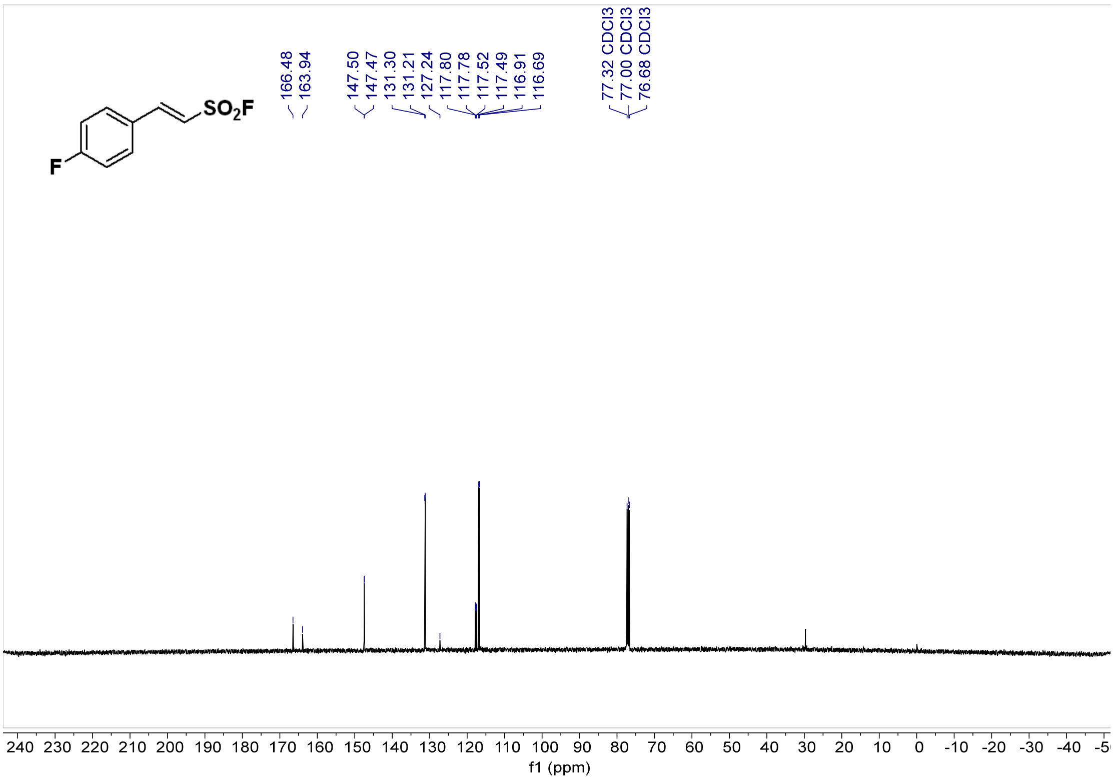

Supplementary Figure 80. ^{13}C NMR (101 MHz, room temperature, $CDCl_3$) spectra of product **3o**

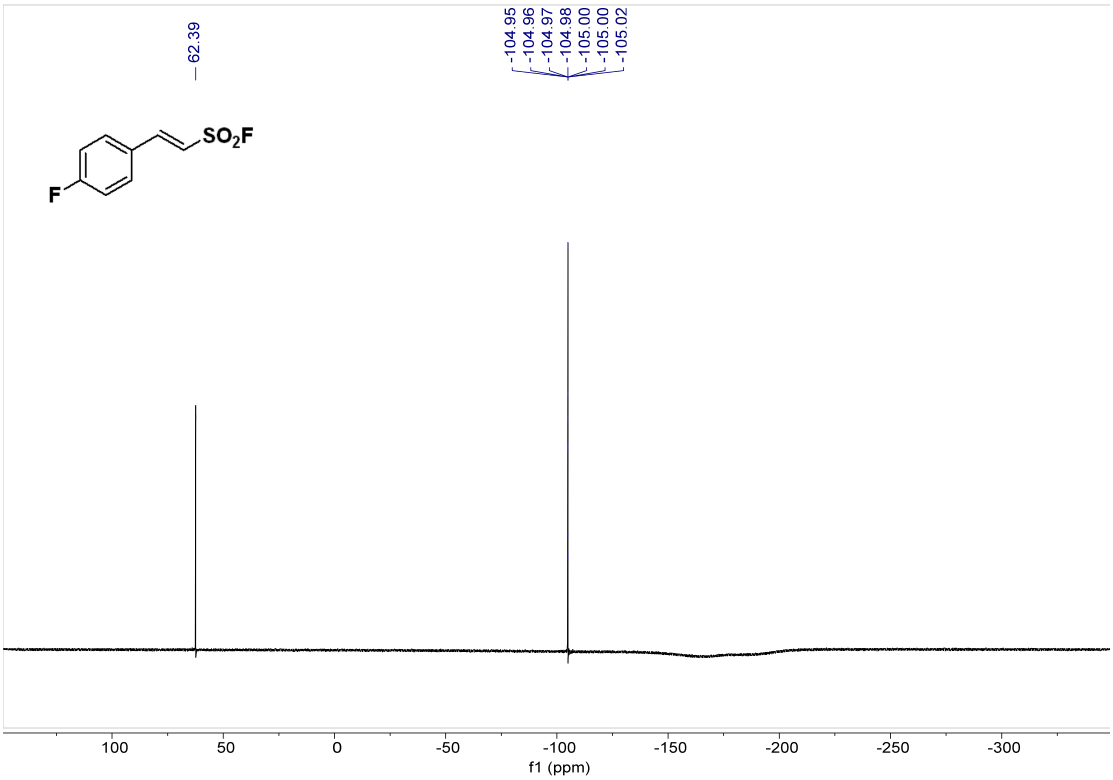

Supplementary Figure 81. ^{19}F NMR (376 MHz, room temperature, $CDCl_3$) spectra of product **3o**

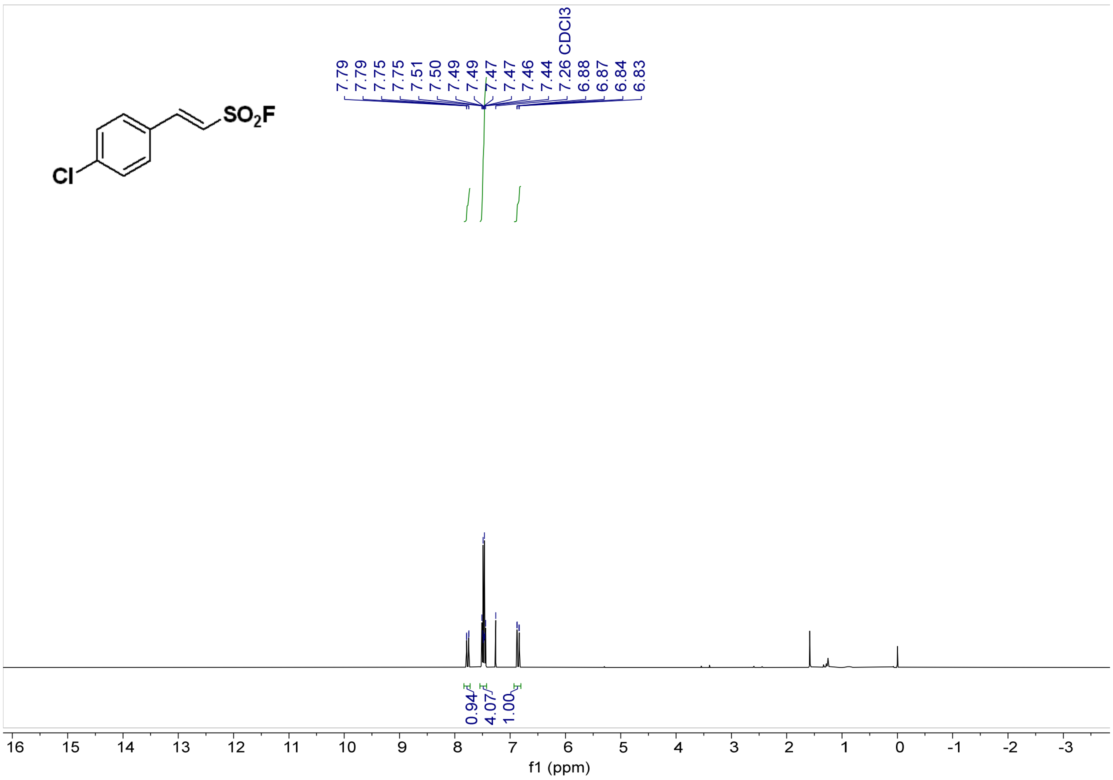

Supplementary Figure 82. ^{1}H NMR (400 MHz, room temperature, $CDCl_3$) spectra of product **3p**

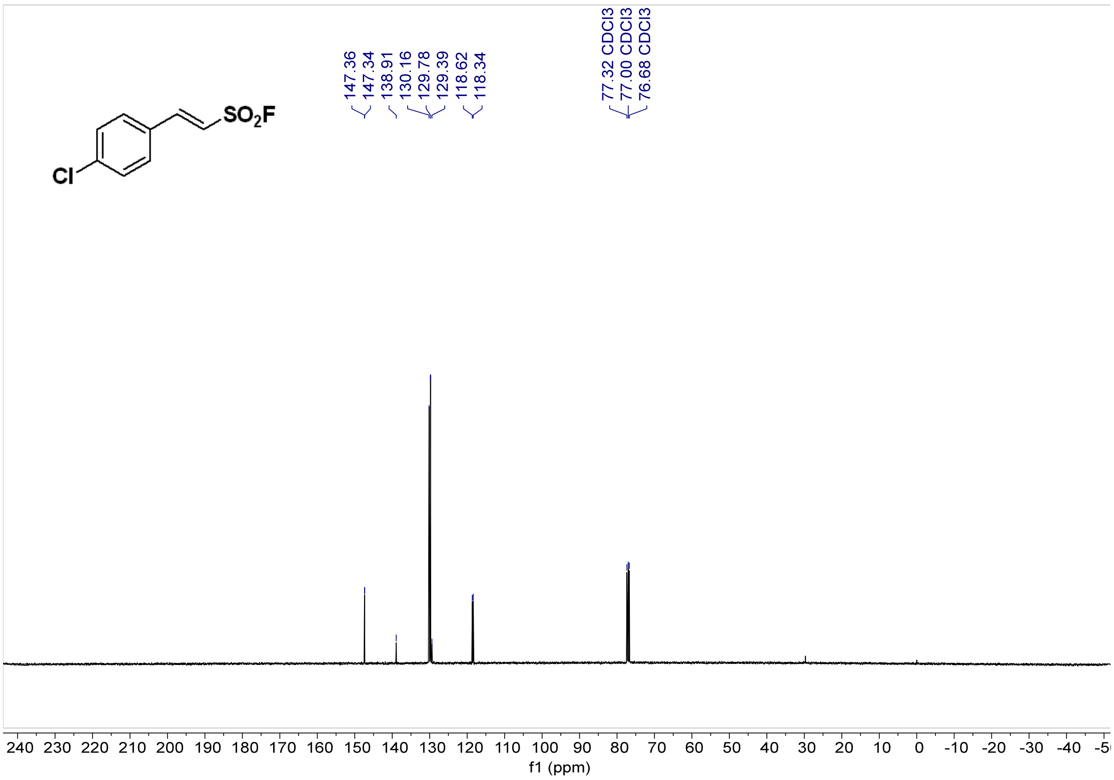

Supplementary Figure 83. ^{13}C NMR (101 MHz, room temperature, $CDCl_3$) spectra of product **3p**

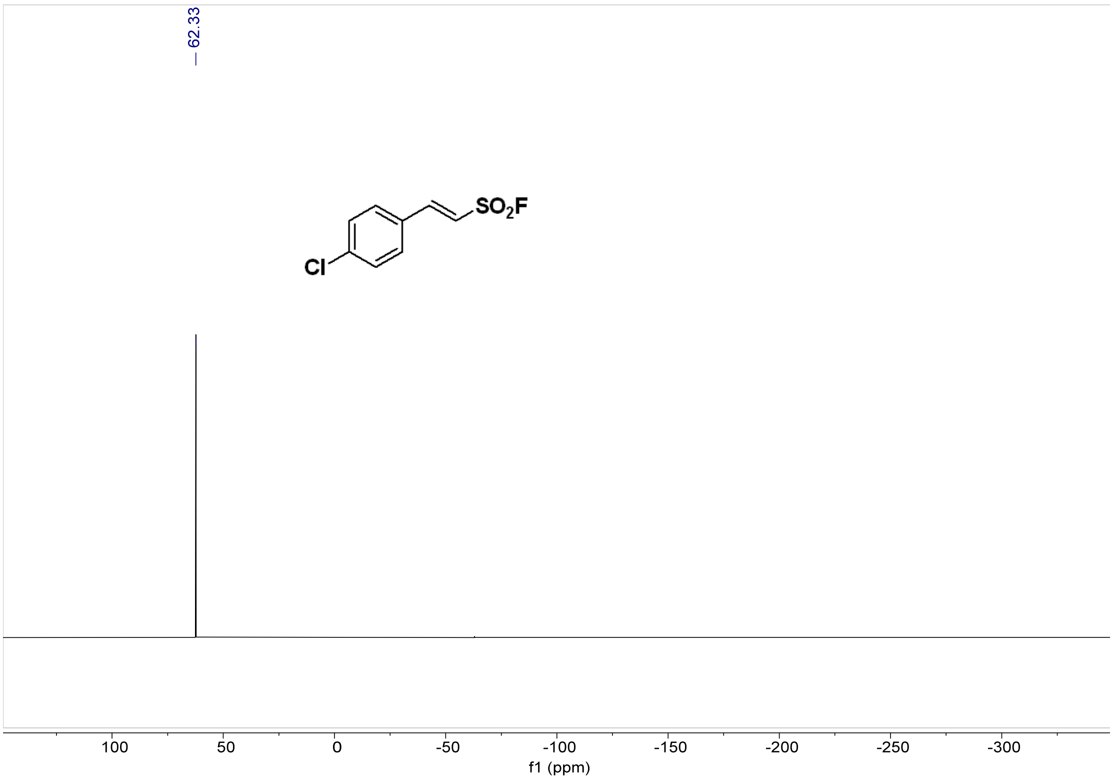

Supplementary Figure 84. ^{19}F NMR (376 MHz, room temperature, $CDCl_3$) spectra of product **3p**

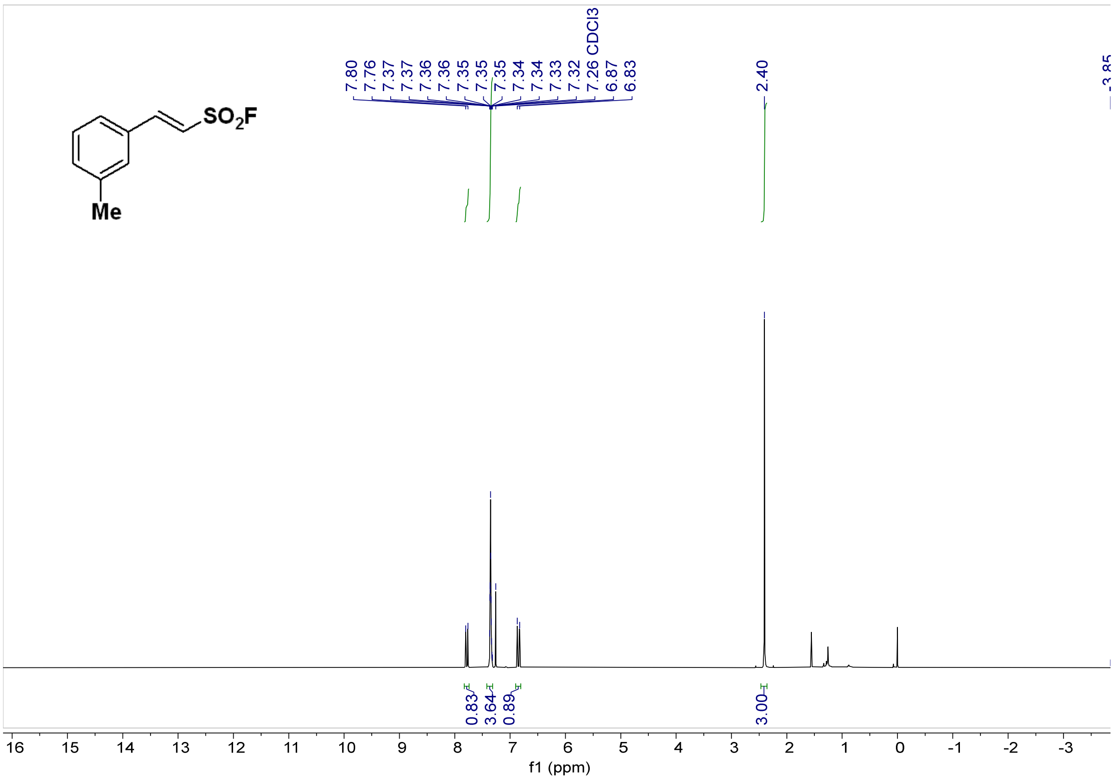

Supplementary Figure 85. ^{1}H NMR (400 MHz, room temperature, $CDCl_3$) spectra of product **3q**

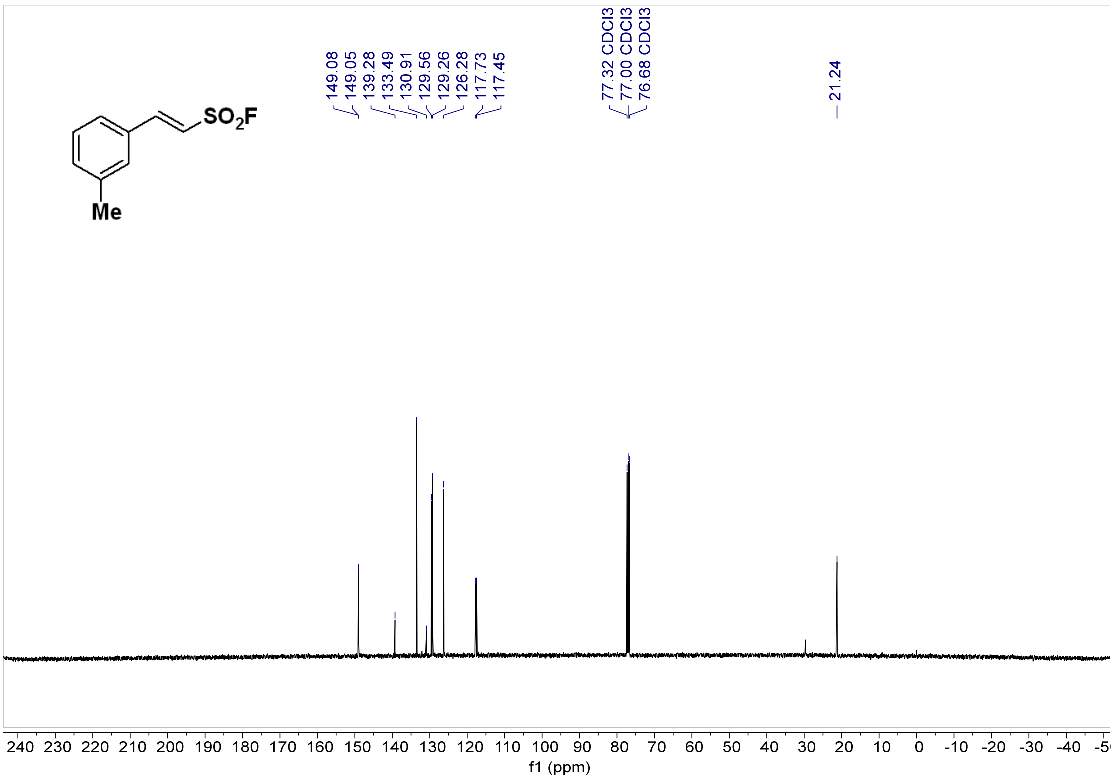

Supplementary Figure 86. ^{13}C NMR (101 MHz, room temperature, $CDCl_3$) spectra of product **3q**

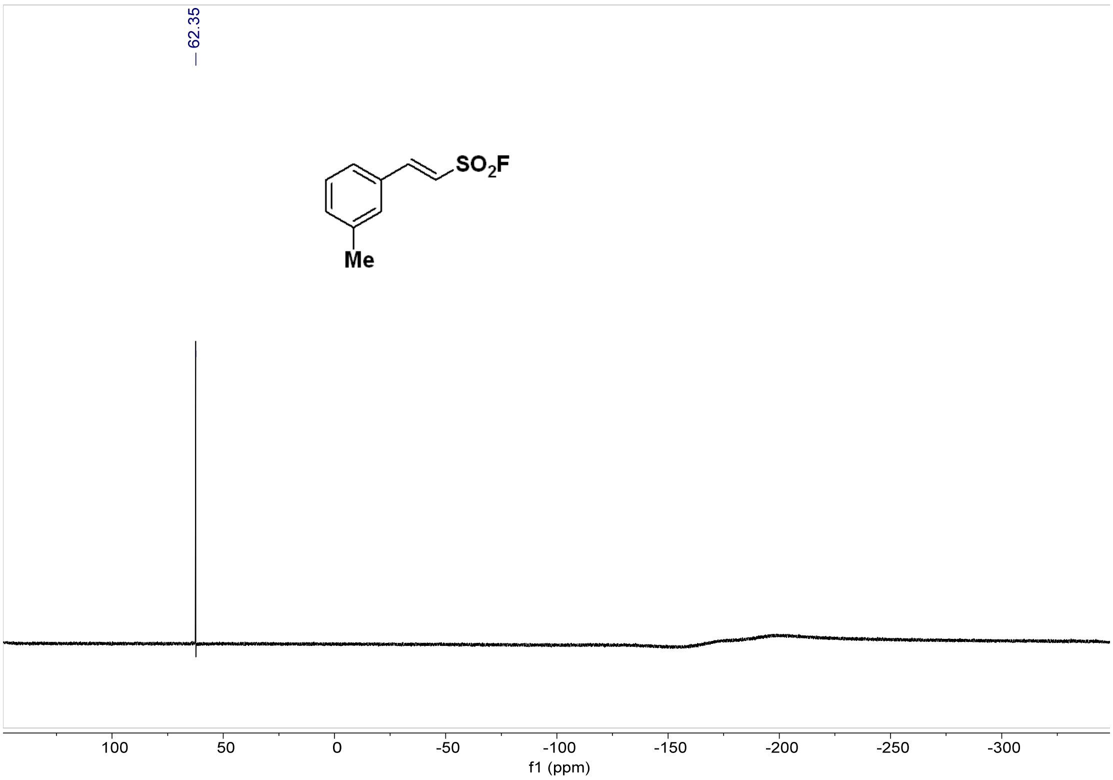

Supplementary Figure 87. ^{19}F NMR (376 MHz, room temperature, $CDCl_3$) spectra of product **3q**

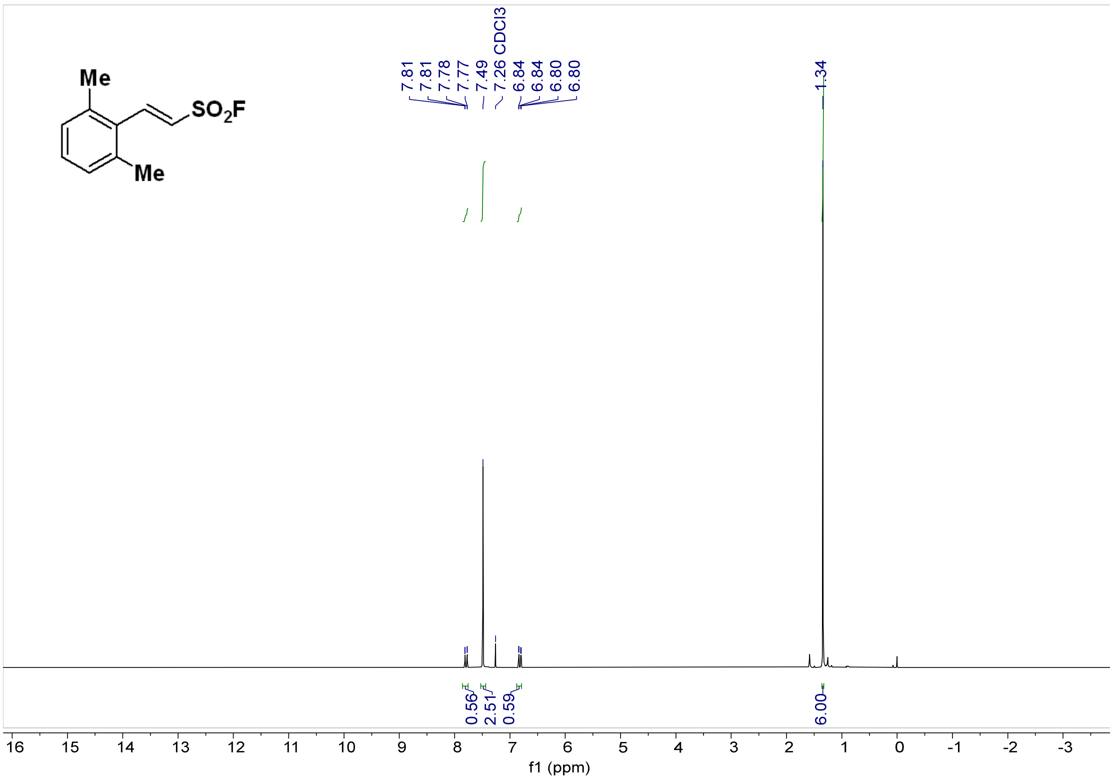

Supplementary Figure 88. ^{1}H NMR (400 MHz, room temperature, $CDCl_3$) spectra of product **3r**

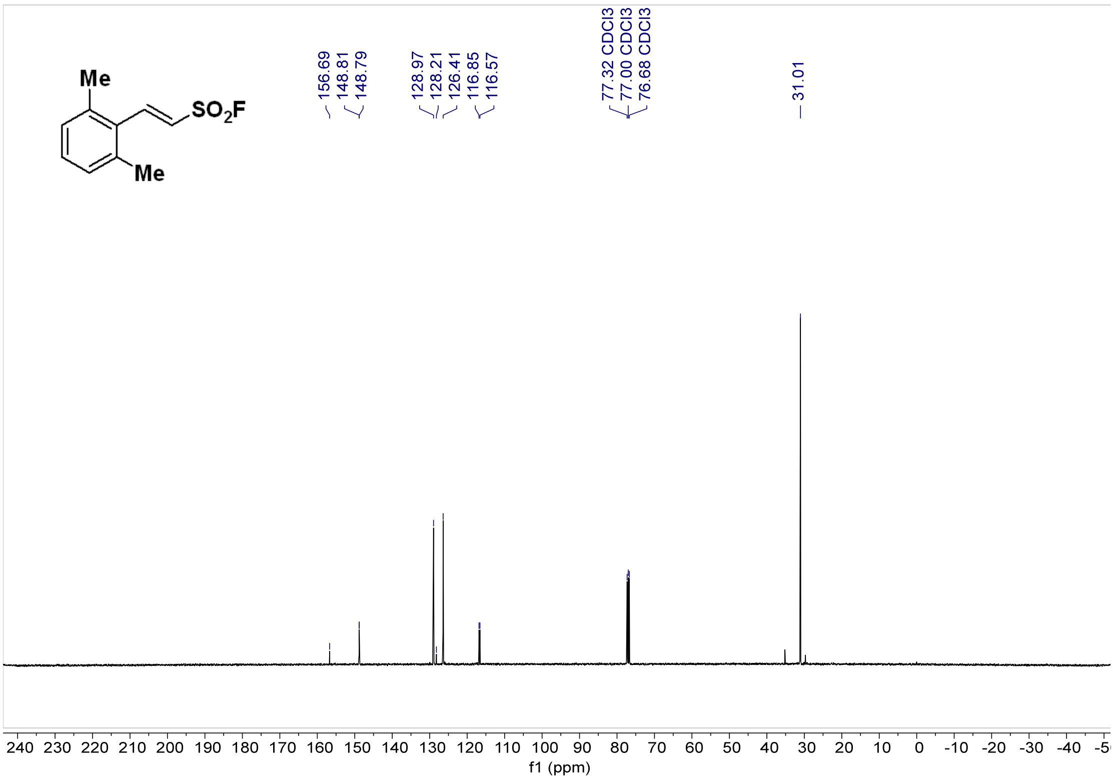

Supplementary Figure 89. ^{13}C NMR (101 MHz, room temperature, $CDCl_3$) spectra of product **3r**

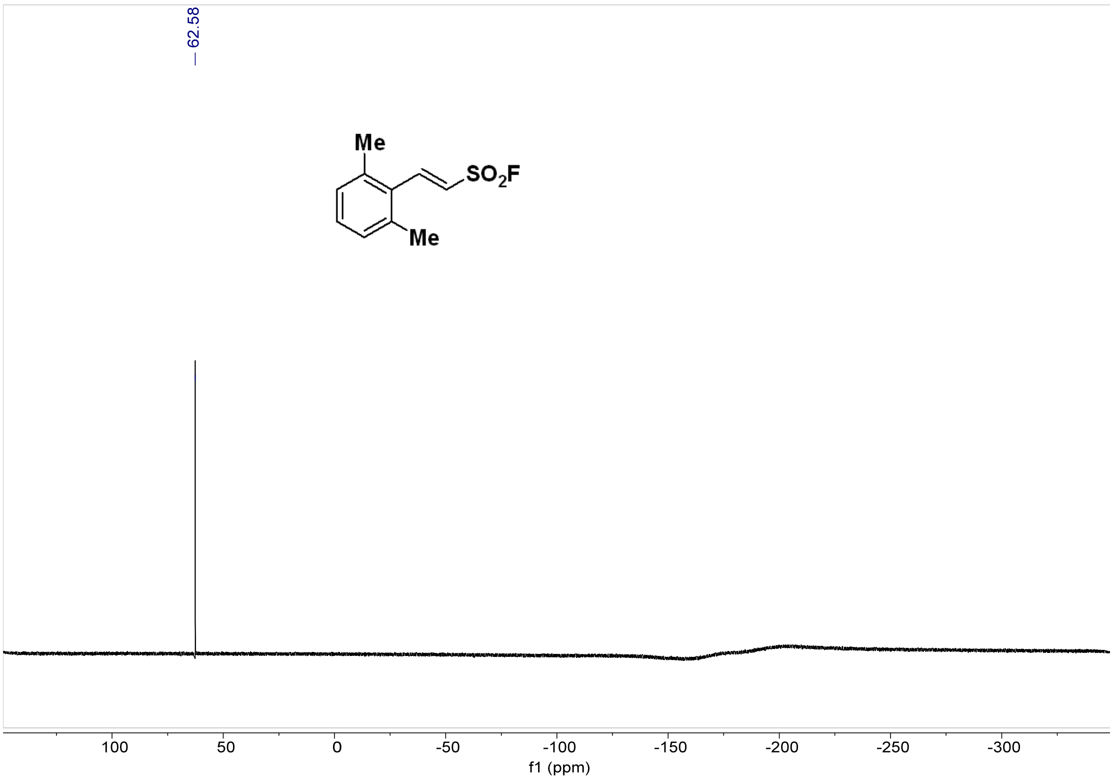

Supplementary Figure 90. ^{19}F NMR (376 MHz, room temperature, $CDCl_3$) spectra of product **3r**

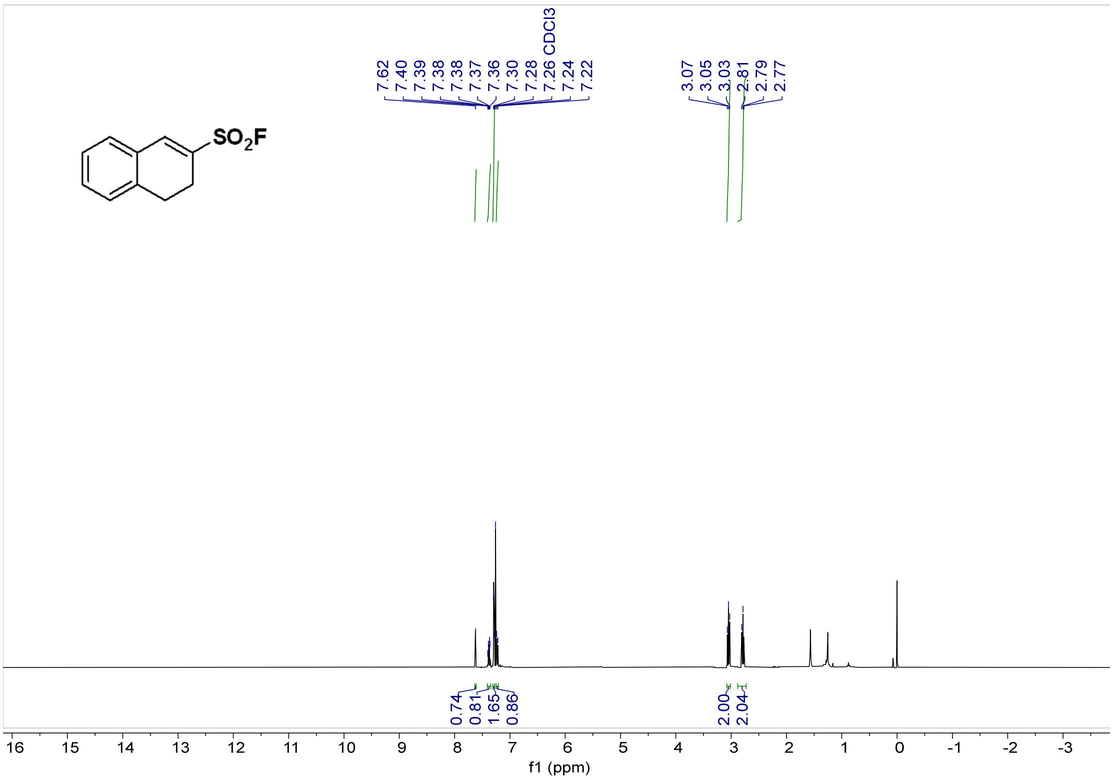

Supplementary Figure 91. ^{1}H NMR (400 MHz, room temperature, $CDCl_3$) spectra of product **3s**

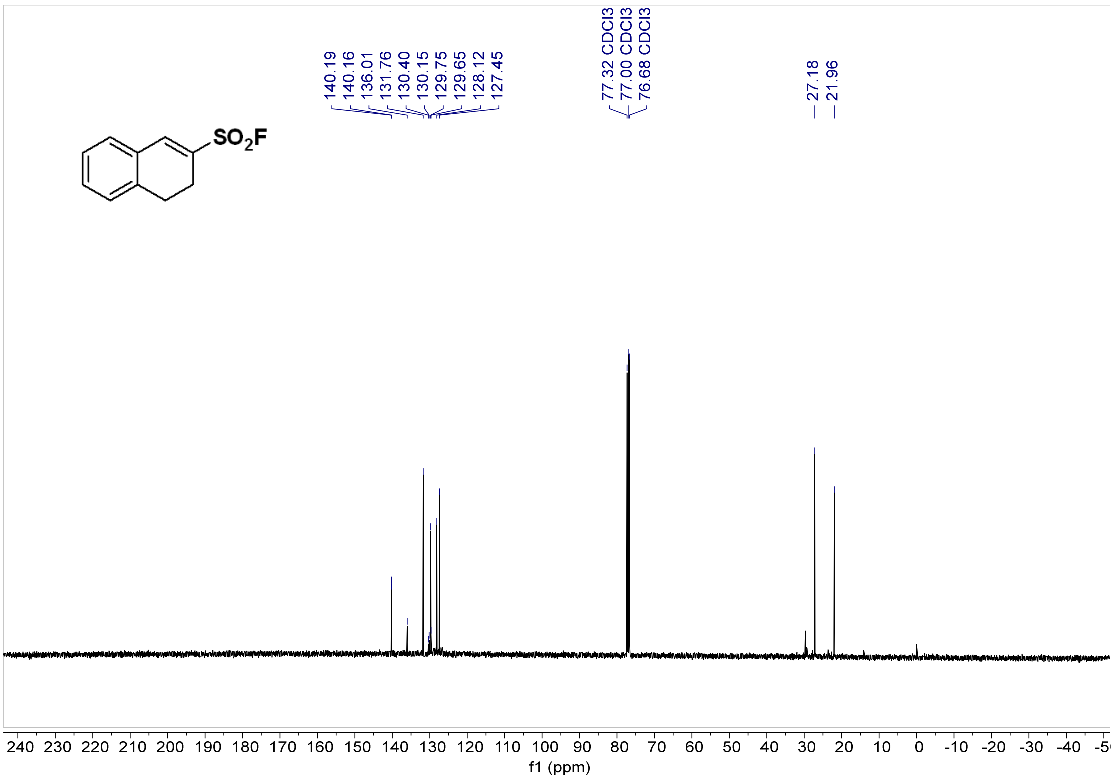

Supplementary Figure 92. ^{13}C NMR (101 MHz, room temperature, $CDCl_3$) spectra of product **3s**

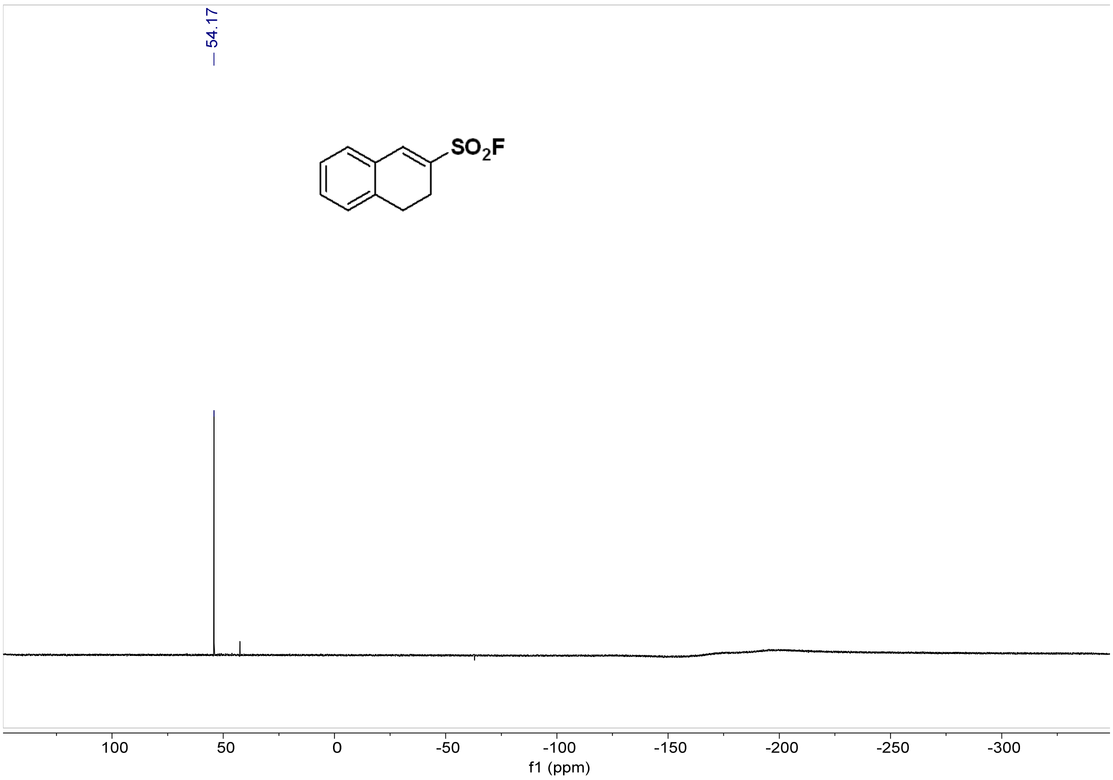

Supplementary Figure 93. ^{19}F NMR (376 MHz, room temperature, $CDCl_3$) spectra of product **3s**

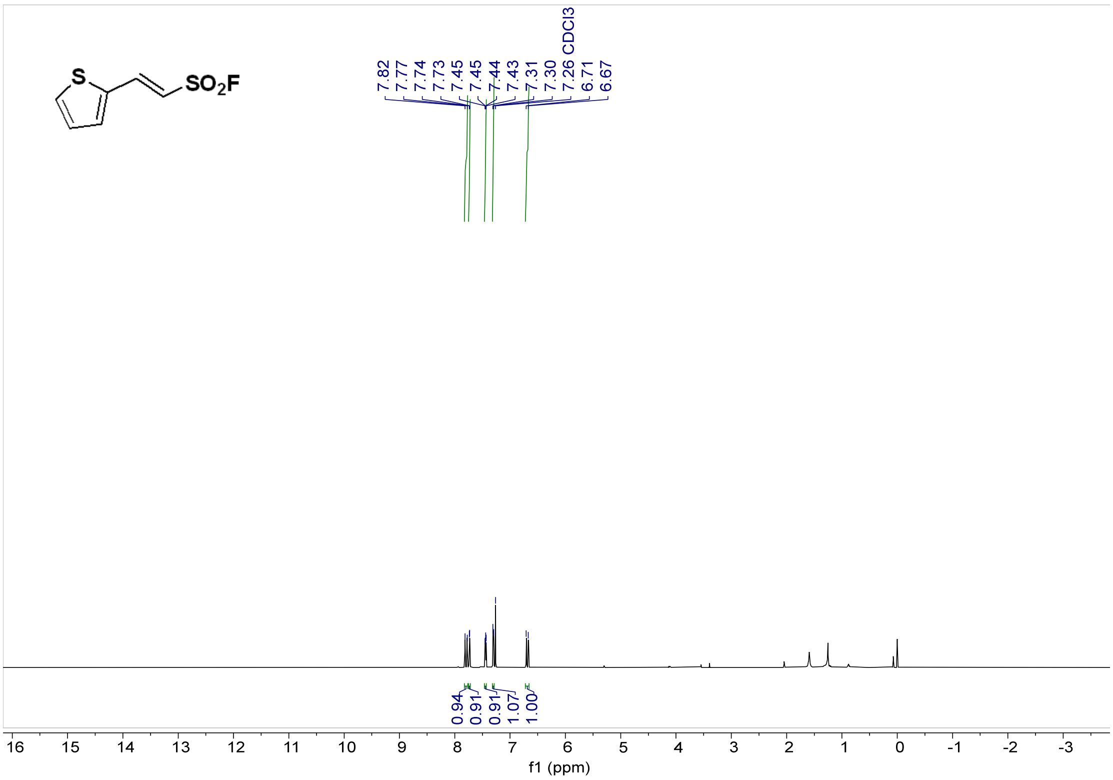

Supplementary Figure 94. ^{1}H NMR (400 MHz, room temperature, $CDCl_3$) spectra of product **3t**

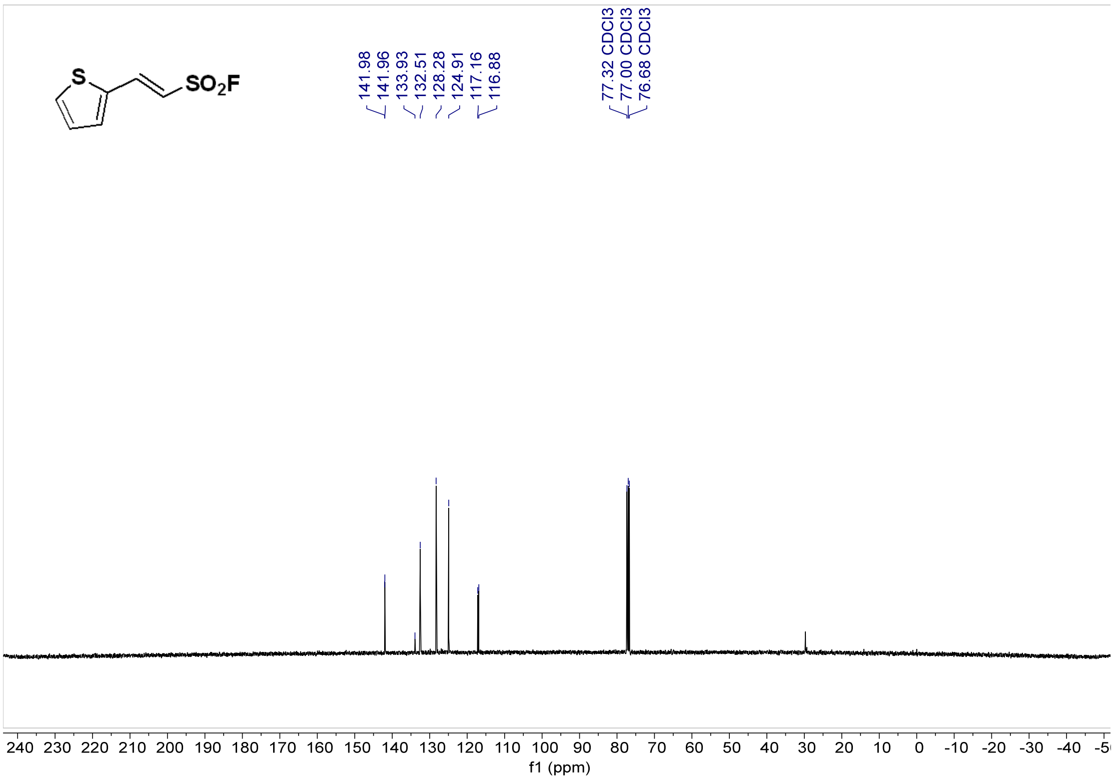

Supplementary Figure 95. ^{13}C NMR (101 MHz, room temperature, $CDCl_3$) spectra of product **3t**

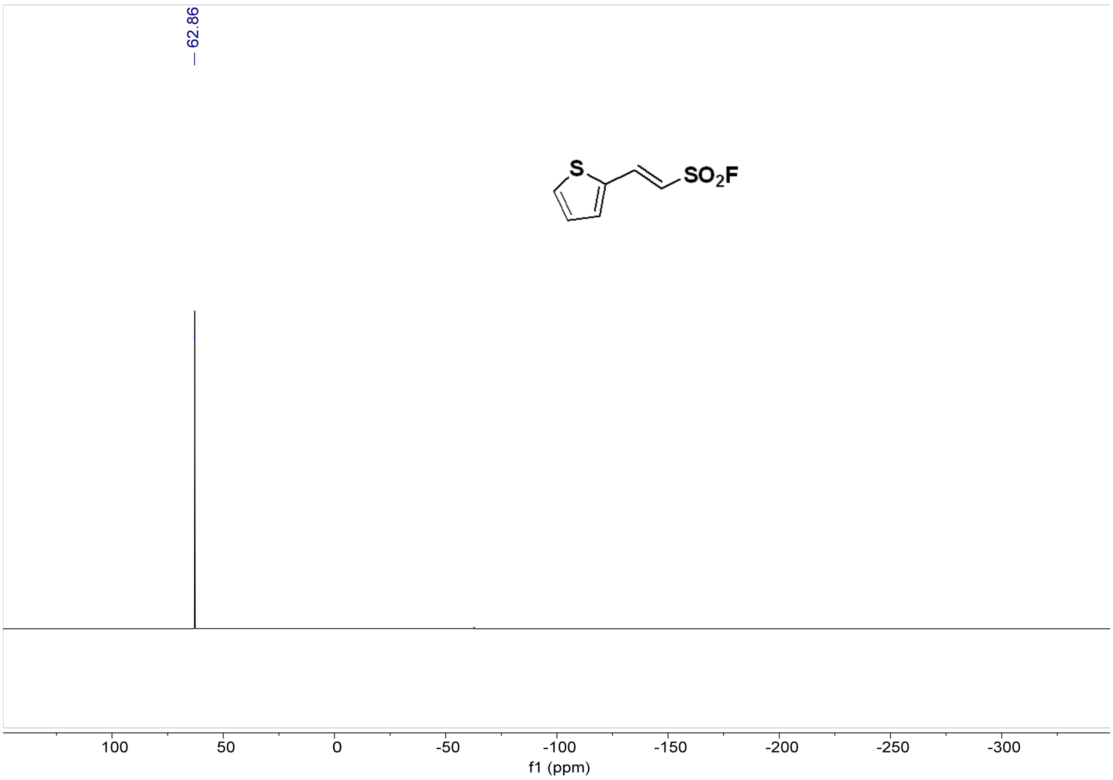

Supplementary Figure 96. ^{19}F NMR (376 MHz, room temperature, $CDCl_3$) spectra of product **3t**

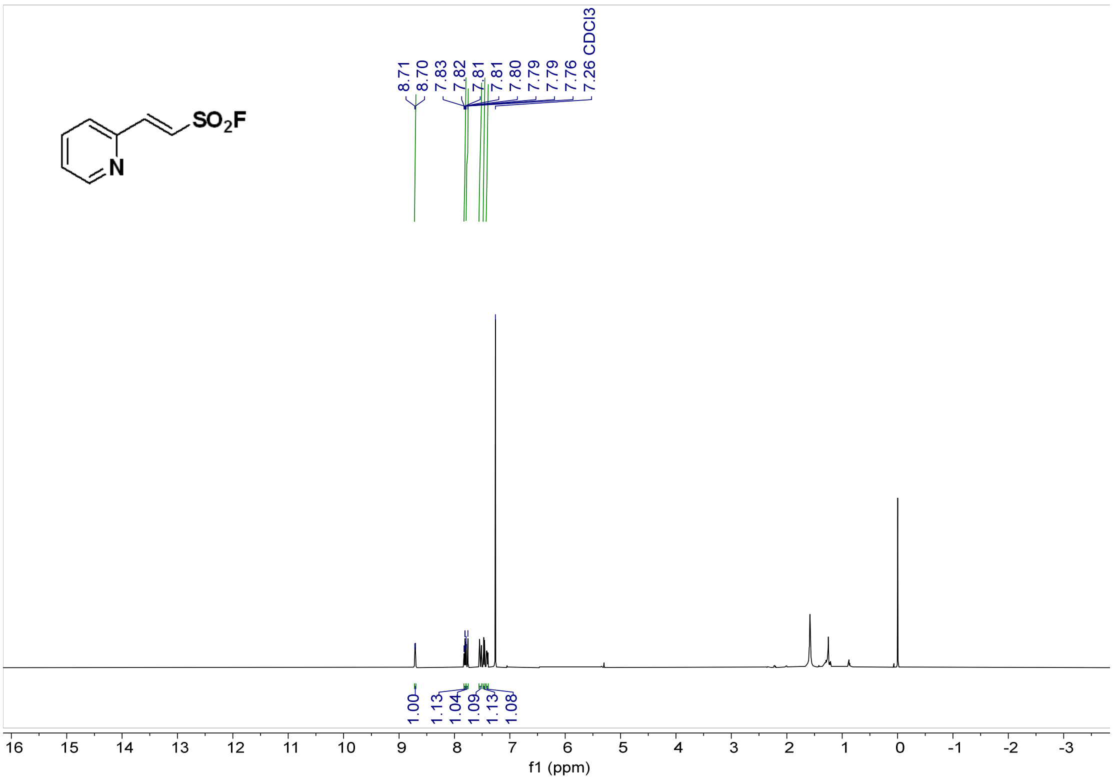

Supplementary Figure 97. ^{1}H NMR (500 MHz, room temperature, $CDCl_3$) spectra of product **3u**

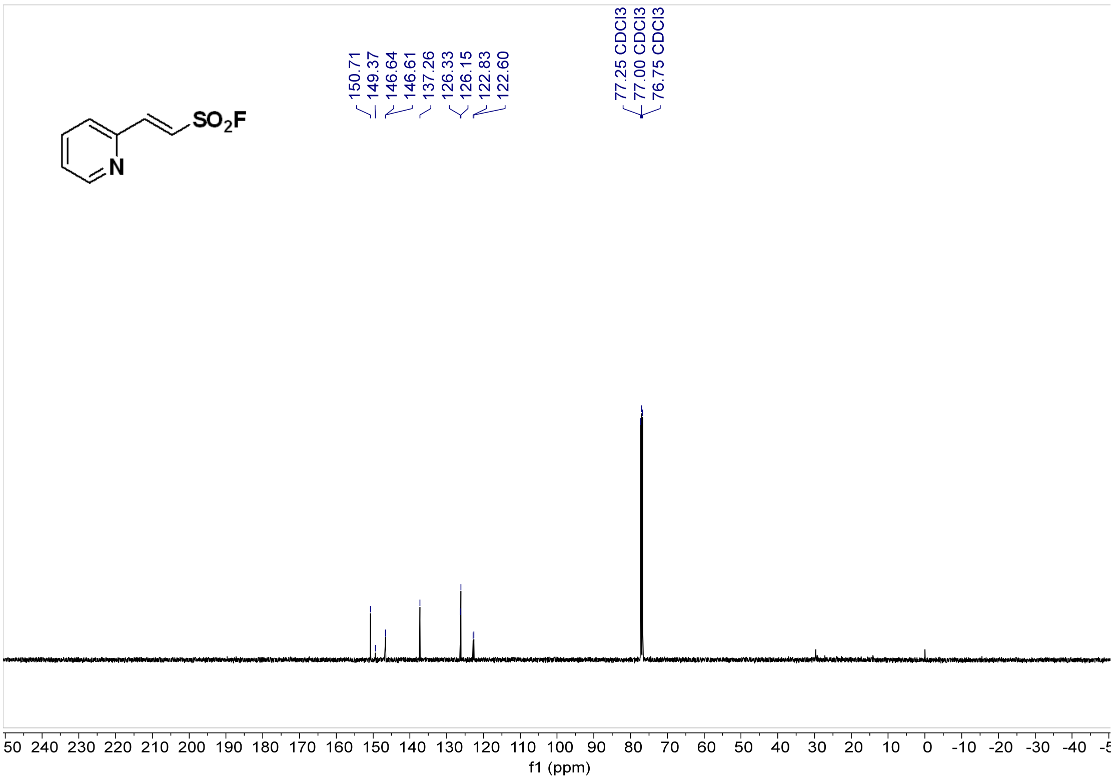

Supplementary Figure 98. ^{13}C NMR (126 MHz, room temperature, $CDCl_3$) spectra of product **3u**

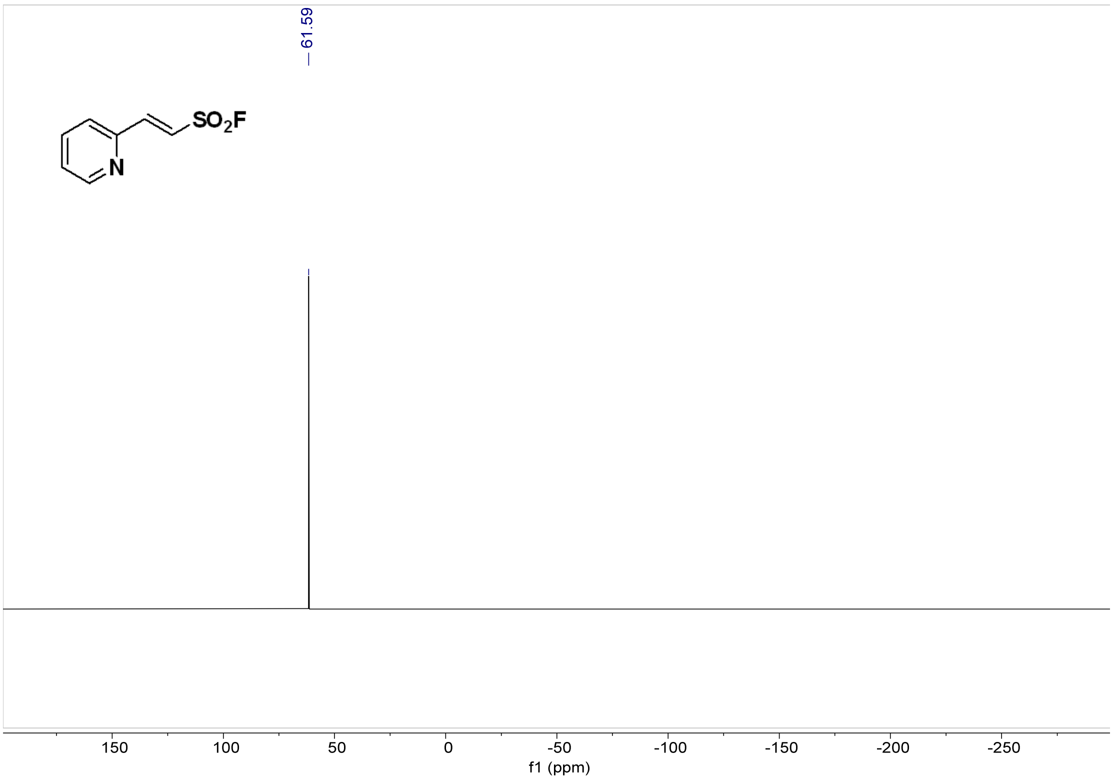

Supplementary Figure 99. ^{19}F NMR (471 MHz, room temperature, $CDCl_3$) spectra of product **3u**

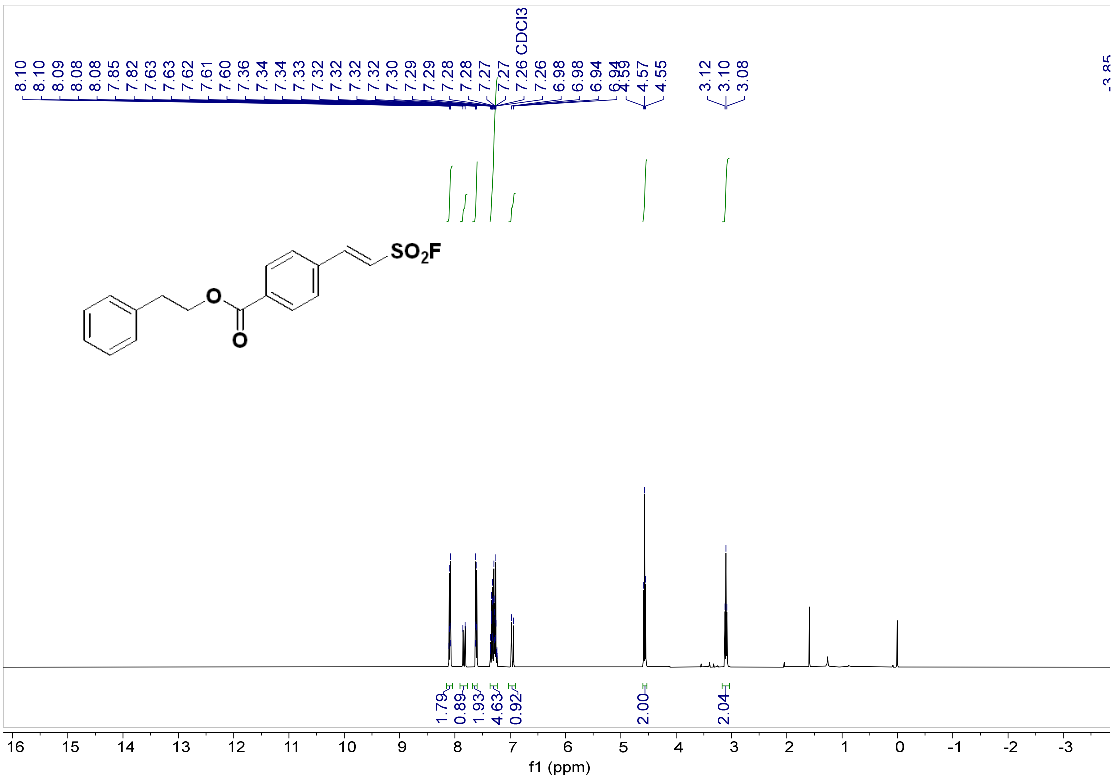

Supplementary Figure 100. ^{1}H NMR (400 MHz, room temperature, $CDCl_3$) spectra of product **3v**

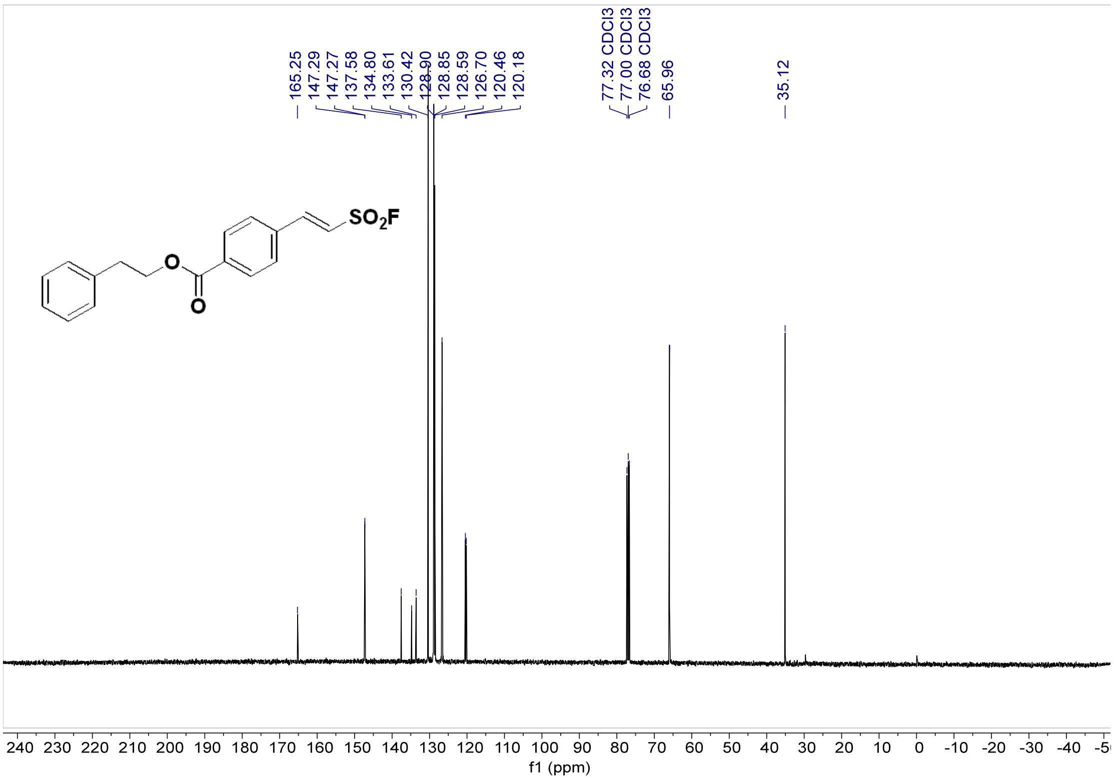

Supplementary Figure 101. ^{13}C NMR (101 MHz, room temperature, $CDCl_3$) spectra of product **3v**

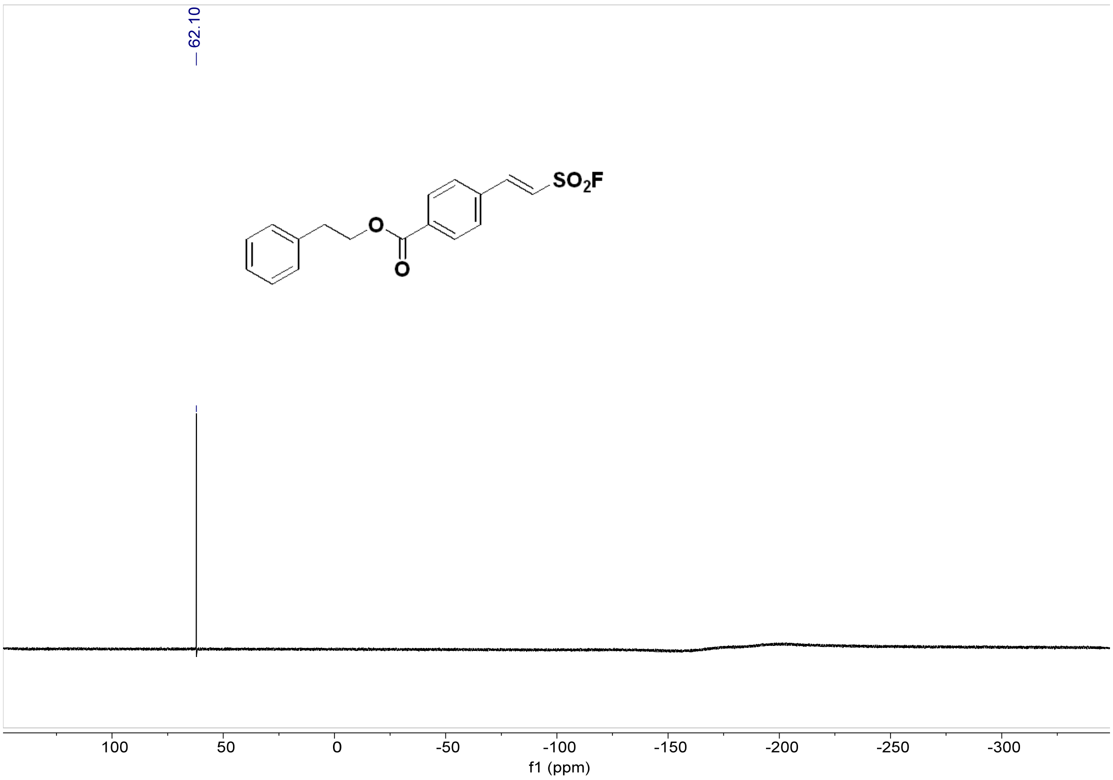

Supplementary Figure 102. ^{19}F NMR (376 MHz, room temperature, $CDCl_3$) spectra of product **3v**

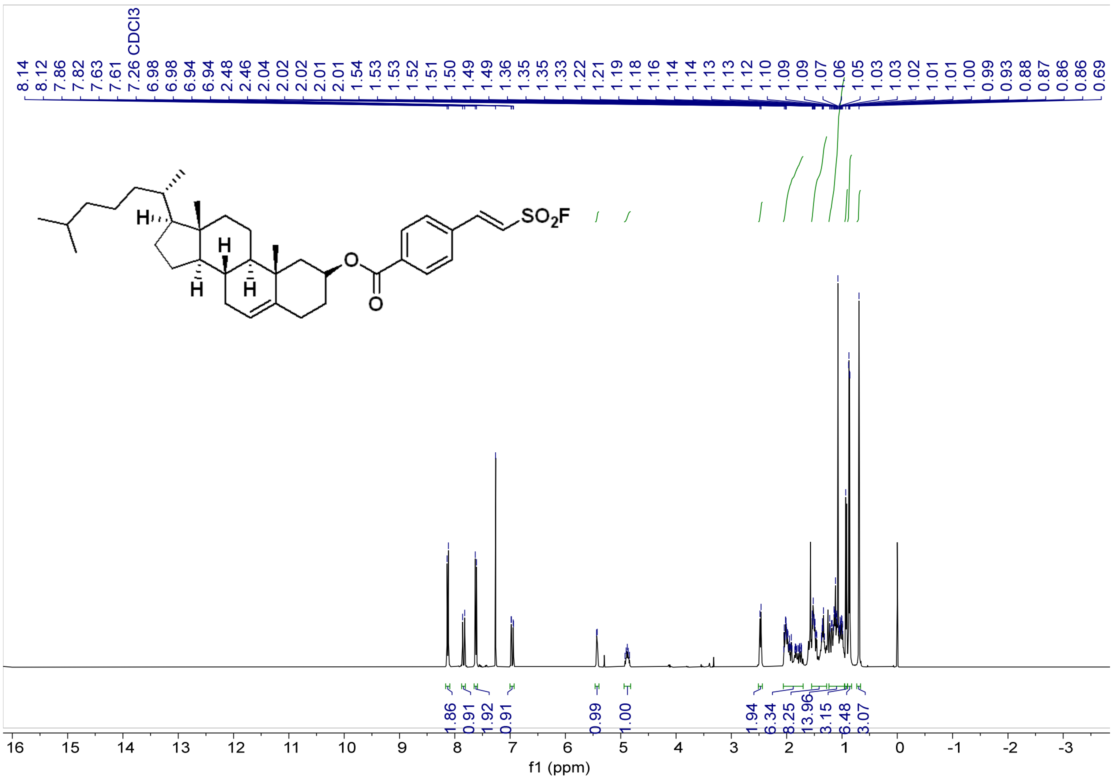

Supplementary Figure 103. ^{1}H NMR (400 MHz, room temperature, $CDCl_3$) spectra of product **3w**

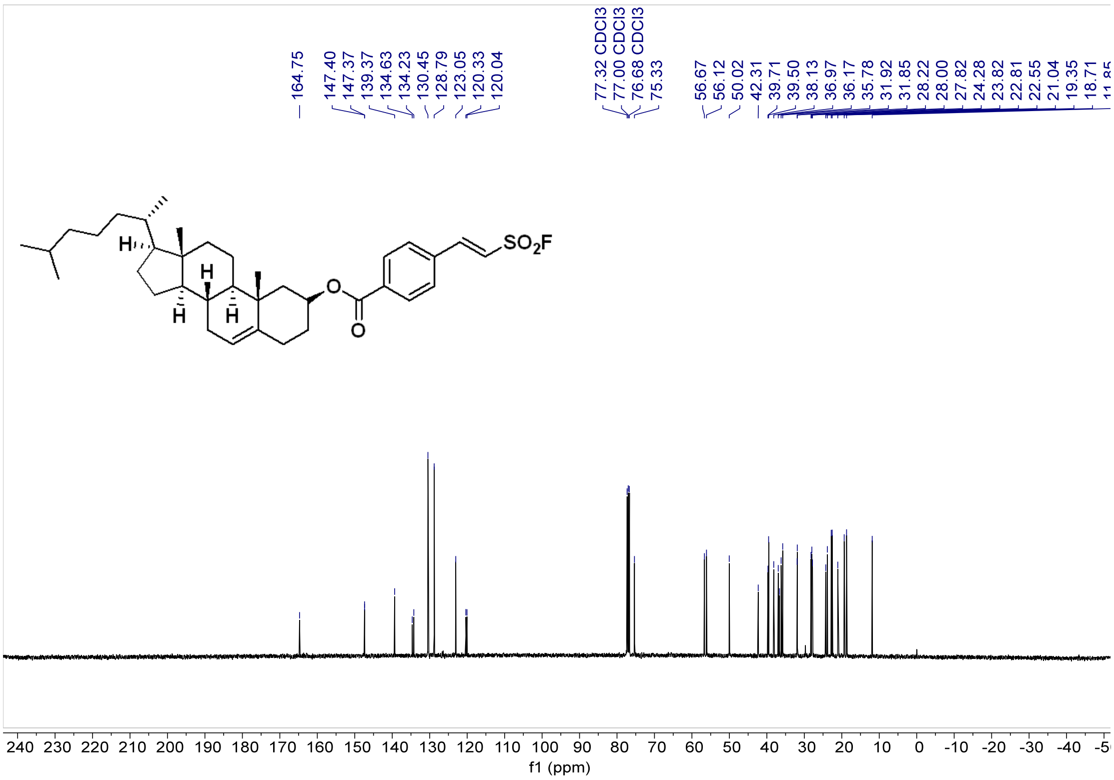

Supplementary Figure 104. ^{13}C NMR (101 MHz, room temperature, $CDCl_3$) spectra of product **3w**

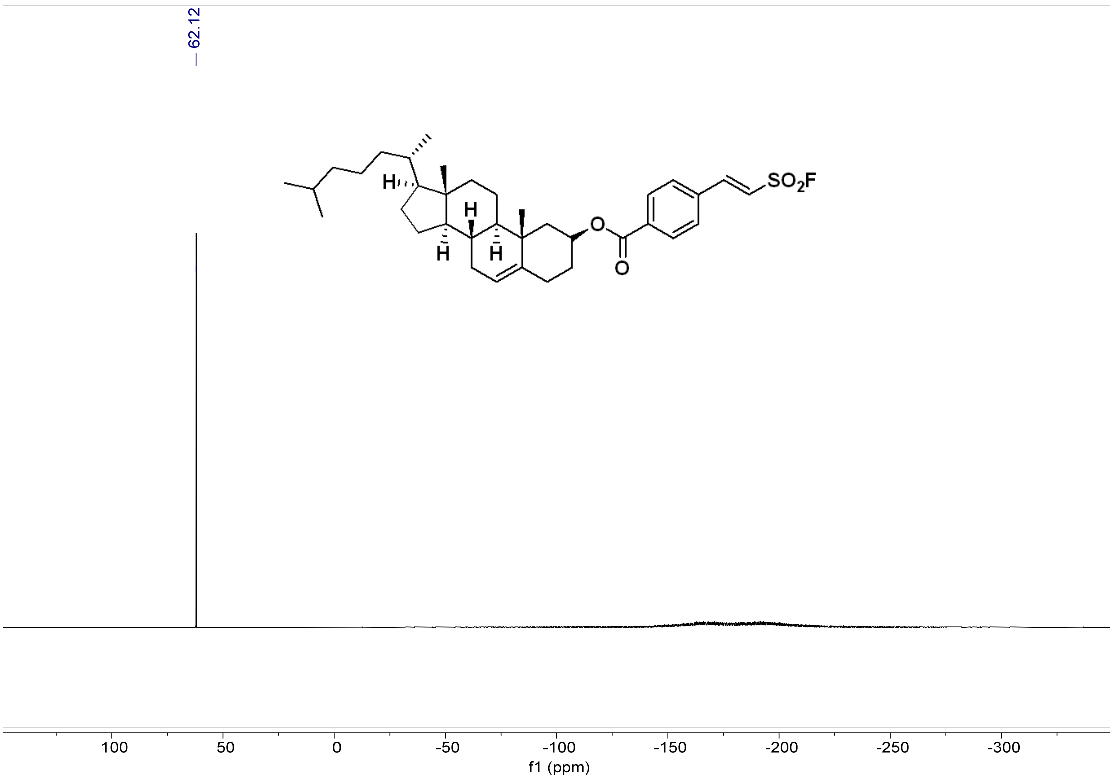

Supplementary Figure 105. ^{19}F NMR (376 MHz, room temperature, $CDCl_3$) spectra of product **3w**

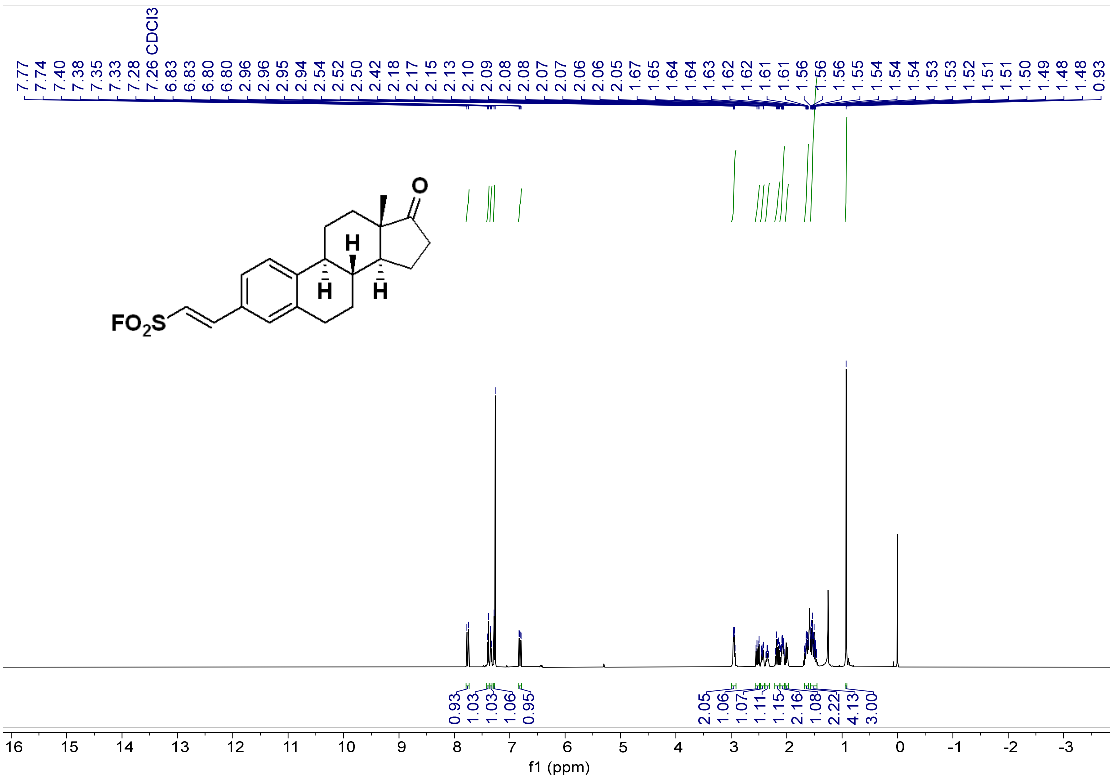

Supplementary Figure 106. ^{1}H NMR (500 MHz, room temperature, $CDCl_3$) spectra of product **3x**

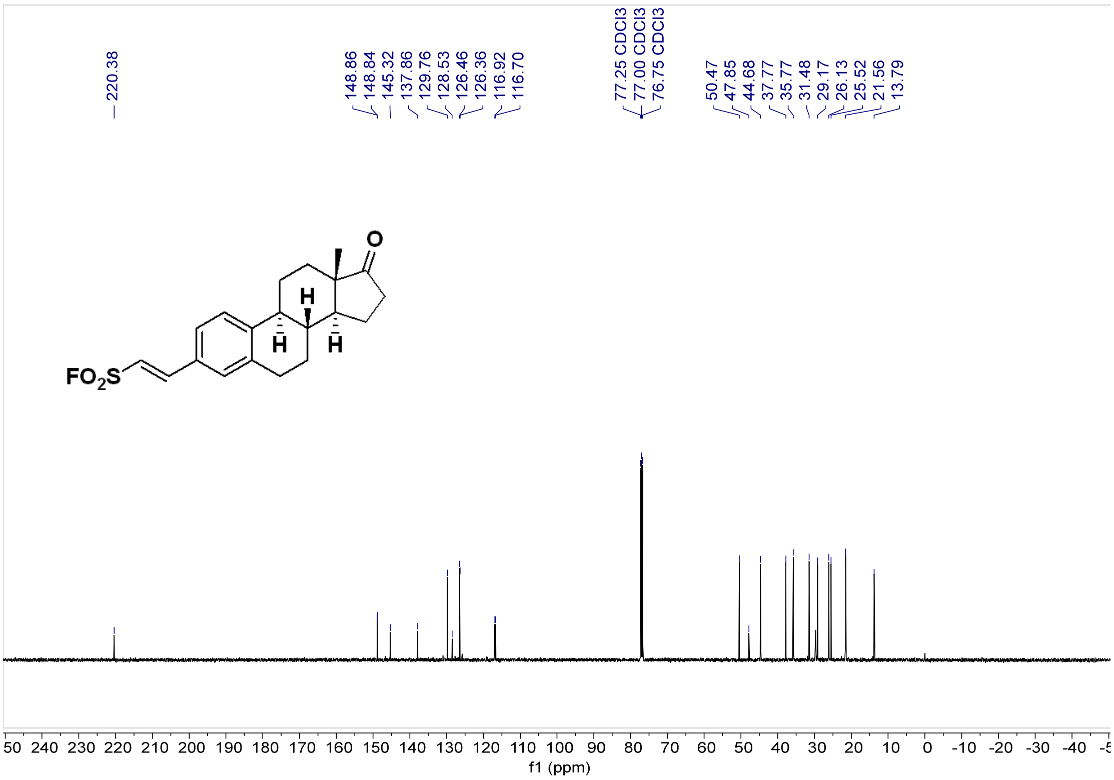

Supplementary Figure 107. ^{13}C NMR (126 MHz, room temperature, $CDCl_3$) spectra of product **3x**

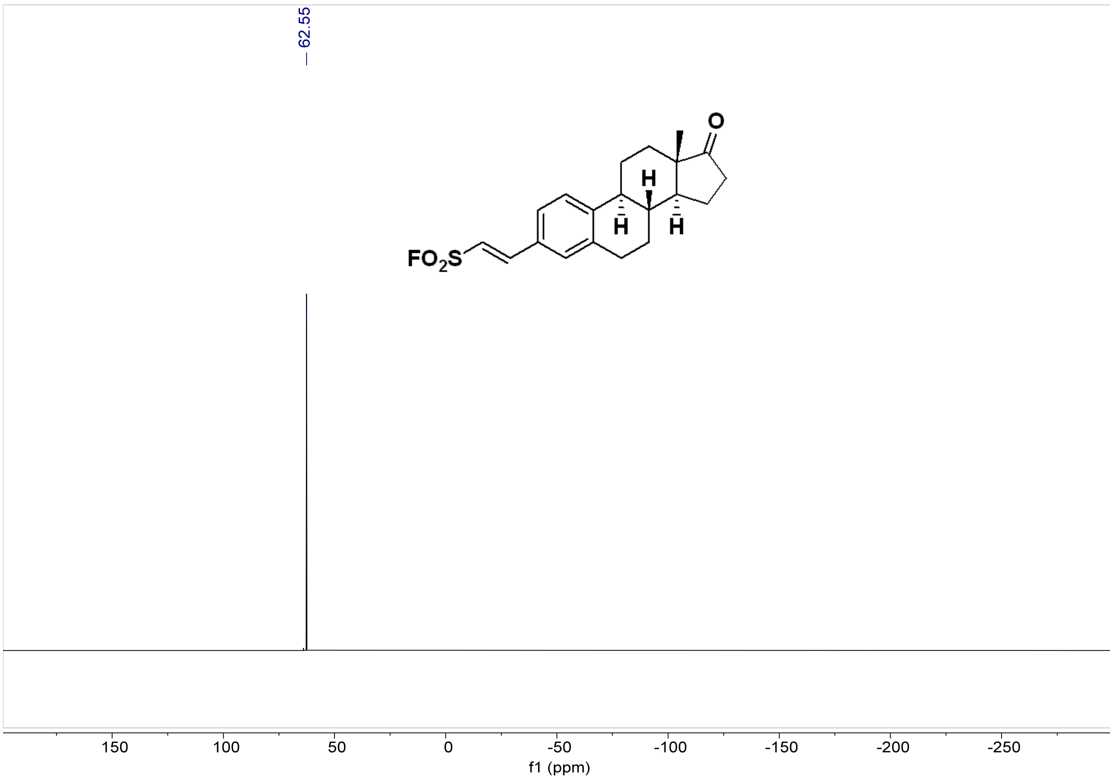

Supplementary Figure 108. ^{19}F NMR (471 MHz, room temperature, $CDCl_3$) spectra of product **3x**

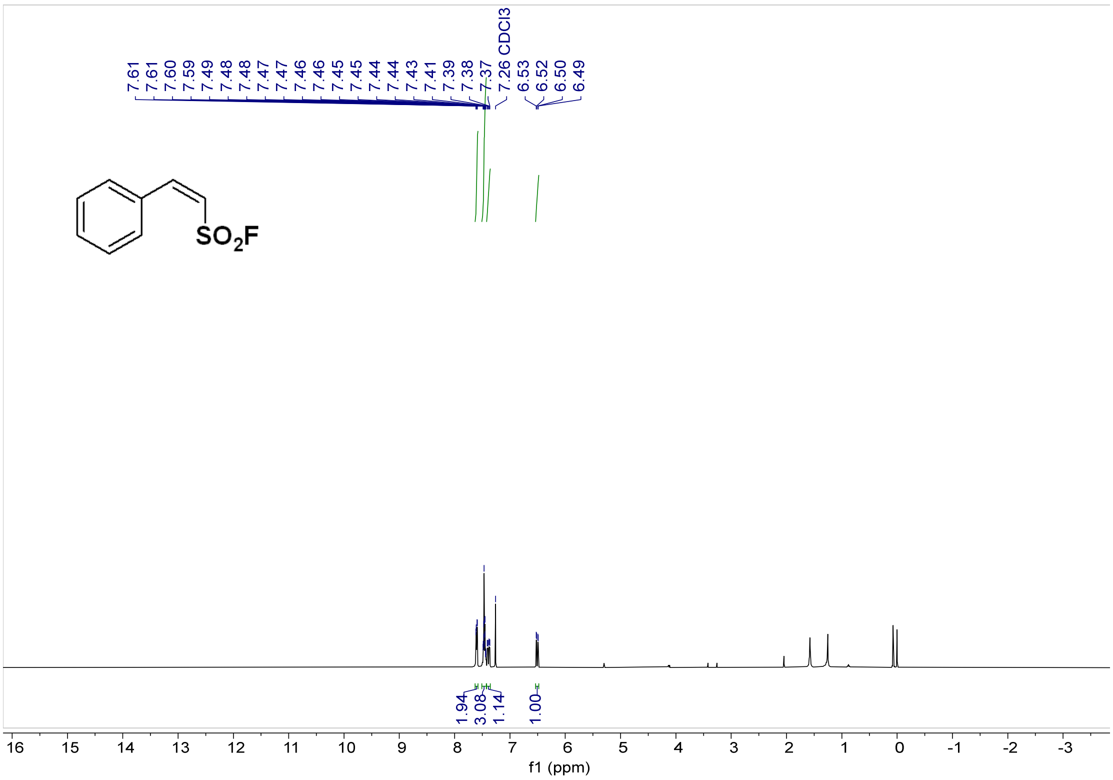

Supplementary Figure 109. ^{1}H NMR (400 MHz, room temperature, $CDCl_3$) spectra of product **4a**

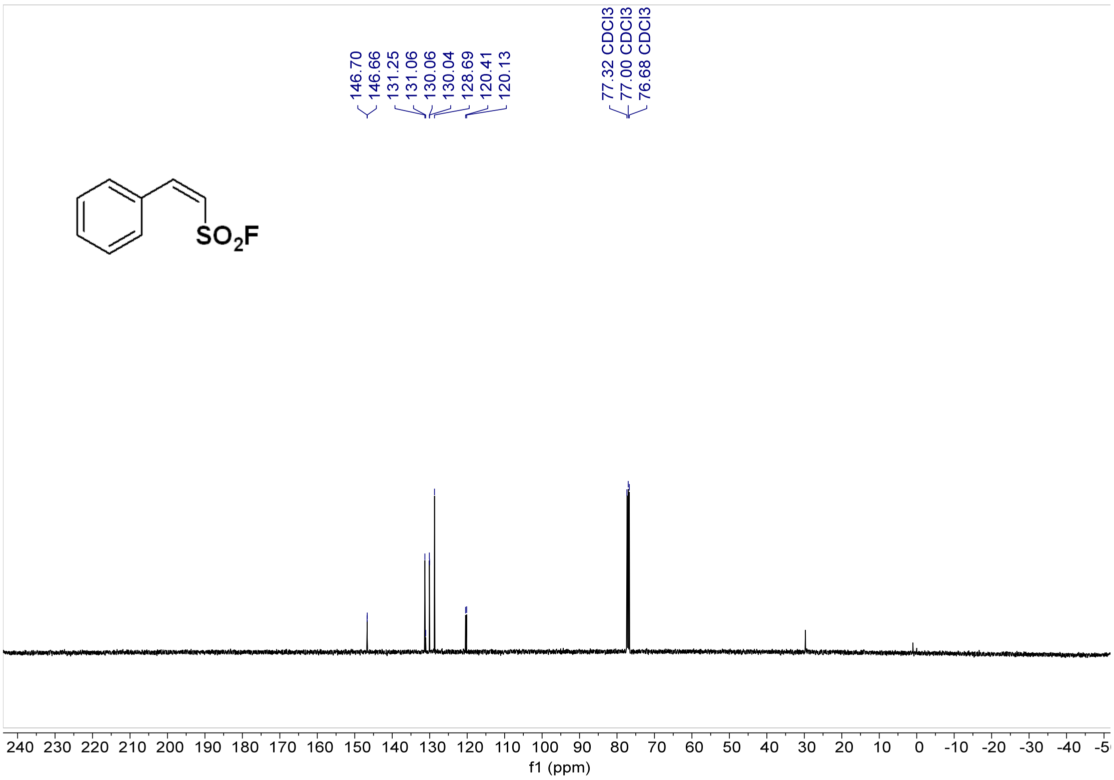

Supplementary Figure 110. ^{13}C NMR (101 MHz, room temperature, $CDCl_3$) spectra of product **4a**

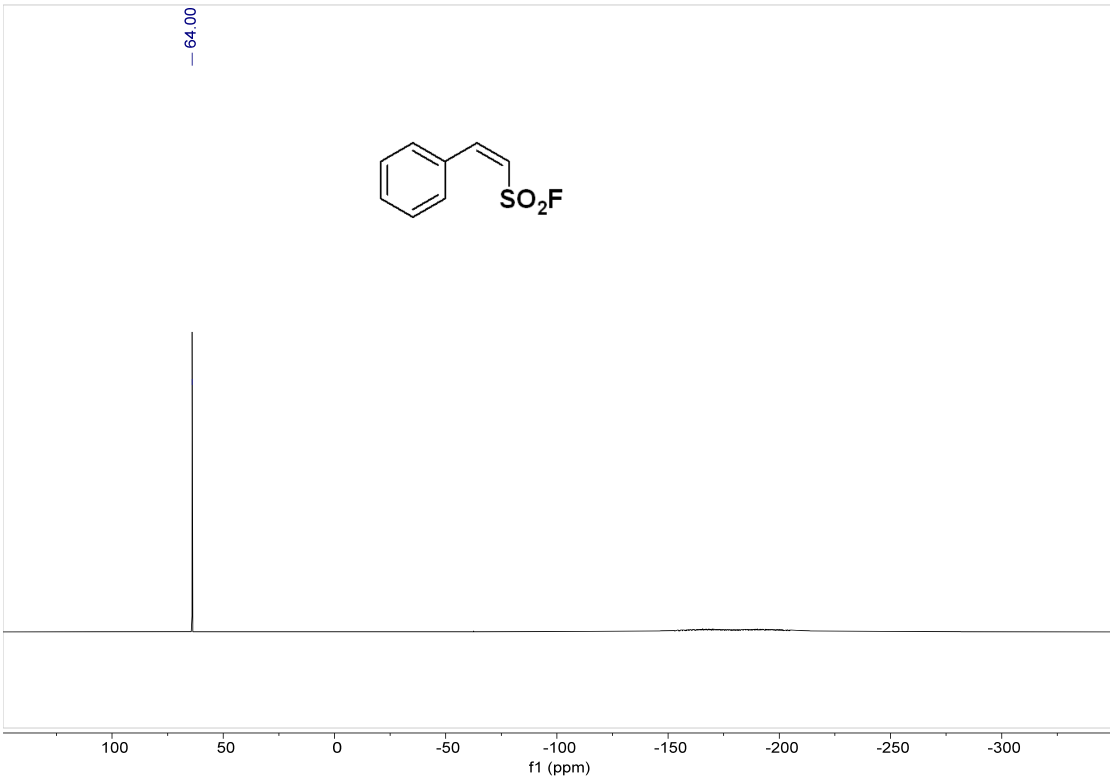

Supplementary Figure 111. ^{19}F NMR (376 MHz, room temperature, $CDCl_3$) spectra of product **4a**

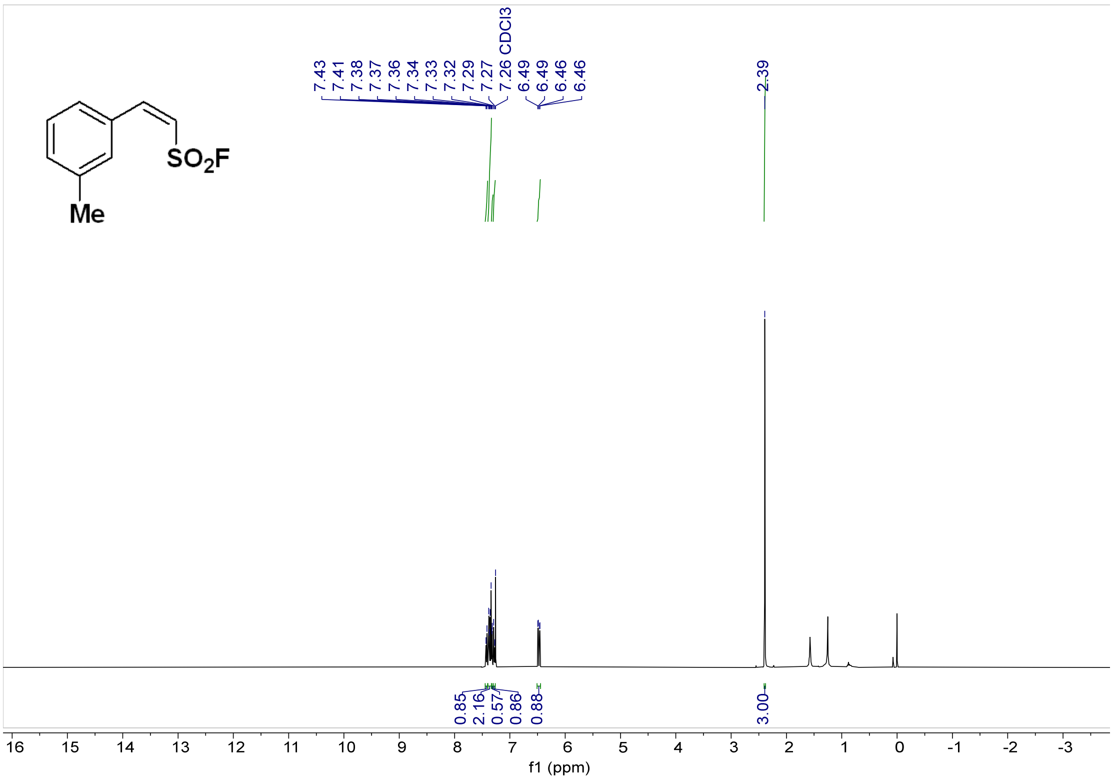

Supplementary Figure 112. ^{1}H NMR (400 MHz, room temperature, $CDCl_3$) spectra of product **4b**

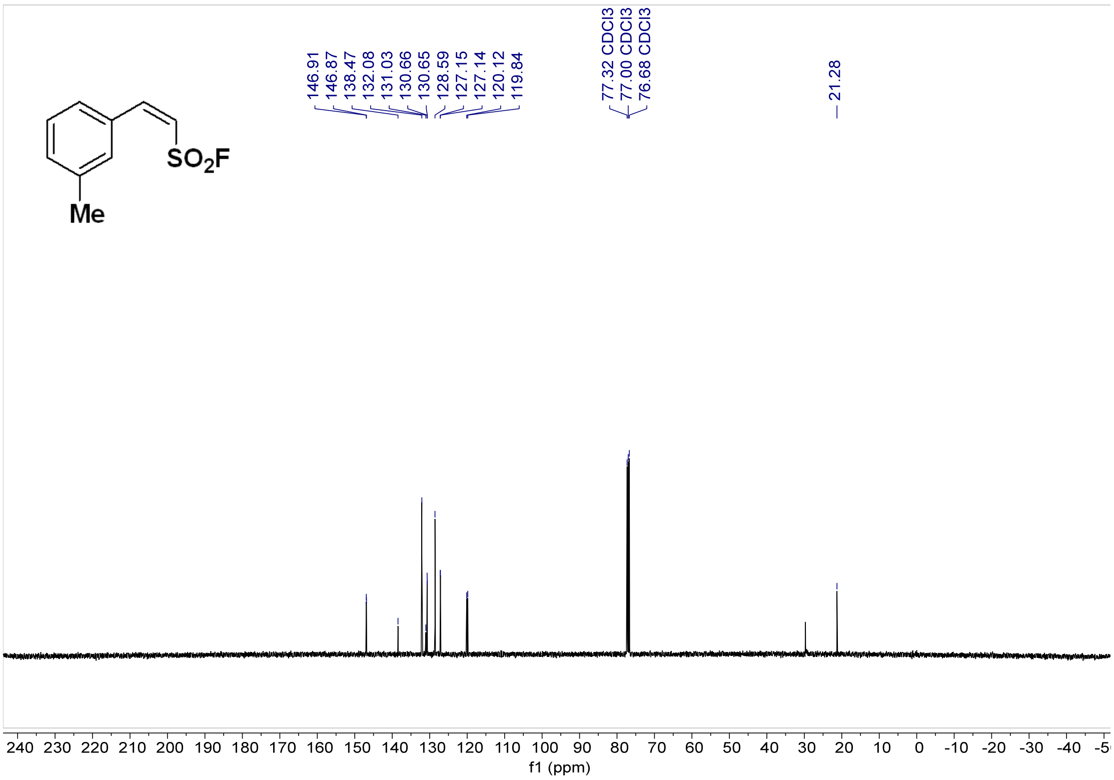

Supplementary Figure 113. ^{13}C NMR (101 MHz, room temperature, $CDCl_3$) spectra of product **4b**

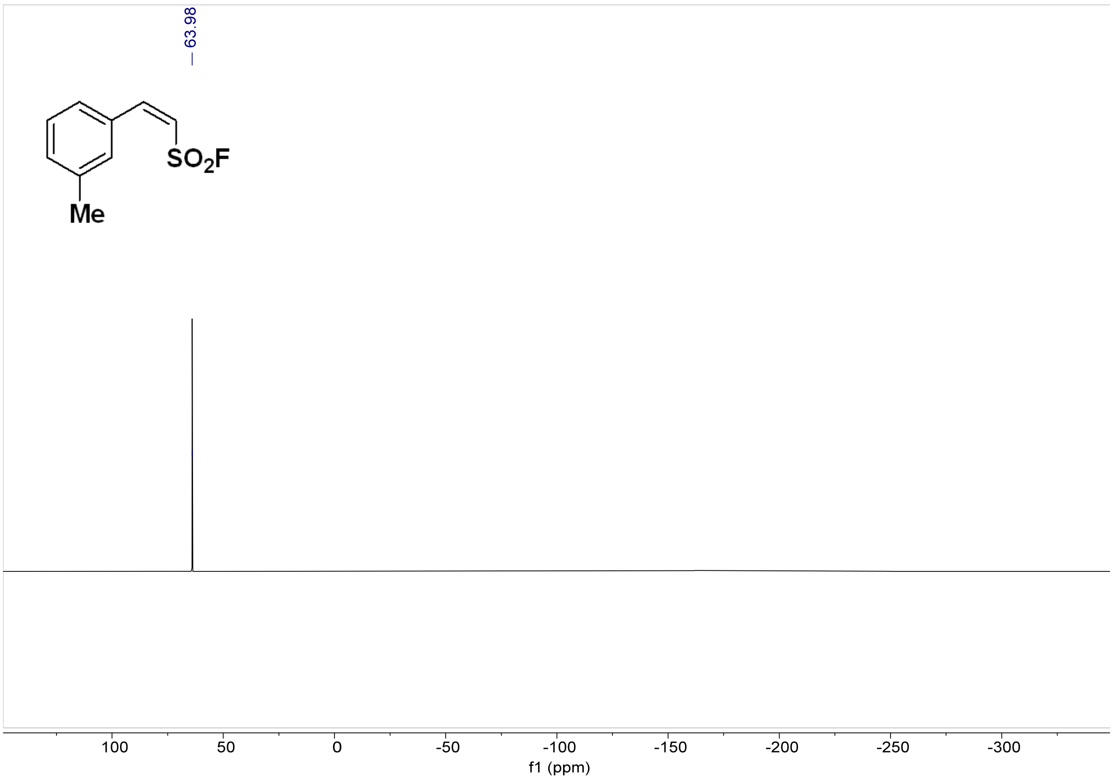

Supplementary Figure 114. ^{19}F NMR (376 MHz, room temperature, $CDCl_3$) spectra of product **4b**

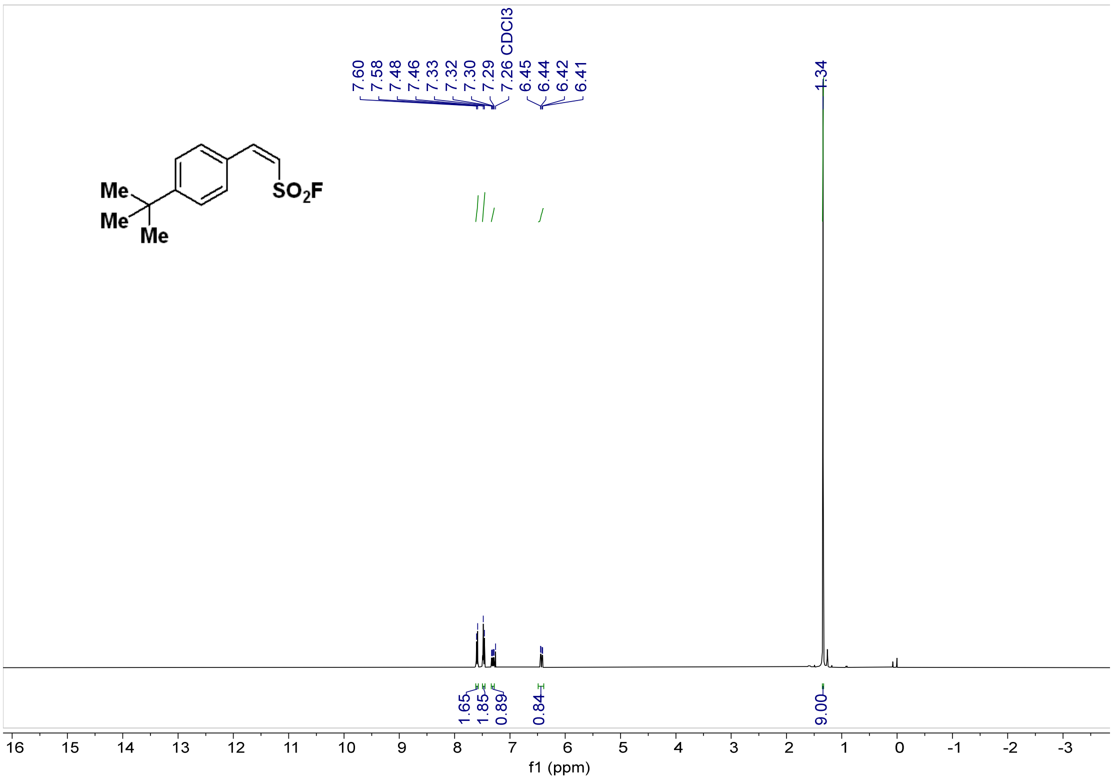

Supplementary Figure 115. ^{1}H NMR (400 MHz, room temperature, $CDCl_3$) spectra of product **4c**

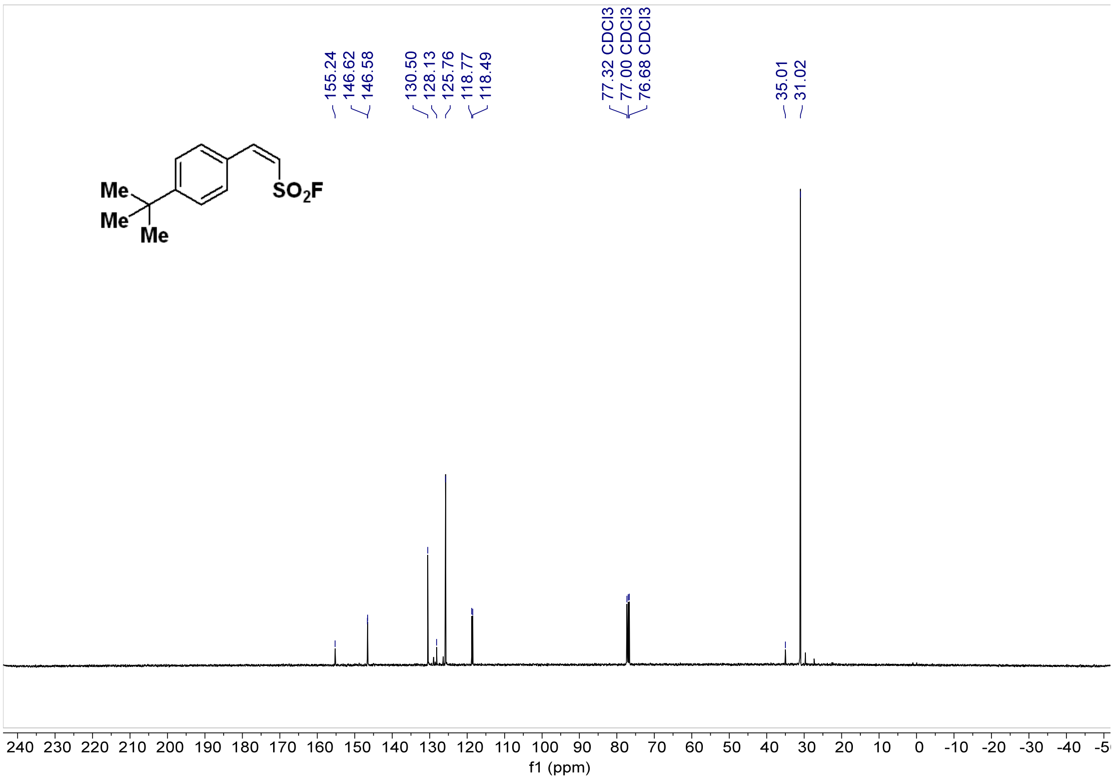

Supplementary Figure 116. ^{13}C NMR (101 MHz, room temperature, $CDCl_3$) spectra of product **4c**

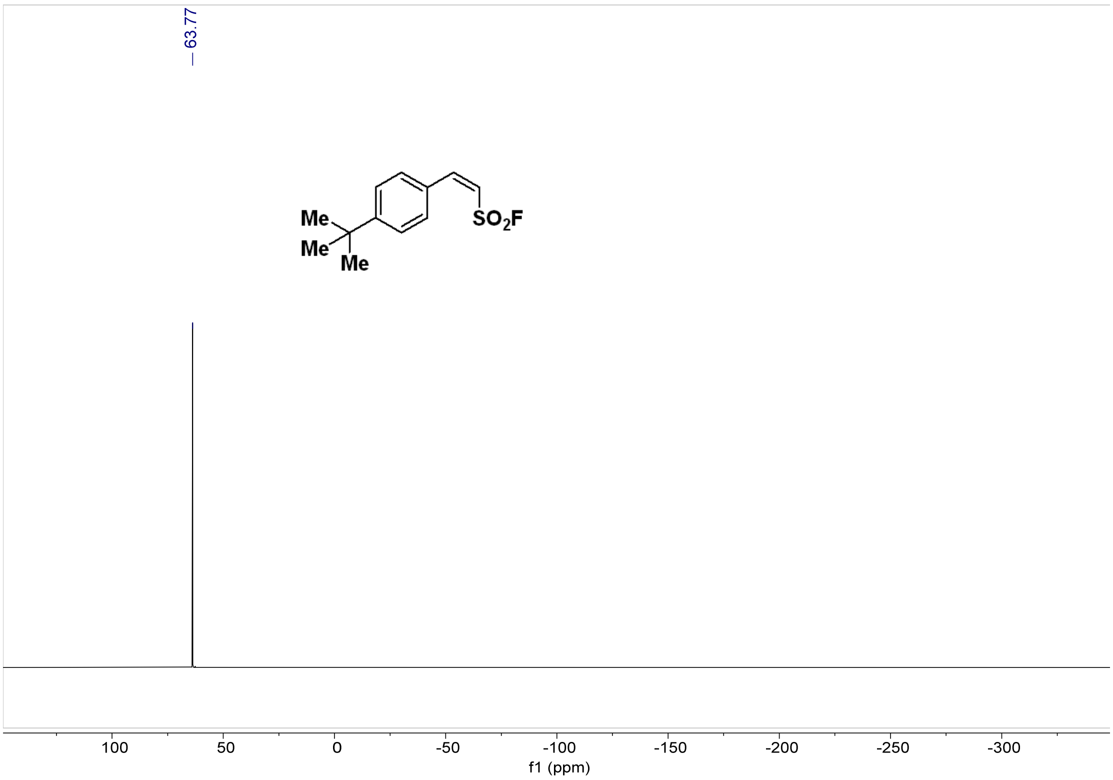

Supplementary Figure 117. ^{19}F NMR (376 MHz, room temperature, $CDCl_3$) spectra of product **4c**

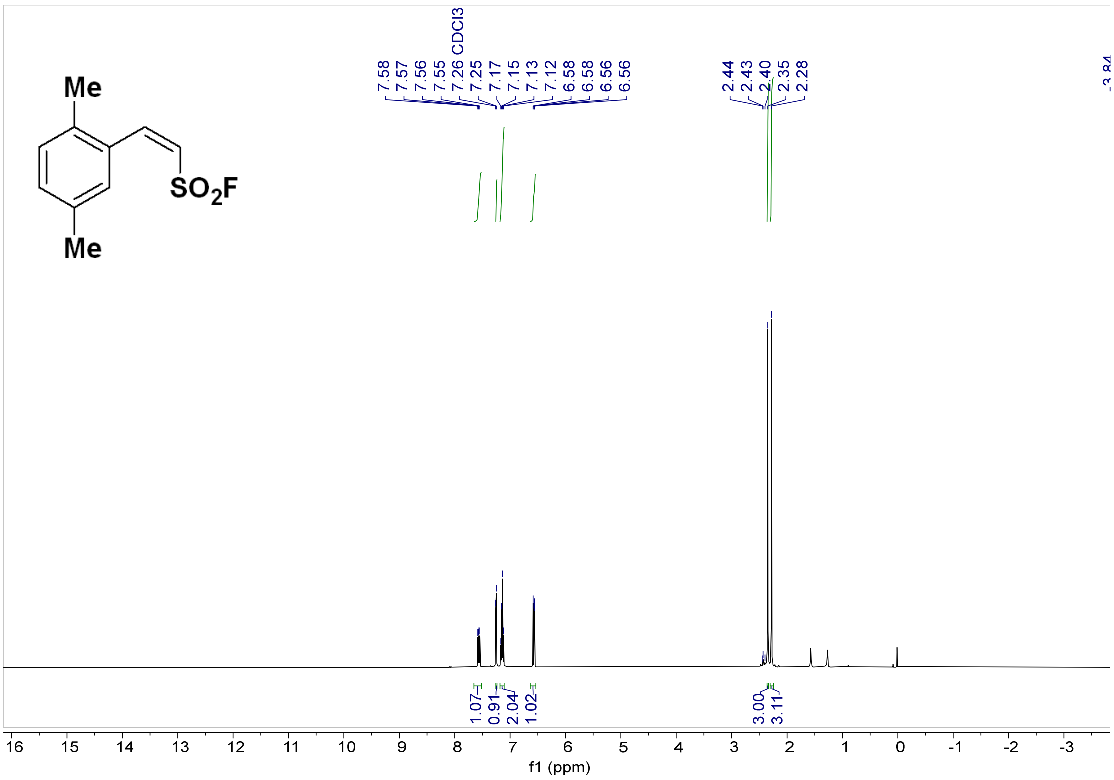

Supplementary Figure 118. ^{1}H NMR (500 MHz, room temperature, $CDCl_3$) spectra of product **4d**

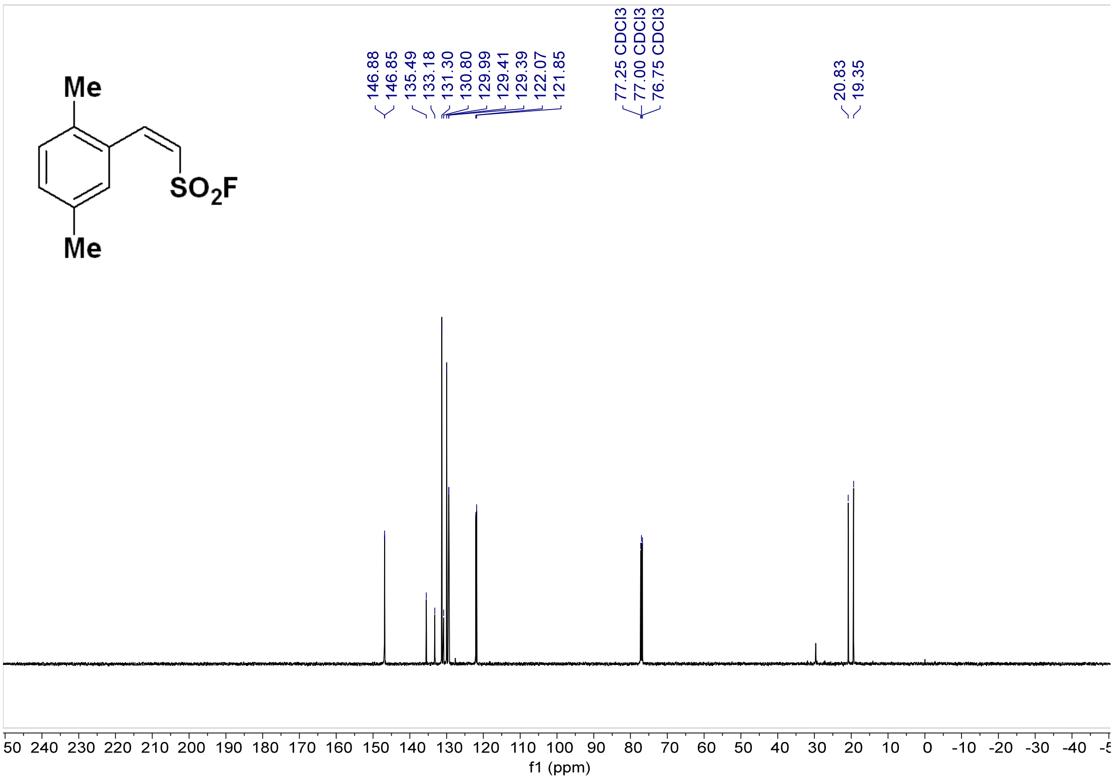

Supplementary Figure 119. ^{13}C NMR (126 MHz, room temperature, $CDCl_3$) spectra of product **4d**

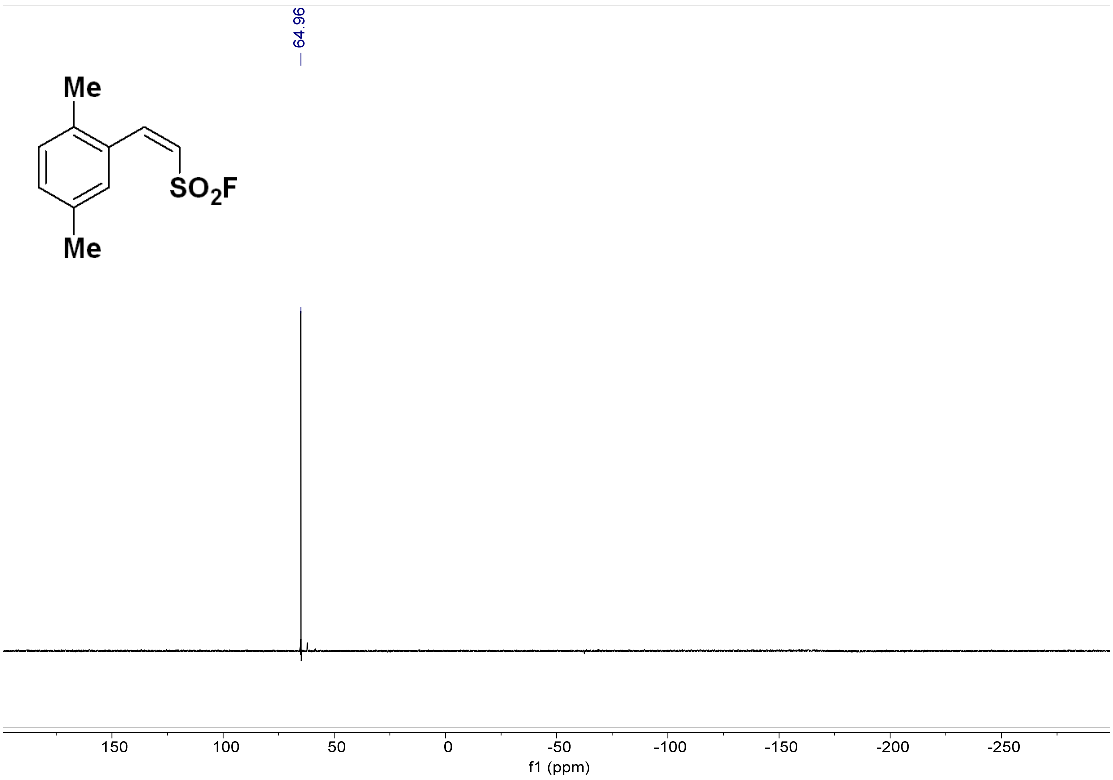

Supplementary Figure 120. ^{19}F NMR (471 MHz, room temperature, $CDCl_3$) spectra of product **4d**

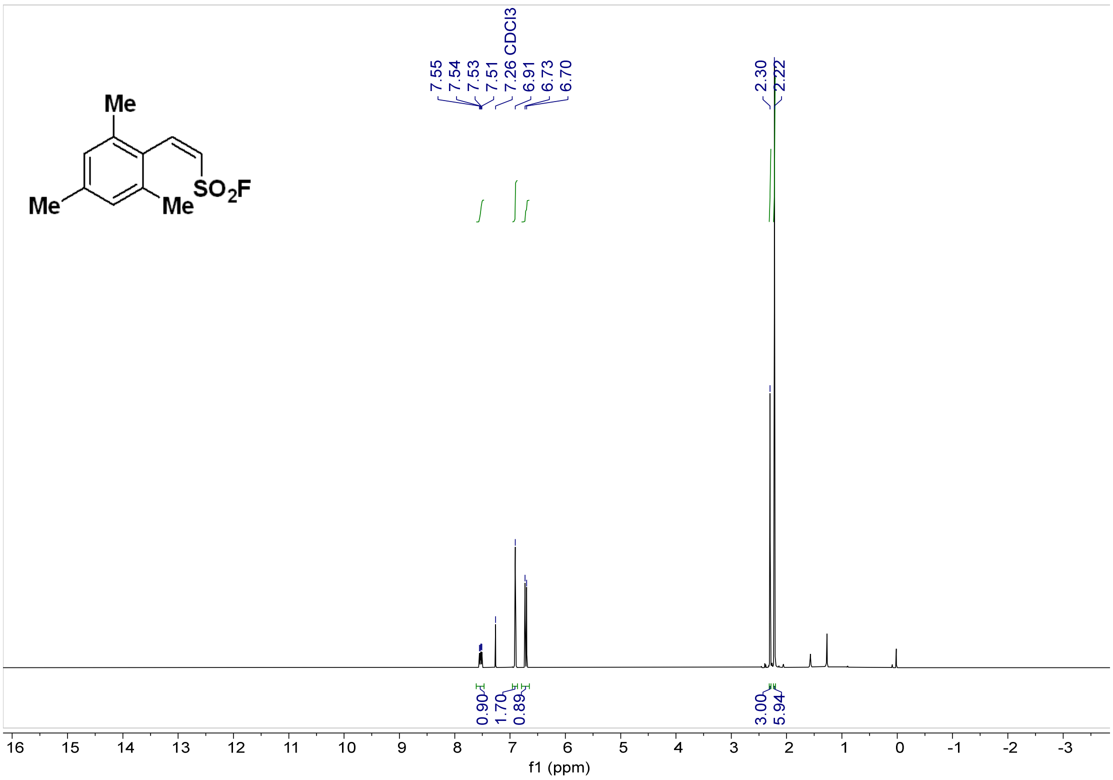

Supplementary Figure 121. ^{1}H NMR (400 MHz, room temperature, $CDCl_3$) spectra of product **4e**

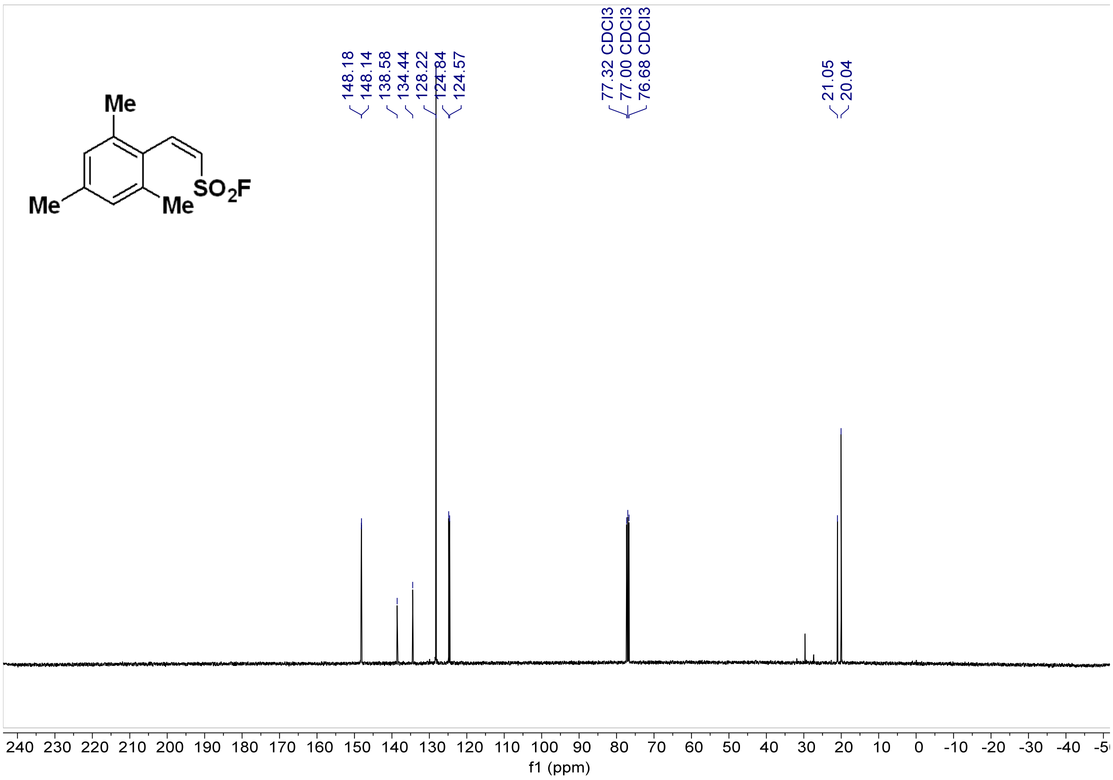

Supplementary Figure 122. ^{13}C NMR (101 MHz, room temperature, $CDCl_3$) spectra of product **4e**

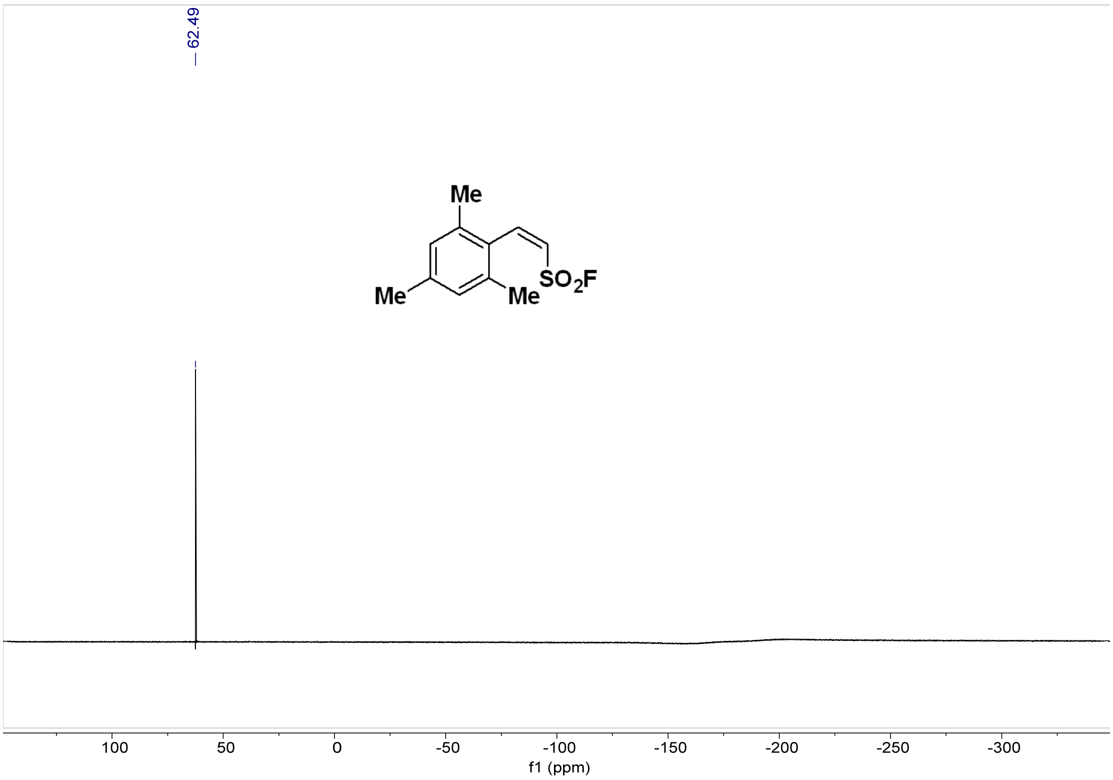

Supplementary Figure 123. ^{19}F NMR (376 MHz, room temperature, $CDCl_3$) spectra of product **4e**

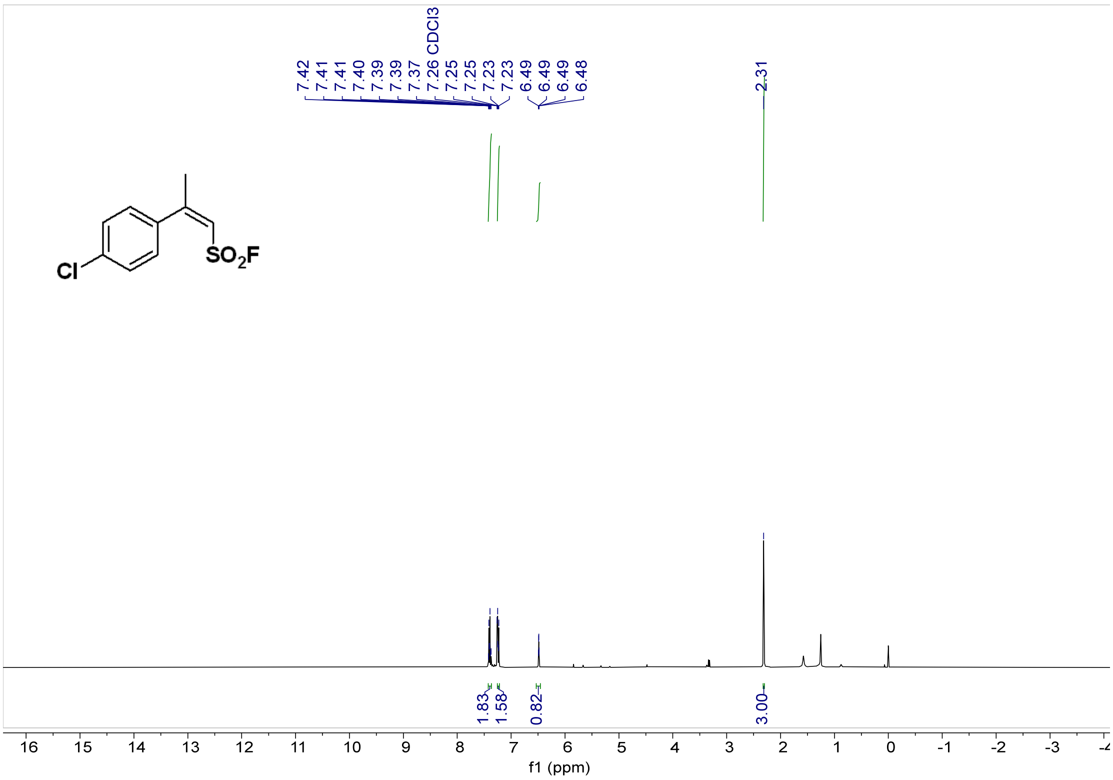

Supplementary Figure 124. ^1H NMR (400 MHz, room temperature, $CDCl_3$) spectra of product **4f**

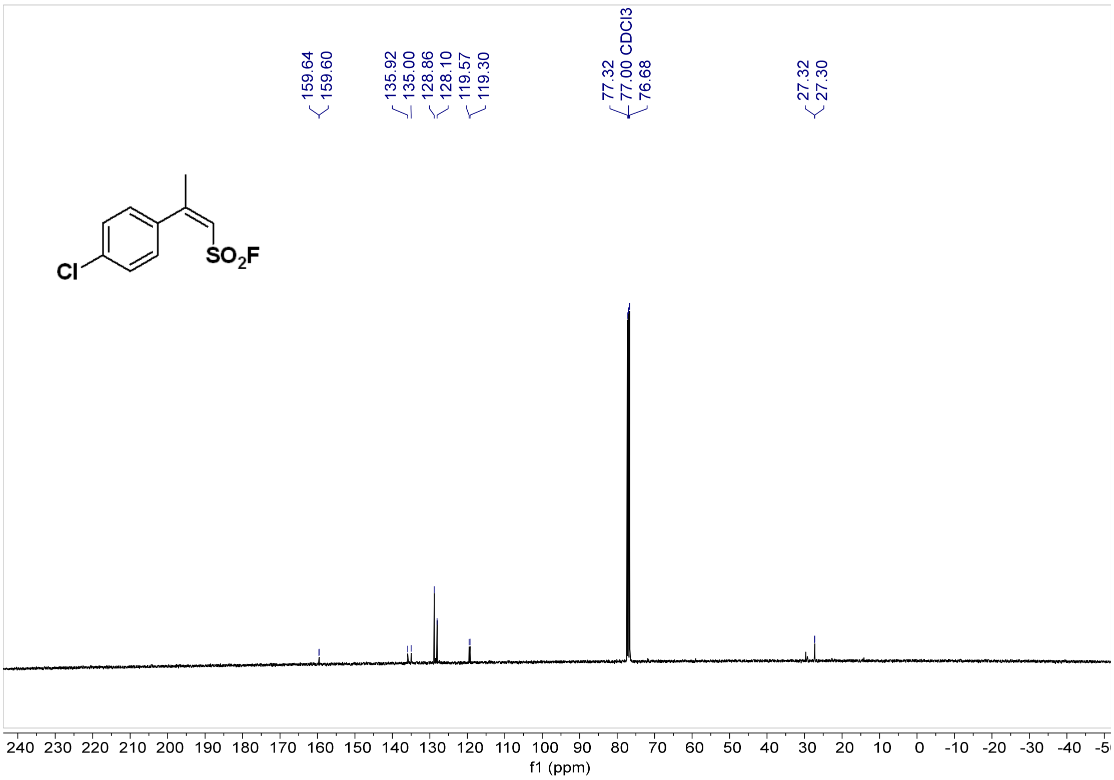

Supplementary Figure 125. ^{13}C NMR (101 MHz, room temperature, $CDCl_3$) spectra of product **4f**

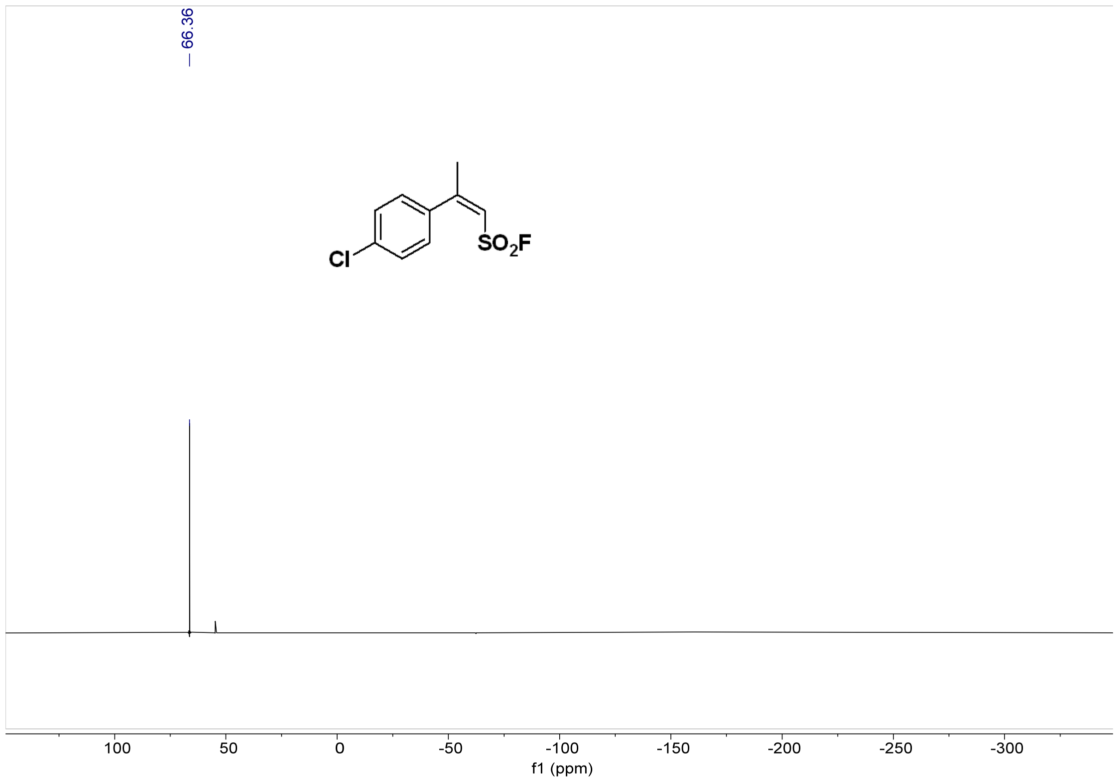

Supplementary Figure 126. ^{19}F NMR (376 MHz, room temperature, $CDCl_3$) spectra of product **4f**

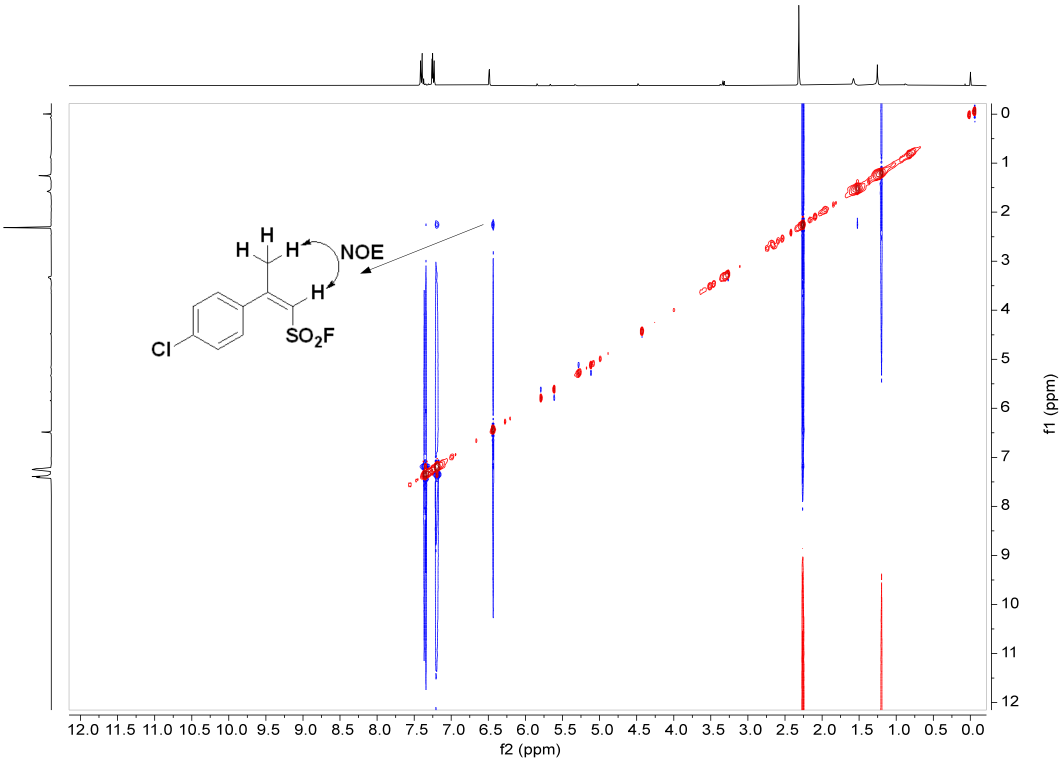

Supplementary Figure 127. NOESY (400 MHz, room temperature, $CDCl_3$) spectra of product **4f**

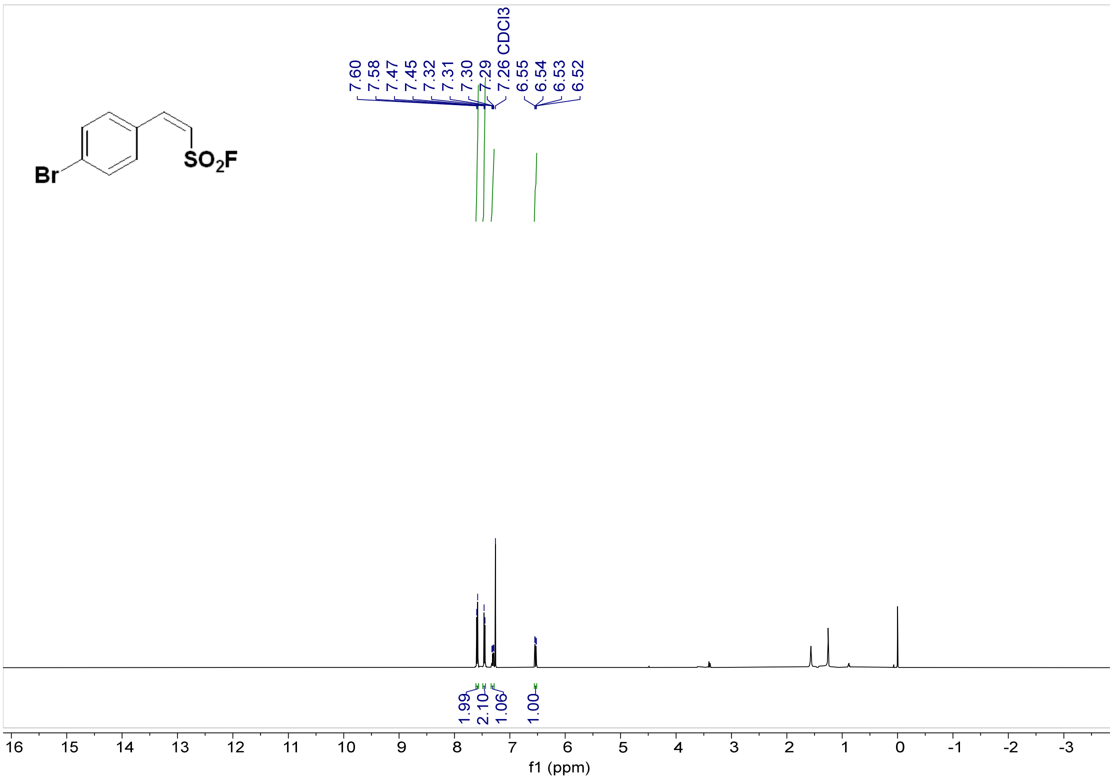

Supplementary Figure 128. ^{1}H NMR (500 MHz, room temperature, $CDCl_3$) spectra of product **4g**

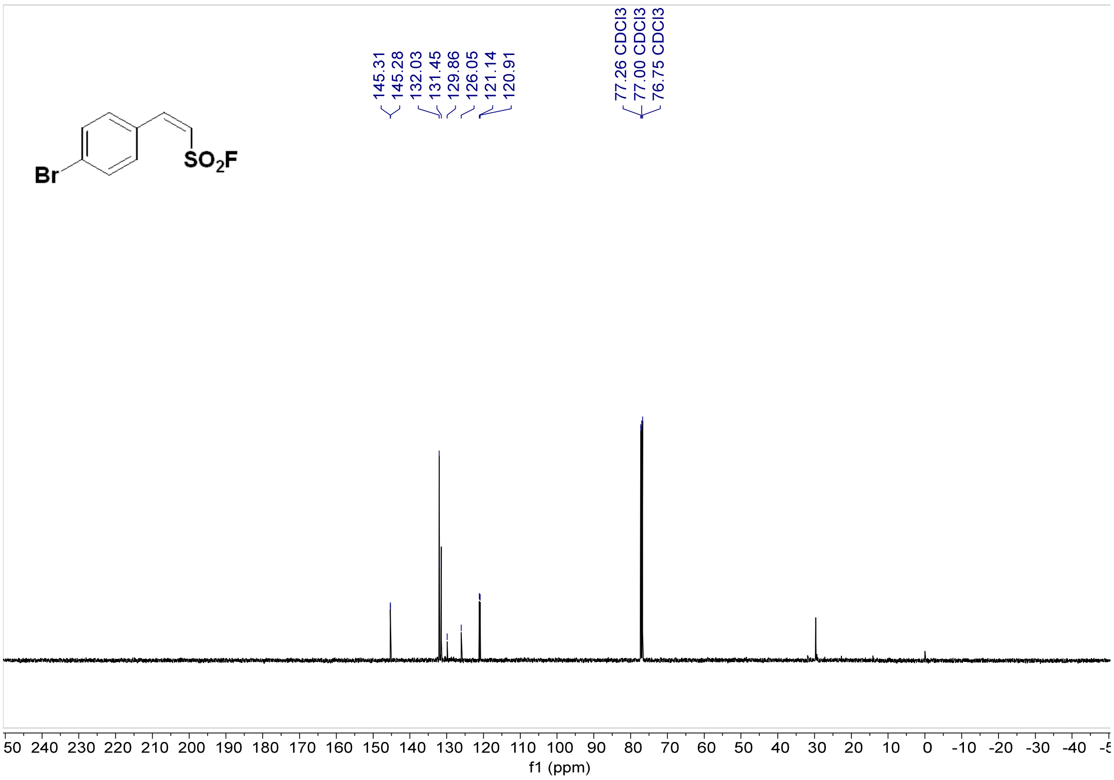

Supplementary Figure 129. ^{19}C NMR (126 MHz, room temperature, $CDCl_3$) spectra of product **4g**

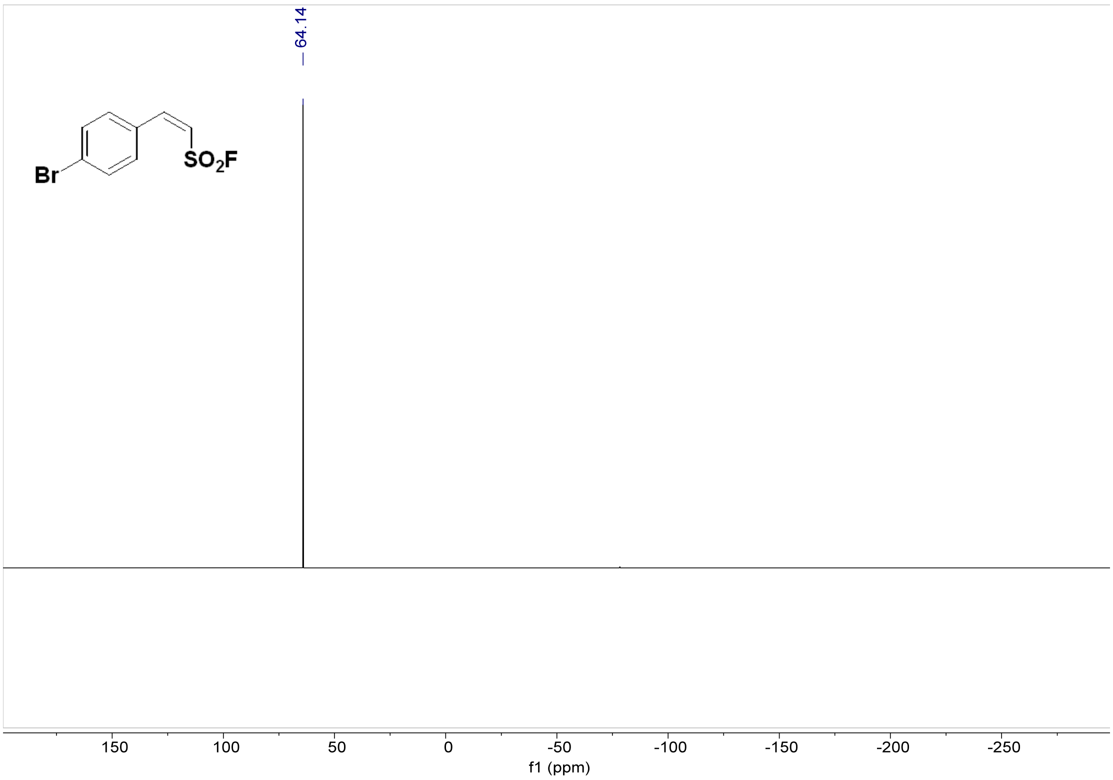

Supplementary Figure 130. ^{19}F NMR (471 MHz, room temperature, $CDCl_3$) spectra of product **4g**

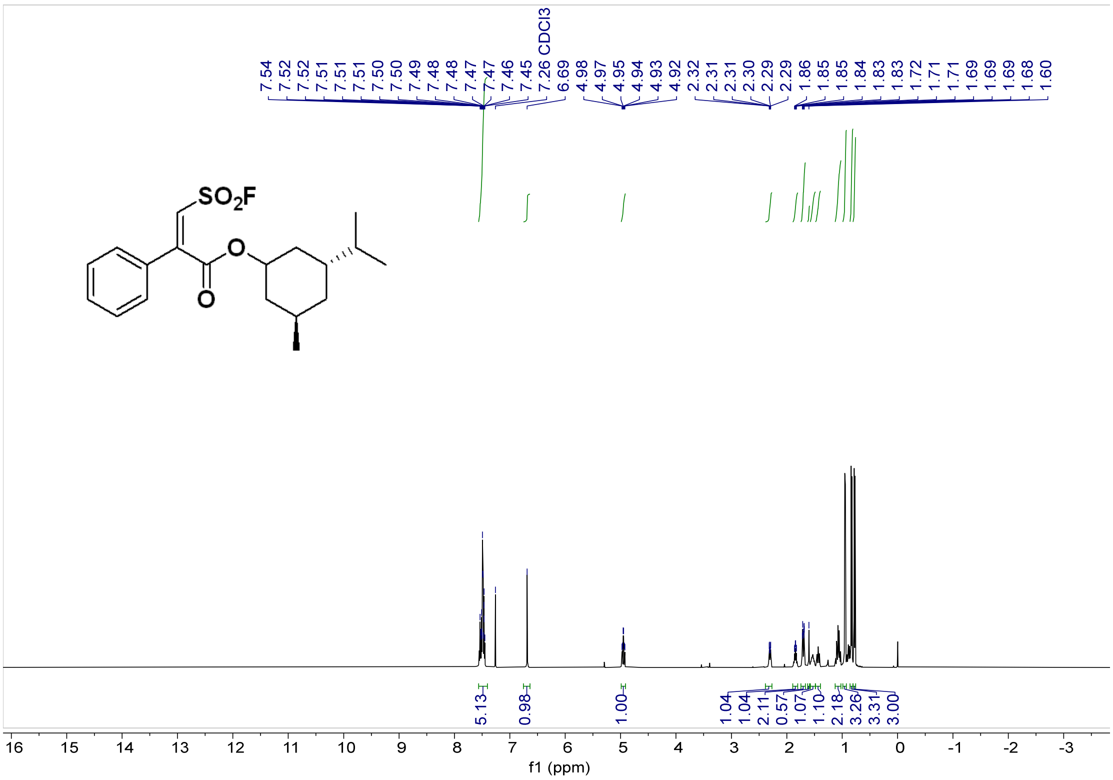

Supplementary Figure 131. ^{1}H NMR (500 MHz, room temperature, $CDCl_3$) spectra of product **4h**

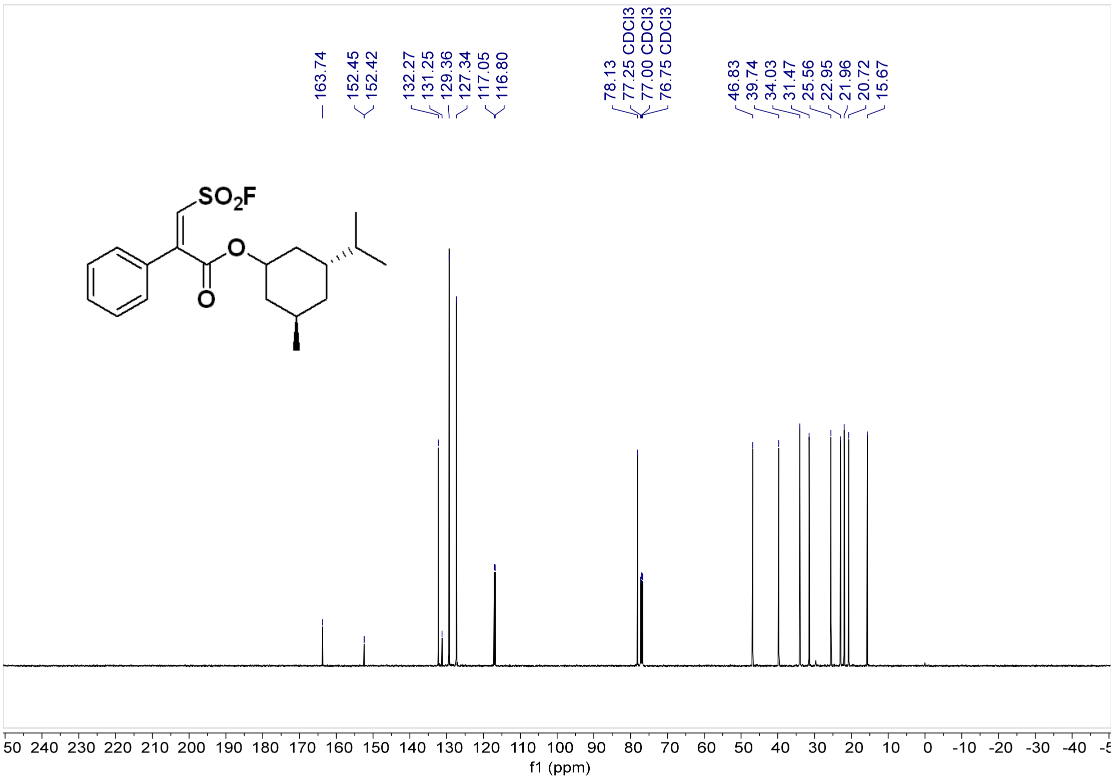

Supplementary Figure 132. ^{13}C NMR (126 MHz, room temperature, $CDCl_3$) spectra of product **4h**

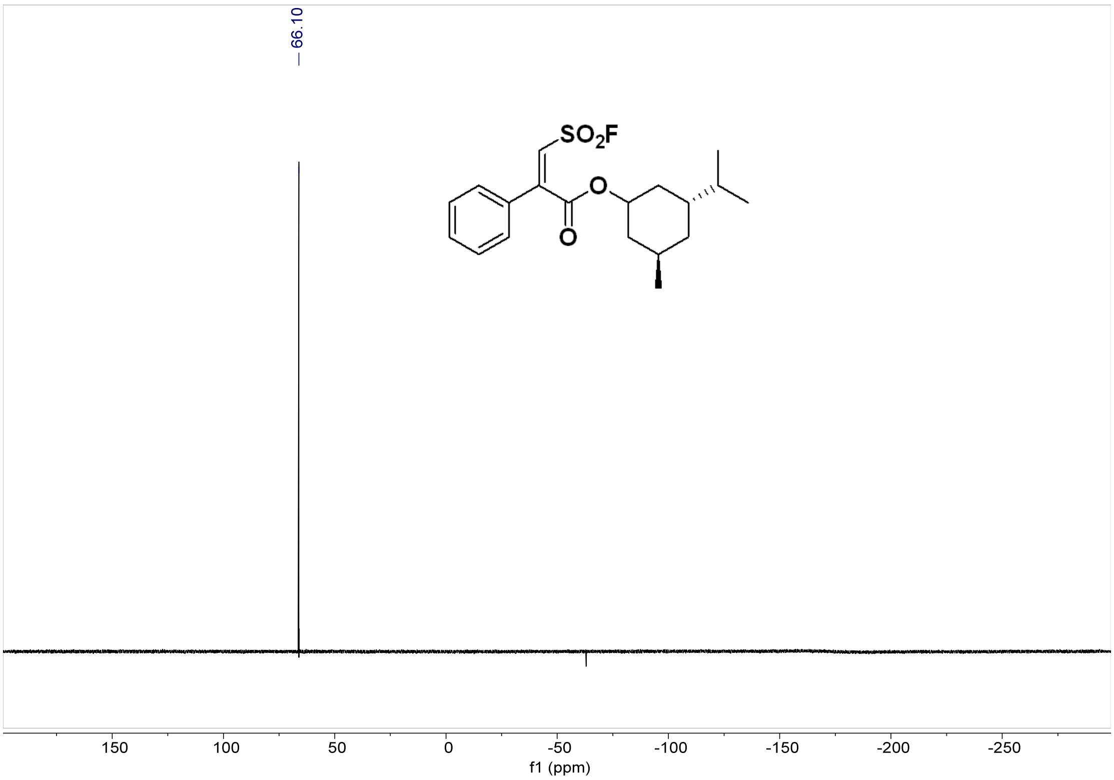

Supplementary Figure 133. ^{19}F NMR (471 MHz, room temperature, $CDCl_3$) spectra of product **4h**

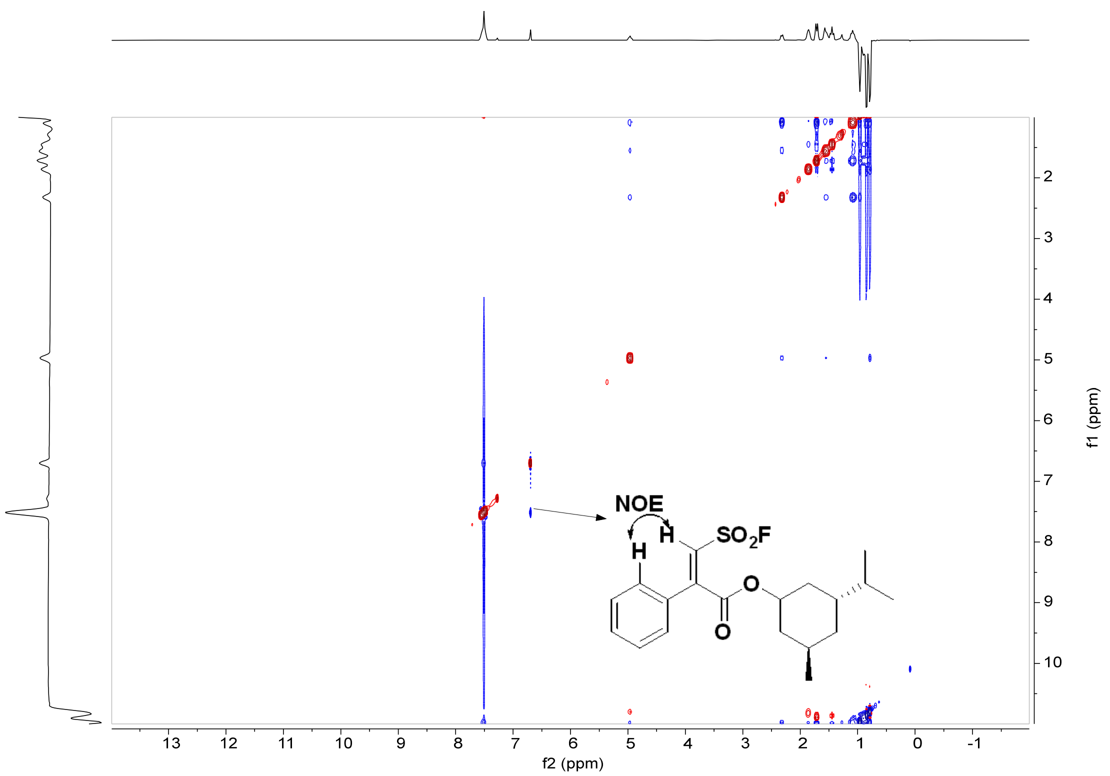

Supplementary Figure 134. NOESY NMR (400 MHz, room temperature, $CDCl_3$) spectra of product **4h**

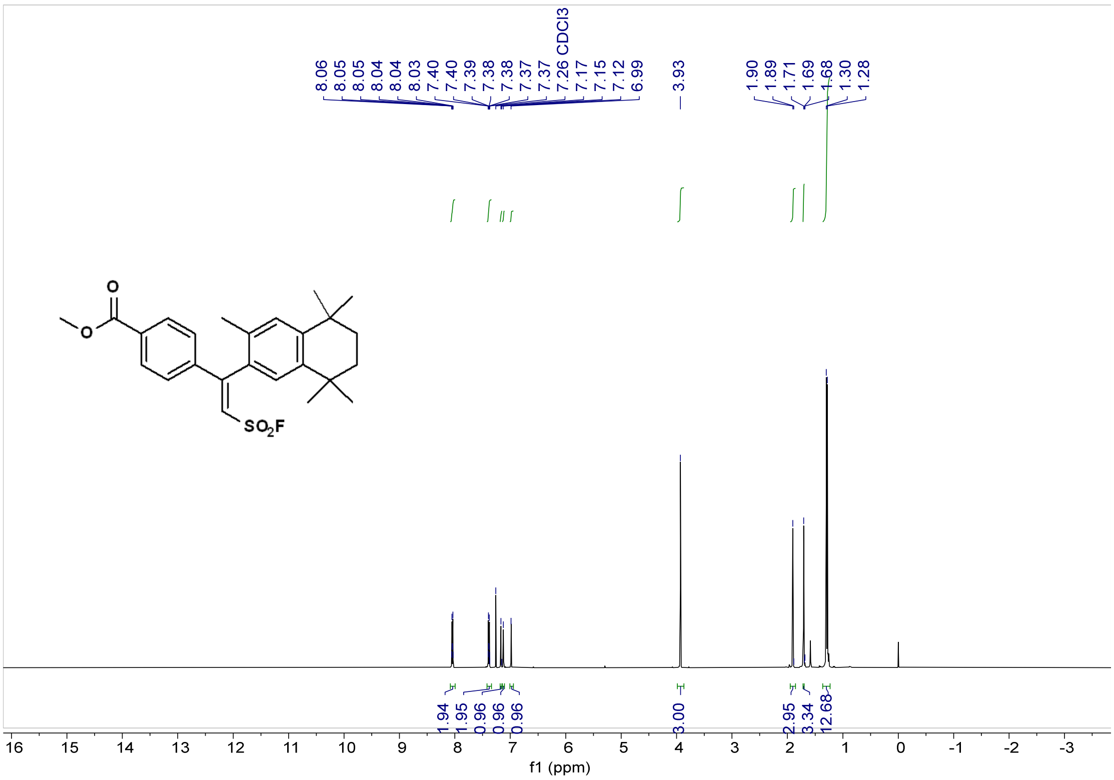

Supplementary Figure 135. ^{1}H NMR (500 MHz, room temperature, $CDCl_3$) spectra of product **4i**

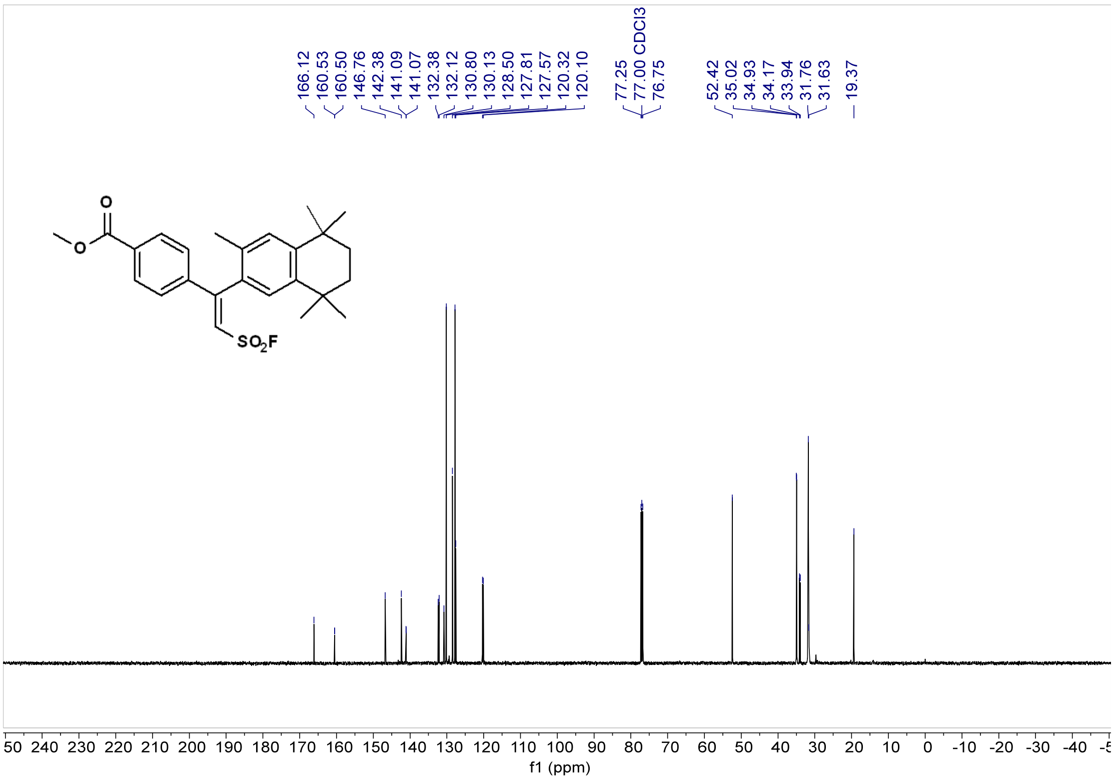

Supplementary Figure 136. ^{13}C NMR (126 MHz, room temperature, $CDCl_3$) spectra of product **4i**

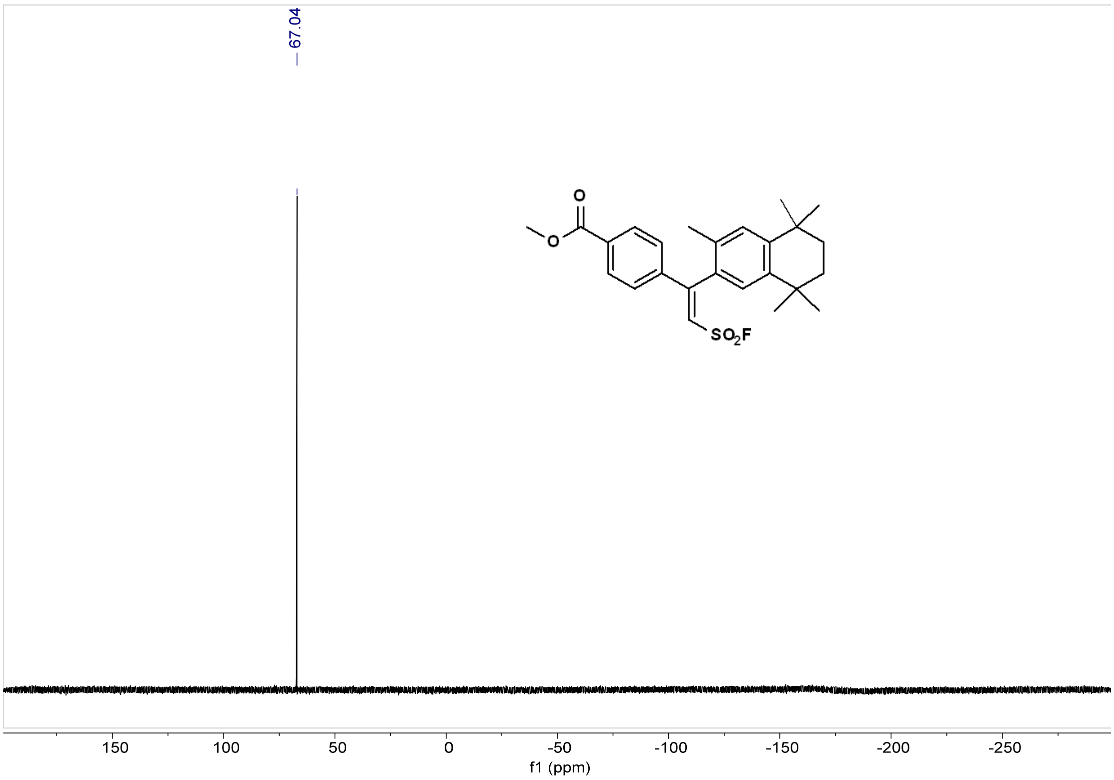

Supplementary Figure 137. ^{19}F NMR (471 MHz, room temperature, $CDCl_3$) spectra of product **4i**

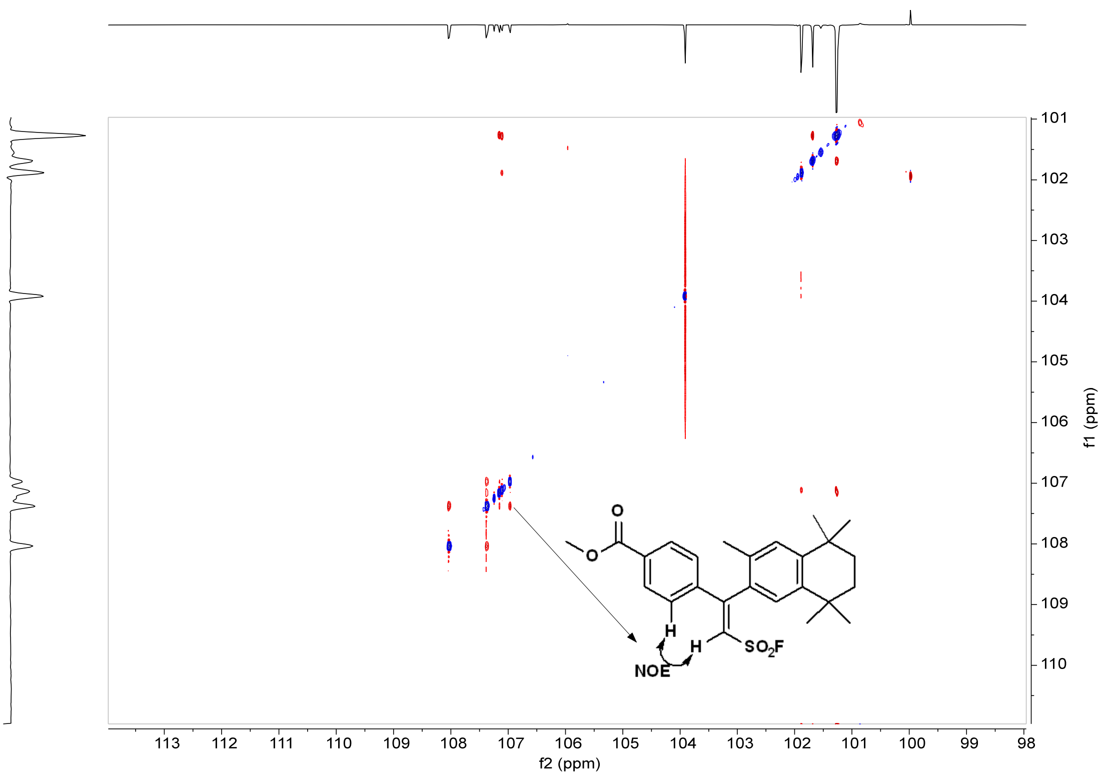

Supplementary Figure 138. NOESY (400 MHz, room temperature, $CDCl_3$) spectra of product **4i**

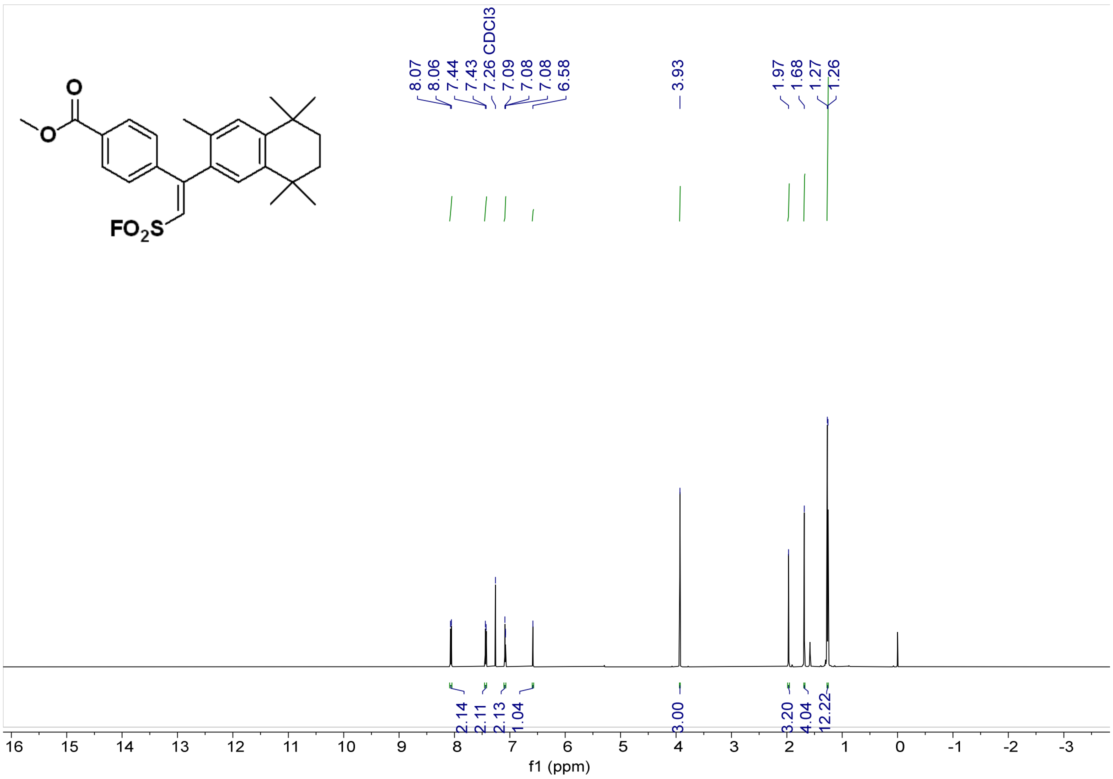

Supplementary Figure 139. ^{1}H NMR (500 MHz, room temperature, $CDCl_3$) spectra of product **4i’**

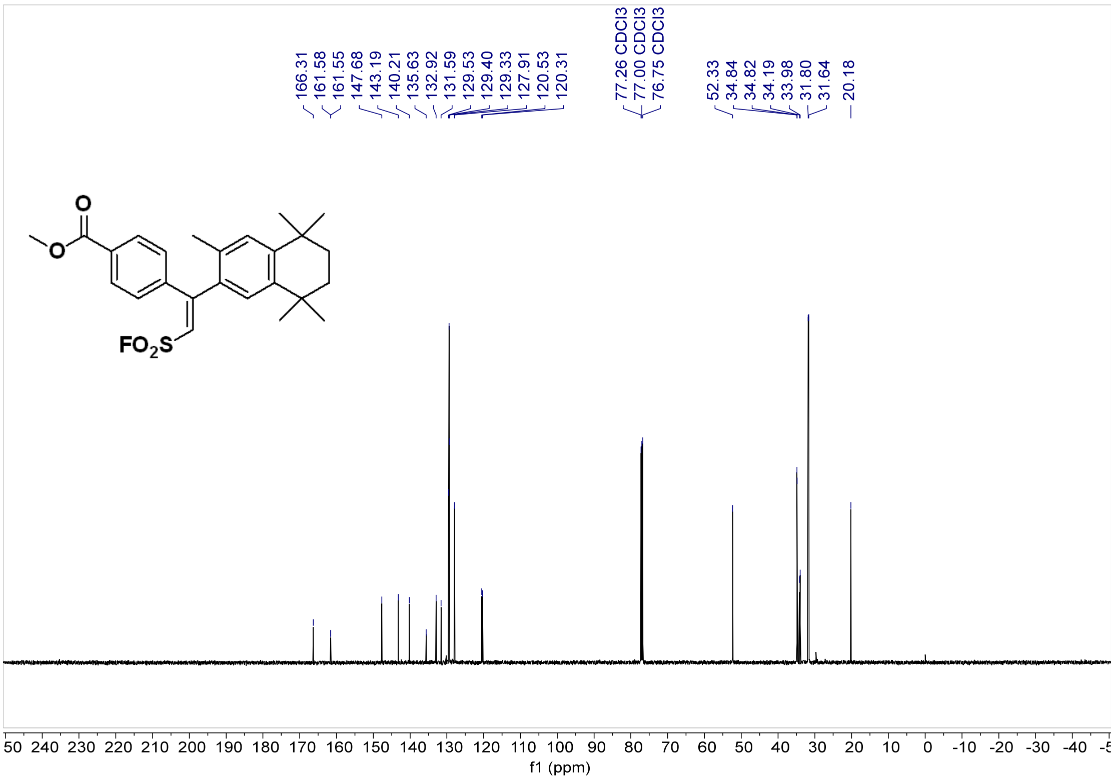

Supplementary Figure 140. ^{13}C NMR (126 MHz, room temperature, $CDCl_3$) spectra of product **4i’**

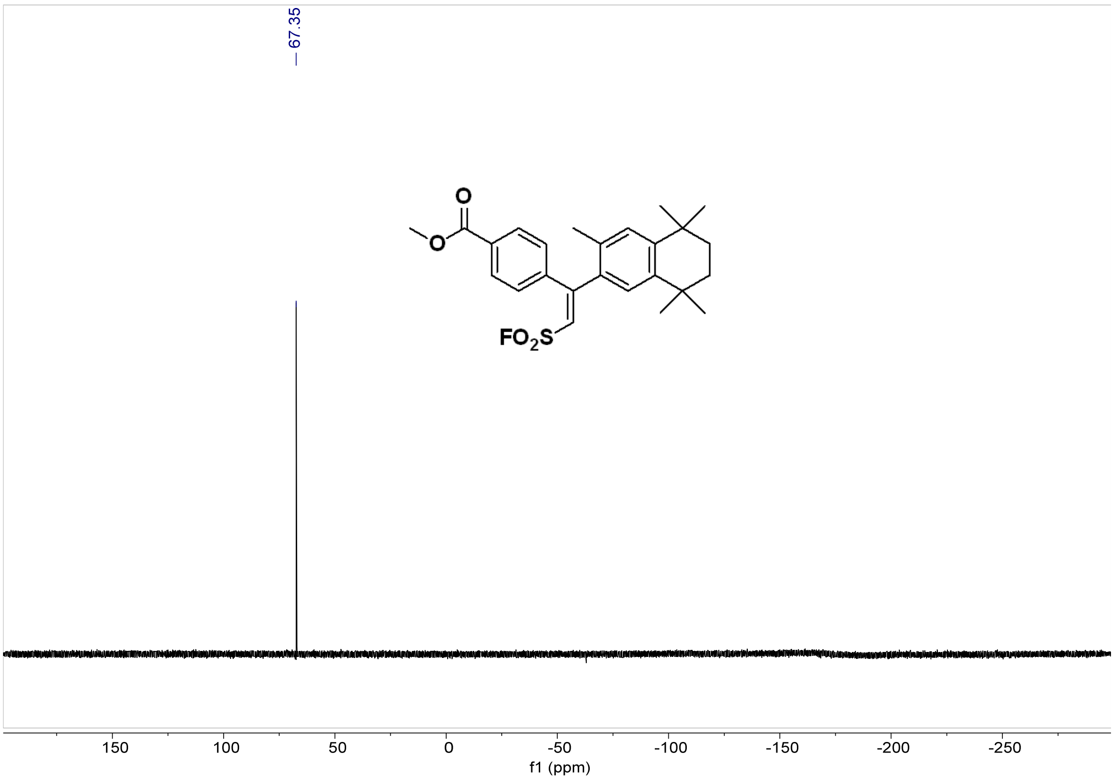

Supplementary Figure 141. ^{19}F NMR (471 MHz, room temperature, $CDCl_3$) spectra of product **4i’**

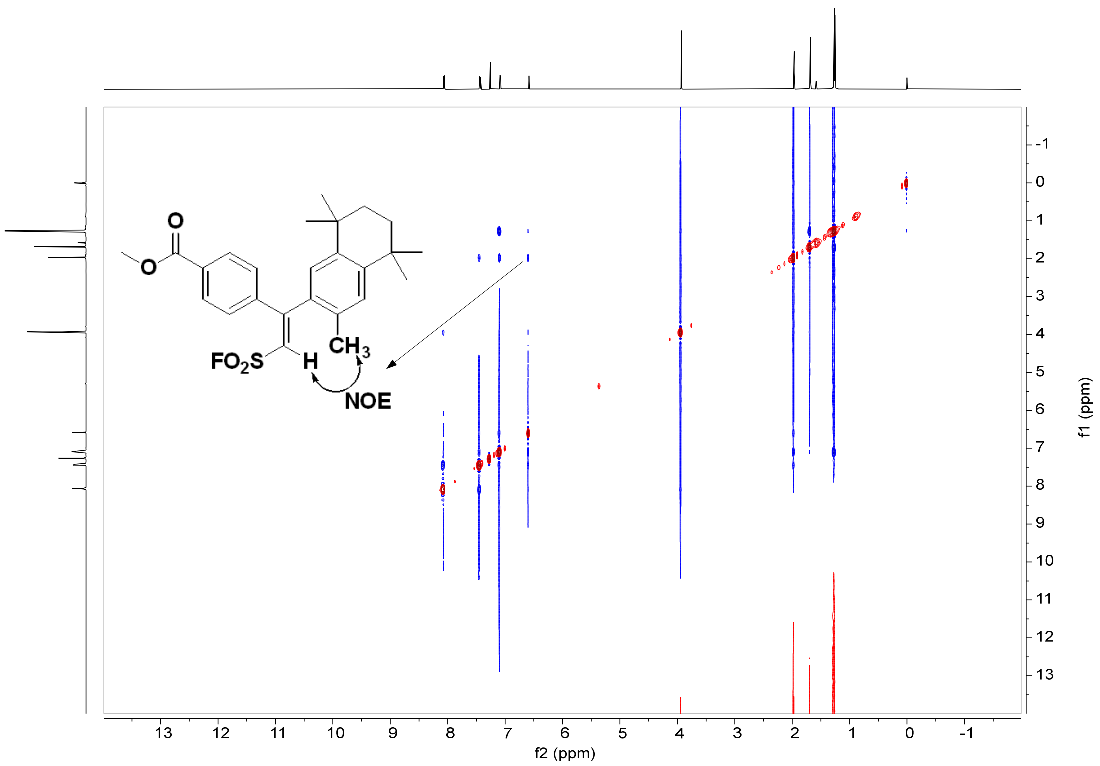

Supplementary Figure 142. NOESY (400 MHz, room temperature, $CDCl_3$) spectra of product **4i’**

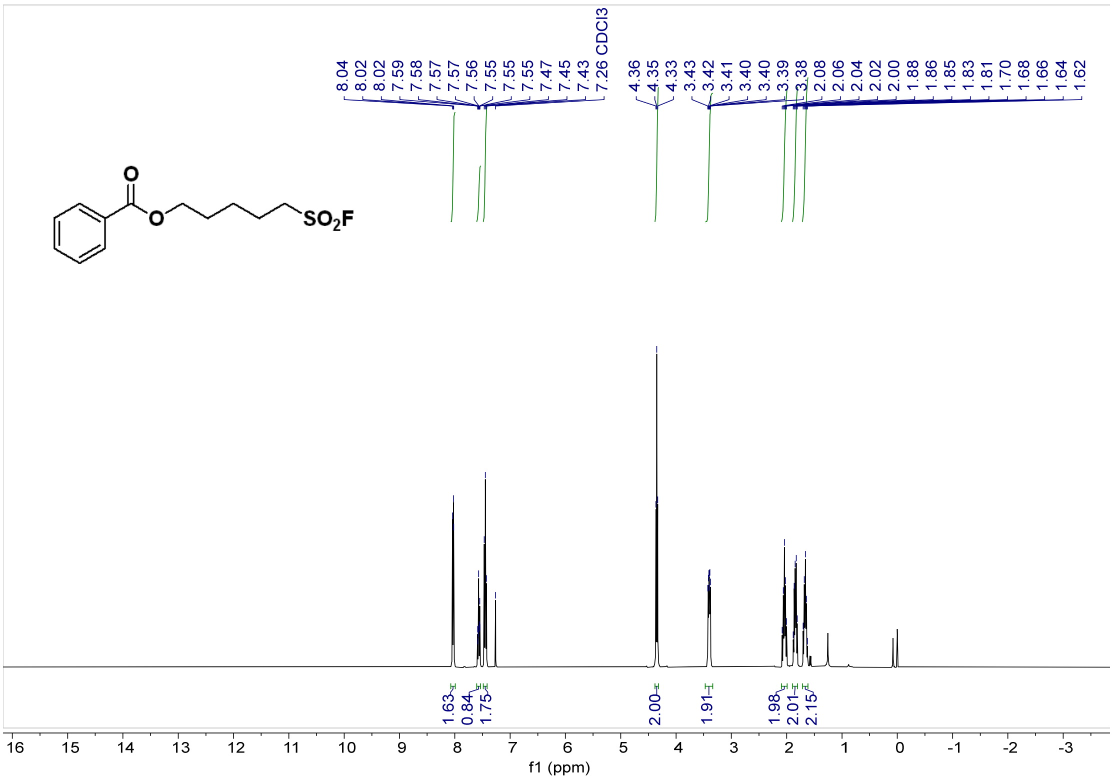

Supplementary Figure 143. ^{1}H NMR (400 MHz, room temperature, $CDCl_3$) spectra of product **6a**

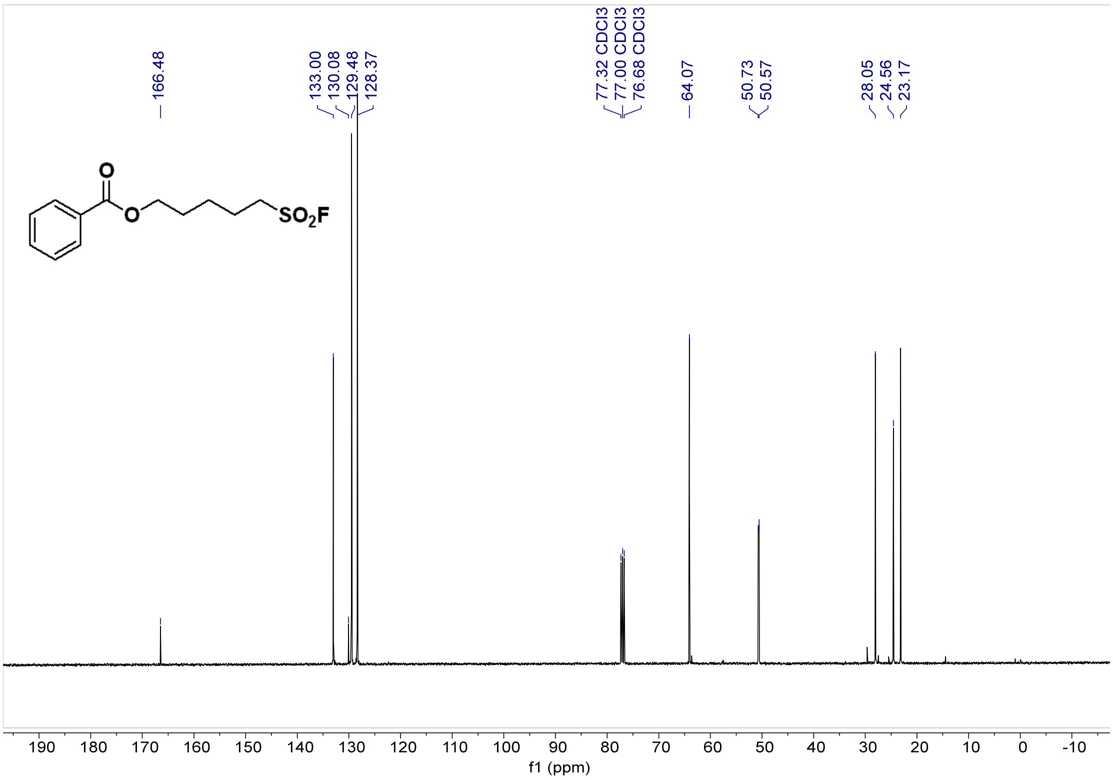

Supplementary Figure 144. ^{13}C NMR (101 MHz, room temperature, $CDCl_3$) spectra of product **6a**

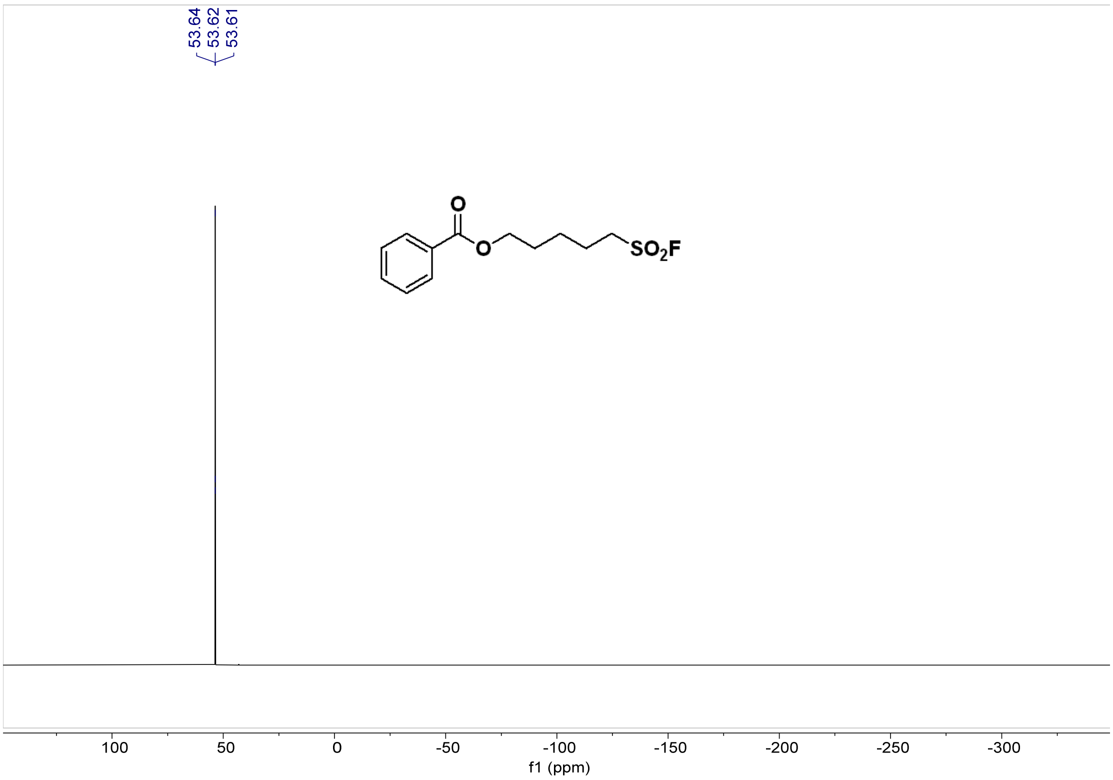

Supplementary Figure 145. ^{19}F NMR (376 MHz, room temperature, $CDCl_3$) spectra of product **6a**

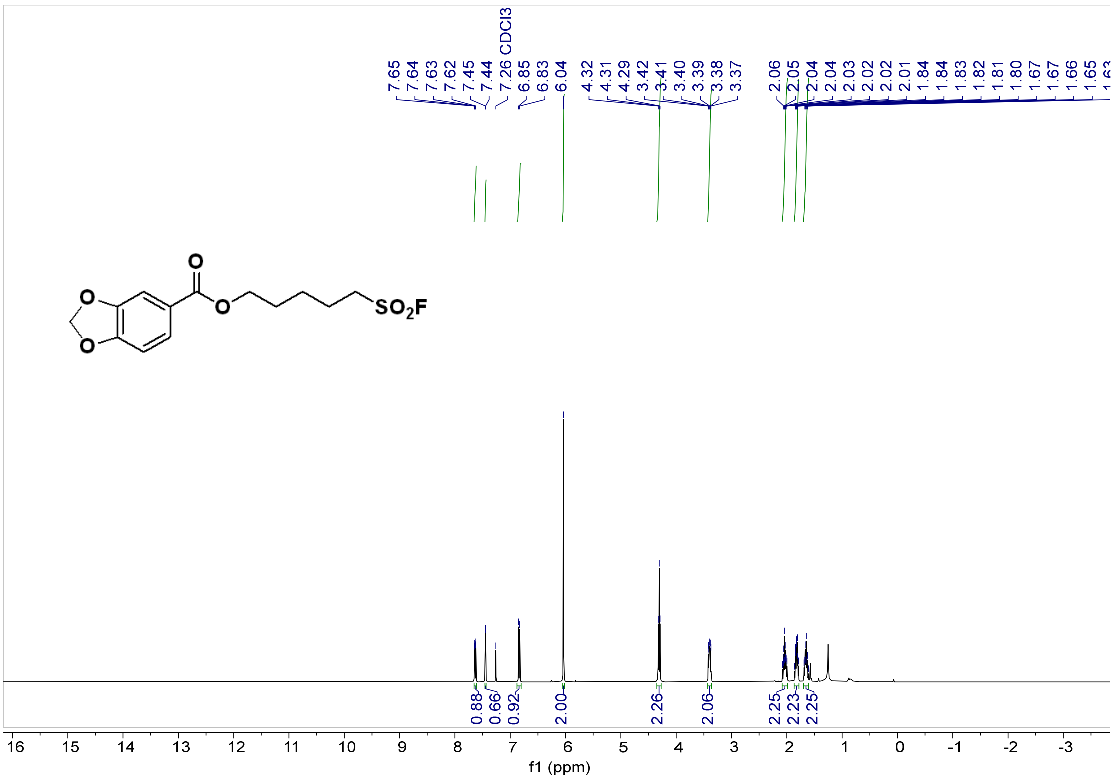

Supplementary Figure 146. ^{1}H NMR (400 MHz, room temperature, $CDCl_3$) spectra of product **6b**

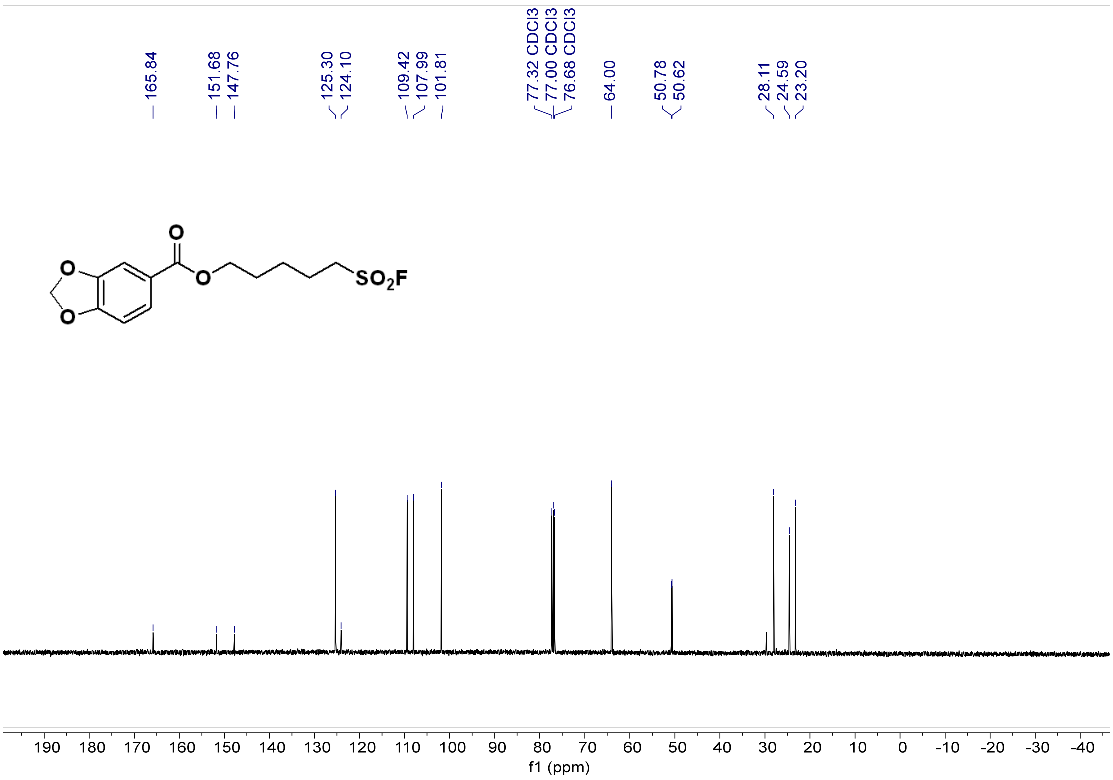

Supplementary Figure 147. ^{13}C NMR (101 MHz, room temperature, $CDCl_3$) spectra of product **6b**

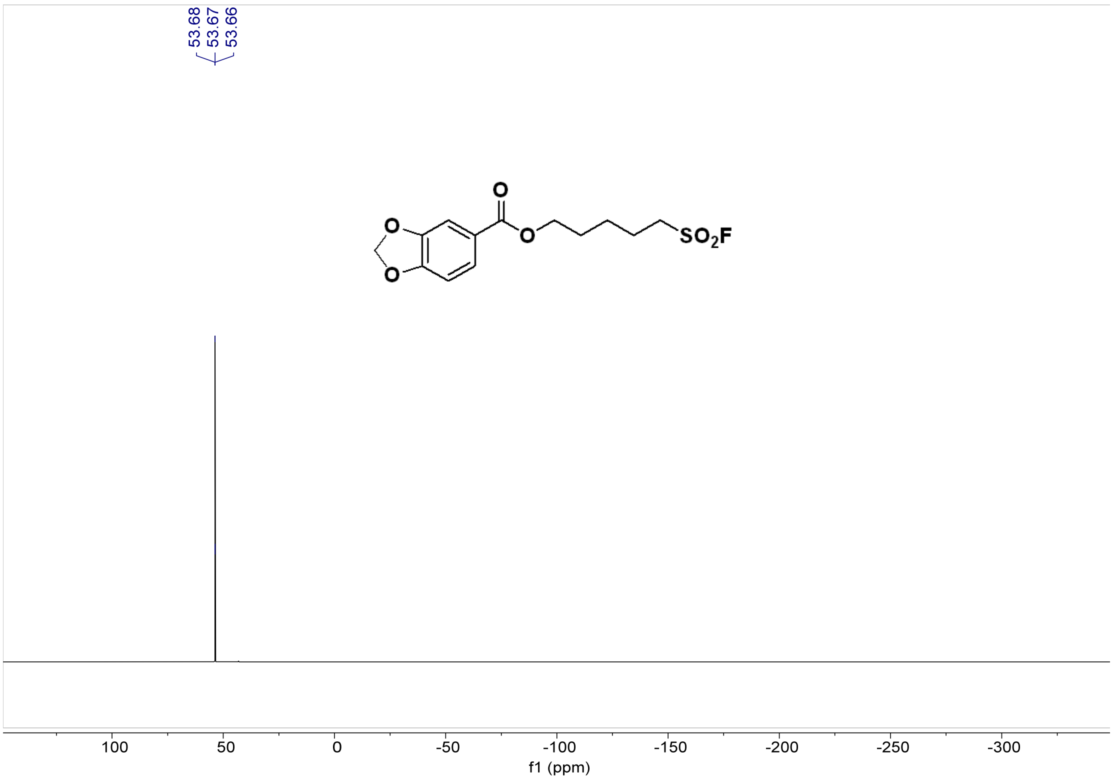

Supplementary Figure 148. ^{19}F NMR (376 MHz, room temperature, $CDCl_3$) spectra of product **6b**

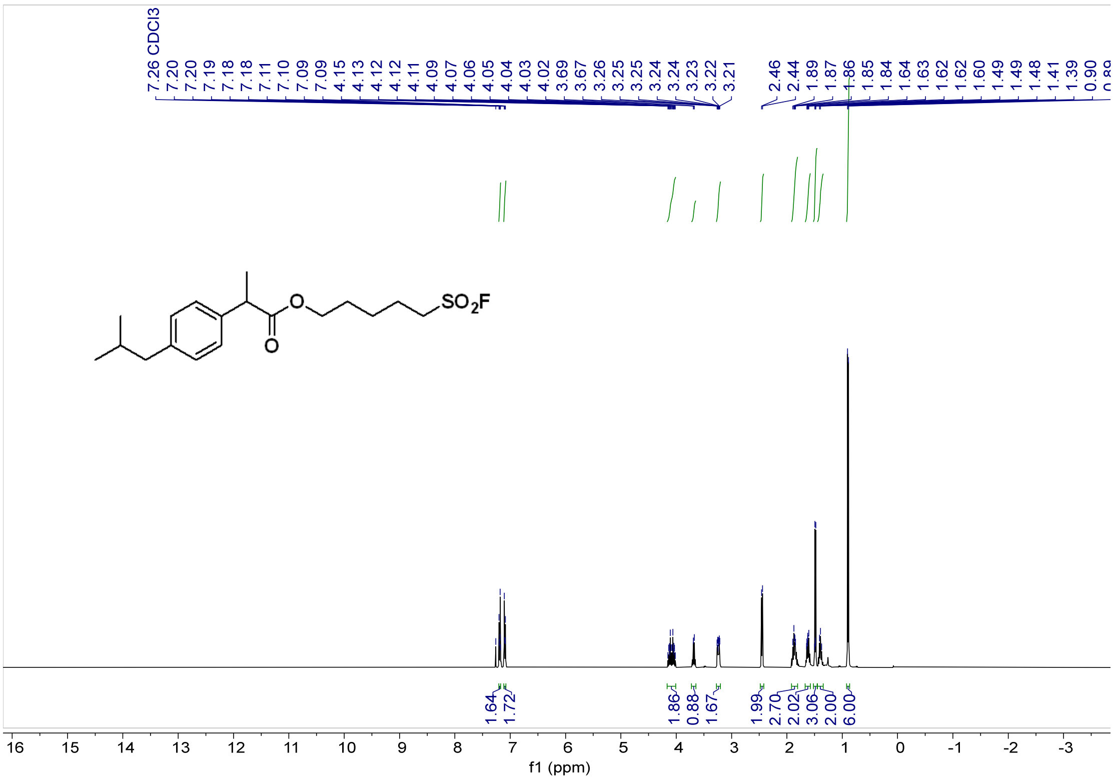

Supplementary Figure 149. ^{1}H NMR (400 MHz, room temperature, $CDCl_3$) spectra of product **6c**

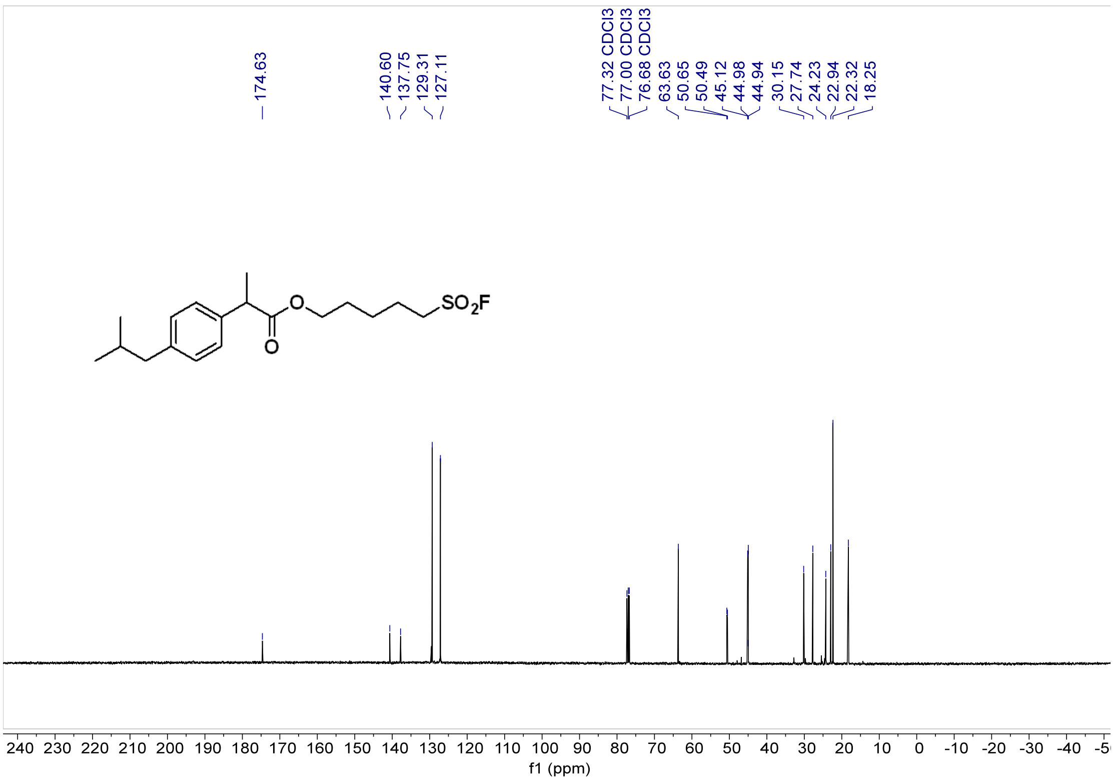

Supplementary Figure 150. ^{13}C NMR (101 MHz, room temperature, $CDCl_3$) spectra of product **6c**

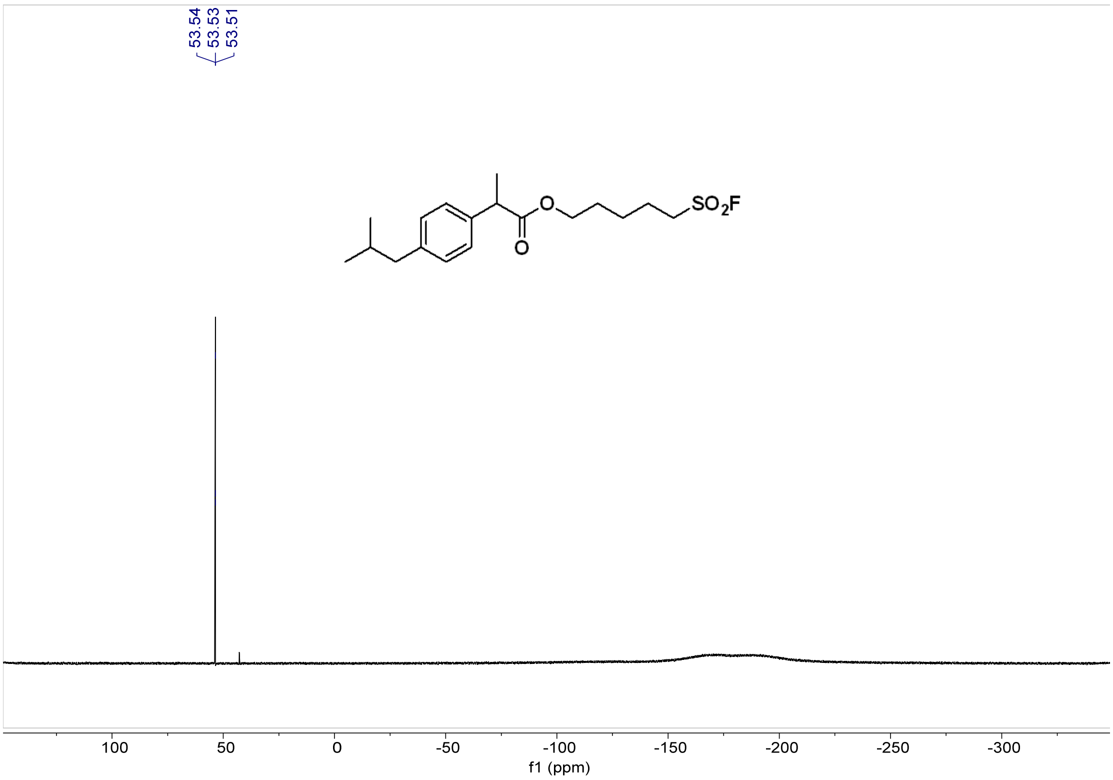

Supplementary Figure 151. ^{19}F NMR (376 MHz, room temperature, $CDCl_3$) spectra of product **6c**

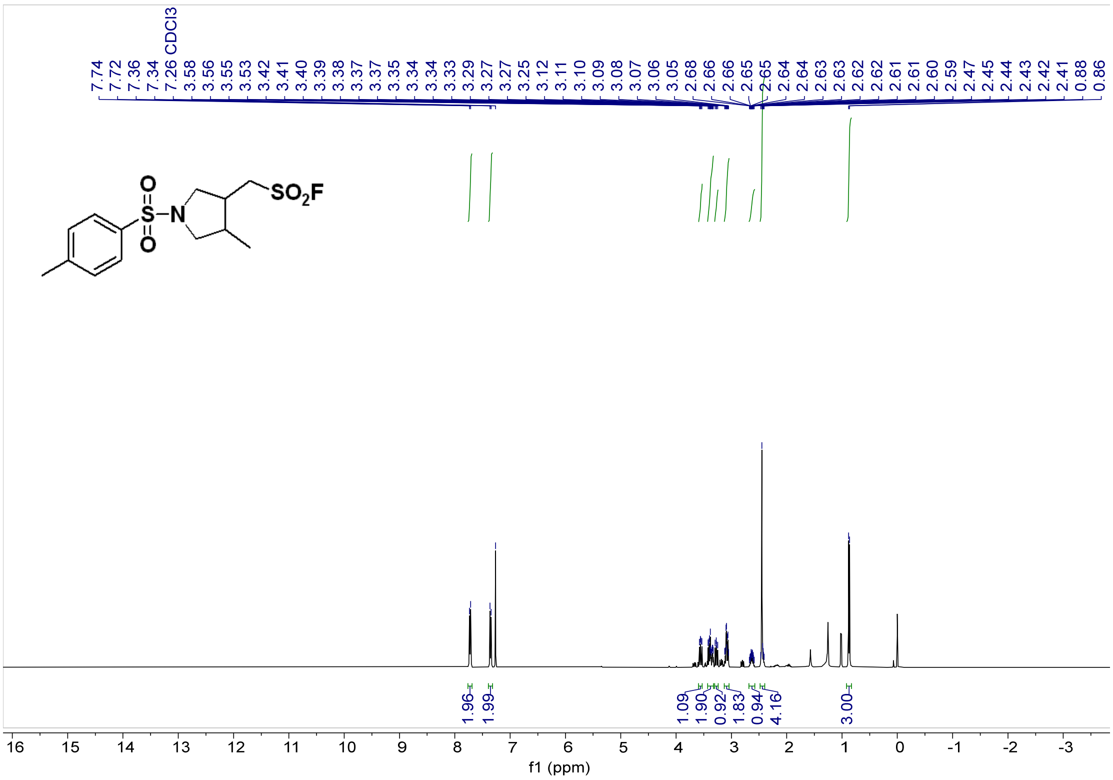

Supplementary Figure 152. ^{1}H NMR (400 MHz, room temperature, $CDCl_3$) spectra of product **6d**

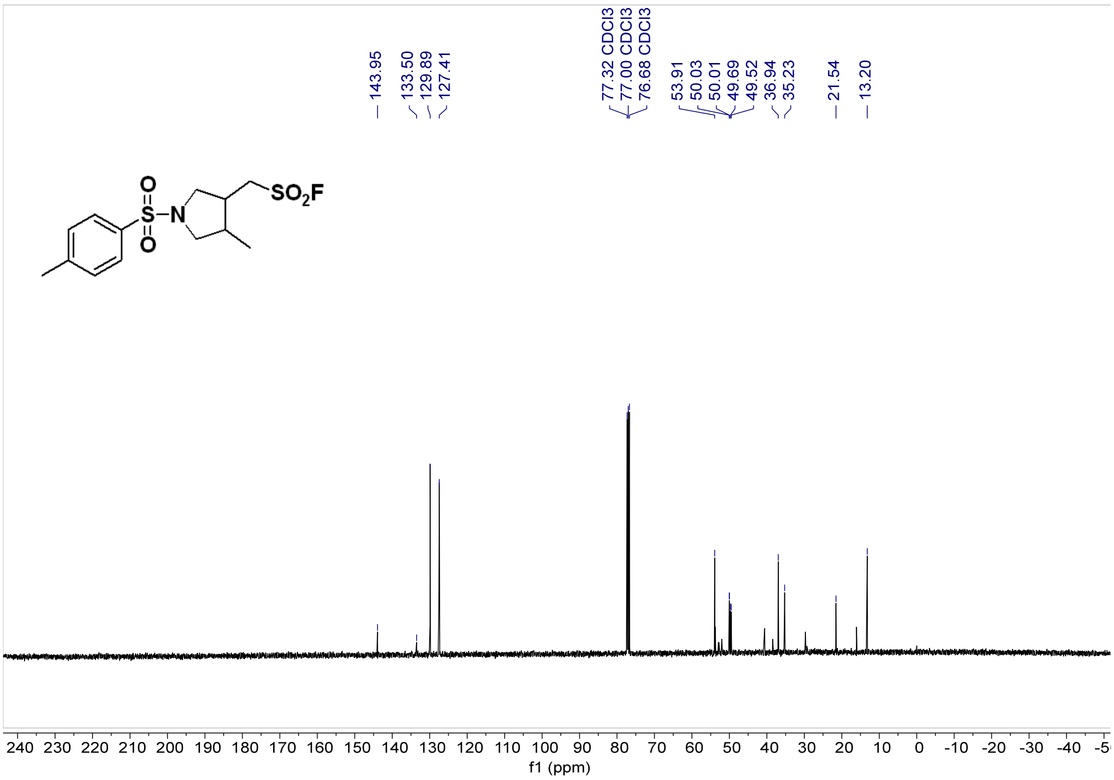

Supplementary Figure 153. ^{13}C NMR (101 MHz, room temperature, $CDCl_3$) spectra of product **6d**

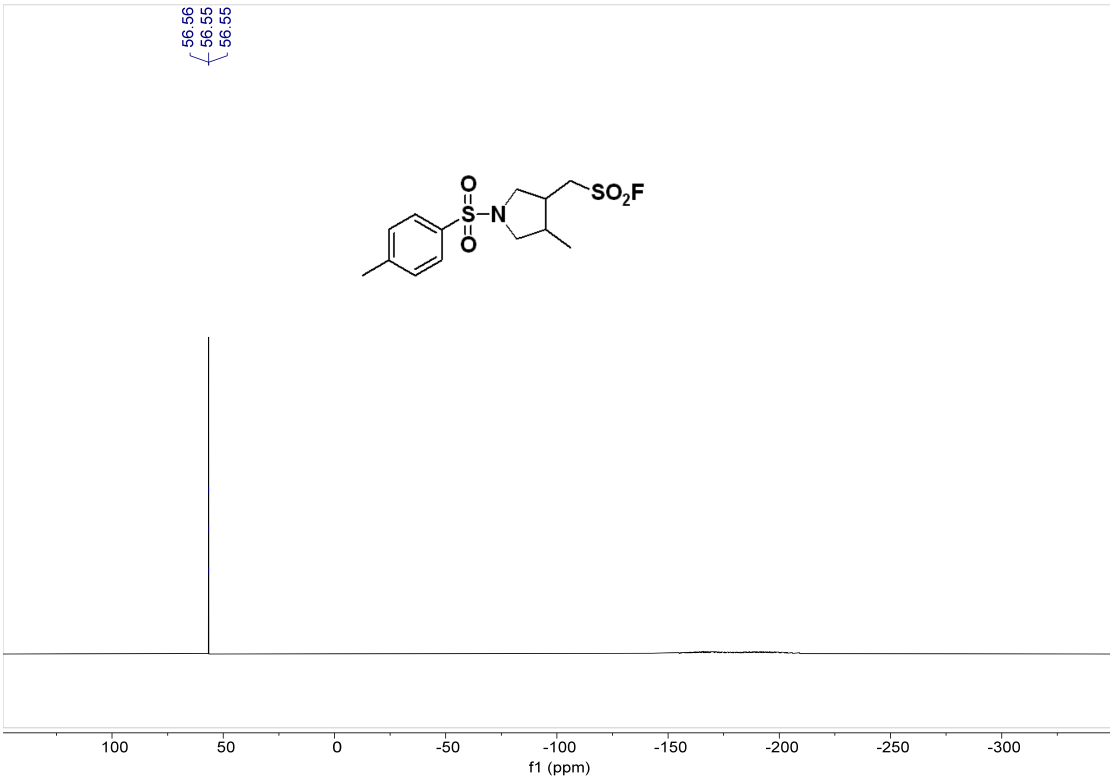

Supplementary Figure 154. ^{19}F NMR (376 MHz, room temperature, $CDCl_3$) spectra of product **6d**

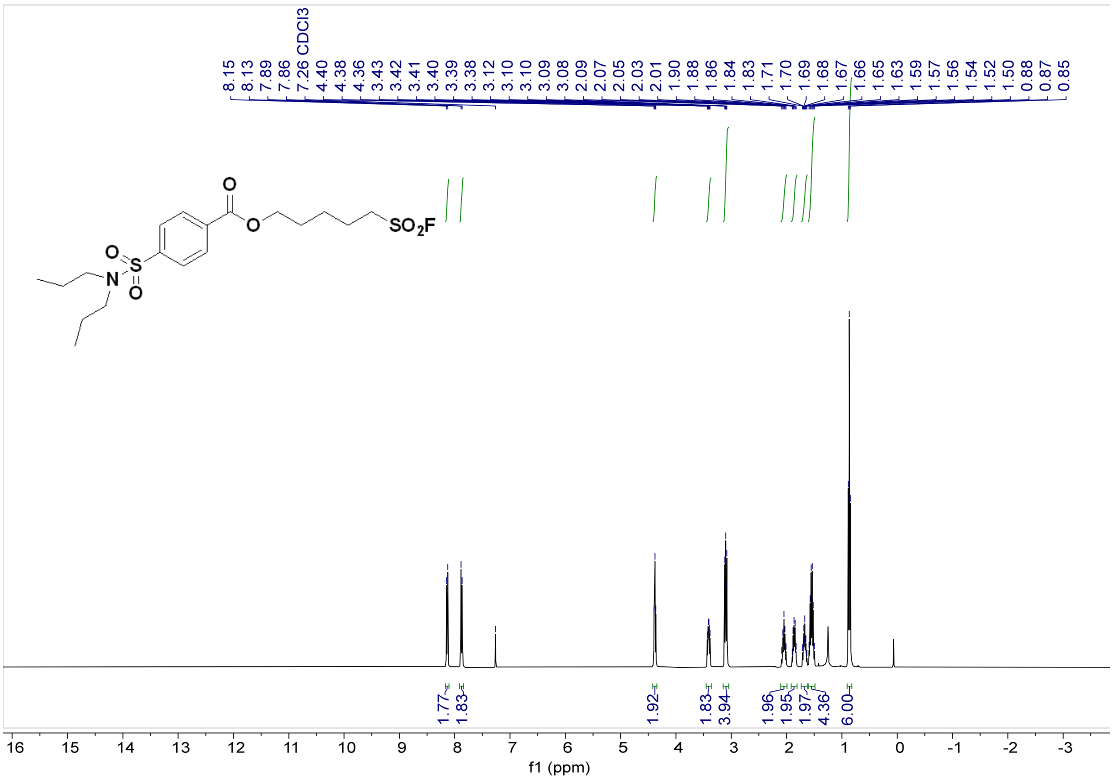

Supplementary Figure 155. ^{1}H NMR (400 MHz, room temperature, $CDCl_3$) spectra of product **6e**

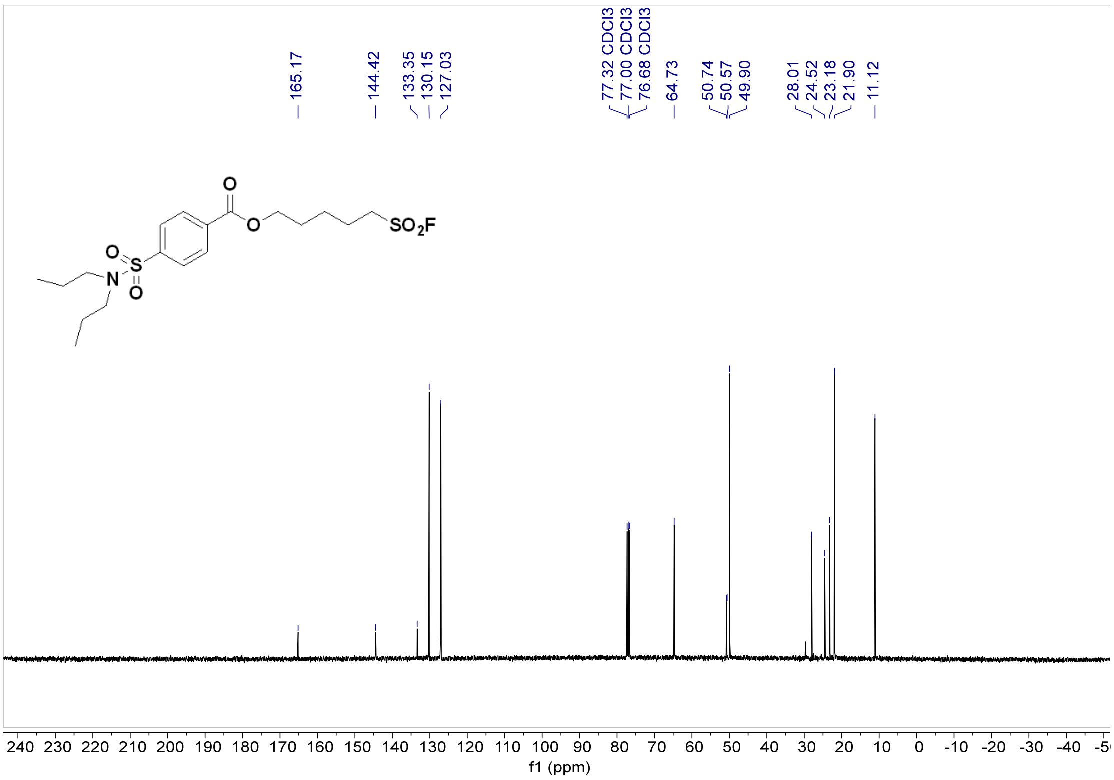

Supplementary Figure 156. ^{13}C NMR (101 MHz, room temperature, $CDCl_3$) spectra of product **6e**

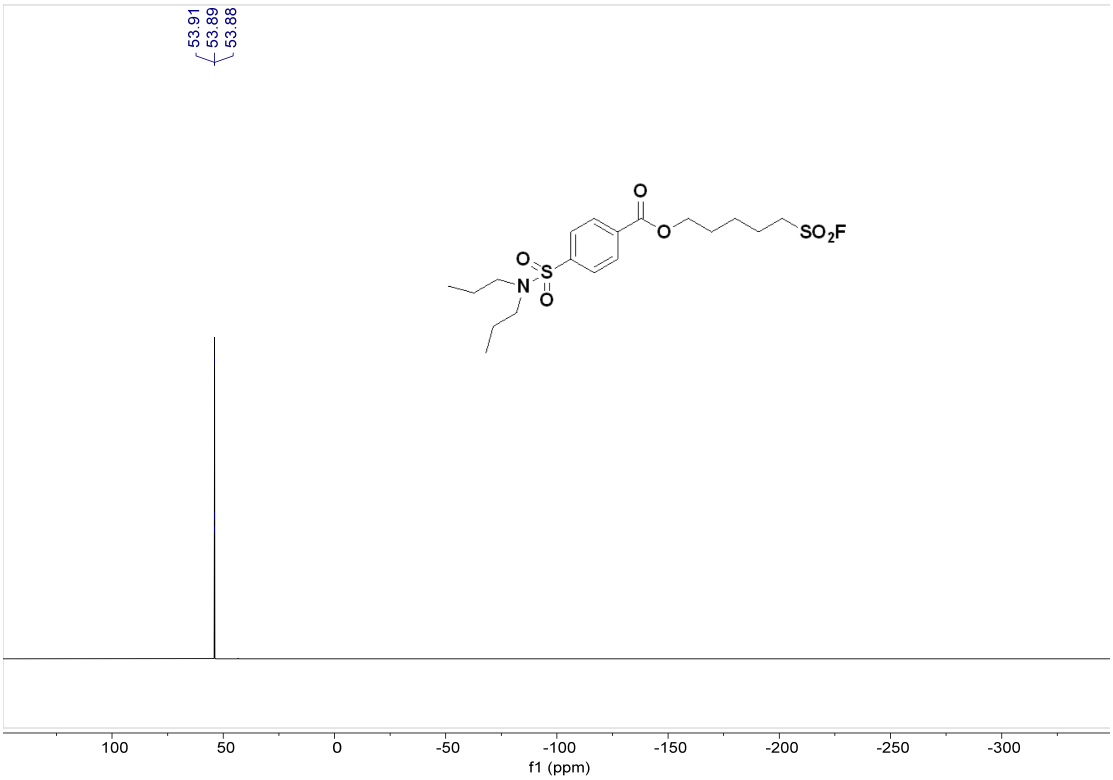

Supplementary Figure 157. ^{19}F NMR (376 MHz, room temperature, $CDCl_3$) spectra of product **6e**

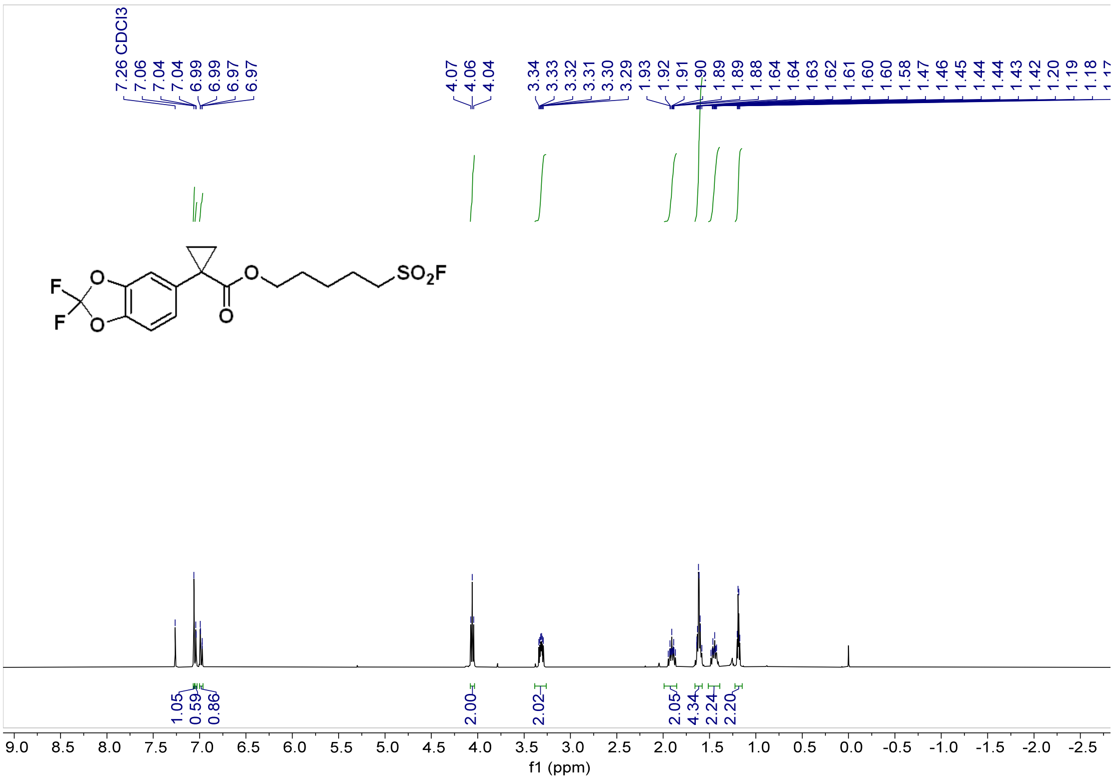

Supplementary Figure 158. ^{1}H NMR (400 MHz, room temperature, $CDCl_3$) spectra of product **6f**

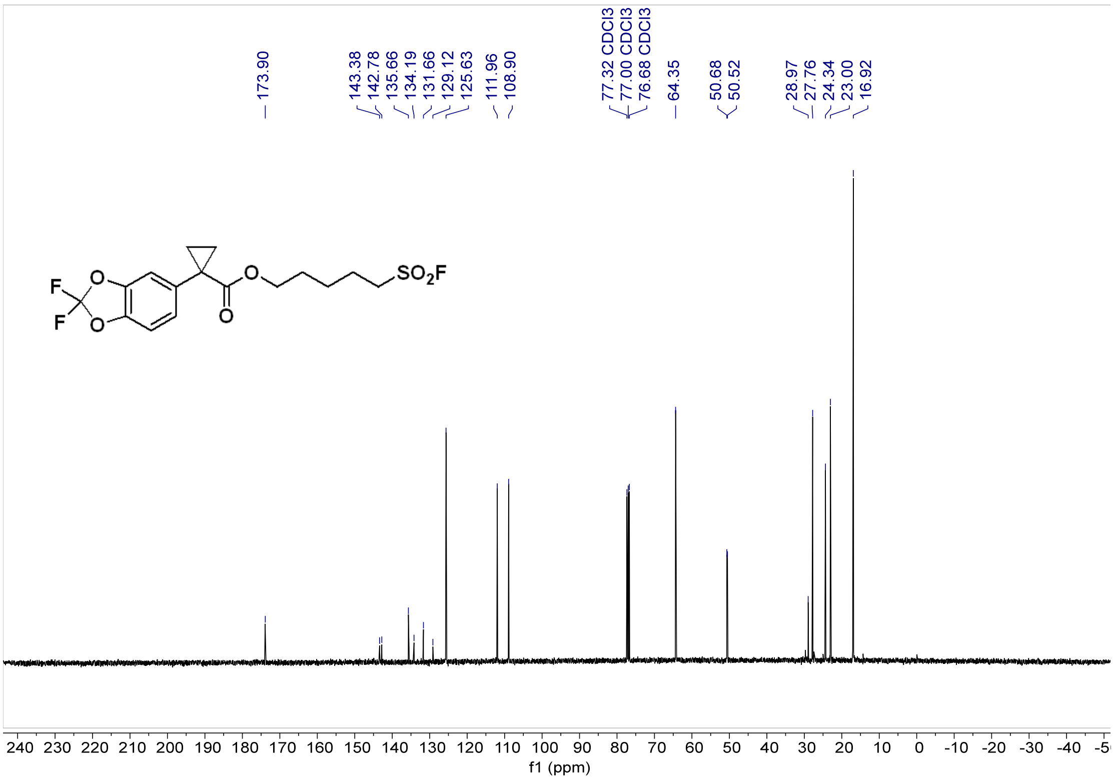

Supplementary Figure 159. ^{13}C NMR (101 MHz, room temperature, $CDCl_3$) spectra of product **6f**

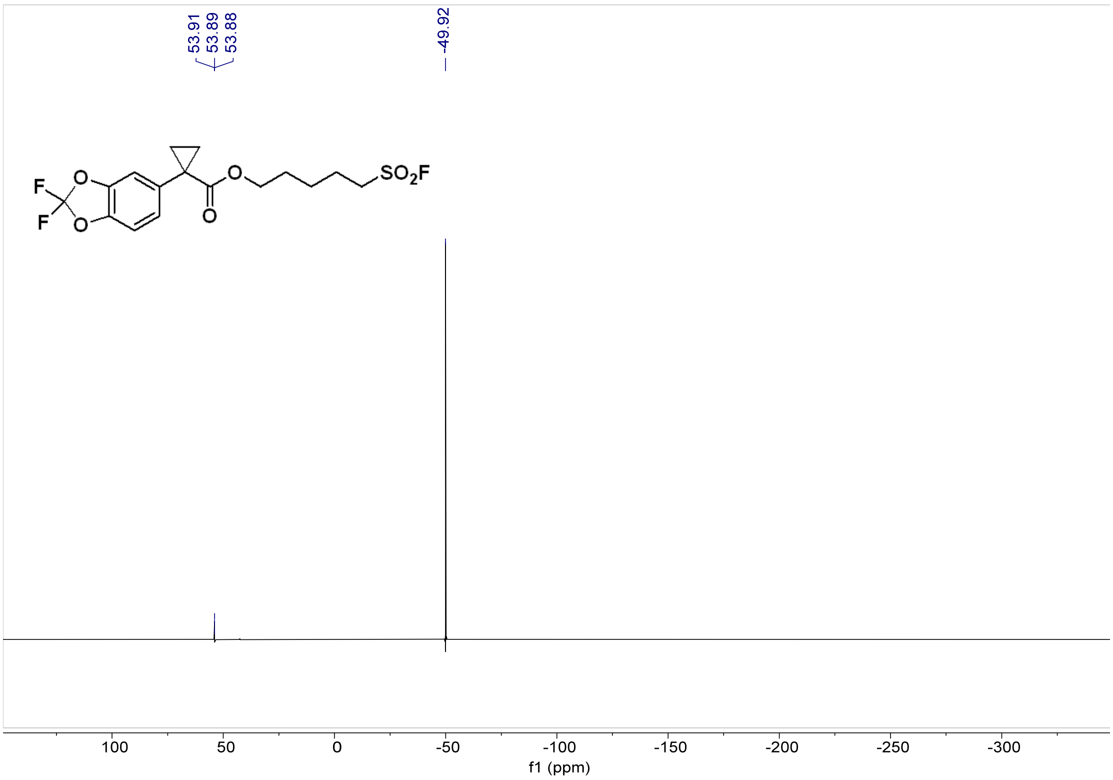

Supplementary Figure 160. ^{19}F NMR (376 MHz, room temperature, $CDCl_3$) spectra of product **6f**

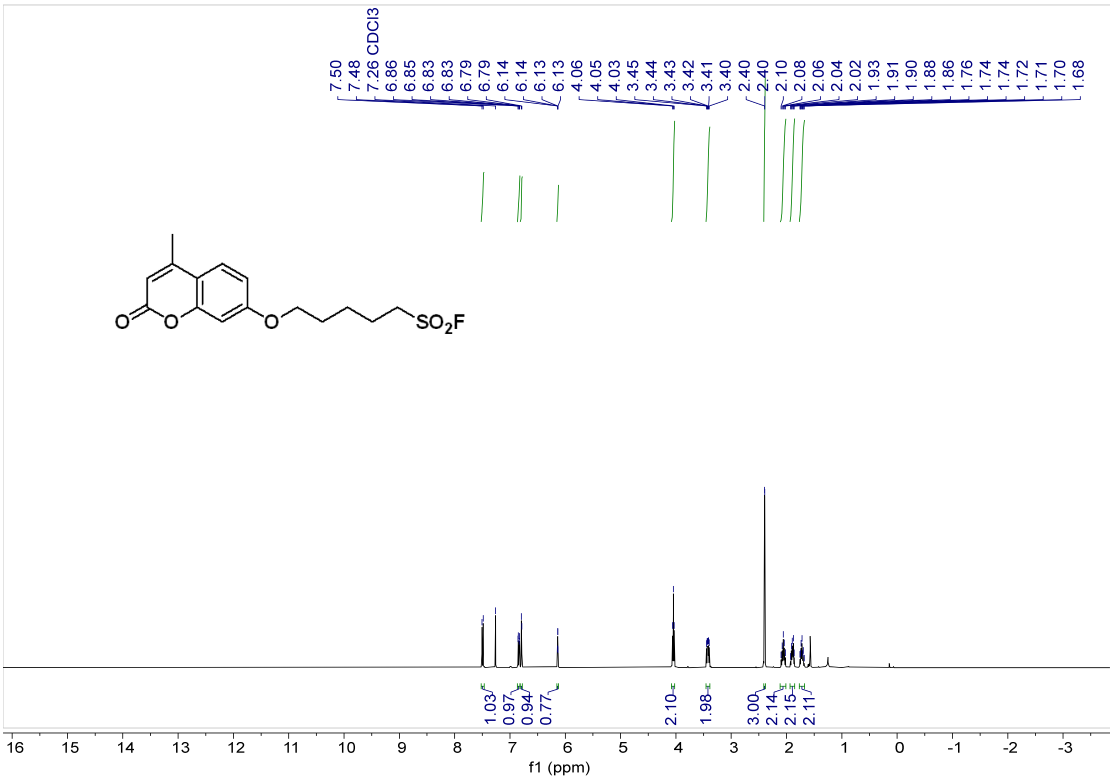

Supplementary Figure 161. ^{1}H (400 MHz, room temperature, $CDCl_3$) NMR spectra of product **6g**

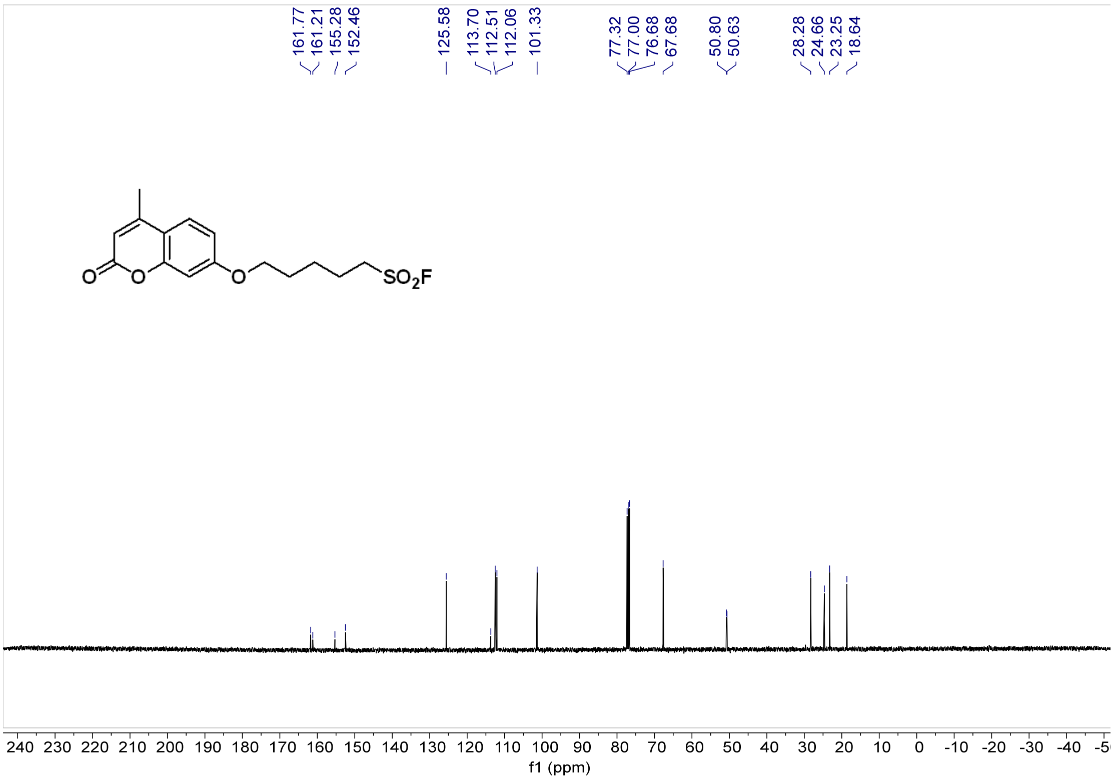

Supplementary Figure 162. ^{13}C NMR (101 MHz, room temperature, $CDCl_3$) spectra of product **6g**

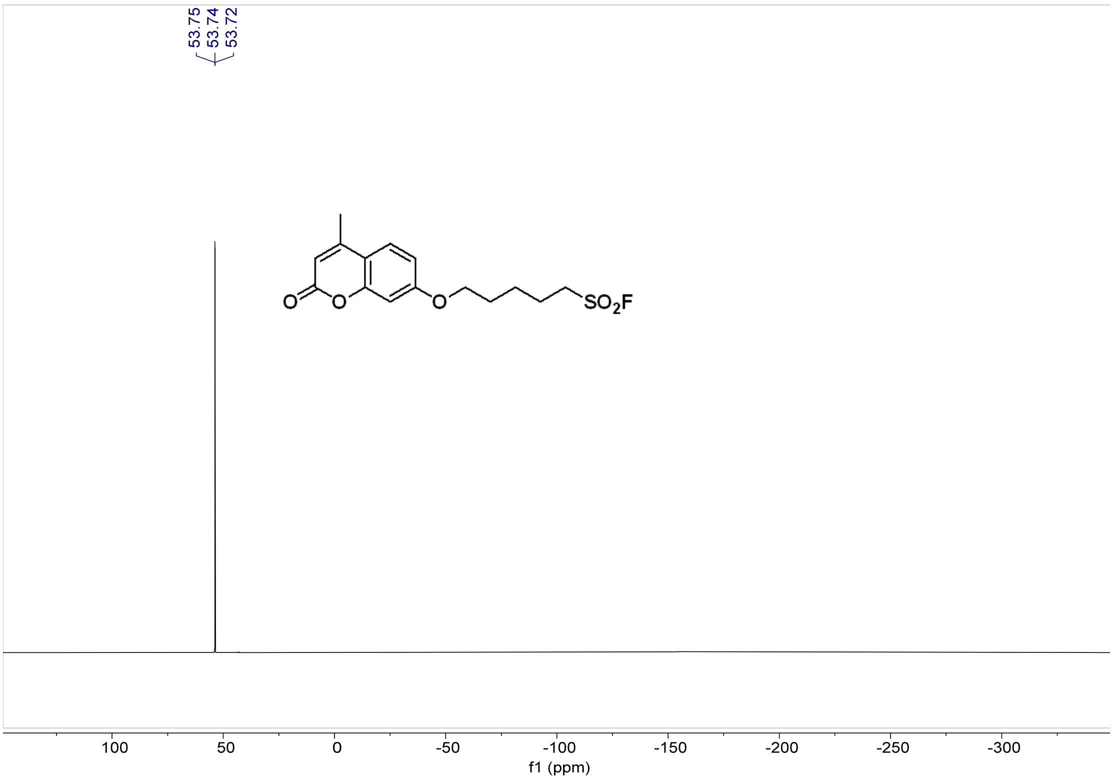

Supplementary Figure 163. ^{19}F NMR (376 MHz, room temperature, $CDCl_3$) spectra of product **6g**

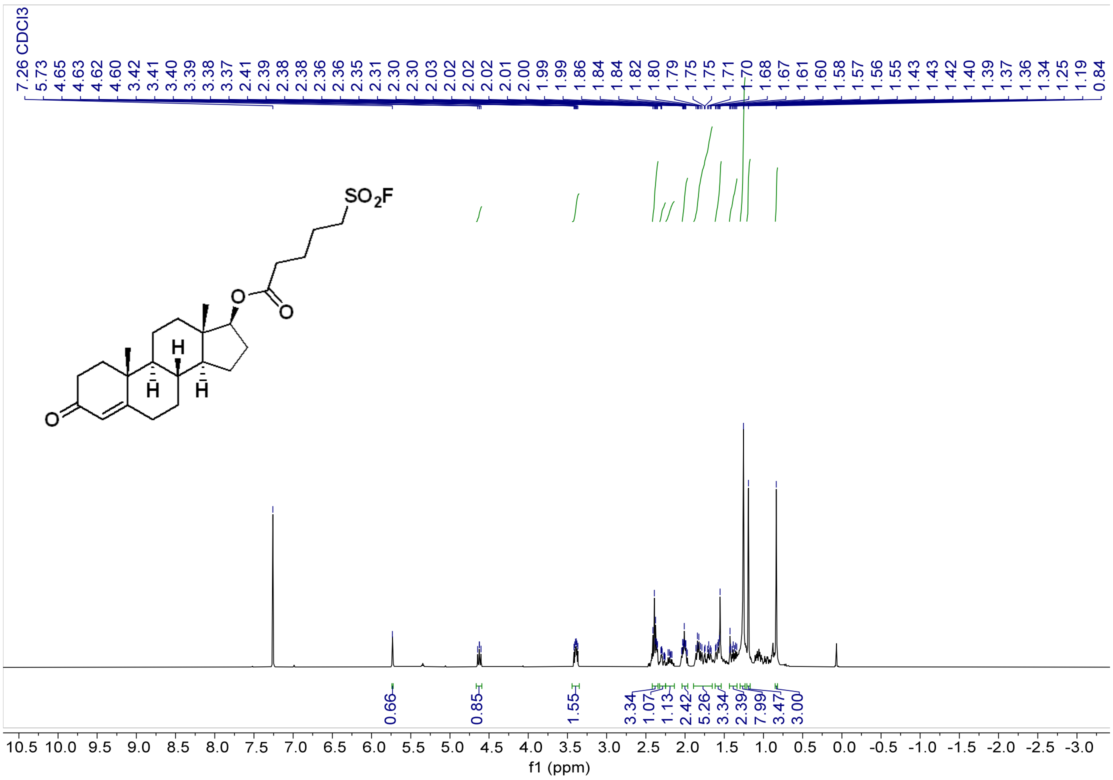

Supplementary Figure 164. ^{1}H NMR (400 MHz, room temperature, $CDCl_3$) spectra of product **6h**

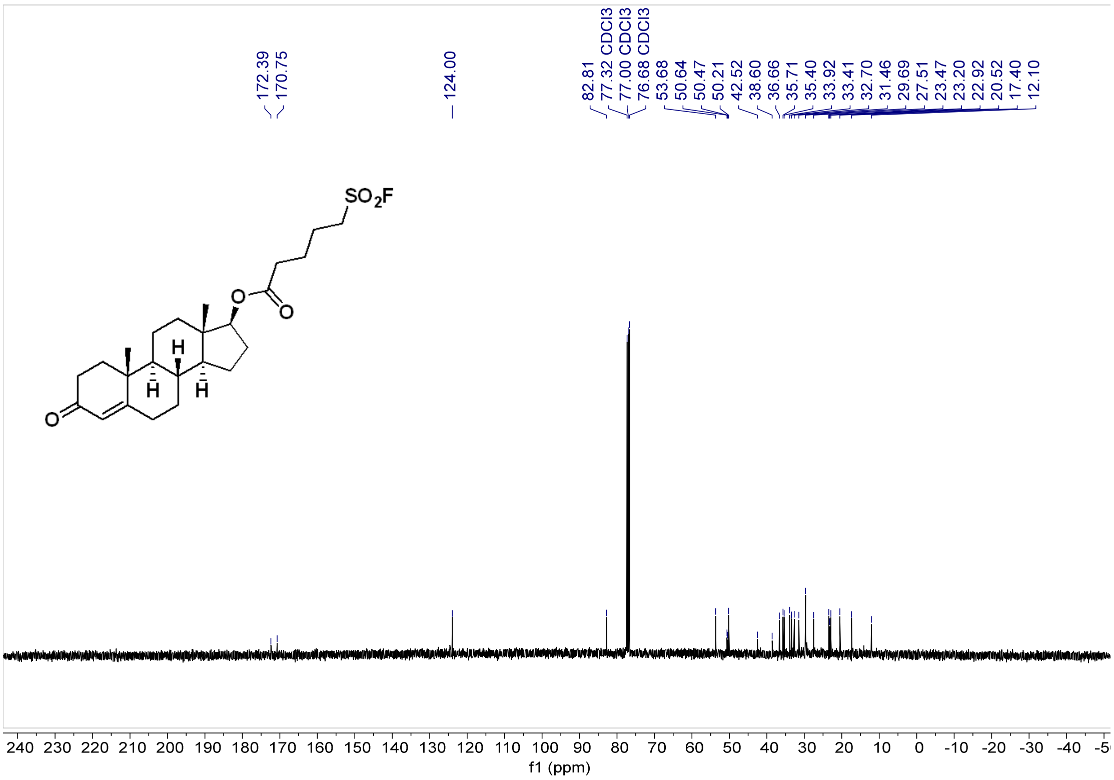

Supplementary Figure 165. ^{13}C NMR (101 MHz, room temperature, $CDCl_3$) spectra of product **6h**

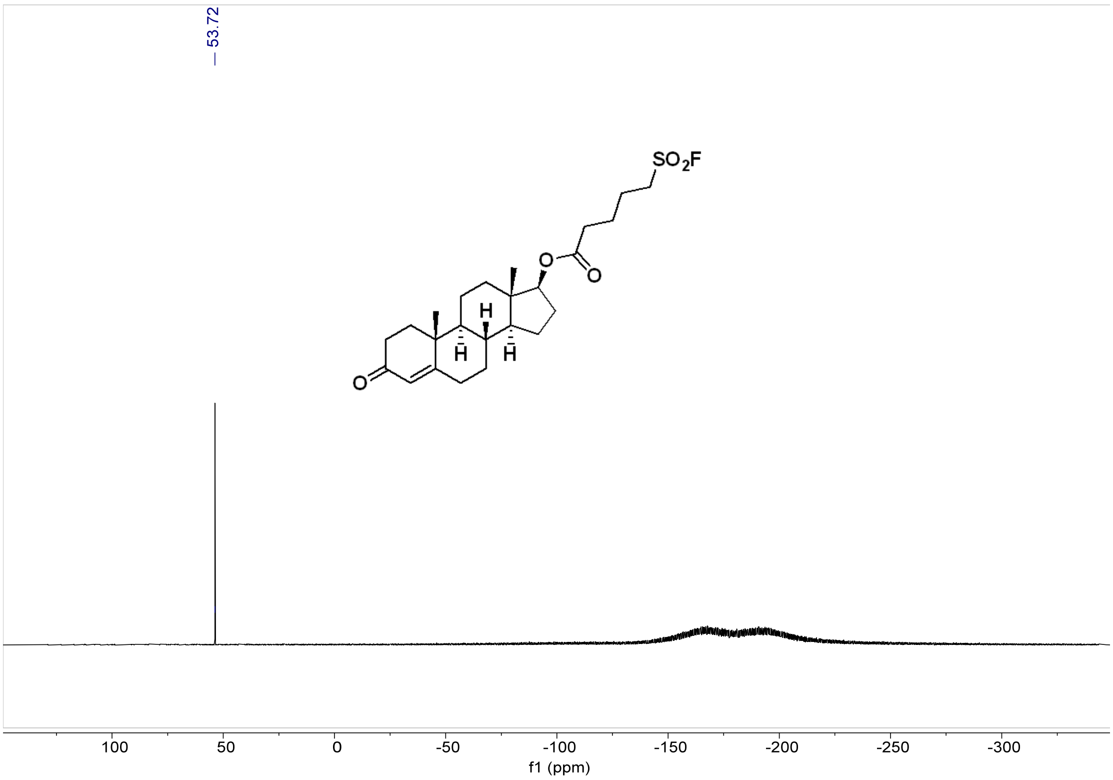

Supplementary Figure 166. ^{19}F NMR (376 MHz, room temperature, $CDCl_3$) spectra of product **6h**

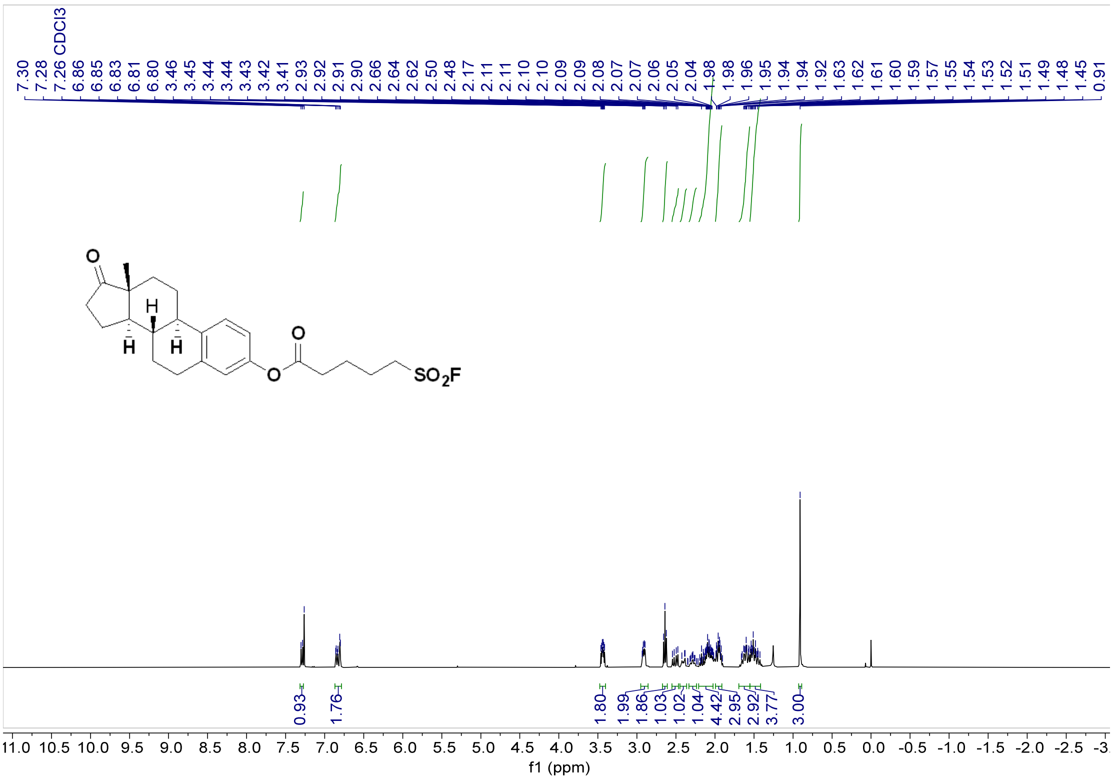

Supplementary Figure 167. ^{1}H NMR (400 MHz, room temperature, $CDCl_3$) spectra of product **6i**

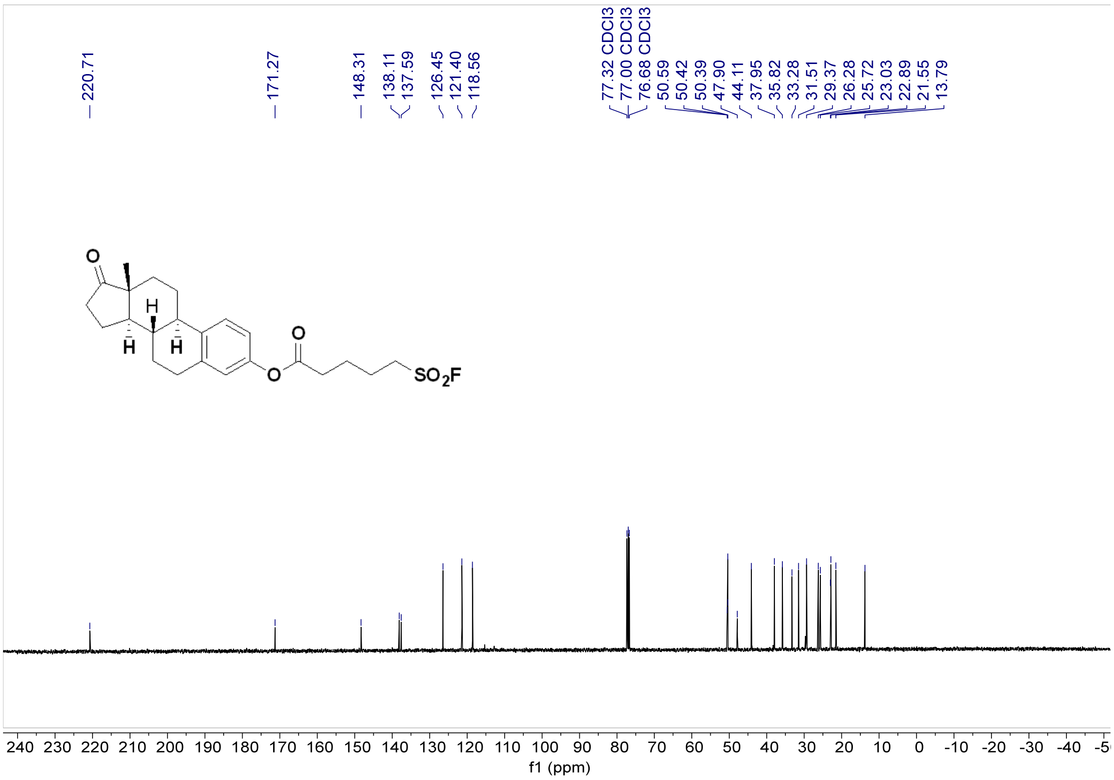

Supplementary Figure 168. ^{13}C NMR (101 MHz, room temperature, $CDCl_3$) spectra of product **6i**

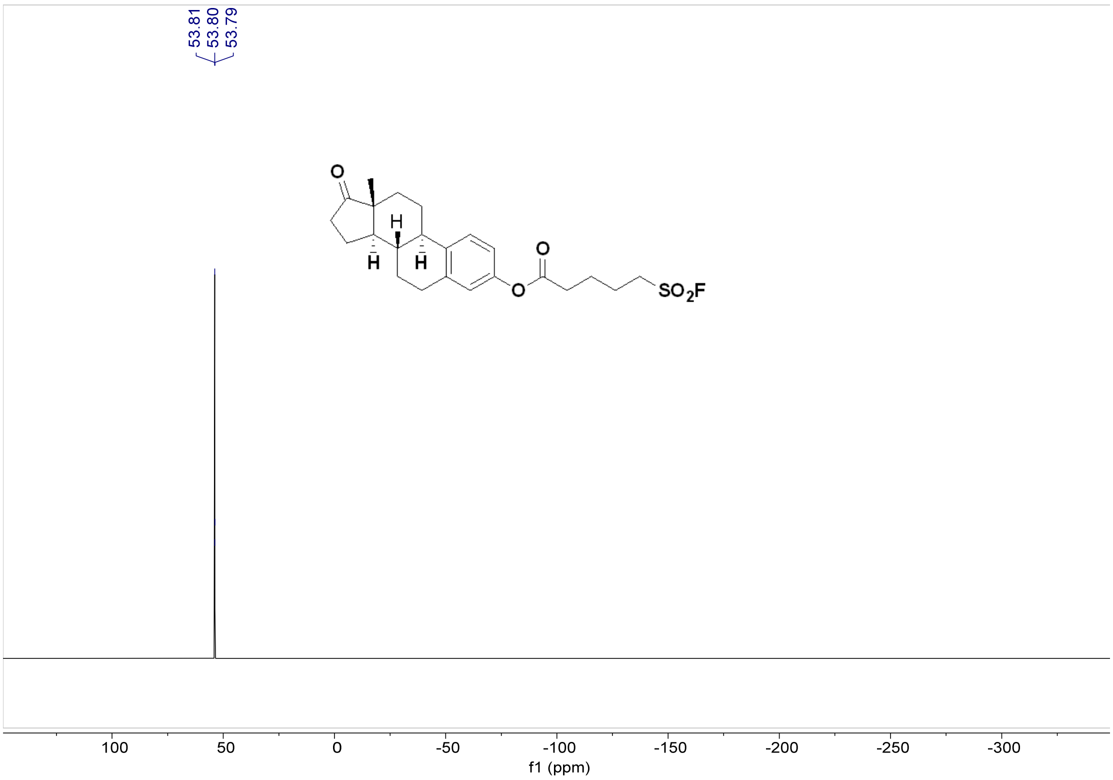

Supplementary Figure 169. ^{19}F NMR (376 MHz, room temperature, $CDCl_3$) spectra of product **6i**

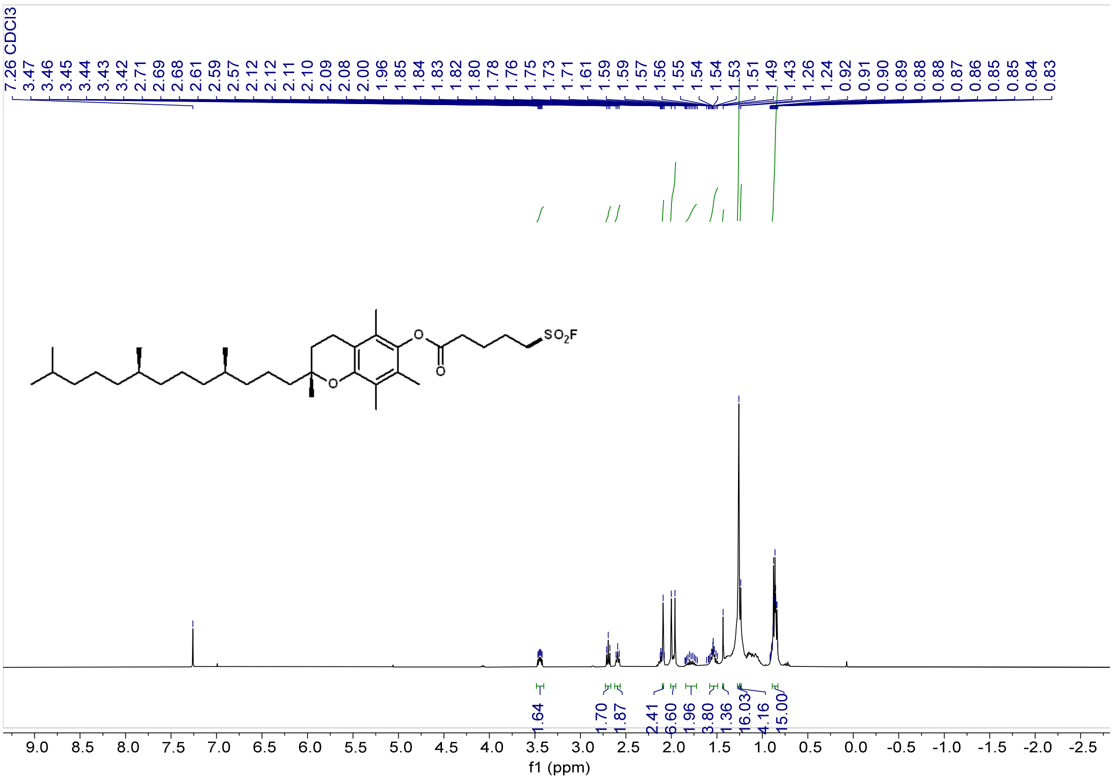

Supplementary Figure 170. ^{1}H NMR (400 MHz, room temperature, $CDCl_3$) spectra of product **6j**

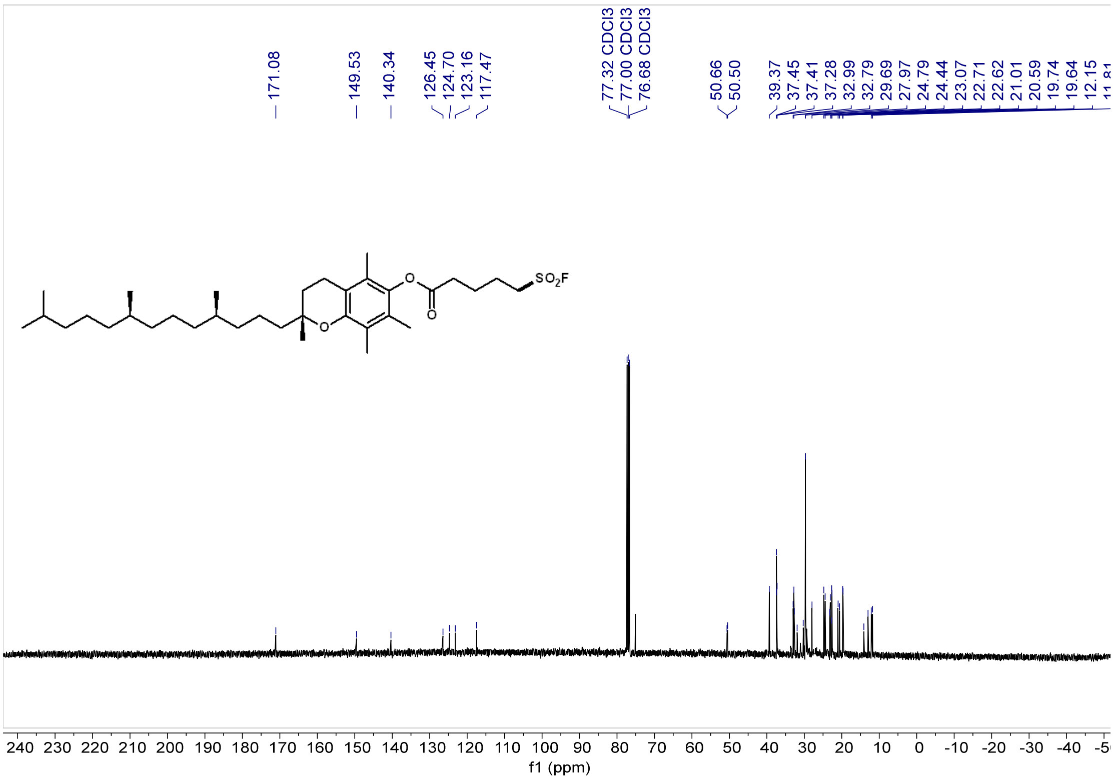

Supplementary Figure 171. ^{13}C NMR (101 MHz, room temperature, $CDCl_3$) spectra of product **6j**

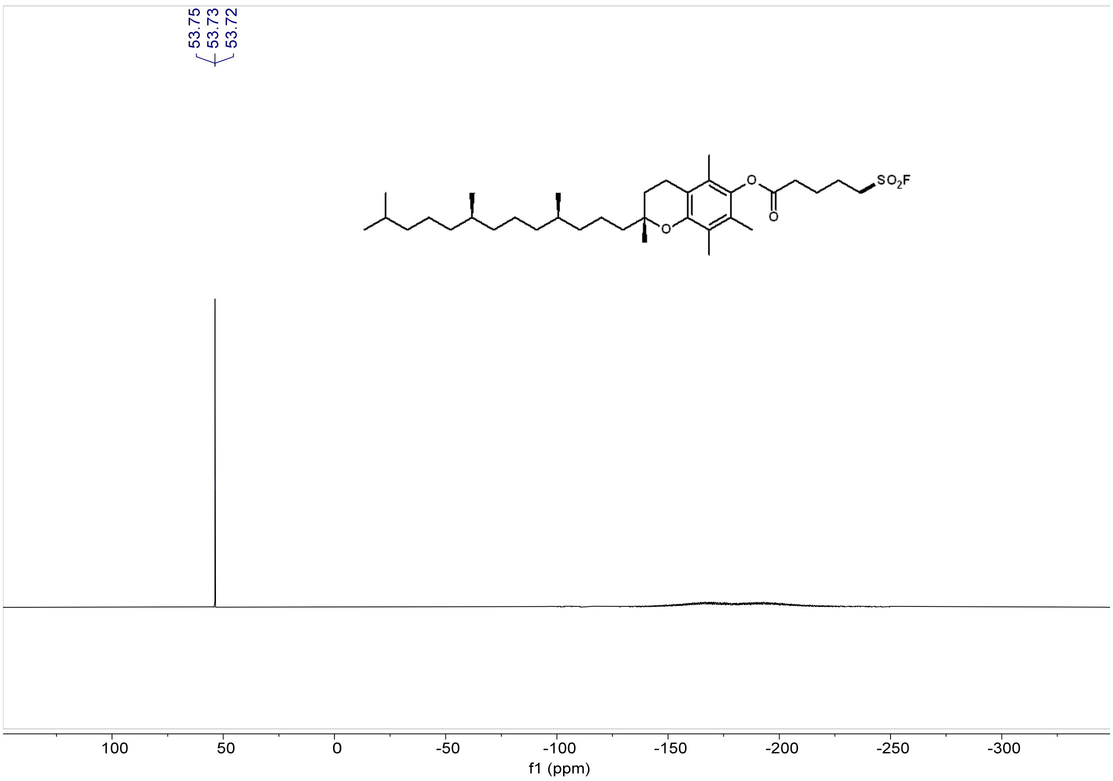

Supplementary Figure 172. ^{19}F NMR (376 MHz, room temperature, $CDCl_3$) spectra of product **6j**

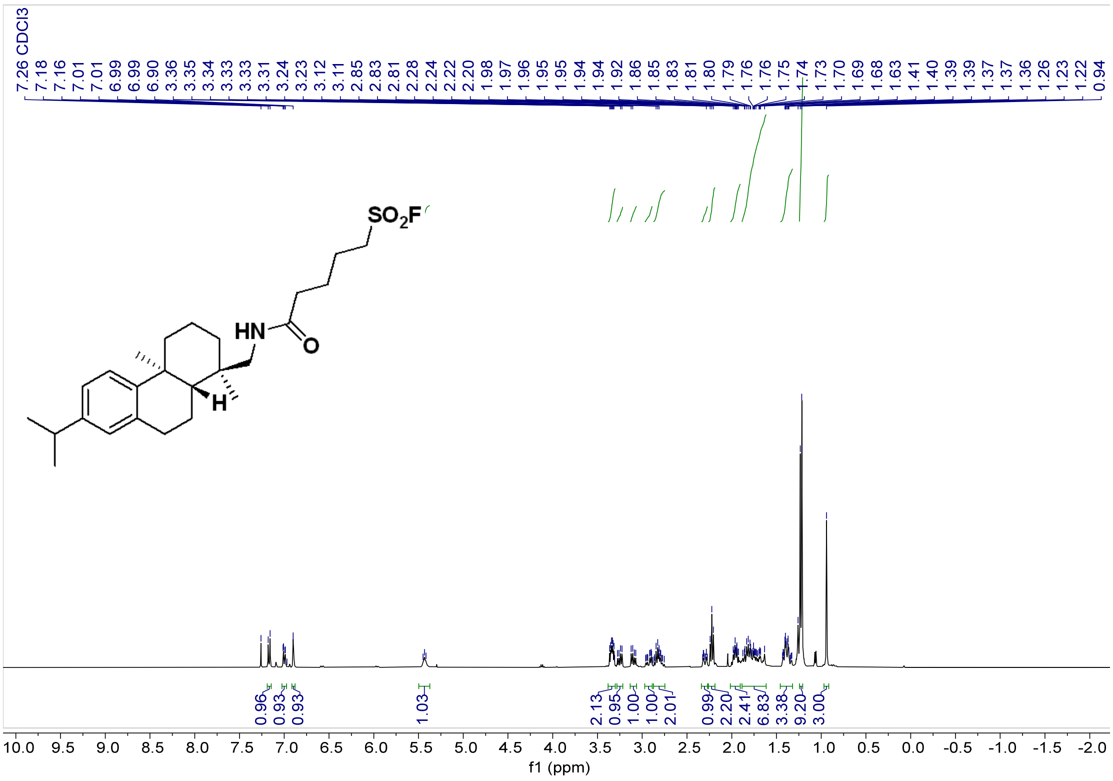

Supplementary Figure 173. ^{1}H NMR (400 MHz, room temperature, $CDCl_3$) spectra of product **6k**

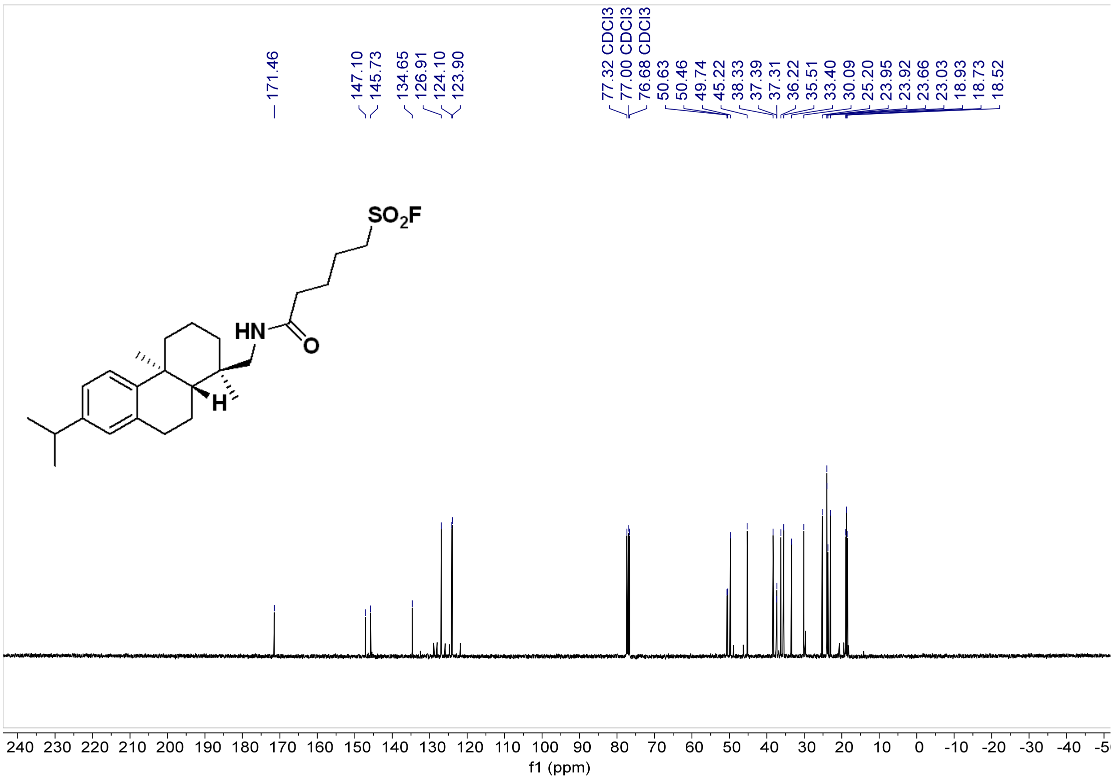

Supplementary Figure 174. ^{13}C NMR (101 MHz, room temperature, $CDCl_3$) spectra of product **6k**

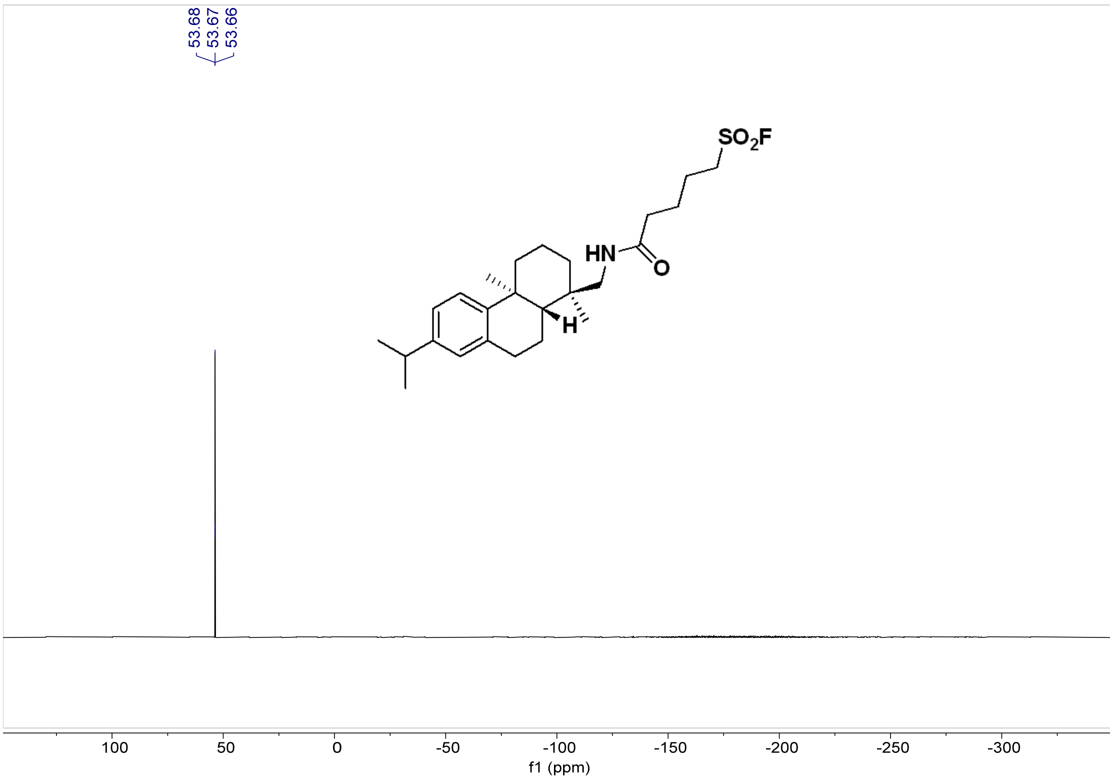

Supplementary Figure 175. ^{19}F NMR (376 MHz, room temperature, $CDCl_3$) spectra of product **6k**

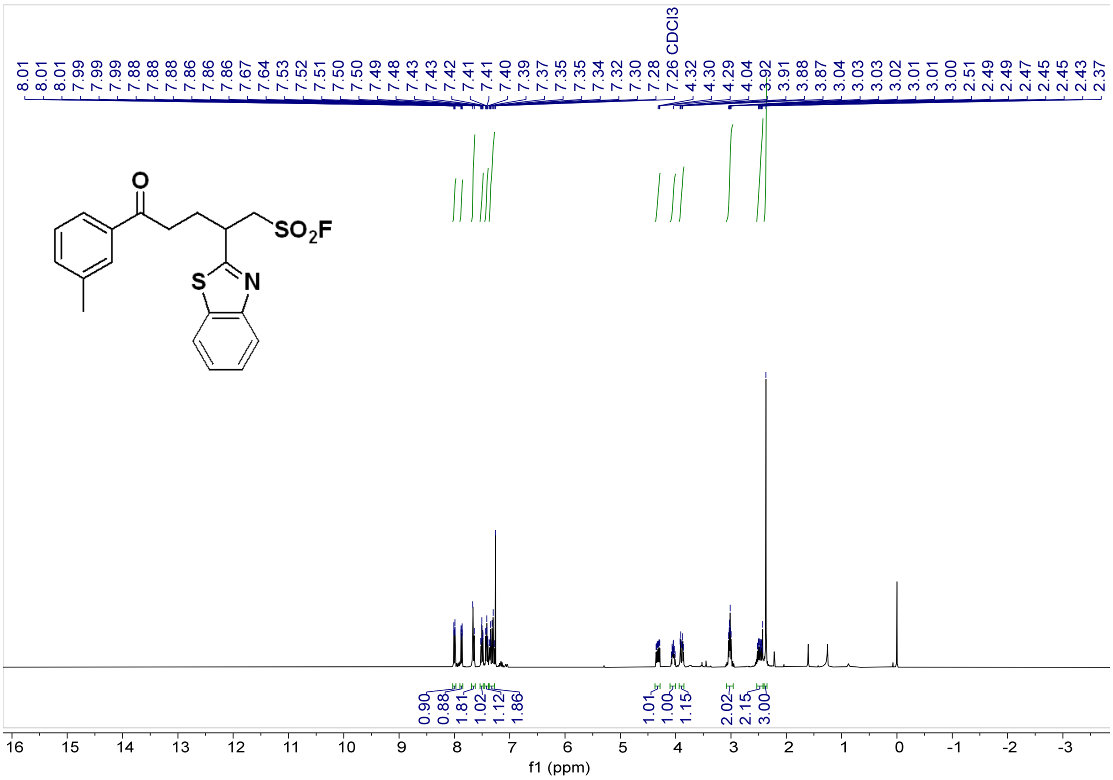

Supplementary Figure 176. ^{1}H NMR (400 MHz, room temperature, $CDCl_3$) spectra of product **8a**

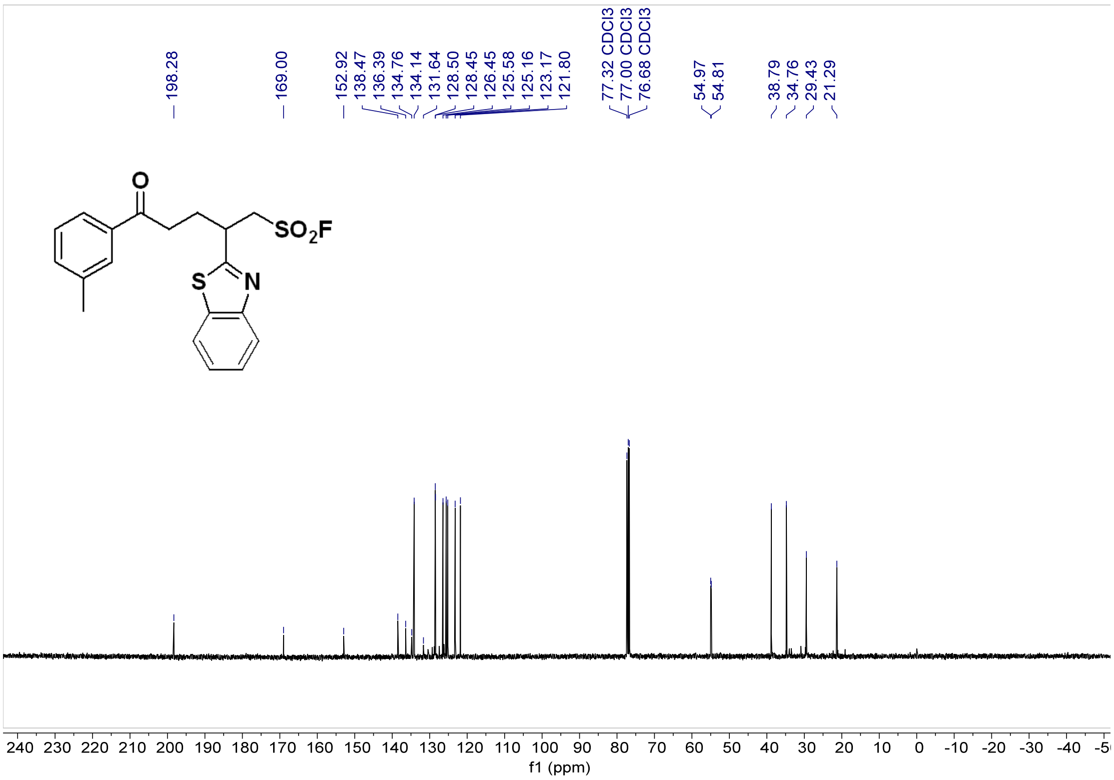

Supplementary Figure 177. ^{13}C NMR (101 MHz, room temperature, $CDCl_3$) spectra of product **8a**

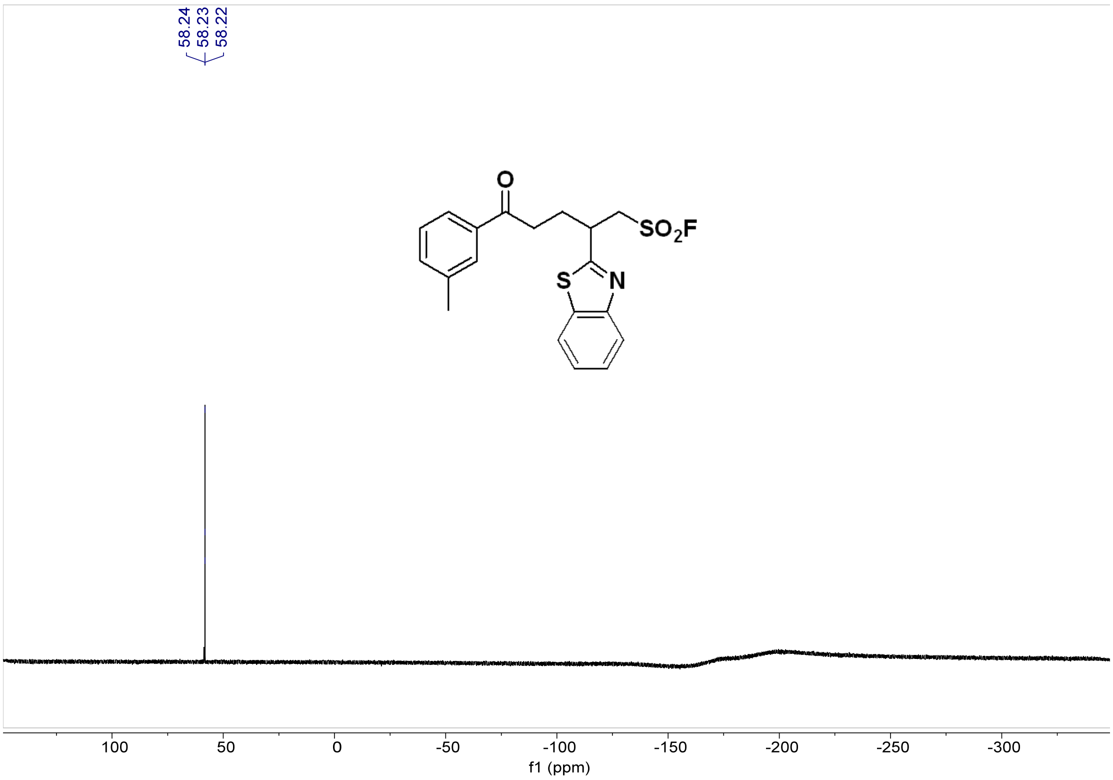

Supplementary Figure 178. ^{19}F NMR (376 MHz, room temperature, $CDCl_3$) spectra of product **8a**

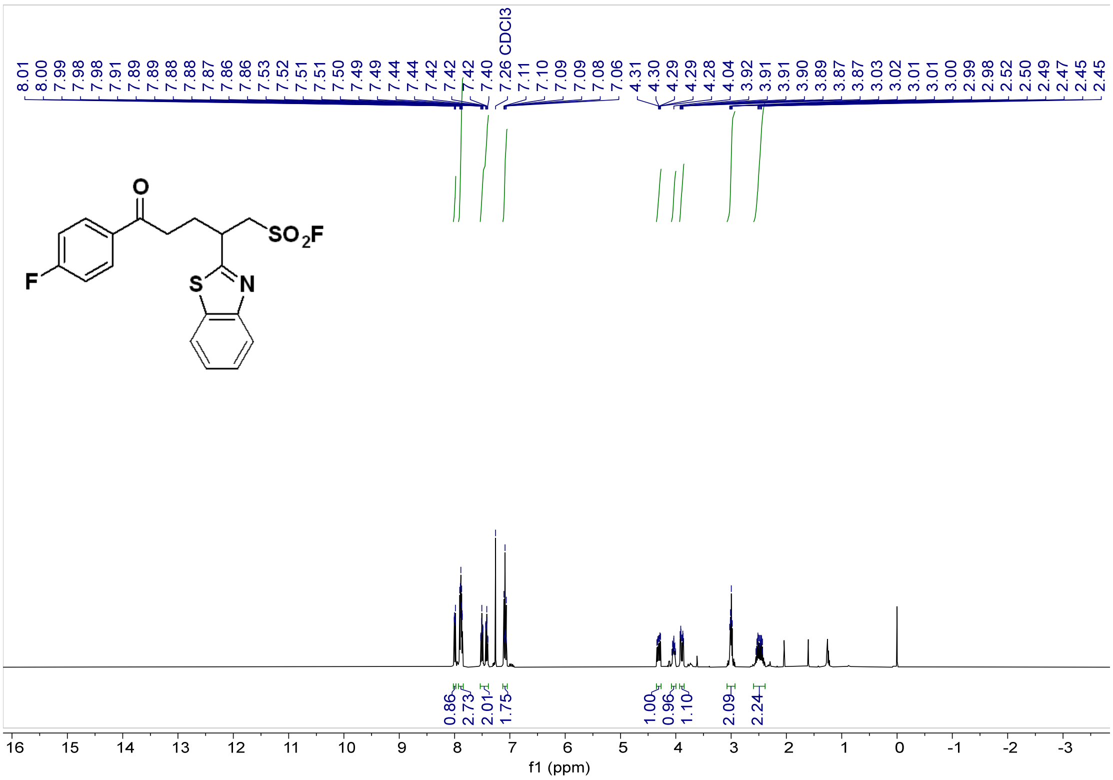

Supplementary Figure 179. ^{1}H NMR (400 MHz, room temperature, $CDCl_3$) spectra of product **8b**

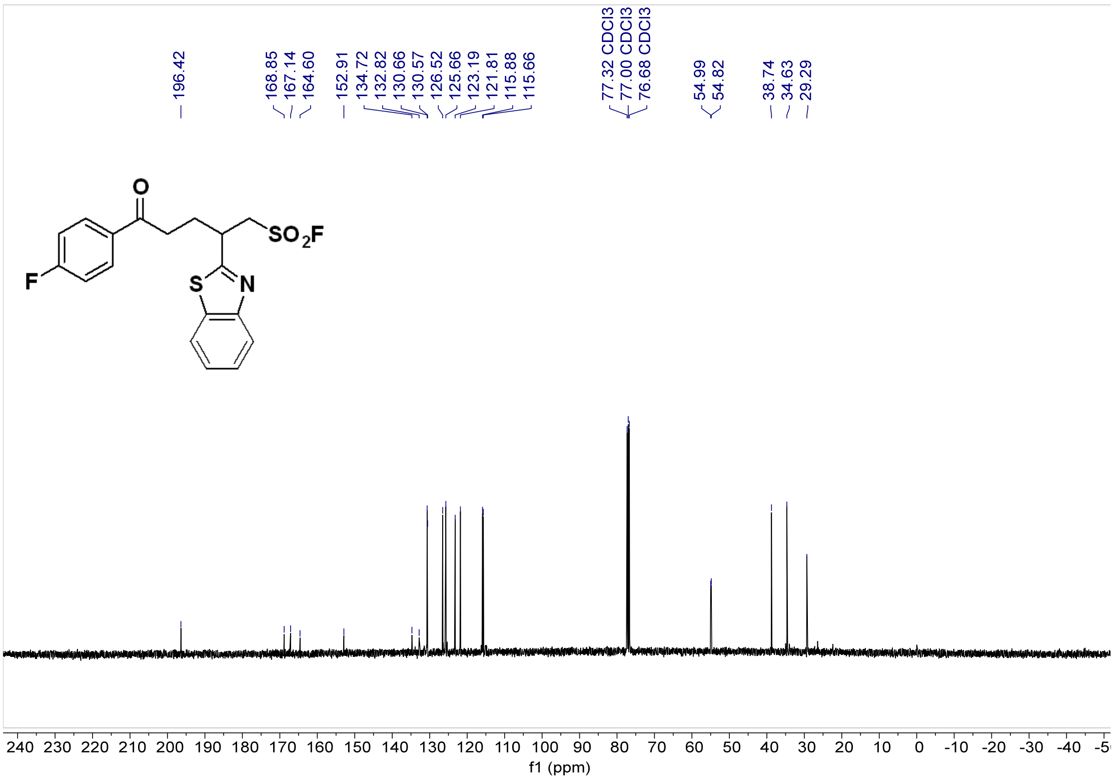

Supplementary Figure 180. ^{13}C NMR (101 MHz, room temperature, $CDCl_3$) spectra of product **8b**

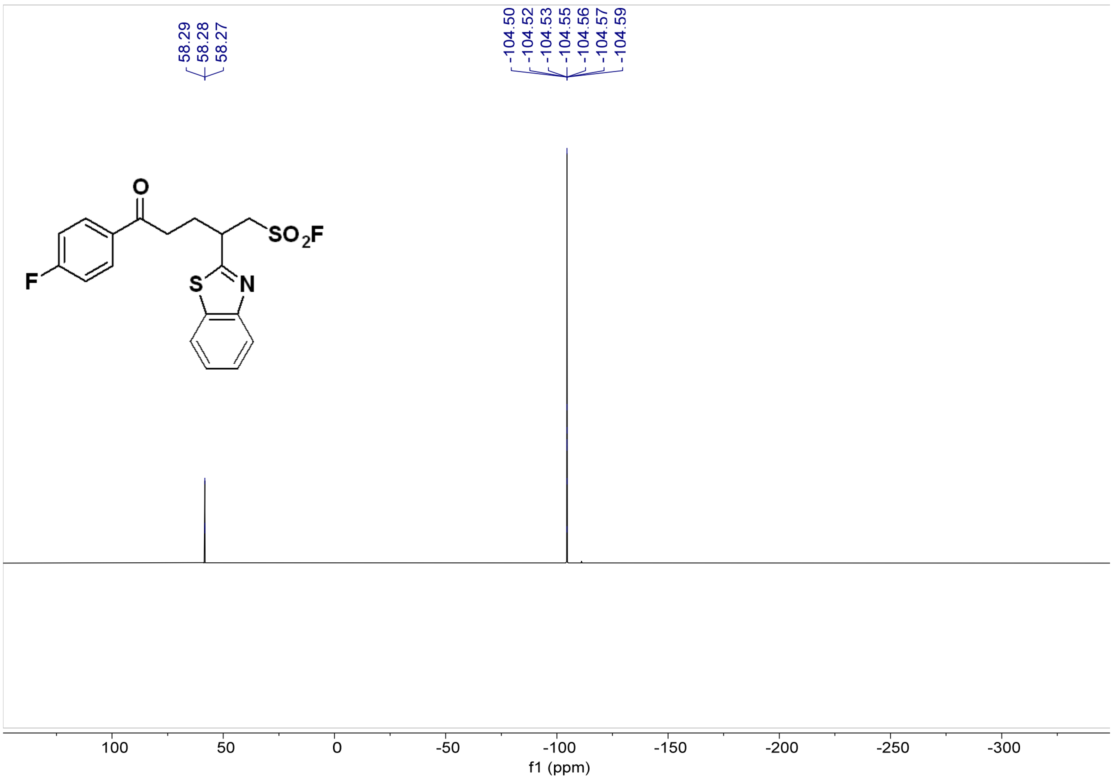

Supplementary Figure 181. ^{19}F NMR (376 MHz, room temperature, $CDCl_3$) spectra of product **8b**

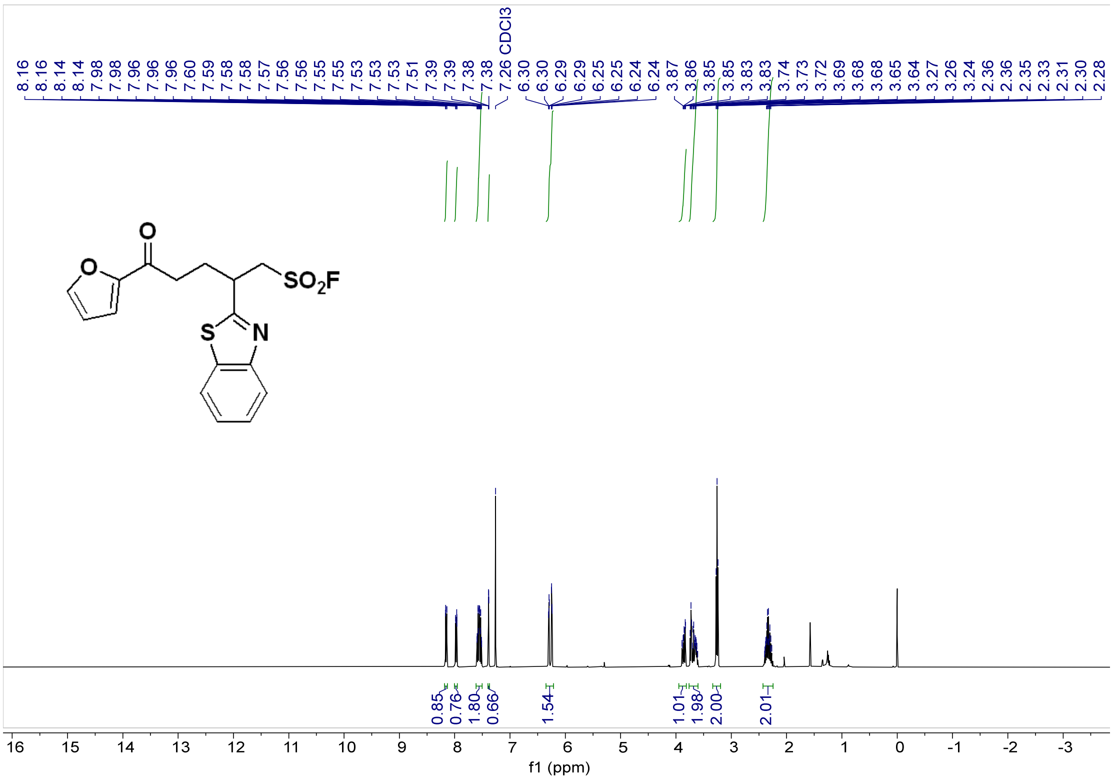

Supplementary Figure 182. ^{1}H NMR (400 MHz, room temperature, $CDCl_3$) spectra of product **8c**

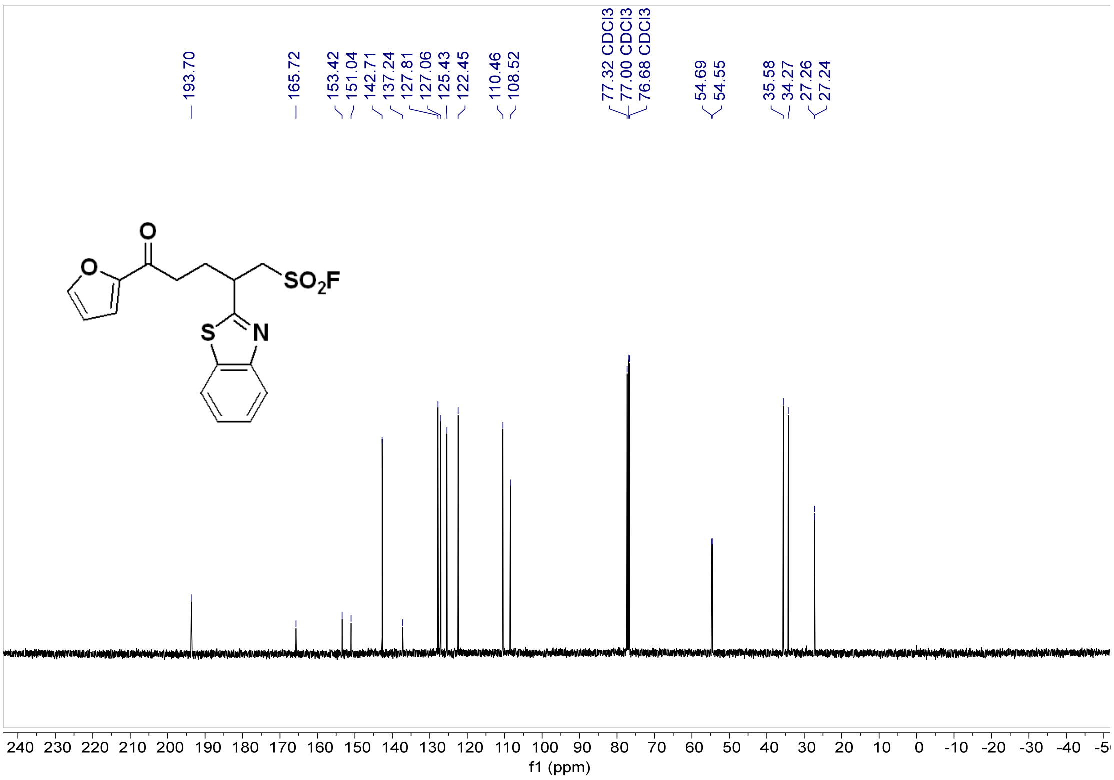

Supplementary Figure 183. ^{13}C NMR (101 MHz, room temperature, $CDCl_3$) spectra of product **8c**

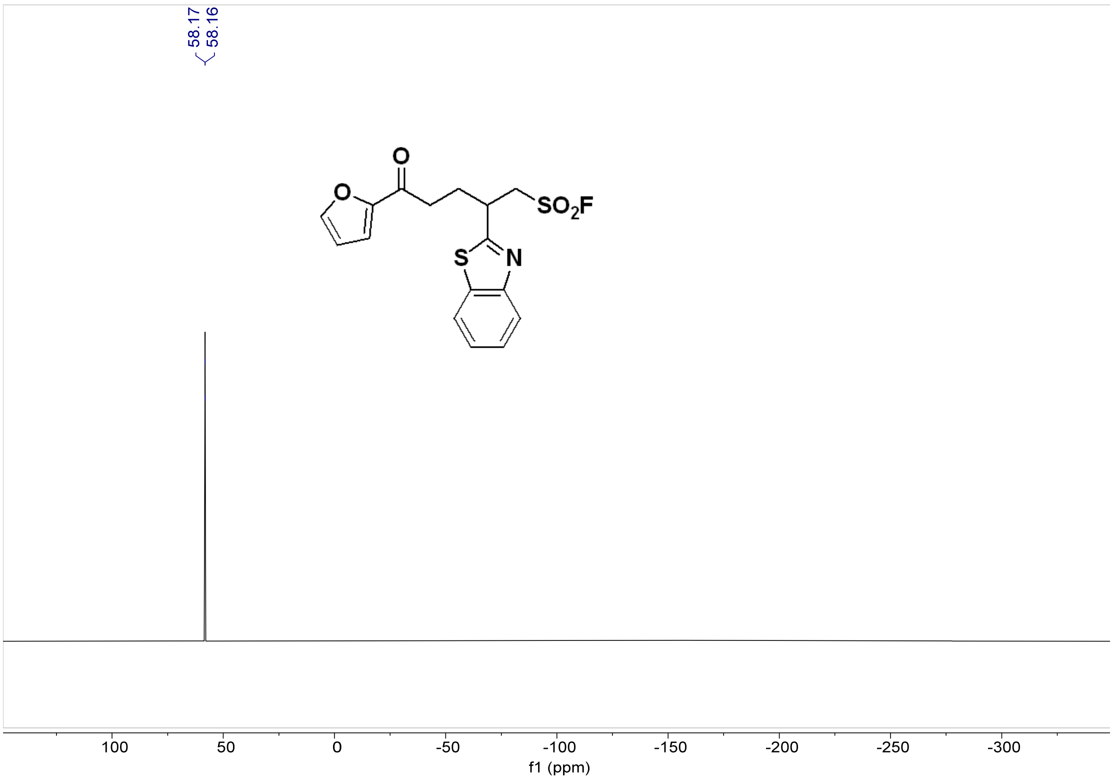

Supplementary Figure 184. ^{19}F NMR (376 MHz, room temperature, $CDCl_3$) spectra of product **8c**

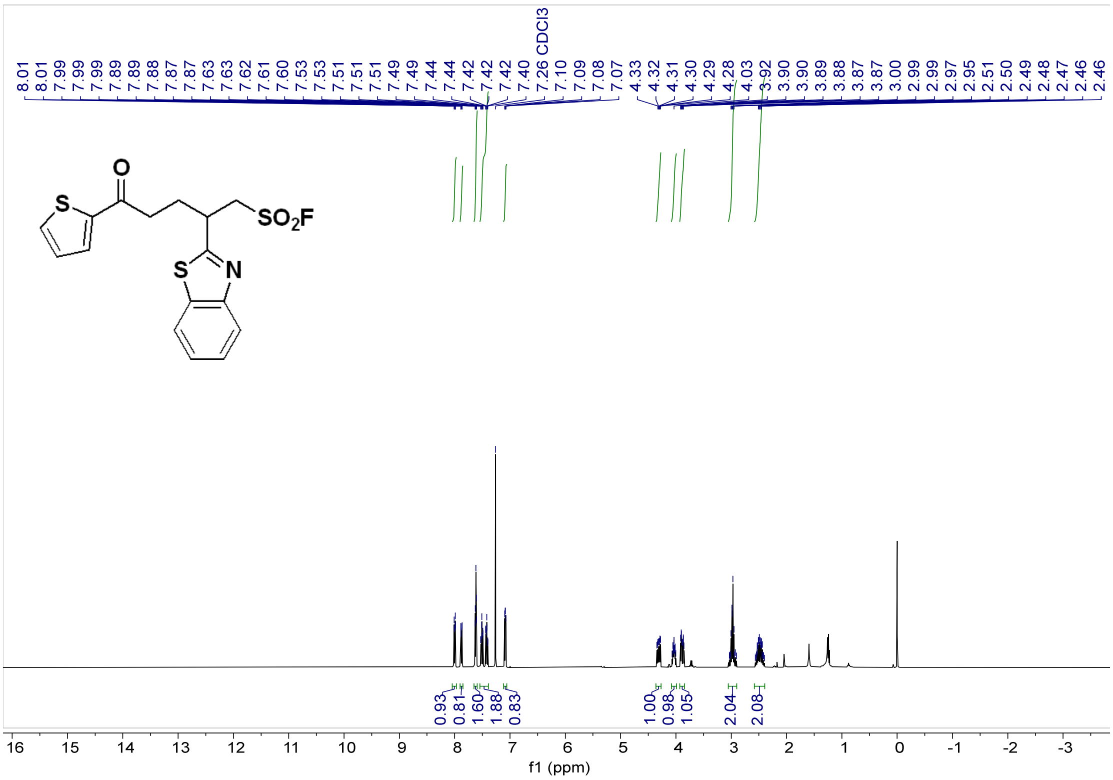

Supplementary Figure 185. ^{1}H NMR (400 MHz, room temperature, $CDCl_3$) spectra of product **8d**

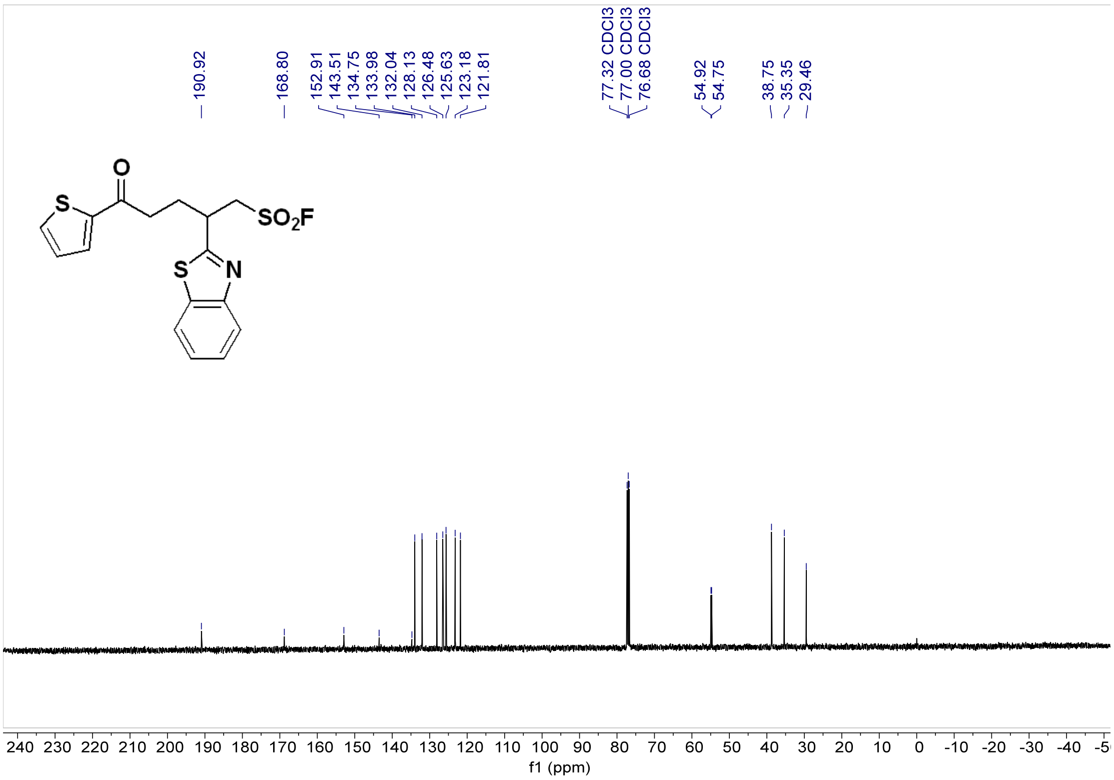

Supplementary Figure 186. ^{13}C (101 MHz, room temperature, $CDCl_3$) NMR spectra of product **8d**

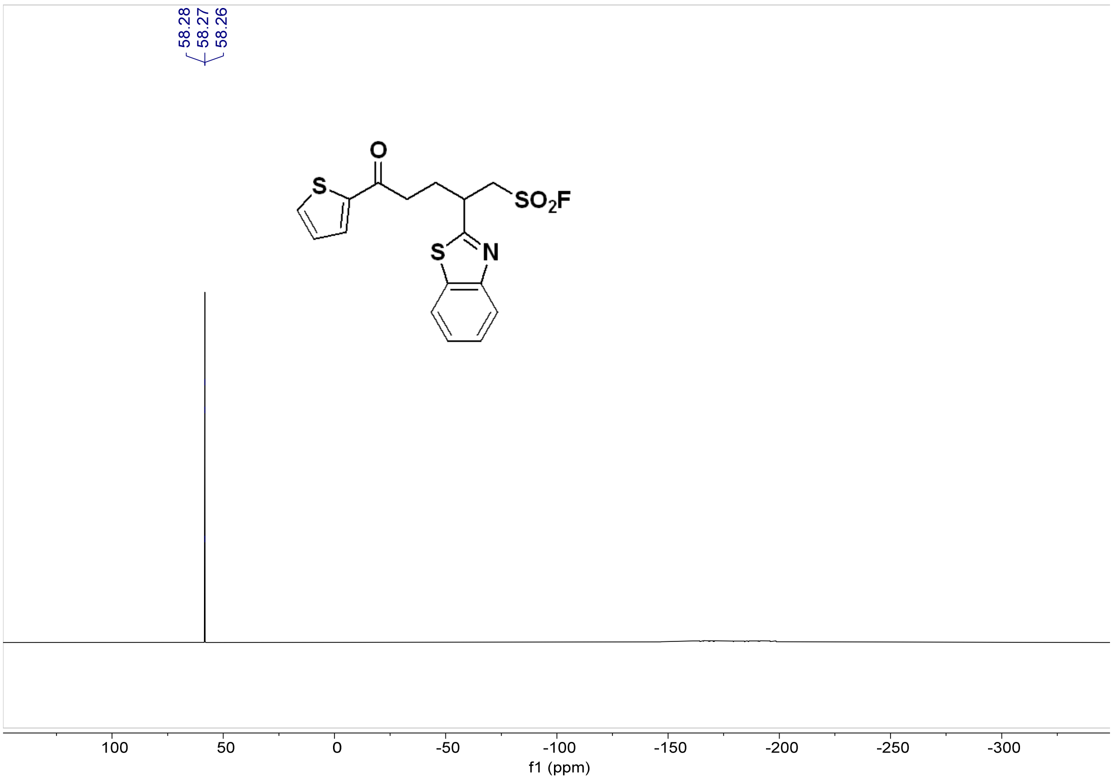

Supplementary Figure 187. ^{19}F NMR (376 MHz, room temperature, $CDCl_3$) spectra of product **8d**

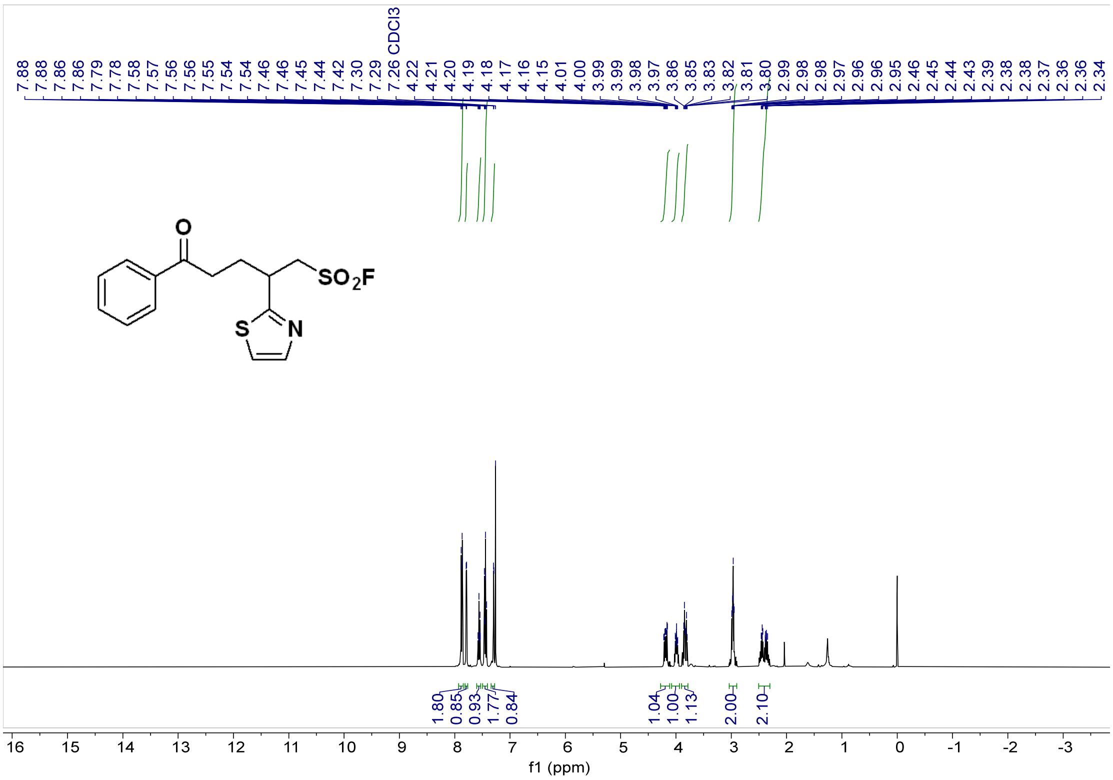

Supplementary Figure 188. ^{1}H NMR (400 MHz, room temperature, $CDCl_3$) spectra of product **8e**

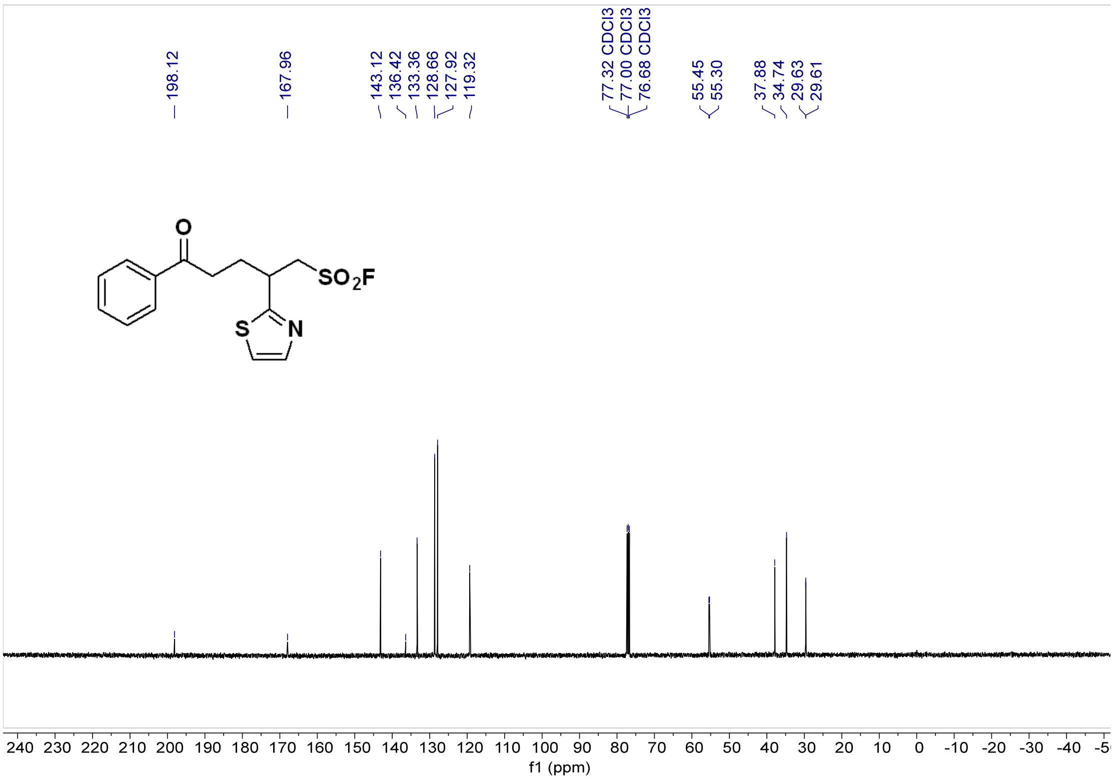

Supplementary Figure 189. ^{13}C NMR (101 MHz, room temperature, $CDCl_3$) spectra of product **8e**

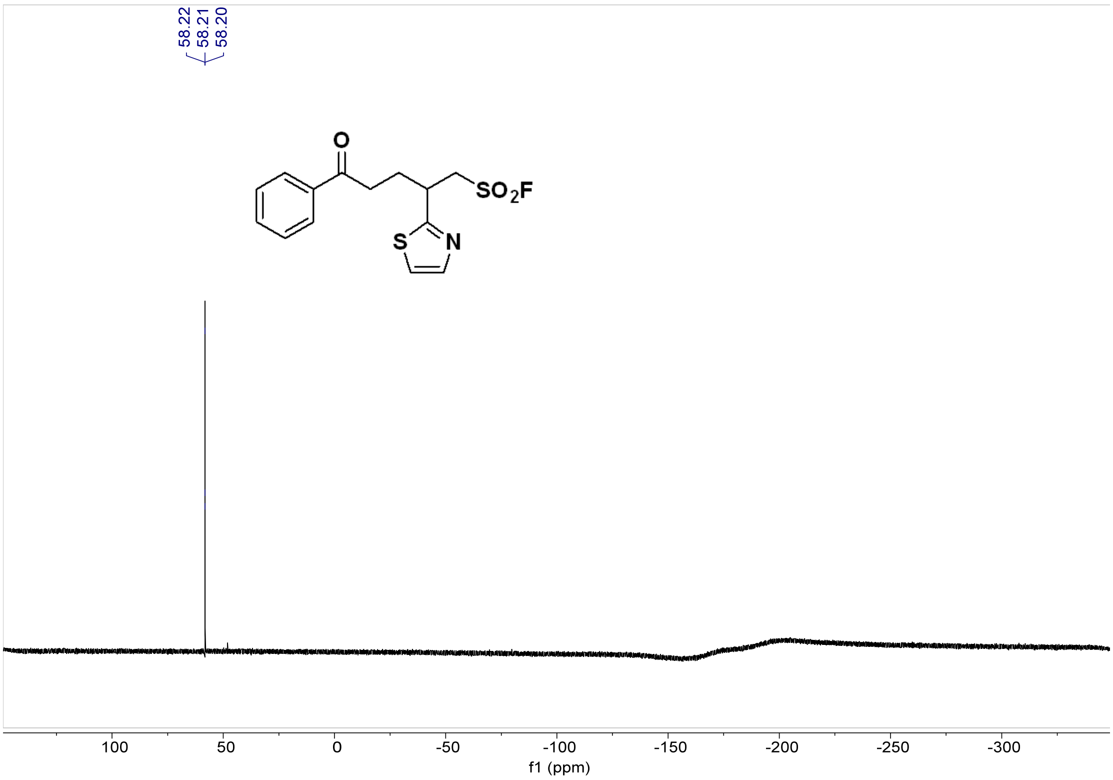

Supplementary Figure 190. ^{19}F NMR (376 MHz, room temperature, $CDCl_3$) spectra of product **8e**

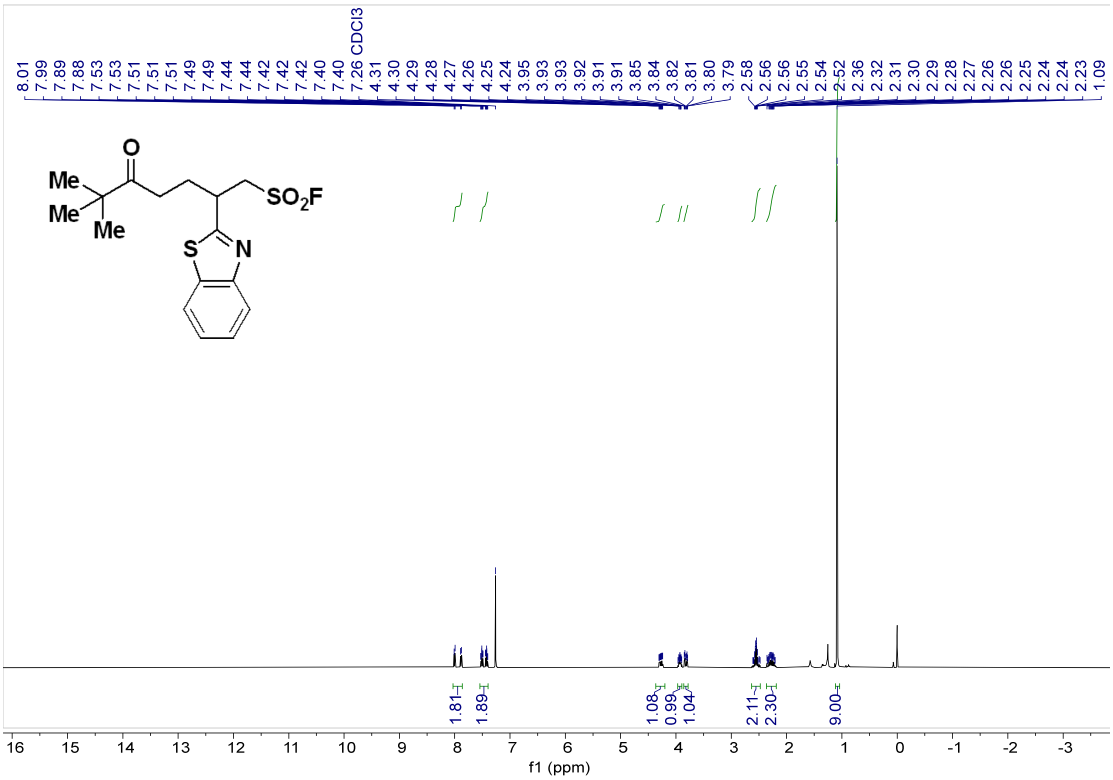

Supplementary Figure 191. ^{1}H NMR (400 MHz, room temperature, $CDCl_3$) spectra of product **8f**

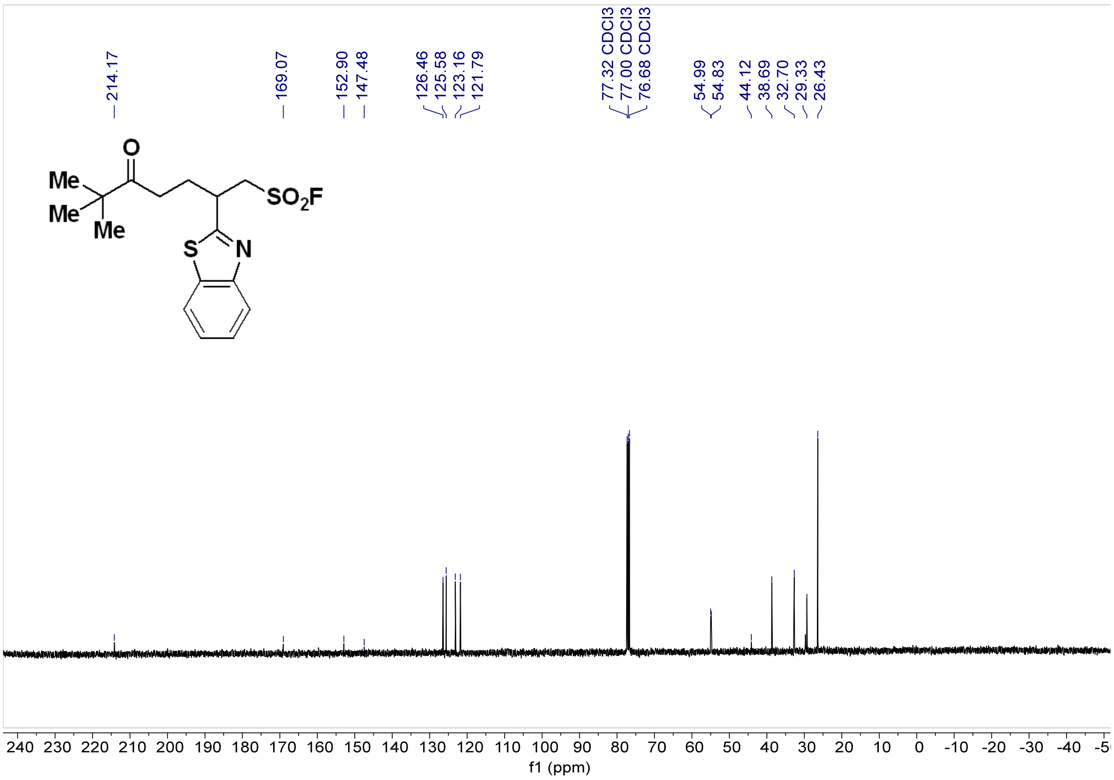

Supplementary Figure 192. ^{13}C NMR (101 MHz, room temperature, $CDCl_3$) spectra of product **8f**

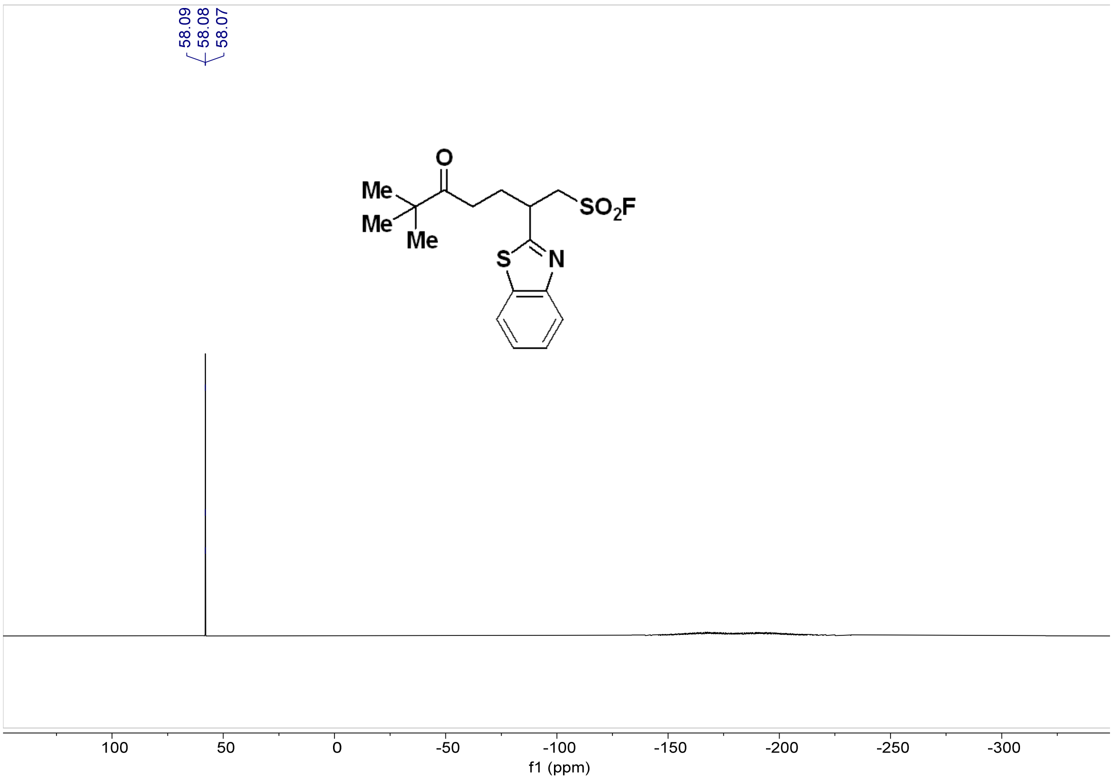

Supplementary Figure 193. ^{19}F NMR (376 MHz, room temperature, $CDCl_3$) spectra of product **8f**

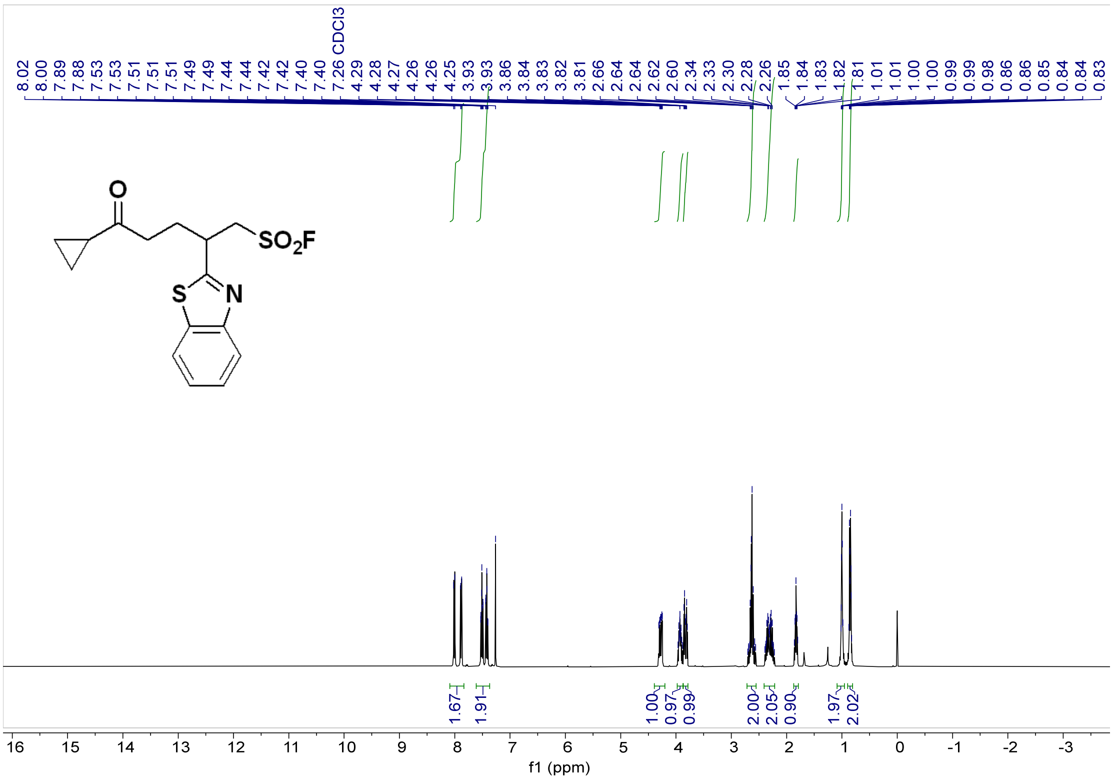

Supplementary Figure 194. ^{1}H NMR (400 MHz, room temperature, $CDCl_3$) spectra of product **8g**

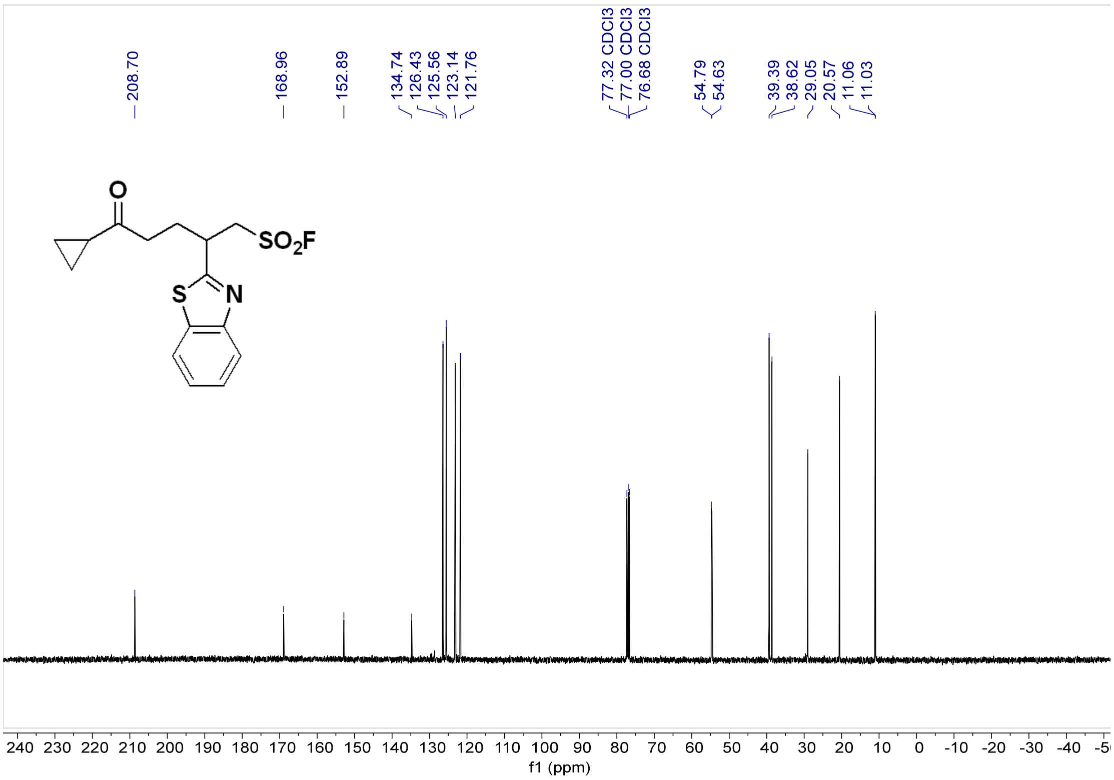

Supplementary Figure 195. ^{13}C NMR (101 MHz, room temperature, $CDCl_3$) spectra of product **8g**

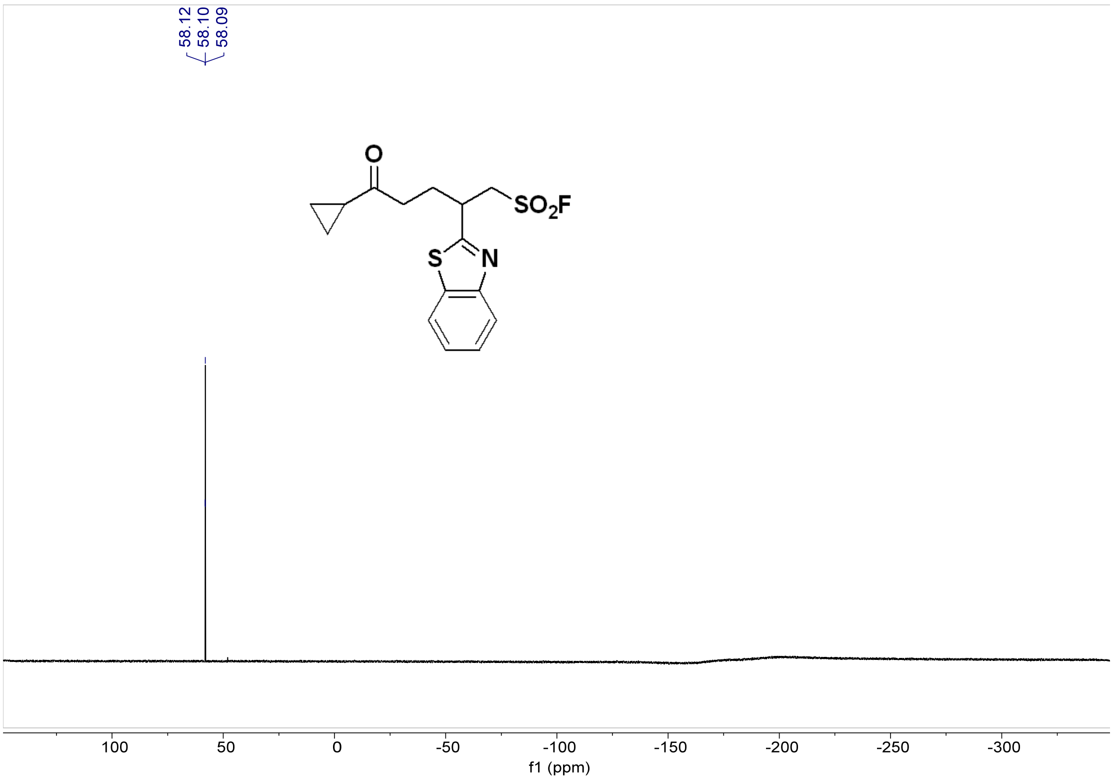

Supplementary Figure 196. ^{19}F NMR (376 MHz, room temperature, $CDCl_3$) spectra of product **8g**

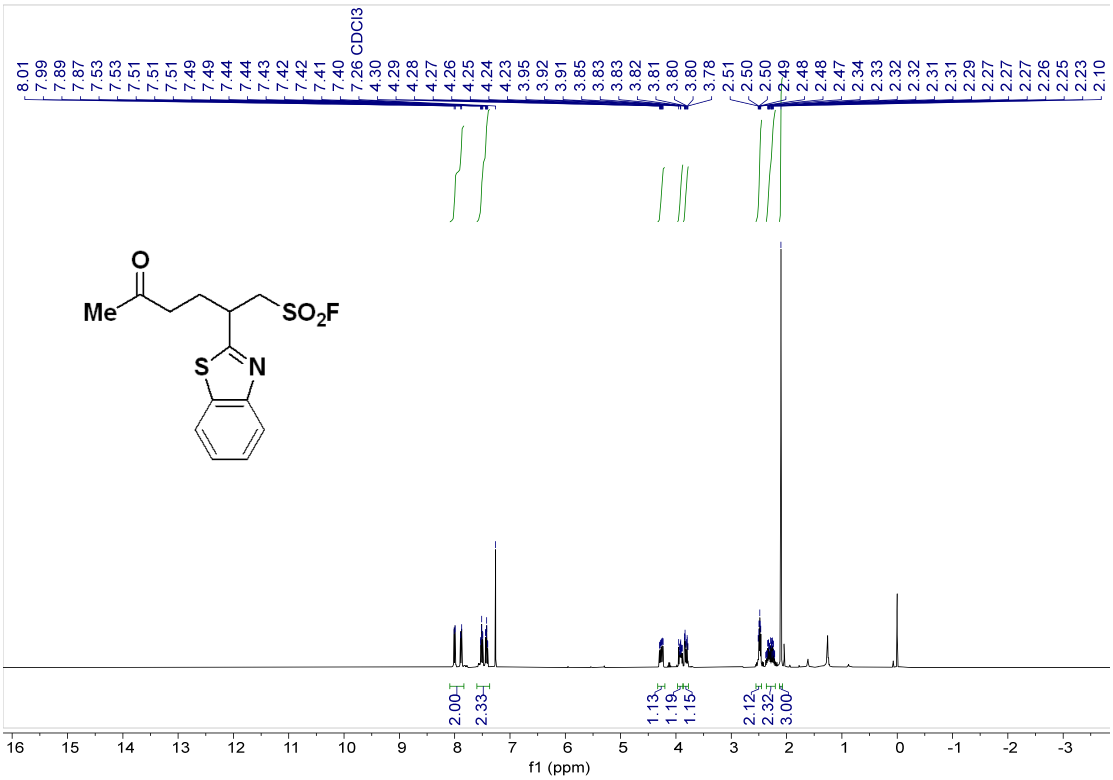

Supplementary Figure 197. ^{1}H NMR (400 MHz, room temperature, $CDCl_3$) spectra of product **8h**

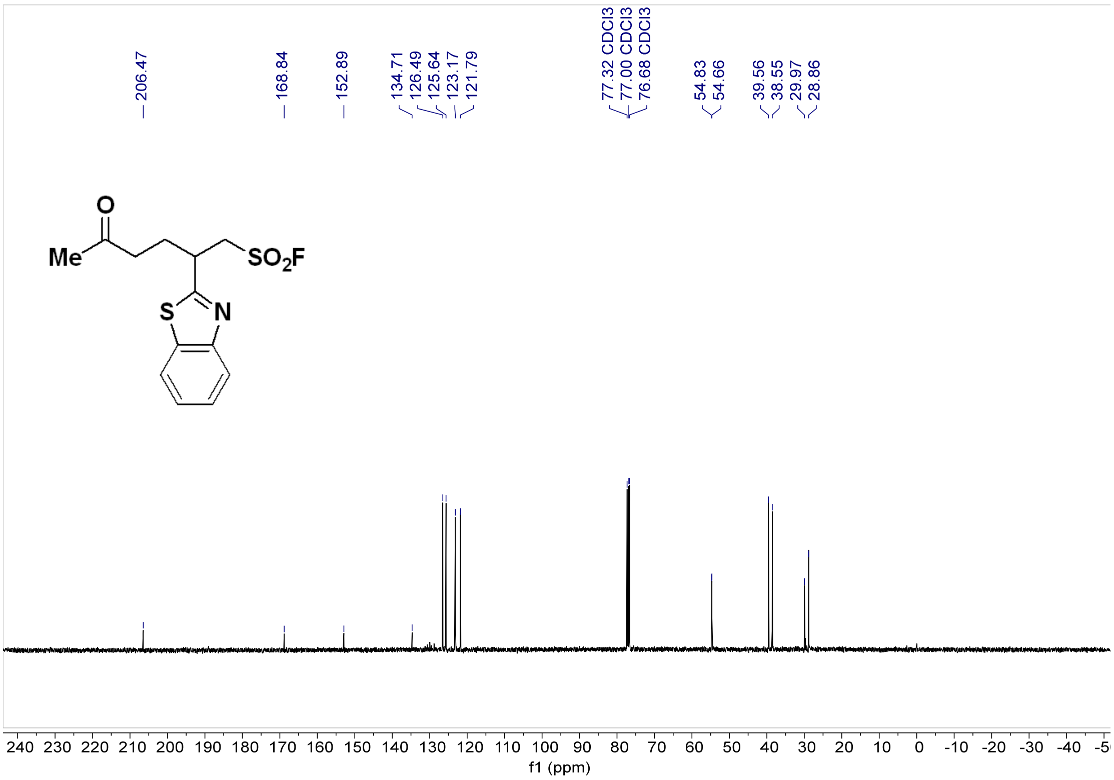

Supplementary Figure 198. ^{13}C NMR (101 MHz, room temperature, $CDCl_3$) spectra of product **8h**

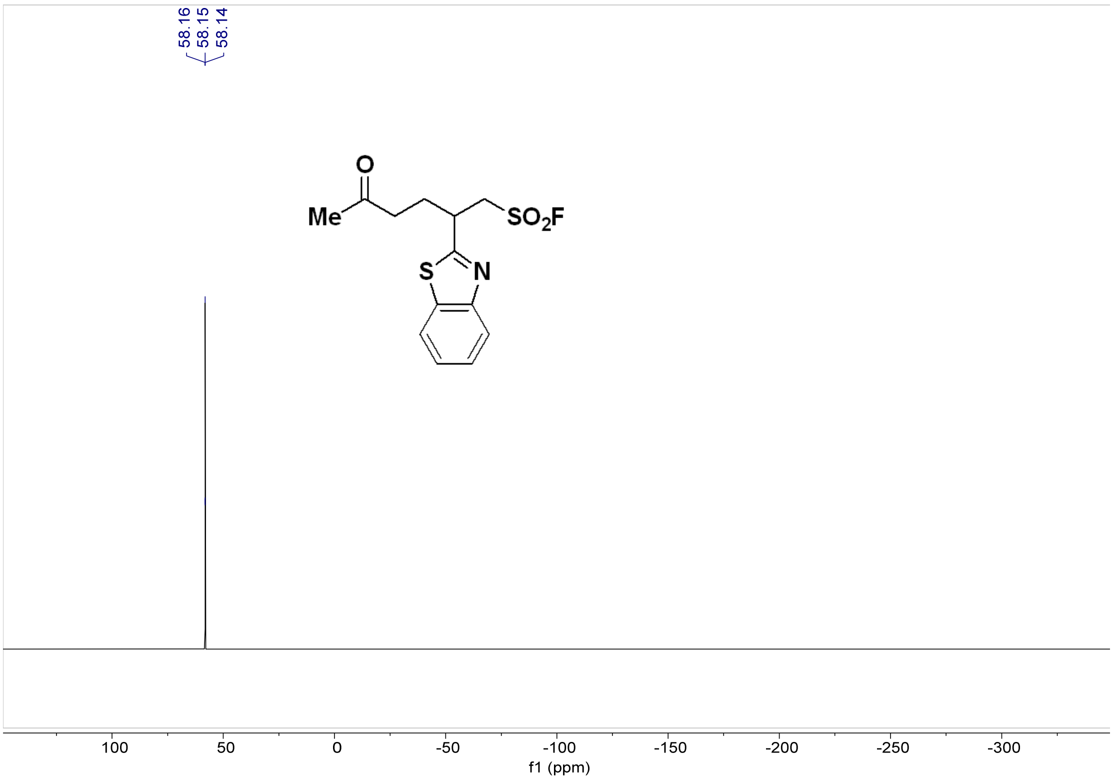

Supplementary Figure 199. ^{19}F NMR (376 MHz, room temperature, $CDCl_3$) spectra of product **8h**

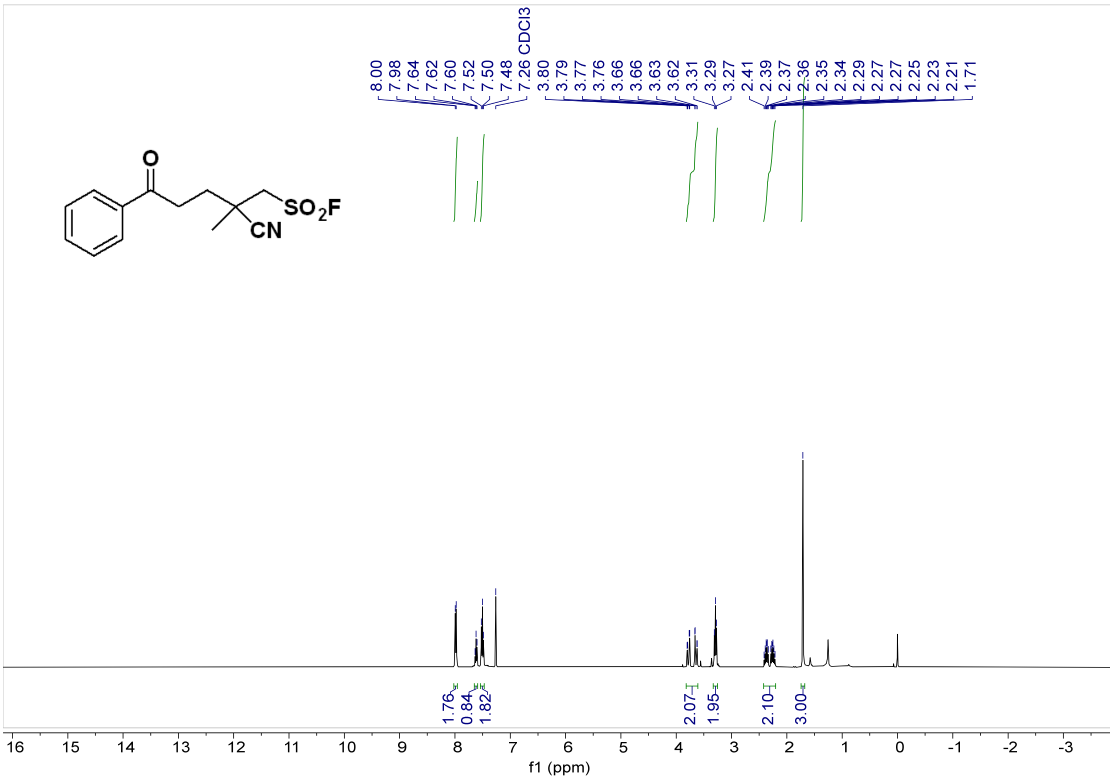

Supplementary Figure 200. ^{1}H NMR (400 MHz, room temperature, $CDCl_3$) spectra of product **8i**

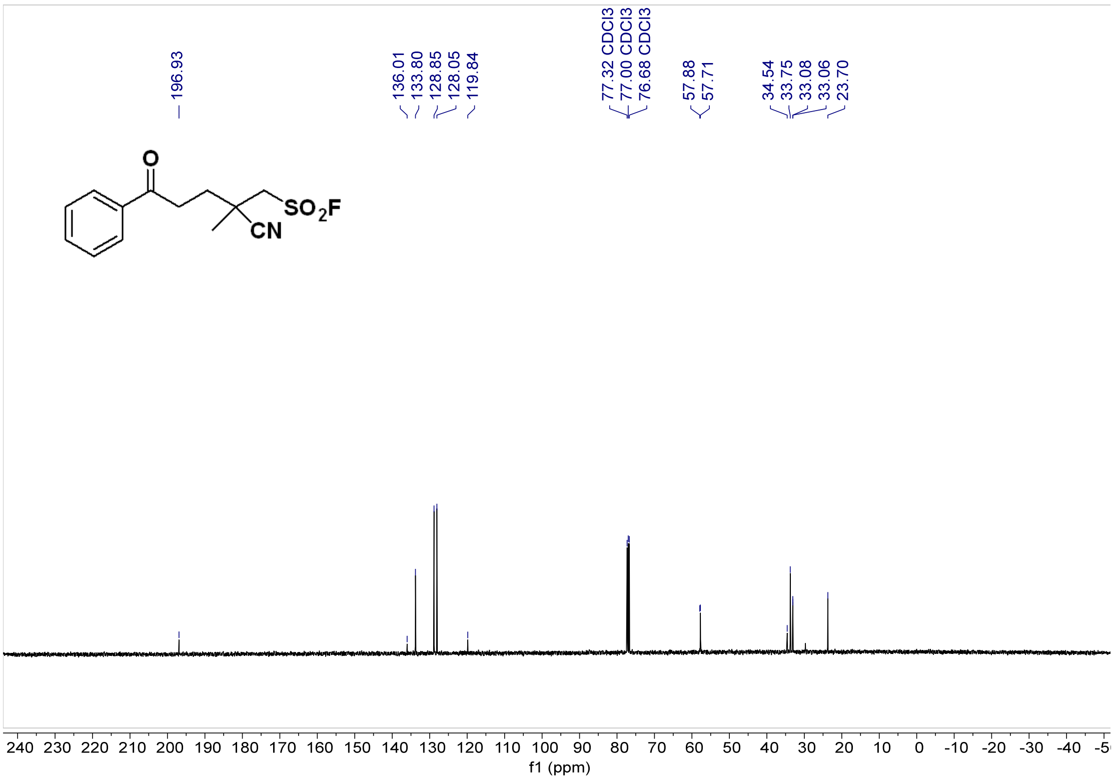

Supplementary Figure 201. ^{13}C NMR (101 MHz, room temperature, $CDCl_3$) spectra of product **8i**

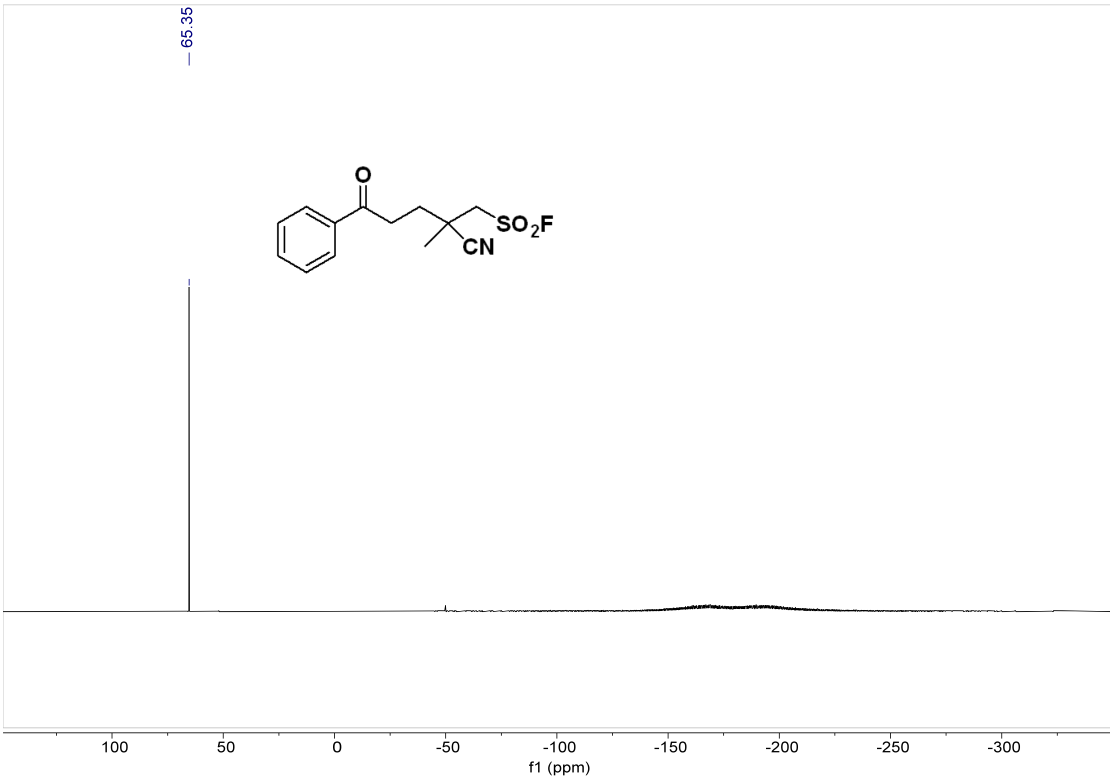

Supplementary Figure 202. ^{19}F NMR (376 MHz, room temperature, $CDCl_3$) spectra of product **8i**

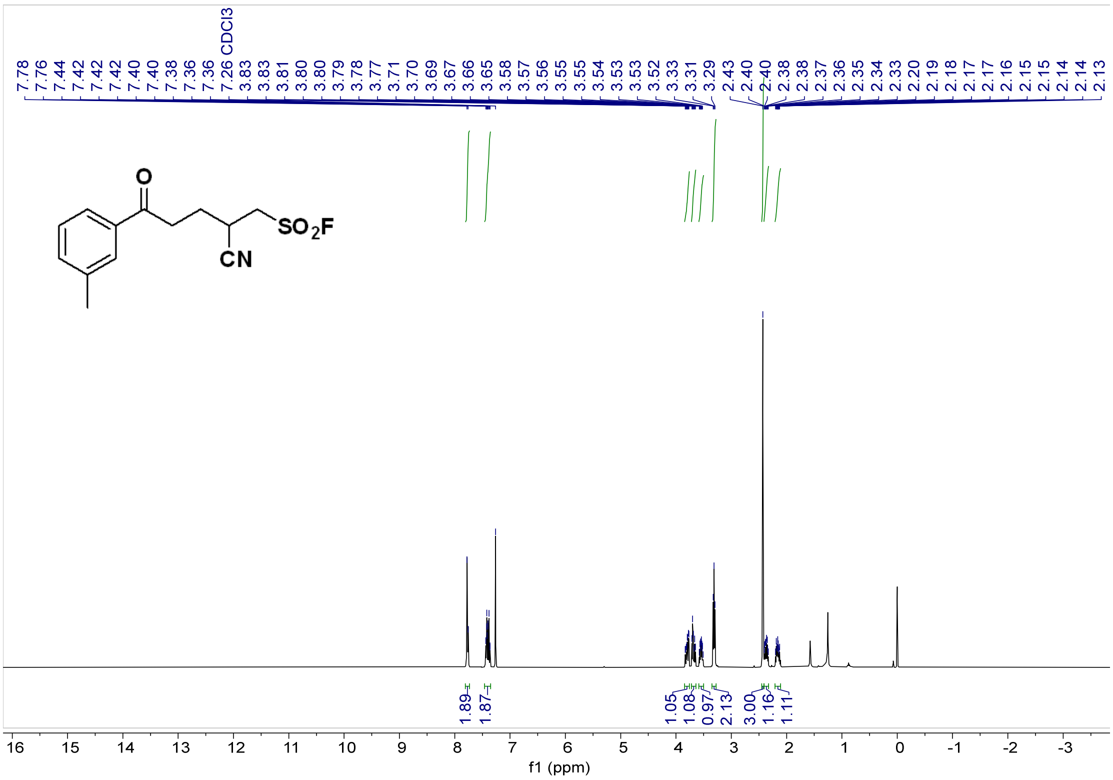

Supplementary Figure 203. ^{1}H NMR (400 MHz, room temperature, $CDCl_3$) spectra of product **8j**

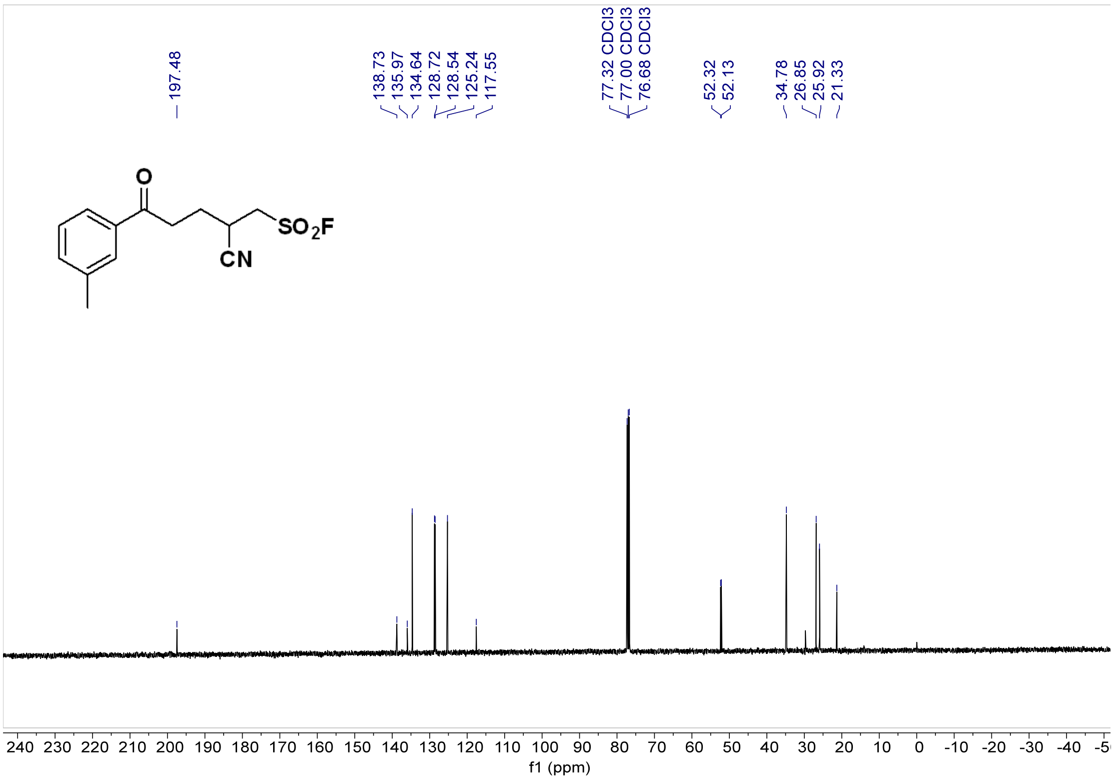

Supplementary Figure 204. ^{13}C NMR (101 MHz, room temperature, $CDCl_3$) spectra of product **8j**

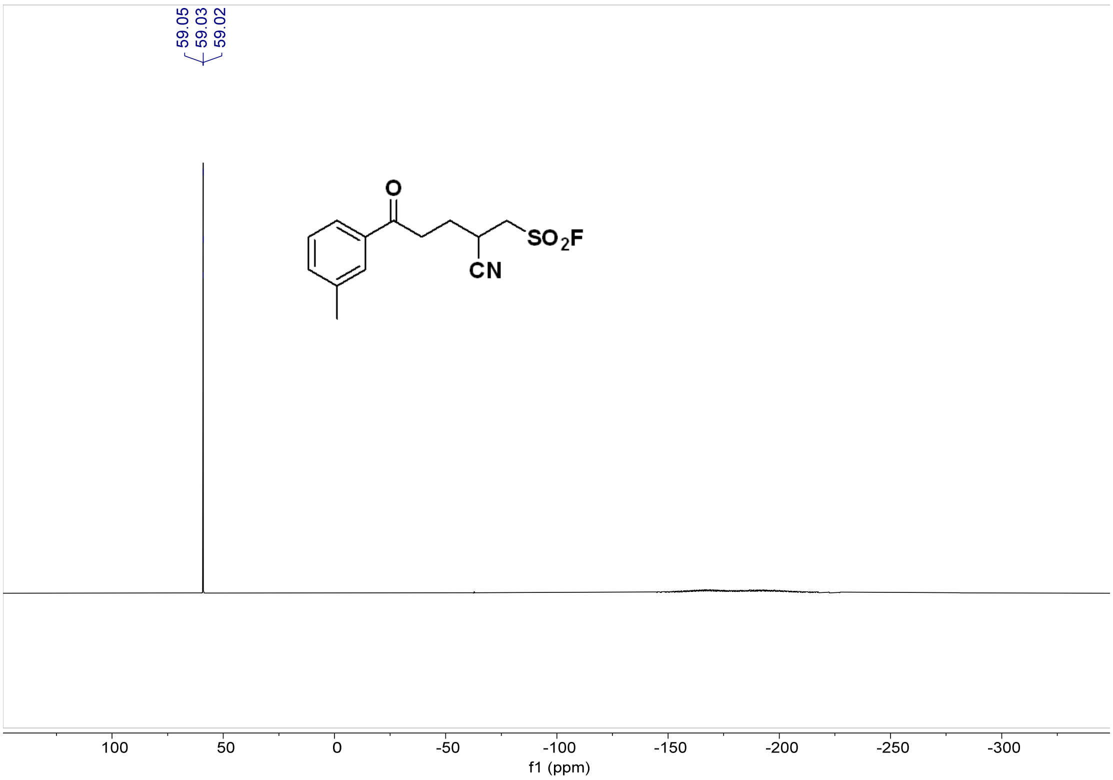

Supplementary Figure 205. ^{19}F NMR (376 MHz, room temperature, $CDCl_3$) spectra of product **8j**

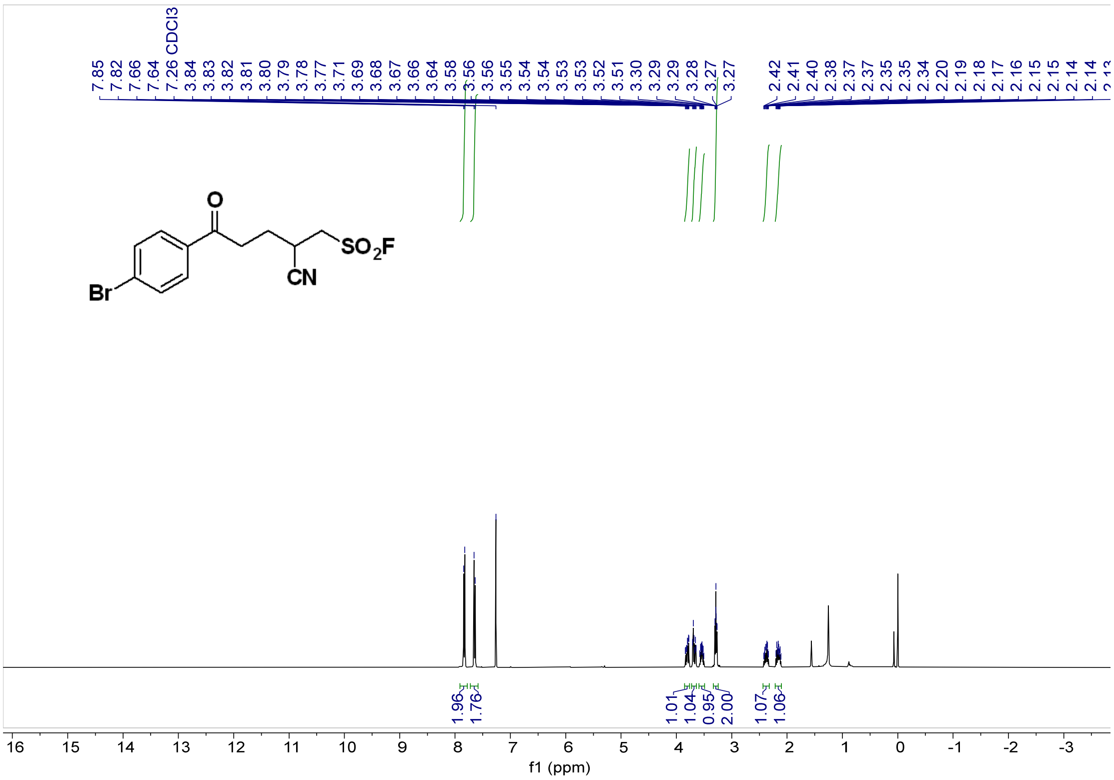

Supplementary Figure 206. ^{1}H NMR (400 MHz, room temperature, $CDCl_3$) spectra of product **8k**

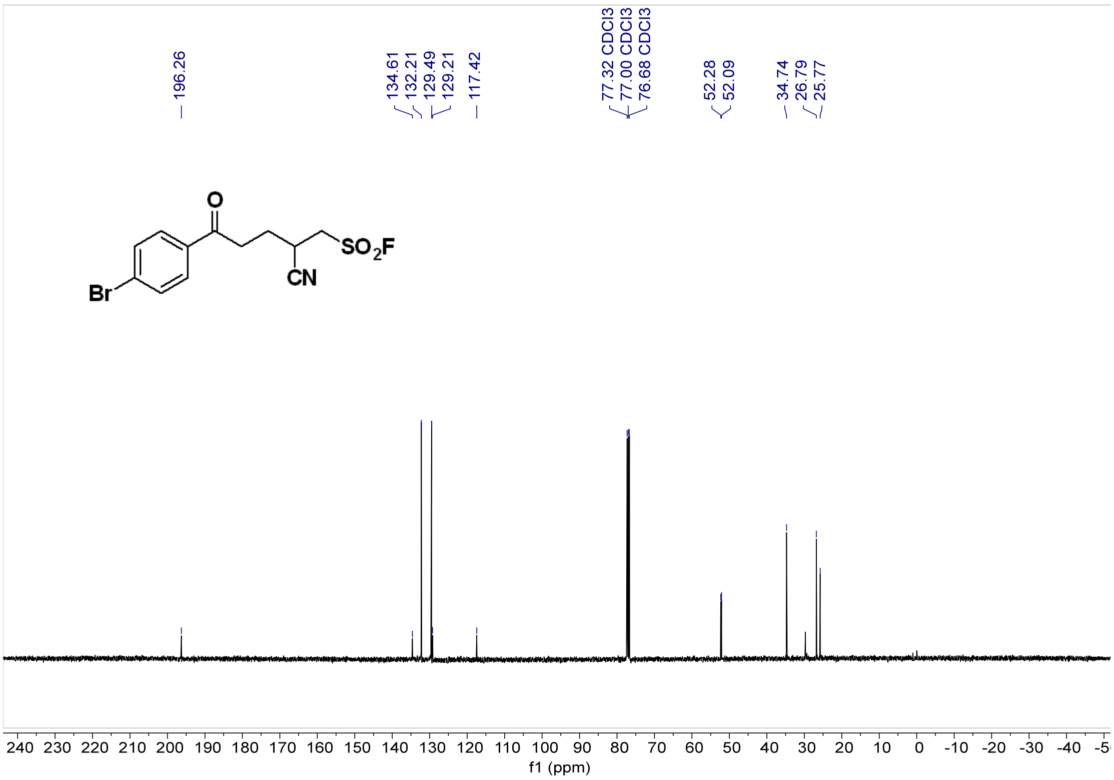

Supplementary Figure 207. ^{13}C NMR (101 MHz, room temperature, $CDCl_3$) spectra of product **8k**

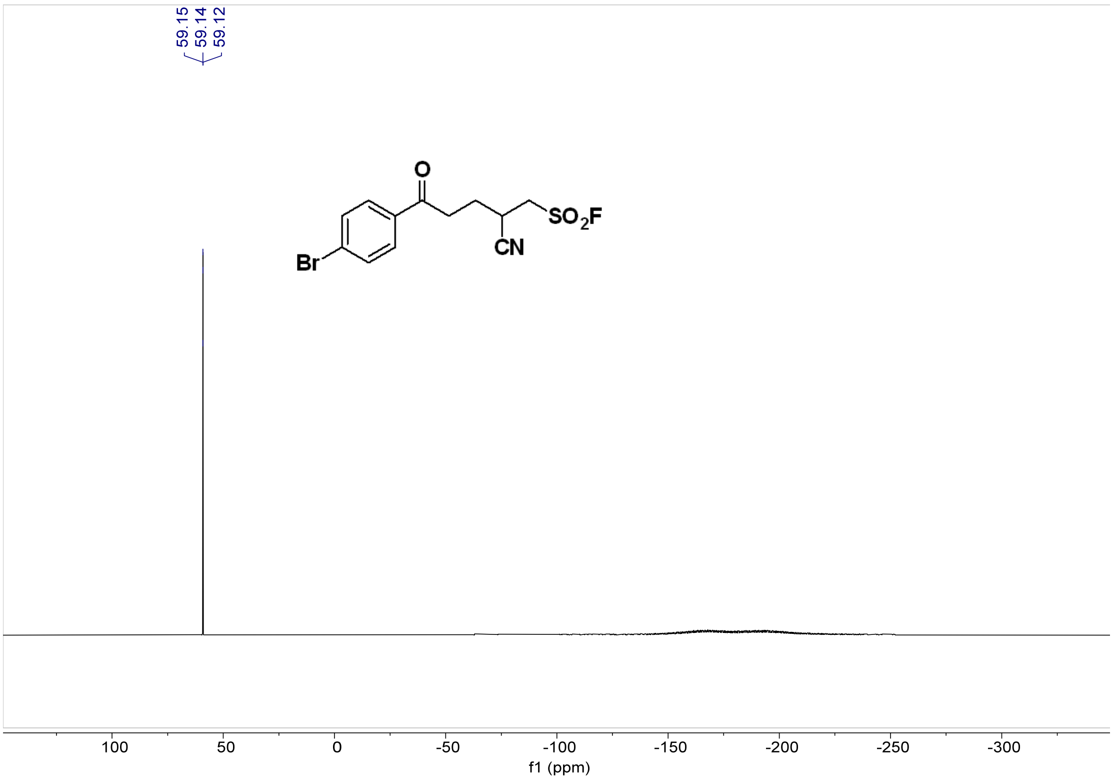

Supplementary Figure 208. ^{19}F NMR (376 MHz, room temperature, $CDCl_3$) spectra of product **8k**

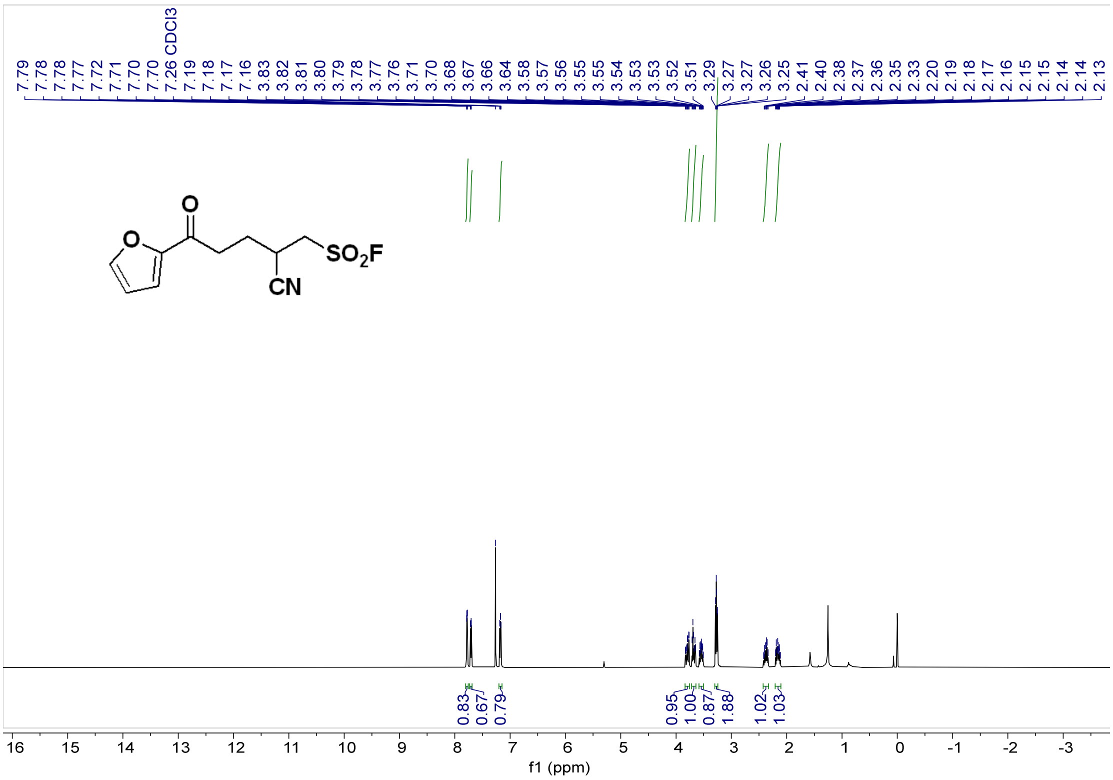

Supplementary Figure 209. ^{1}H NMR (400 MHz, room temperature, $CDCl_3$) spectra of product **8l**

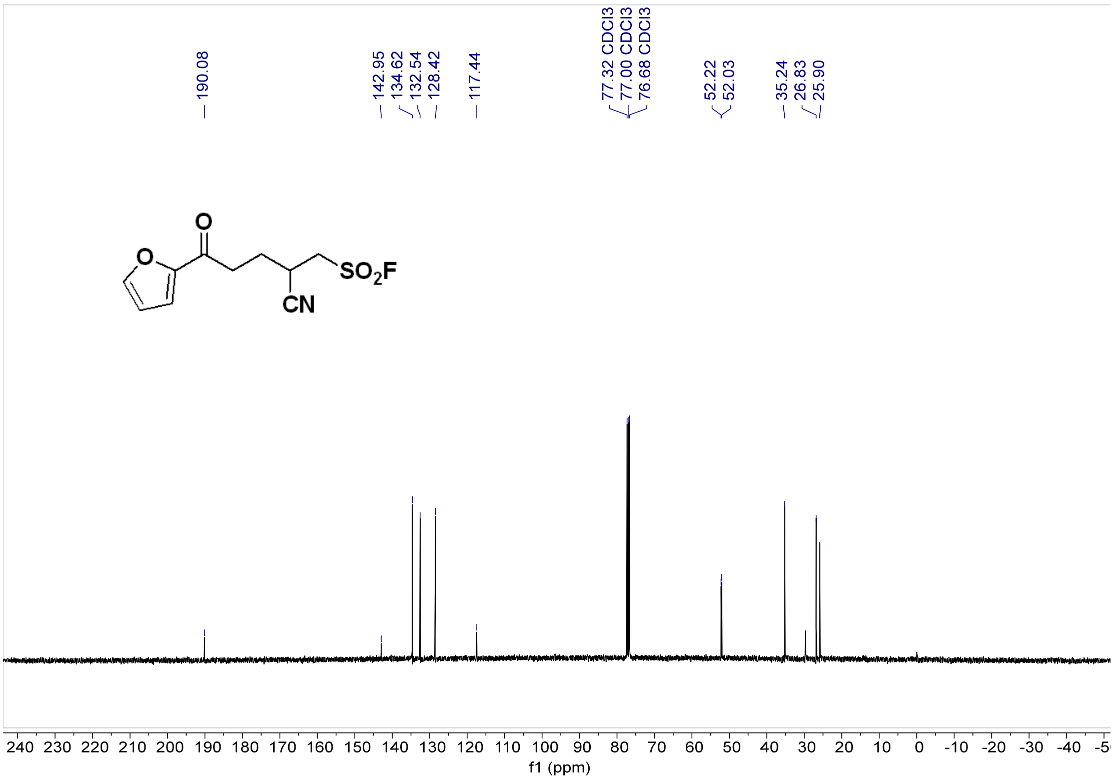

Supplementary Figure 210. ^{13}C NMR (101 MHz, room temperature, $CDCl_3$) spectra of product **8l**

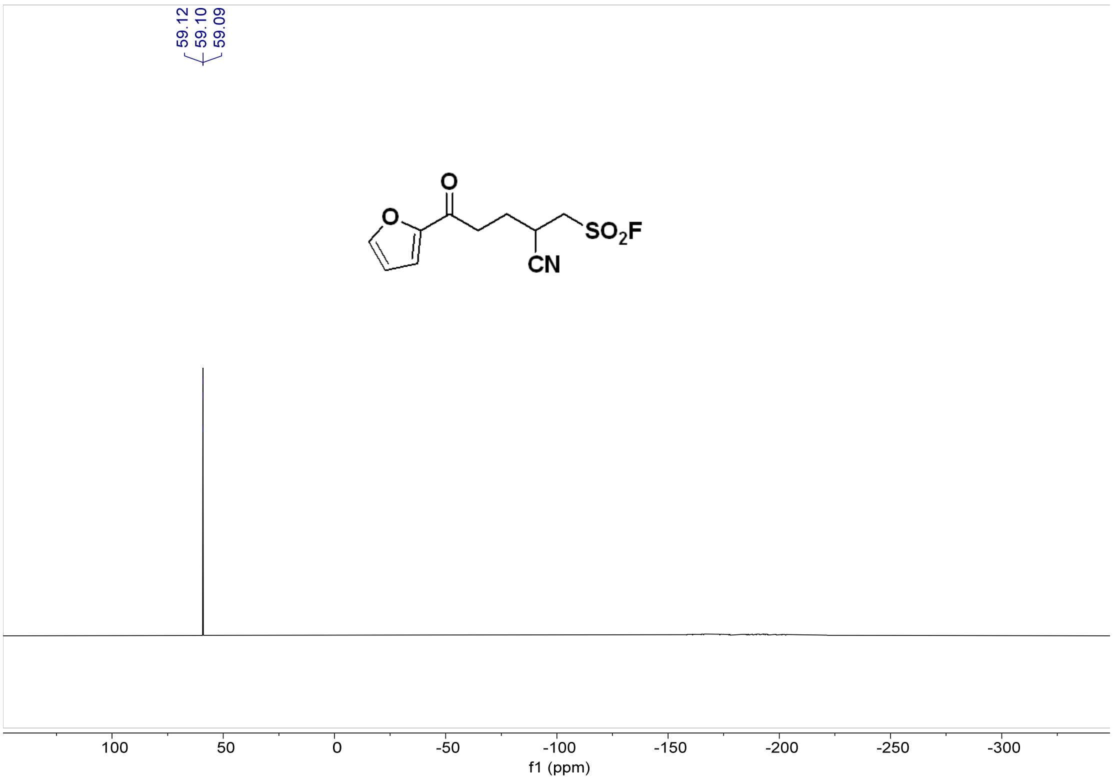

Supplementary Figure 211. ^{19}F NMR (376 MHz, room temperature, $CDCl_3$) spectra of product **8l**

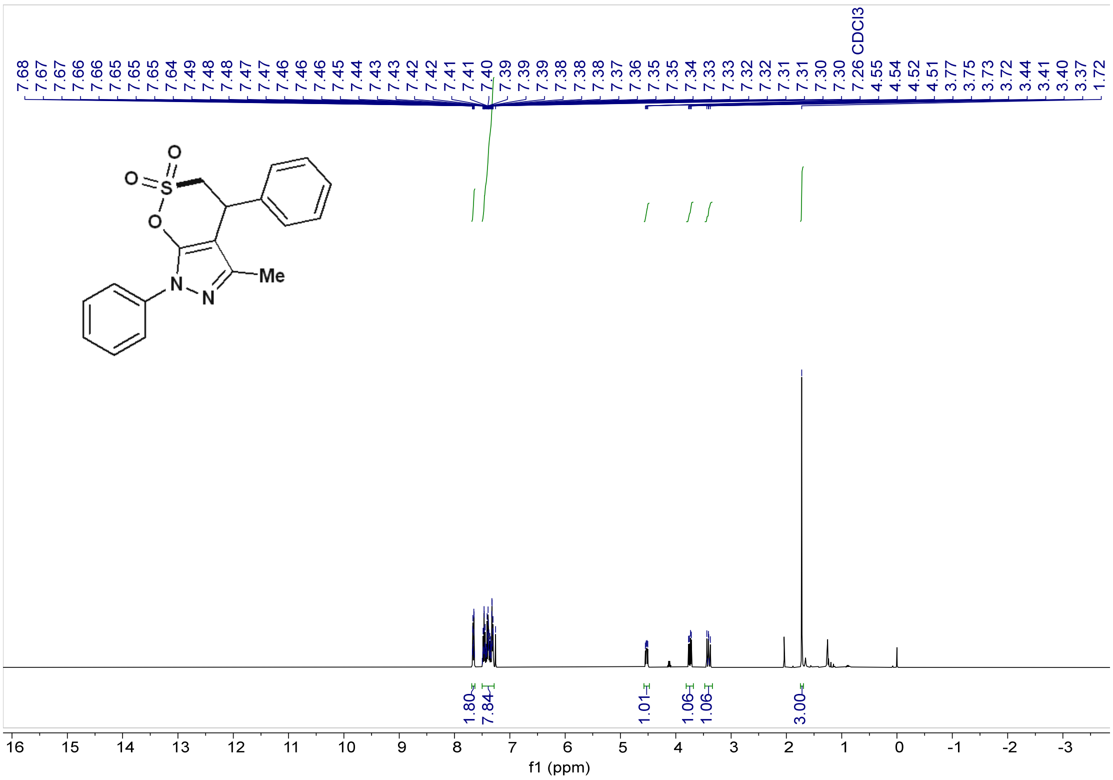

Supplementary Figure 212. ^{1}H NMR (400 MHz, room temperature, $CDCl_3$) spectra of product **10**

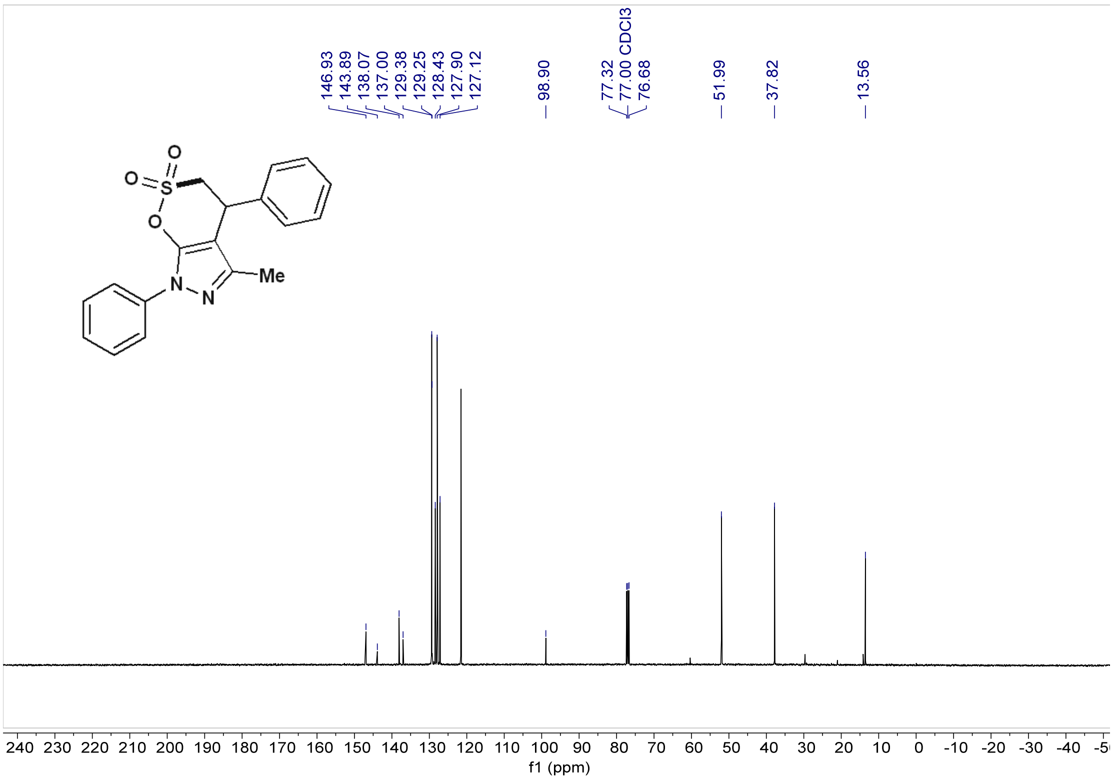

Supplementary Figure 213. ^{13}C NMR (101 MHz, room temperature, $CDCl_3$) spectra of product **10**

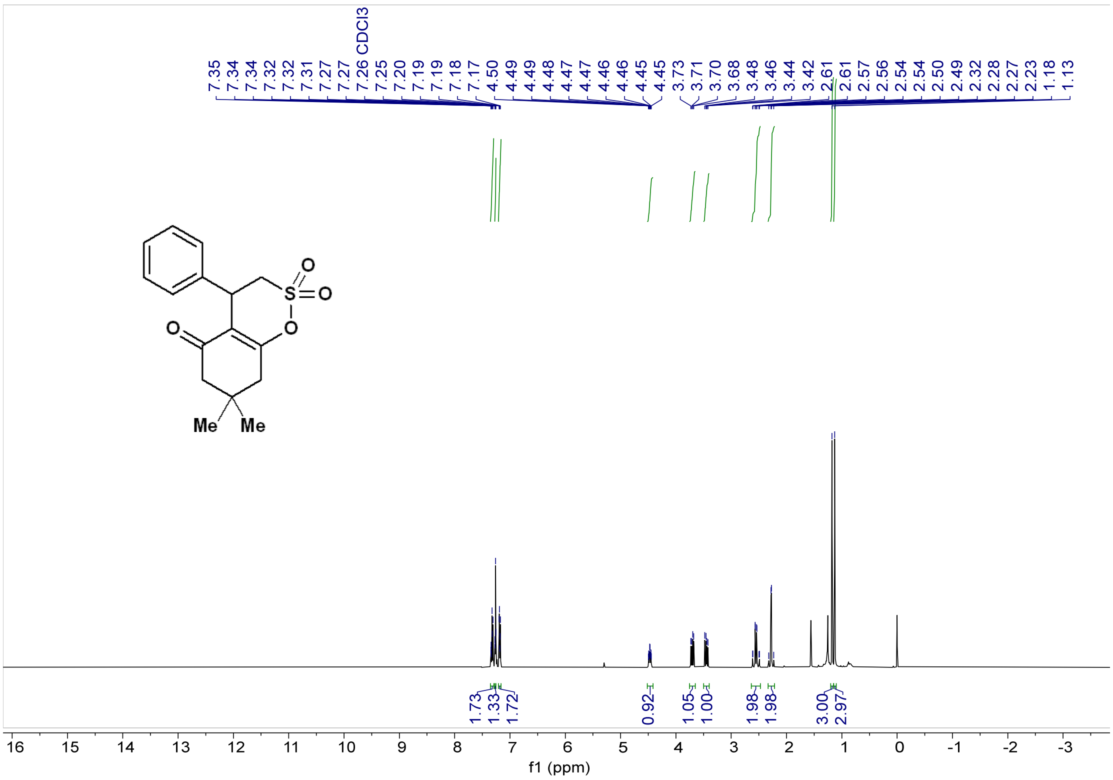

Supplementary Figure 214. ^{1}H NMR (400 MHz, room temperature, $CDCl_3$) spectra of product **12**

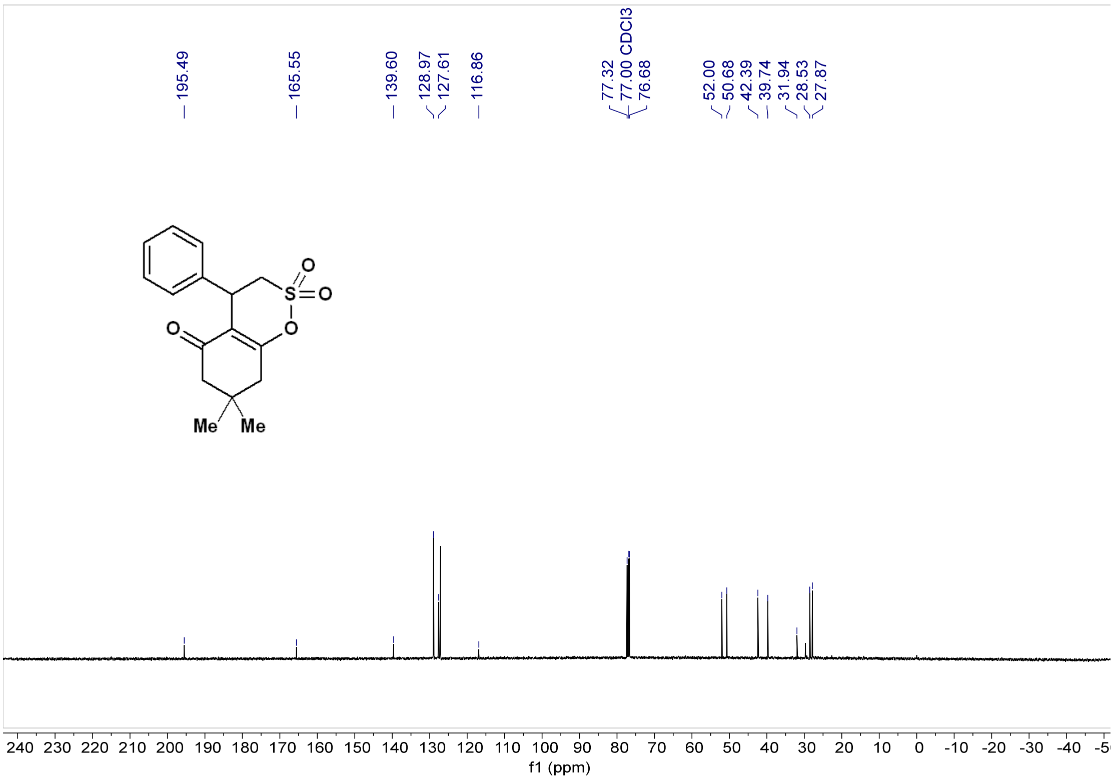

Supplementary Figure 215. ^{13}C NMR (101 MHz, room temperature, $CDCl_3$) spectra of product **12**

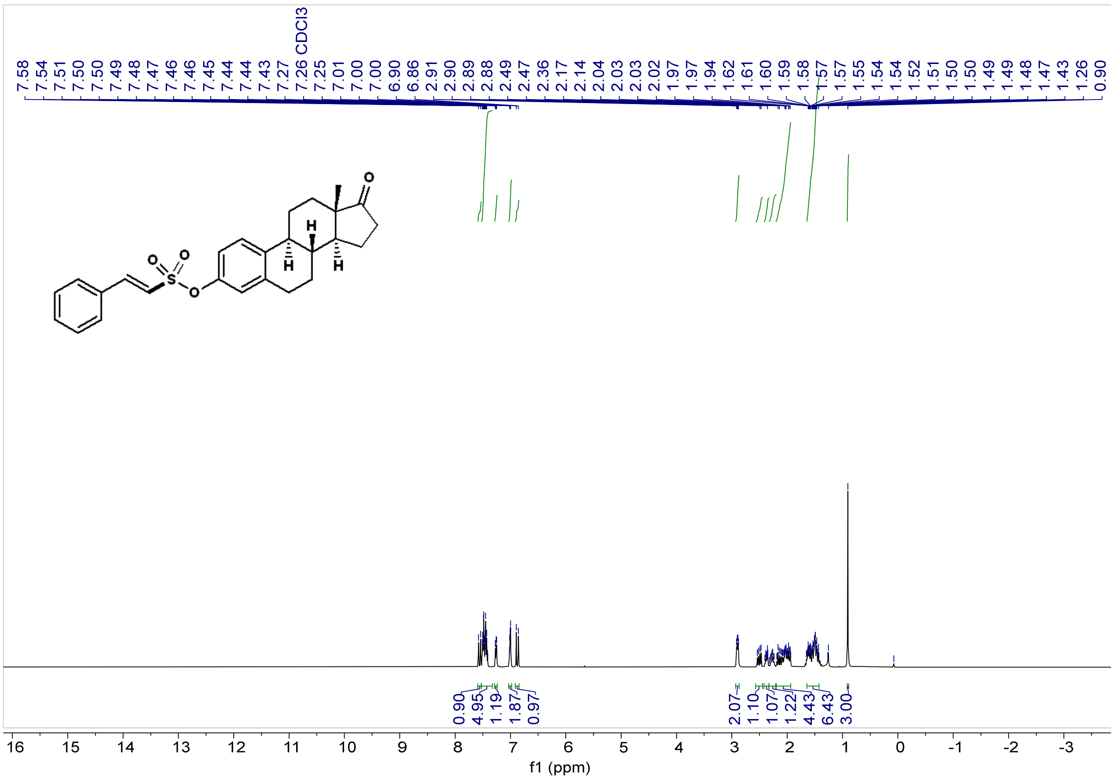

Supplementary Figure 216. ^{1}H NMR (400 MHz, room temperature, $CDCl_3$) spectra of product **14**

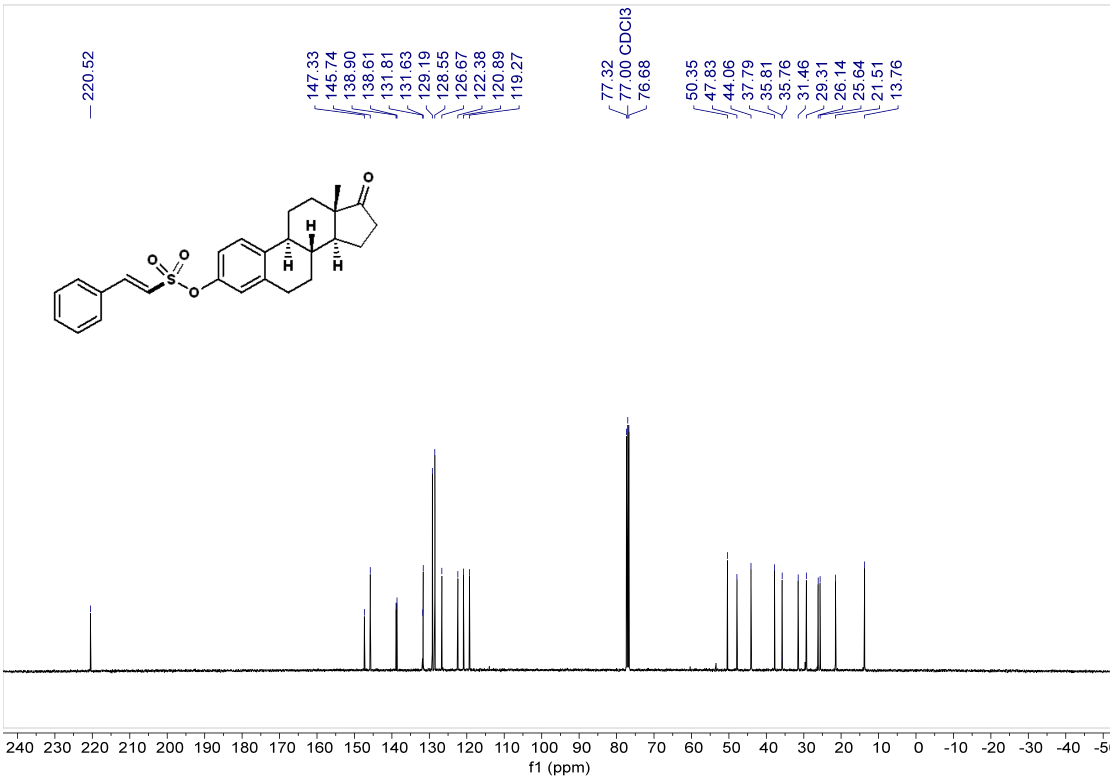

Supplementary Figure 217. ^{13}C NMR (101 MHz, room temperature, $CDCl_3$) spectra of product **14**

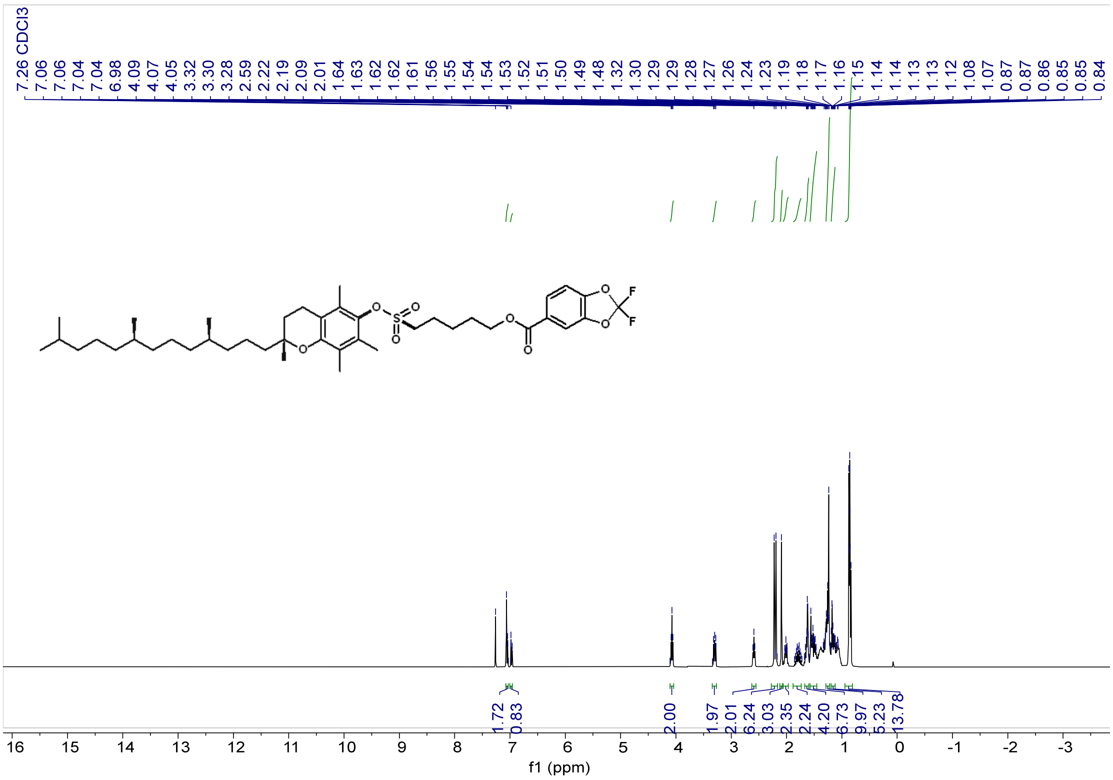

Supplementary Figure 218. ^{1}H NMR (400 MHz, room temperature, $CDCl_3$) spectra of product **16**

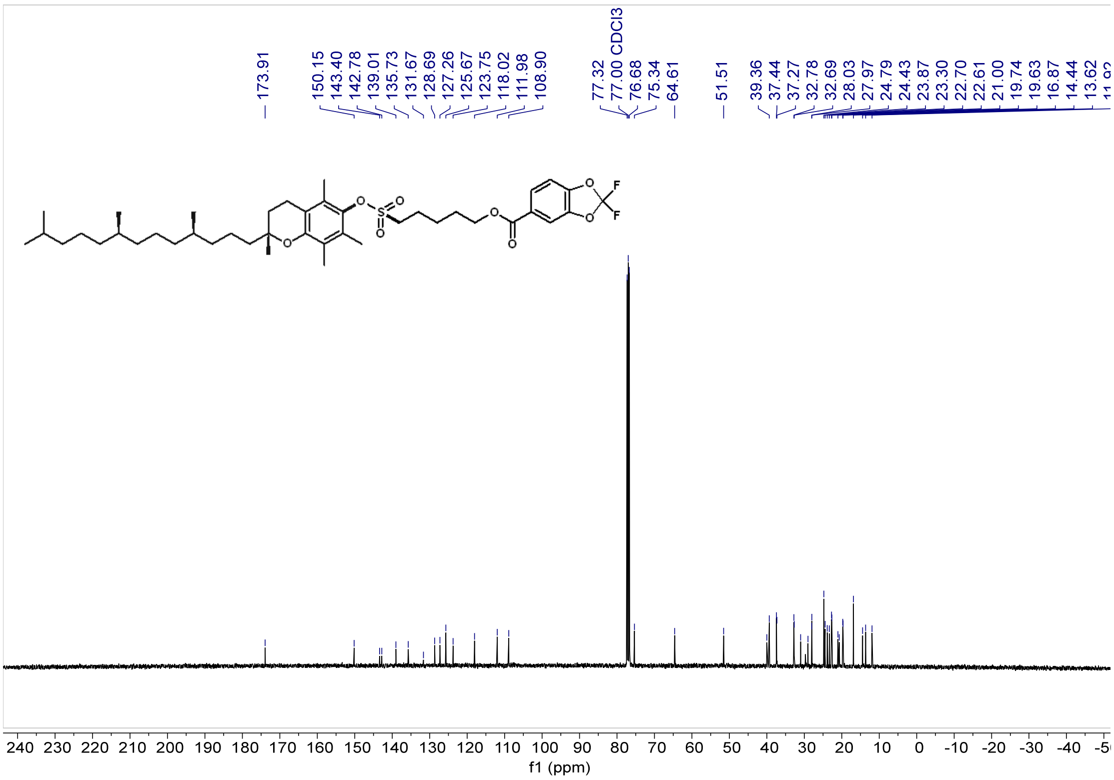

Supplementary Figure 219. ^{13}C NMR (101 MHz, room temperature, $CDCl_3$) spectra of product **16**

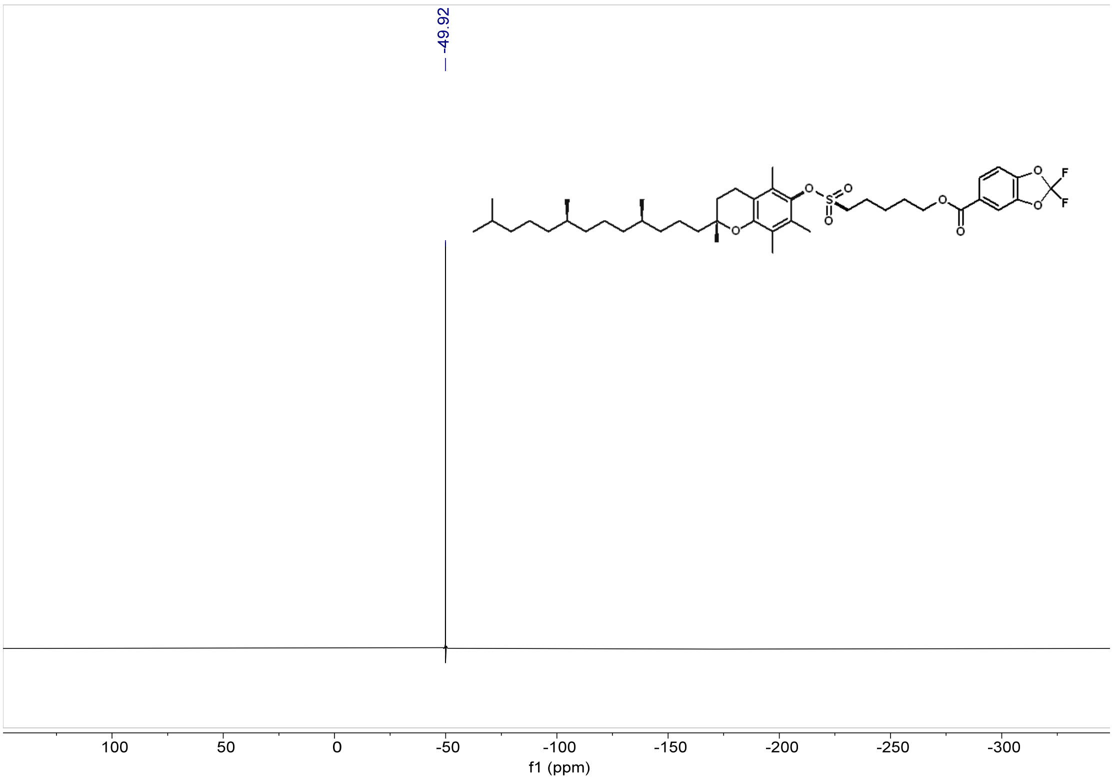

Supplementary Figure 220. ^{19}F NMR (376 MHz, room temperature, $CDCl_3$) spectra of product **16**

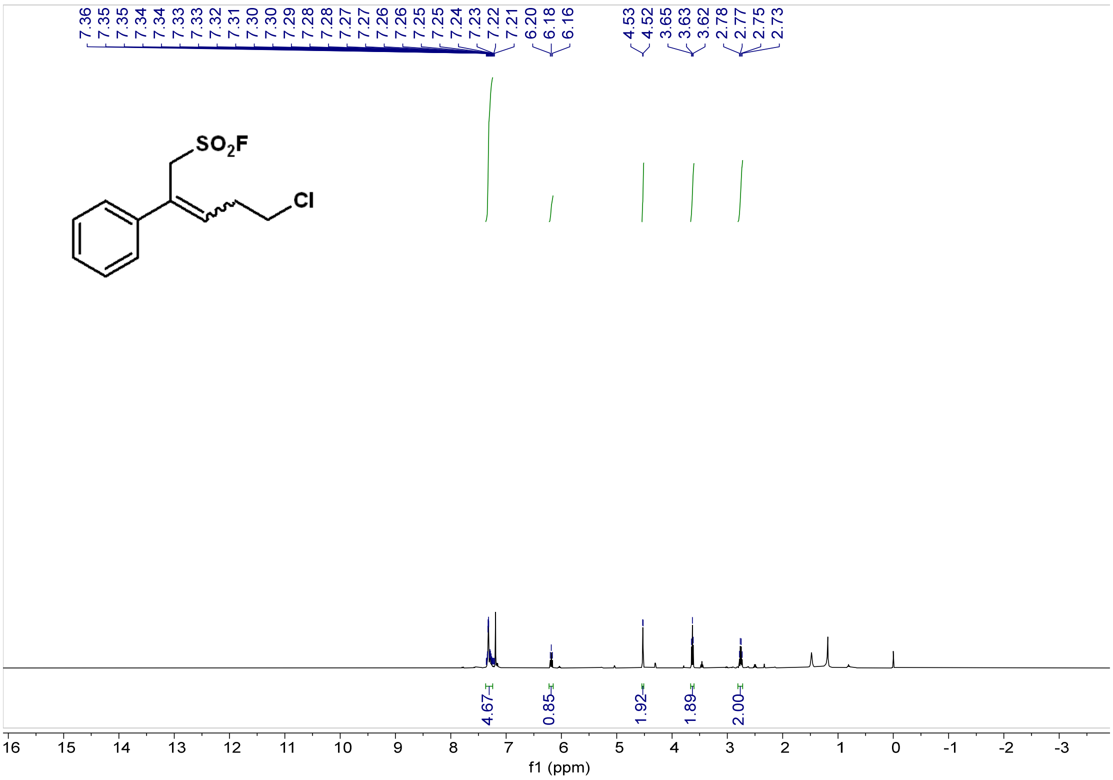

Supplementary Figure 221. ^{1}H NMR (400 MHz, room temperature, $CDCl_3$) spectra of product **19**

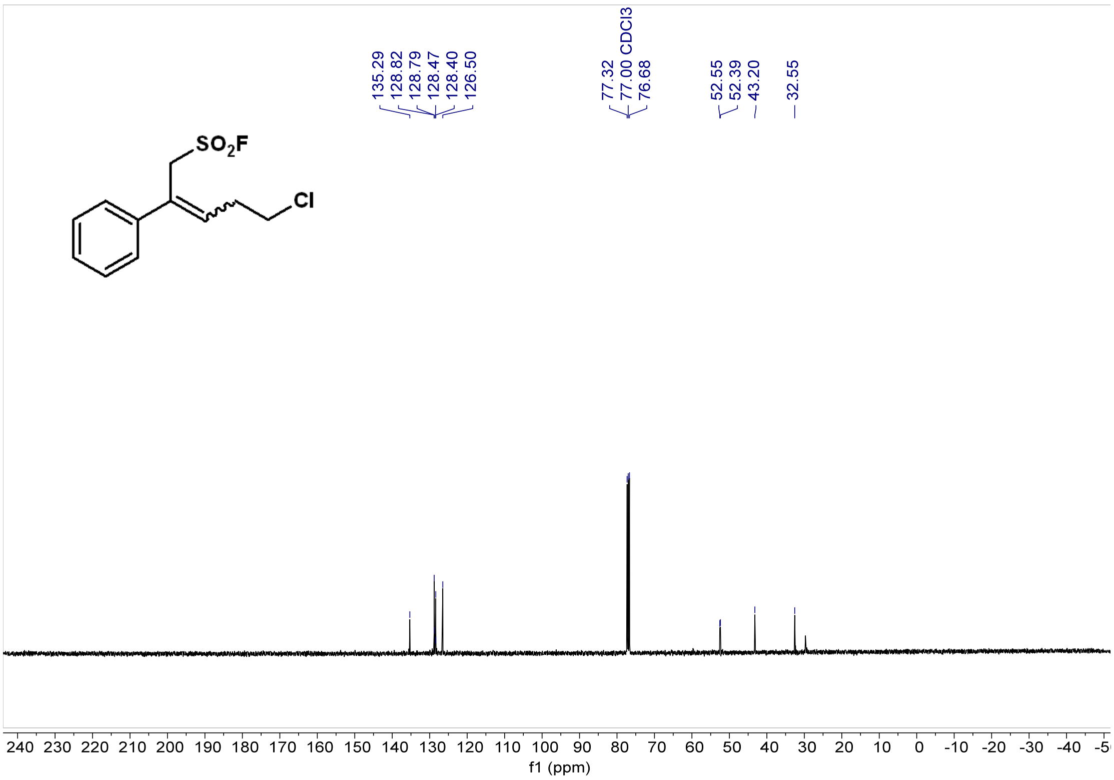

Supplementary Figure 222. ^{13}C NMR (101 MHz, room temperature, $CDCl_3$) spectra of product **19**

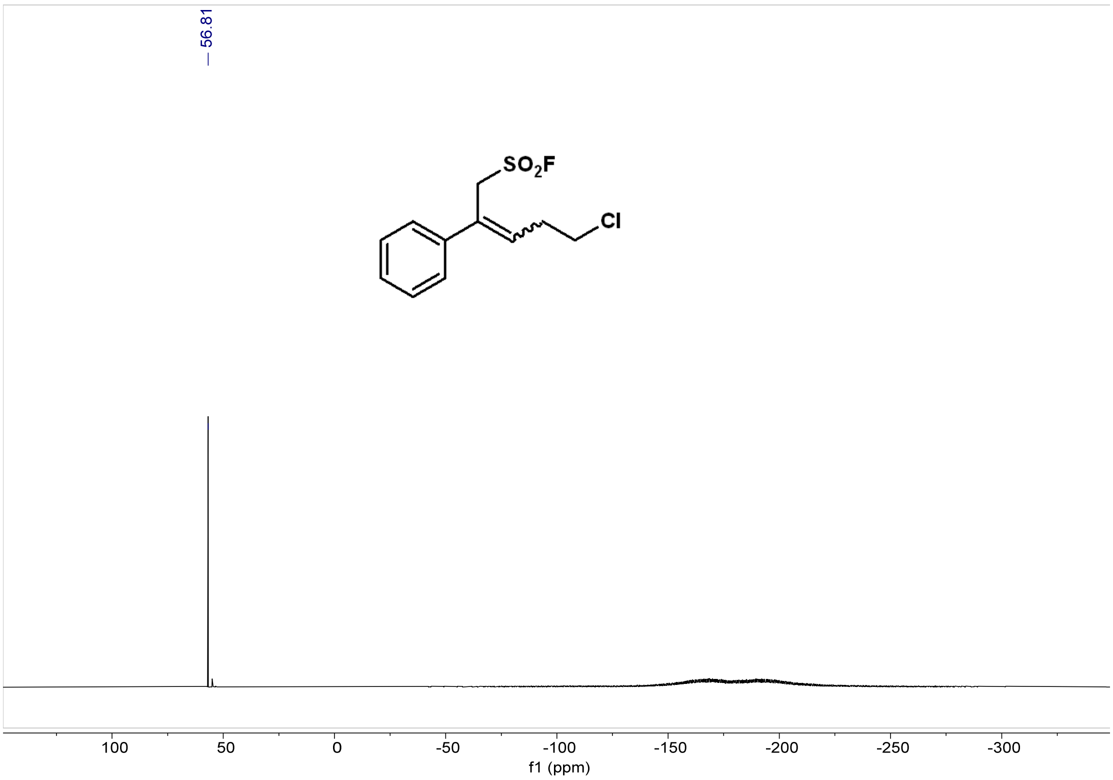

Supplementary Figure 223. ^{19}F NMR (376 MHz, room temperature, $CDCl_3$) spectra of product **19**

XII. References

1. K. Tang, Y. Chen, J. Guan, Z. Wang, K. Chen, H. Xiang, et al. *Org. Biomol. Chem.* ***2021**, 19,* 7475
2. T. Guo, G. Meng, X. Zhan, Q. Yang, T. Ma, L. Xu, et al. *Angew. Chem. Int. Ed.* ***2018**, 57,* 2605-2610
3. W. Zhang, Z. Zou, Y. Wang, Y. Wang, Y. Liang, Z. Wu, et al. *Angew. Chem. Int. Ed.* ***2019***, 58, 624-627
4. Z. Zou, W. Zhang, Y. Wang, L. Kong, G. Karotsis, Y. Wang, et al. *Org. Lett.* ***2019**, 21*, 1857−1862
